# Chemo-, Regio-, and Stereoselective *cis*-Hydroboration of 1,3-Enynes: Copper-Catalyzed Access to (*Z,Z*)- and (*Z,E*)-2-Boryl-1,3-dienes

**DOI:** 10.1021/acs.orglett.4c01929

**Published:** 2024-07-17

**Authors:** Nicklas W. Buchbinder, Long H. Nguyen, Owen N. Beck, Andrew D. Bage, Carla Slebodnick, Webster L. Santos

**Affiliations:** Department of Chemistry, Virginia Tech, 900 West Campus Drive, Blacksburg, Virginia 24061, United States

## Abstract

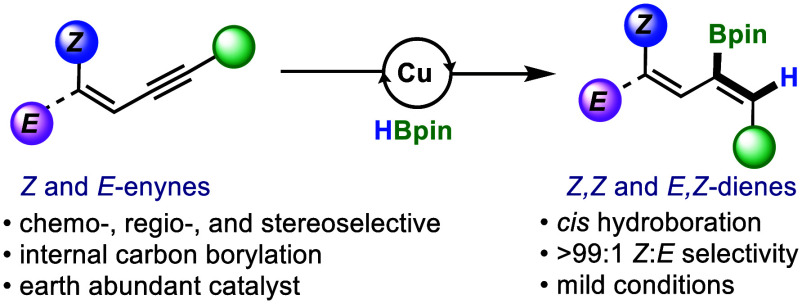

A copper-catalyzed alkyne-selective hydroboration of 1,3-enynes is disclosed, providing access to the previously elusive 2-boryl-1,3-dienes. Using CuOAc, Xantphos, and HBpin, Bpin was installed on the internal carbon of a series of symmetric and nonsymmetric 1,3-enynes, affording products with excellent *Z*:*E* selectivity. The utility of the 2-boryl-1,3-diene products was demonstrated by transformation to useful functional groups.

The importance of organoboron compounds as valuable synthetic intermediates is exemplified by their widespread adoption in organic chemistry and their convenient transformation into other functional groups.^1−3^ This versatility underpins the value of the novel installation of boron moieties into organic molecules with a precise stereochemical arrangement. Furthermore, incorporation of boron functionalities in medicinal and materials chemistry is of increasing prominence, with boronic acids and oxaboroles used as new pharmacophores,^4,5^ biological systems imaged with boron-based fluorescent probes,^6^ and boron-doped materials employed in optoelectronics.^7^ Organic π-conjugated frameworks display favorable photochemical properties that have been applied to organic electroluminescent (EL) devices and fluorescent probes.^8^ Thus, stereoselective syntheses of diverse π-conjugated frameworks warrant further investigation.^9^

Selective hydroboration reactions have garnered significant attention in recent years.^10−14^ Highly unsaturated molecules, such as 1,3-diynes, have been subjected to hydroboration reactions;^15−23^ however, 1,3-enynes remain challenging due to the array of products possible from chemo-, regio-, and stereoselectivity issues. Methods to prepare boryl-1,3-dienes include both transition metal catalysis and stoichiometric methods.^24,25^ Yamanaka and Nagasawa showed that tri- and tetrasubstituted borylallenes can undergo 1,3-boryl shift in the presence of organolithium reagents to form 2-boryl-1,3-dienes.^24^ Alternatively, the hydroboration of 1,3-enynes has the potential for the synthesis of a broader range of boryldienes; however, taming such reactions has proven difficult.^26^ Elegant work by Liu and co-workers reported the palladium-catalyzed *trans*-hydroboration of 1,3-enynes with HBcat and a designer ligand, giving 1-boryl-1,3-dienes.^25^ Recently, a cobalt-hydride catalyst was used to generate 1-boryl-1,3-dienes from 1,3-enynes.^23^

Copper catalysis is an attractive option for hydroboration reactions, as copper-boryl and copper hydride species are readily formed from simple reagents and can insert across unsaturated bonds in a *syn* fashion.^21 ,26 ,27^ Enantioenriched allenylboronates have been prepared from the copper hydride-catalyzed hydroboration of 1,3-enynes (Scheme 1a).^28−30^ In contrast, 2-boryl-1,3-dieneoates are generated when enynoates are used as substrates in a copper-catalyzed hydroboration reaction. However, a polarized activating group from the ester functionality is required for reactivity (Scheme 1b).^31^ Ito developed a copper boryl-catalyzed chemodivergent method for the synthesis of 1-boryl-1,3-dienes or 3-alkynylboronates from 1,3-enynes (Scheme 1c).^26^ Steric effects between the ligand and substrate determined the chemoselectivity of this reaction, as a less sterically encumbered copper hydride catalyst might react more smoothly than the copper-boryl counterpart. However, there is only one example of enyne hydroboration where 2-boryl-1,3-diene was observed with modest selectivity and is an outlier in their study. Given this background, methods to access 2-boryl-1,3-dienes are severely lacking, notwithstanding hydroboration of nonpolarized 1,3-enynes. Inspired by previous copper-catalyzed hydroboration methods, we envisioned using a copper catalyst to generate 2-boryl-1,3-dienes from 1,3-enynes (Scheme 1d). A major challenge in our studies is the identification of conditions that provide 2-boryl-1,3-dienes with the requisite regio- and stereoselectivity while avoiding alkene hydroboration, [1,3]-sigmatropic shift resulting in allenylboronates, and overhydroboration.^11^ Herein, we report a copper-catalyzed (*Z*)-3,4-selective hydroboration of 1,3-enynes with HBpin.

**Scheme 1:**
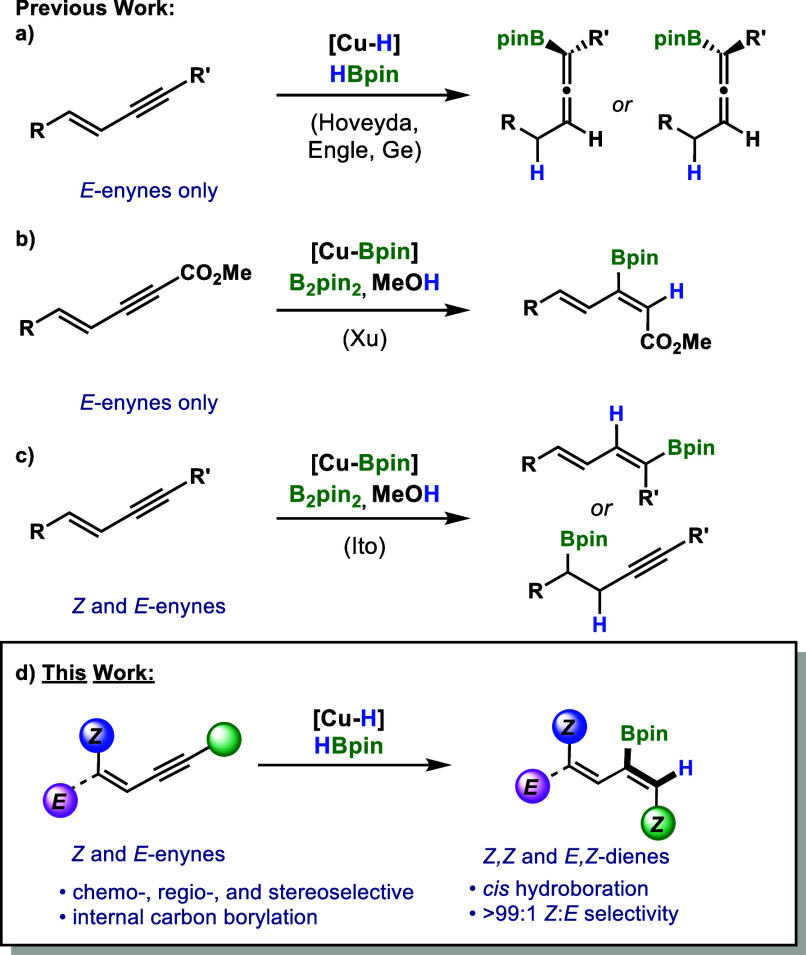
Copper-Catalyzed 1,3-Enyne Hydroboration Reactions

Investigations into selective formation of 2-boryl-1,3-dienes from 1,3-enynes began by treating the model substrate (*Z*)-but-1-en-3-yne-1,4-diyldibenzene (**1a**) with CuOAc (10 mol %), Xantphos (10 mol %), and HBpin (2.0 equiv) in toluene at 50 °C for 16 h (Table 1, entry 1). Interestingly, hydroboration occurred on the alkyne moiety in a *cis* fashion with Bpin installed on the internal carbon, proving access to these elusive conjugated vinyl boronates. Thus, (*Z*,*Z*)-2-boryl-1,3-diene **2a** was isolated in 75% yield with >99:1 *Z*:*E* selectivity. Stereochemistry was established by protodeborylation with KHF_2_ where the alkene coupling constants of 9.4 Hz were consistent with the *Z* geometry. We next tested alternative Cu(I) and Cu(II) salts, but these displayed decreased yields of **2a** (entries 2–5). The use of the monodentate phosphine ligand triphenylphosphine or bidentate ligand (oxybis(2,1-phenylene))bis(diphenylphosphane) (DPEphos) had a negative impact on the isolated yield and *Z*:*E* ratio (entries 6, 7). A survey of solvents such as tetrahydrofuran (THF), 1,4-dioxane, or acetonitrile (MeCN) decreased the yield (entries 8–10). Catalyst loading was also evaluated; interestingly, a reduced loading (5 mol %) of the copper precatalyst (CuOAc) and ligand (Xantphos) resulted in an increase in yield of **2a** (80%), possibly due to the decreased amount of sacrificial HBpin in formation of the active copper hydride catalyst (entry 11) (*vide infra*). We next investigated the effect of the temperature and found that the reaction was optimal when performed at 40 °C (entries 12–14). Control reactions without catalyst or ligand gave no product formation, suggesting that both are essential for reactivity (entries 15, 16). To ensure hidden borane catalysis was not occurring,^32^ substoichiometric TMEDA was introduced to the reaction, and no borane-TMEDA adduct was observed (see Supporting Information). The optimized conditions were found to be copper(I) acetate (5 mol %), Xantphos (5 mol %), and HBpin (2.0 equiv) in toluene at 40 °C for 16 h, which provided **2a** in an 89% isolated yield and >99:1 Z:*E* ratio (entry 13).

**Table 1:**
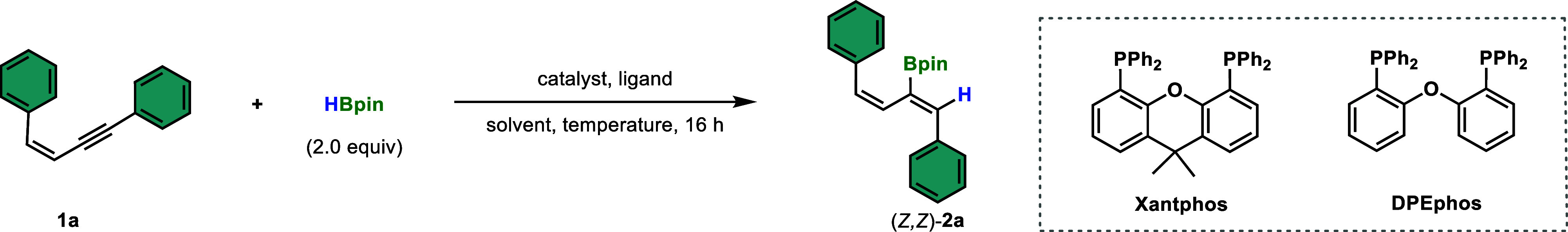
Optimization of Reaction Conditions^a^

Entry	Catalyst	Ligand	Cat. Loading (mol %)^b^	Solvent	Temp (°C)	Yield (%)	*Z*:*E*^c^
1	CuOAc	Xantphos	10	toluene	50	75	>99:1
2	CuCN	Xantphos	10	toluene	50	8	>99:1
3	CuCl	Xantphos	10	toluene	50	4	>99:1
4	Cu(OAc)_2_	Xantphos	10	toluene	50	55	>99:1
5	CuCl_2_	Xantphos	10	toluene	50	4	>99:1
6	CuOAc	PPh_3_	10	toluene	50	25	96:4
7	CuOAc	DPEphos	10	toluene	50	13	76:24
8	CuOAc	Xantphos	10	THF	50	53	>99:1
9	CuOAc	Xantphos	10	MeCN	50	13	>99:1
10	CuOAc	Xantphos	10	1,4-dioxane	50	72	>99:1
11	CuOAc	Xantphos	5	toluene	50	80	>99:1
12	CuOAc	Xantphos	5	toluene	60	80	>99:1
13	CuOAc	Xantphos	5	toluene	40	89	>99:1
14	CuOAc	Xantphos	5	toluene	25	80	>99:1
15		Xantphos	10	toluene	50	0	
16	CuOAc		10	toluene	50	0	

a Performed under a N_2_ atmosphere, 0.125 mmol scale, 0.25 M, isolated yield.

b Refers to copper salt and ligand loading.

c Determined by GC-MS.

With the reaction conditions optimized, our focus turned to probing the selectivity of the transformation by the completion of a substrate scope (Schemes 2 and 3). The model substrate **1a** underwent hydroboration to give **2a** in good yield (73%) (Scheme 2). Methyl substitution on the aryl rings was well tolerated in both the *para* (**2b**) and *meta* positions (**2c**) with 78% and 69% isolated yields, respectively. The (*Z*,*Z*)-configuration of **2b** (CCDC 2297662) was unambiguously confirmed by X-ray crystallography.^33^ Larger alkyl substitutions such as *tert*-butyl and *n*-propyl reacted smoothly into their corresponding 2-boryldienes (**2d**,**e**) with comparable yields. Likewise, the presence of methoxy groups in either the *para* (**2f**) or *meta* (**2g**) positions resulted in excellent yields. Performing the reaction on a 1.6 mmol scale resulted in a good yield (65%) of boryldiene **2f** without affecting selectivity. Benzyl-protected substrate **1h** was tolerated under the reaction conditions, forming 2-boryl-1,3-diene **2h** in good yield (61%), while the thioether 2-boryldiene **2i** was successfully prepared in a similar yield (58%). Boryldienes bearing electron-withdrawing functionalities such as trifluoromethyl (**2j**) and trifluoromethoxy (**2k**) were obtained in moderate yields. Halogen-containing substrates such as fluoro in the *meta* (**2m**) and *para* (**2l**) positions served as good substrates, while chloro substitution in the *ortho* (**2p**), *meta* (**2o**), and *para* (**2n**) positions resulted in the formation of the corresponding boryldienes in moderate to good yields (34%, 55%, 65%), with increasing yields in the order of *ortho* to *meta* and *para*, suggesting steric hindrance at the *ortho* position may affect reactivity. Heterocycles were also tested under the reaction conditions. A boryldiene bearing a thiophene moiety (**2q**) was obtained in a good yield (58%).

**Scheme 2:**
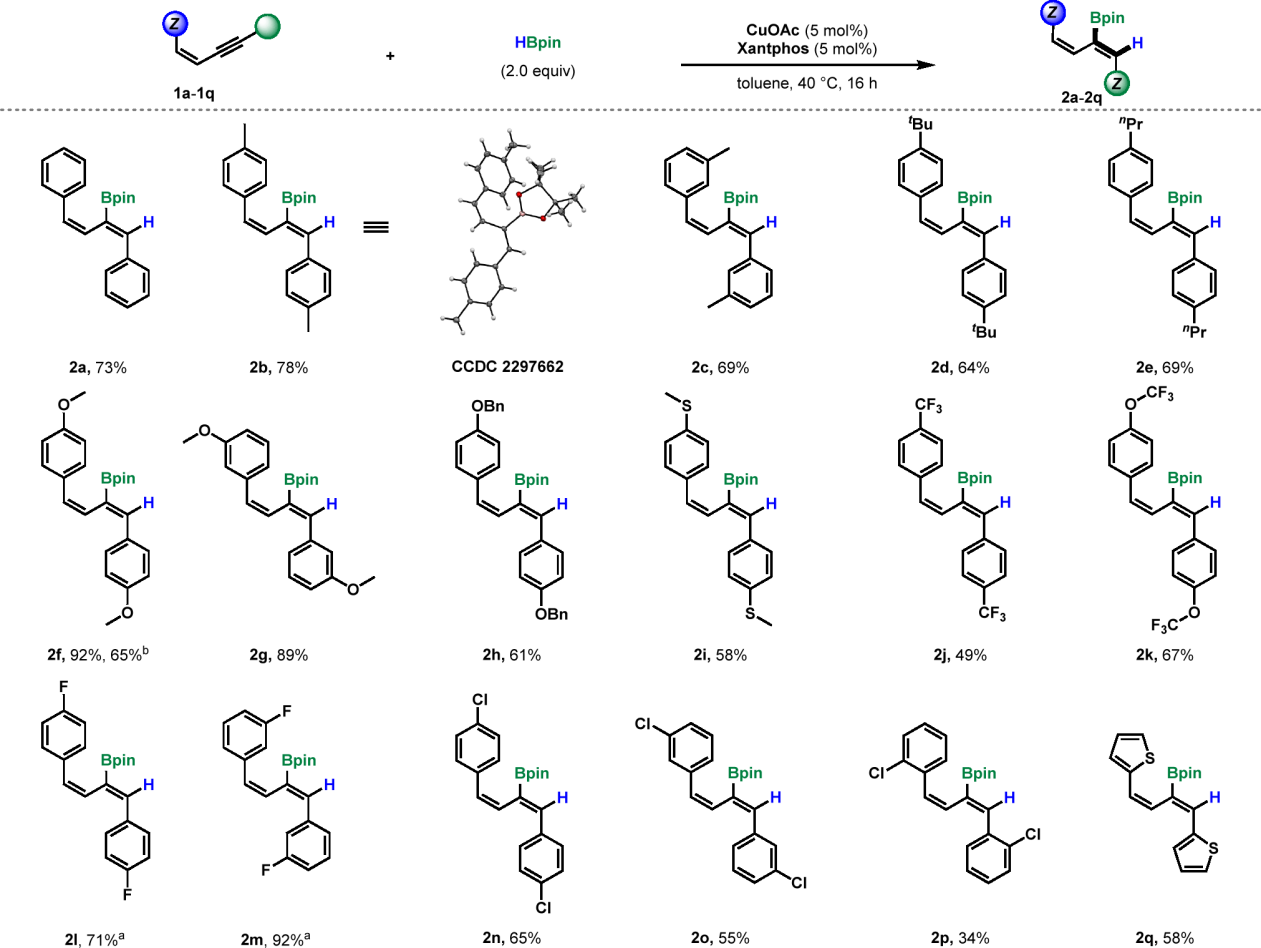
Substrate Scope of (*Z*)-1,3-Enynes Isolated yields are reported. >99:1 *Z*:*E* determined by ^1^H NMR. ^a^1.2 equiv HBpin, 4 h. ^b^1.6 mmol scale.

**Scheme 3:**
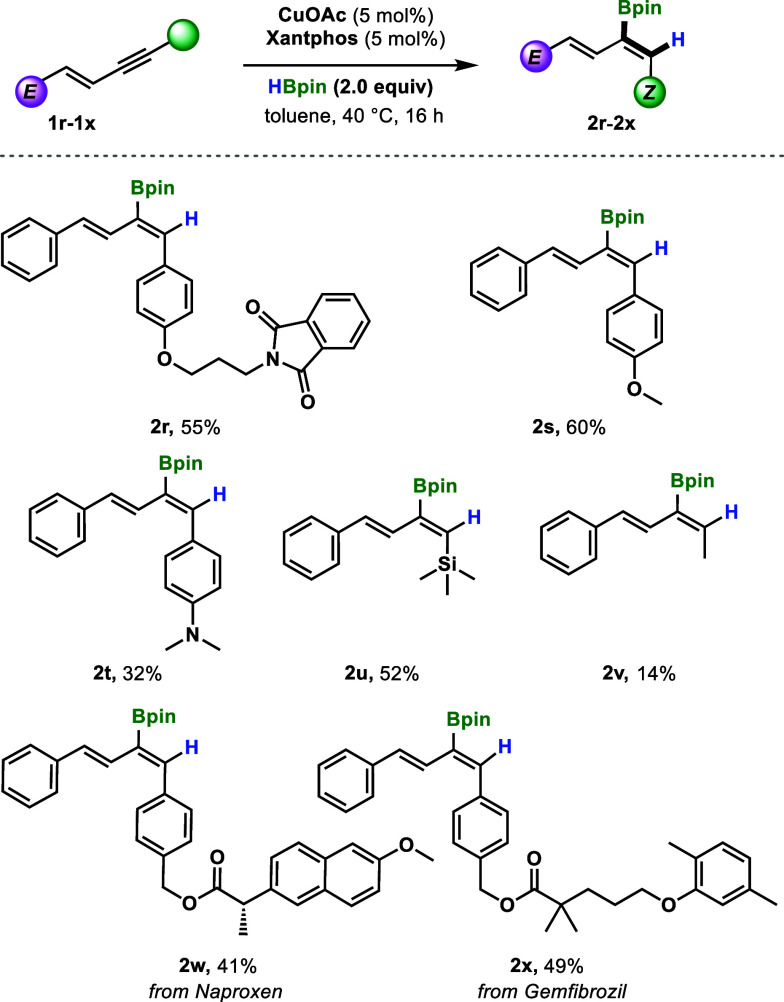
Substrate Scope of (*E*)-1,3-Enynes Isolated yields are reported. >99:1 *Z*:*E* determined by ^1^H NMR.

With the substrate scope of the (*Z*)-enynes established, we next investigated the complementary (*E*)-enynes as substrates (Scheme 3). Thus, we synthesized unsymmetrical phthalimide-bearing enyne **1r**. To our delight, the corresponding boryldiene **2r** was afforded exclusively and the Bpin moiety was chemoselectively added to the alkyne unit (55% yield). The anisyl-containing boryldiene **2s** was isolated in 60% yield. Likewise, boryldiene bearing a tertiary aniline **2t** was obtained in a 32% yield as a single isomer. Monoaryl, unsymmetrical substrates were tested next. A trimethylsilyl-capped enyne (**1u**) underwent hydroboration to give the corresponding 2-boryldiene in 52% yield. While most alkyl substrates resulted in a complex mixture of products (see Supporting Information), boryldiene **2v** was isolated in a 14% yield as a single isomer. Finally, enyne derivatives of the nonsteroidal anti-inflammatory drug naproxen and lipid lowering agent gemfibrozil were synthesized (**1w**, **1x**) and subjected to the optimized conditions to give boryldienes **2w** and **2x** in moderate yields. These results demonstrate chemoselectivity for the alkyne in substrates bearing carbonyls, as no reduction of the amide (**1r**) or esters (**1w**, **1x**) was observed.

To demonstrate the synthetic potential of the 2-boryl-1,3-dienes, compound **2f** was subjected to various transformations (Scheme 4). First, boronic ester **2f** underwent protodeboration using KHF_2_ and acetic acid to produce the (*Z*,*Z*)-disubstituted 1,3-diene **3** with >99:1 *Z*:*E* retention of stereochemistry. Suzuki–Miyaura cross-coupling was performed with Pd_2_(dba)_3_ and 4-iodobenzonitrile to afford the trisubstituted diene **4** in 74% yield. Oxidation with hydrogen peroxide afforded α,ß-unsaturated ketone **5** in good yield (54%) with concomitant alkene isomerization to the more stable (*E*)-geometry. Finally, under Matteson homologation conditions, 2-boryl-1,3-diene **2f** underwent stereoretentive homologation to generate allylic alcohol **6**.

**Scheme 4:**
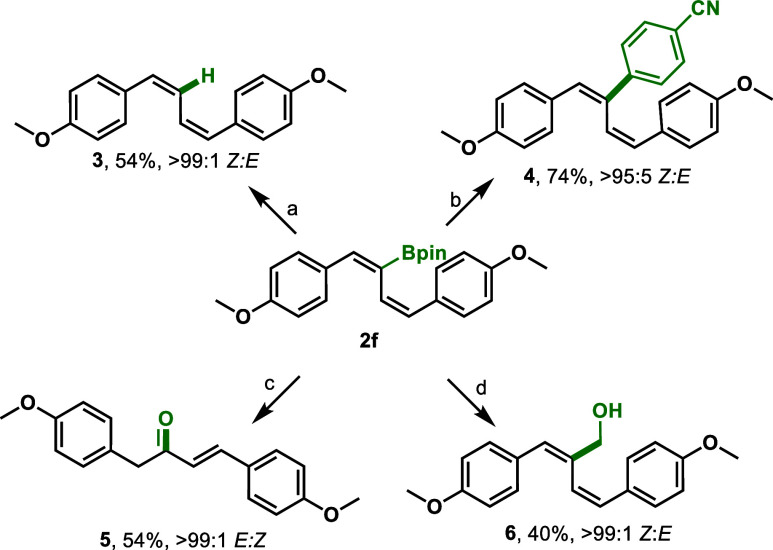
Synthetic Applications of 2-Boryl-1,3-Dienes Reaction conditions: a: KHF_2_ (3.0 equiv), acetic acid (0.5 mL). b: Pd_2_(dba)_3_ (4 mol %), SPhos (5 mol %), 4-iodobenzonitrile (1.5 equiv), THF/3 M NaOH (3:1), 70 °C. c: H_2_O_2_ (30 equiv), 3 M NaOH (30 equiv), THF (0.5 mL). d: *n*BuLi (3.0 equiv), dibromomethane (4.0 equiv), THF (1.0 mL) −78 to 25 °C, 4 h then H_2_O_2_ (5.0 equiv), 3 M NaOH (5.0 equiv).

In conclusion, we have developed a copper-catalyzed method for the *cis*-alkyne-hydroboration of 1,3-enynes. Notably, Bpin was installed on the internal carbon, which was previously elusive. This protocol utilizes commercially available reagents and mild conditions to achieve moderate to excellent yields with excellent chemo-, regio-, and stereoselectivity. Both (*Z*)- and (*E*)-1,3-enynes were successfully converted to their corresponding 2-boryl-1,3-dienes. The synthetic utility of the 2-boryl-1,3-dienes was showcased through further functionalization, undergoing exemplar reactions including oxidation, protodeborylation, homologation, and cross-coupling. The selectivity and the mechanistic intricacies of this reaction are currently under investigation and will be reported in due course.

## Data Availability

The data underlying this study are available in the published article and its Supporting Information.

## Supporting Information Available

Experimental procedures, compound characterization data, crystallographic data, and copies of NMR spectra (PDF)
