# Supplementary material for: Chemo-, Regio-, and Stereoselective cis-Hydroboration of 1,3-Enynes: Copper-Catalyzed Access to (Z,Z)- and (Z,E)-2-Boryl-1,3-dienes
Source: Org Lett. 2024 Jul 17;26(29):6136–41. doi: 10.1021/acs.orglett.4c01929 (PMC11287746; doi:10.1021/acs.orglett.4c01929)
Supplement: Supplementary file 1 — ol4c01929_si_001.pdf [file ol4c01929_si_001.pdf]

Chemo-, Regio-, and Stereoselective *cis*-Hydroboration of 1,3-Enynes: Copper-catalyzed Access to (*Z,Z*)- and (*Z,E*)-2-Boryl-1,3-dienes

Nicklas W. Buchbinder, Long H. Nguyen, Owen N. Beck, Andrew D. Bage, Carla Slebodnick, and Webster L. Santos*

Department of Chemistry, Virginia Tech, 900 West Campus Drive, Blacksburg, VA 24061, USA
email: santosw@vt.edu

Table of Contents

1. General Information 3

2. General Procedures 4

   2.1 General Procedure A 4

   2.2 General Procedure B 4

   2.3 General Procedure C 5

   2.4 General Procedure D 5

   2.5 General Procedure E 6

   2.6 General Procedure F 6

   2.7 General Procedure G: 7

   2.8 Characterization of substrates **S1a**-**1x** 8

3. Synthesis of Substrate **1r** 21

   3.1 Synthesis of 2-(3-(4-iodophenoxy)propyl)isoindoline-1,3-dione (**S1r**) 21

   3.2 Synthesis of 2-(3-(4-((trimethylsilyl)ethynyl)phenoxy)propyl)isoindoline-1,3-dione (**S2r**) 22

   3.3 Synthesis of 2-(3-(4-ethynylphenoxy)propyl)isoindoline-1,3-dione (**S3r**) 23

   3.4 Synthesis of (*E*)-2-(3-(4-(4-phenylbut-3-en-1-yn-1-yl)phenoxy)propyl)isoindoline-1,3-dione (**1r**) 24

4. Optimization Table 25

5. Characterization of 2-Boryl-1,3-Dienes 27

6. Experimental for Applications of 2-boryl-1,3-dienes 38

   6.1 Synthesis of (1*Z*,3*Z*)-1,4-bis(4-methoxyphenyl)buta-1,3-diene 38

   6.2 Synthesis of (*E*)-1,4-bis(4-methoxyphenyl)but-3-en-2-one 39

   6.3 Synthesis of (*Z*)-2-((*E*)-4-methoxybenzylidene)-4-(4-methoxyphenyl)but-3-en-1-ol 40

   6.4 Synthesis of 4-((1*E*,3*Z*)-1,4-bis(4-methoxyphenyl)buta-1,3-dien-2-yl)benzonitrile 41

7. Hidden Borane Catalysis Experiment 42

8. Crystallographic Data 43

9. Failed Substrates 48

10. Spectra 49

11. References 134

1. General Information

Materials

Chemicals were obtained from commercial sources (Sigma Aldrich, Fisher Scientific, Acros Organics, Ambeed Inc., Oakwood Chemical, Combi-Blocks) unless otherwise noted. Anhydrous THF (Fisher), toluene (Fisher), dichloromethane (Fisher) and acetonitrile (Fisher) were obtained from the Innovative Technology Pure SolvMD solvent purification system (drying agent: alumina; under argon atmosphere).

Methods

Reactions were performed using standard Schlenk techniques under a nitrogen atmosphere unless otherwise noted. All glassware was flame-dried before use. All reported temperatures are based upon external heating plate temperature. Room temperature was approximately 25 °C.

Column Chromatography

Flash column chromatography was performed on Teledyne ISCO CombiFlash systems, using SiliaFlash P60 40-63 μm, 60 Å. TLC analyses were performed using Silicycle aluminum backed silica gel F-254 plates.

NMR Spectroscopy

NMR spectroscopic experiments were performed using Agilent 400-MR 400 MHz, Agilent U4-DD2 400 Hz and Bruker Avance II 500 MHz spectrometers. Chemical shifts are reported in δ ppm, coupling constants, *J*, are reported in Hz (to 1 decimal place). ^{1}H NMR spectra are referenced to the residual protonated solvent signal ($CDCl_3$, 7.26 ppm; CD_3OD, 3.31 ppm; DMSO-d_6, 2.50 ppm; acetone-d_6, 2.05 ppm) and ^{13}C NMR spectra are referenced to the deuterated solvent signal ($CDCl_3$, 77.16 ppm; CD_3OD, 49.00 ppm; DMSO-d_6, 39.52 ppm; acetone-d_6; 29.84 ppm) and are ^{1}H decoupled. Signal multiplicities reported as singlet (s), doublet (d), triplet (t), quartet (q), multiplet (m).

Mass Spectrometry

Mass spectrometry was performed at VT-MSI HRMS facility. High resolution mass spectra (HRMS) were recorded on Agilent 6220 using flow injection analysis (FIA). LC flow is 0.1 ml/min with a composition of 25:75 water: acetonitrile with 0.1% formic acid. Run times were 1 minute. ESI mass spectra were acquired with an Agilent 6220 LC-ESI-TOF or a Thermo Scientific Q-Exactive Orbitrap.

2. General Procedures

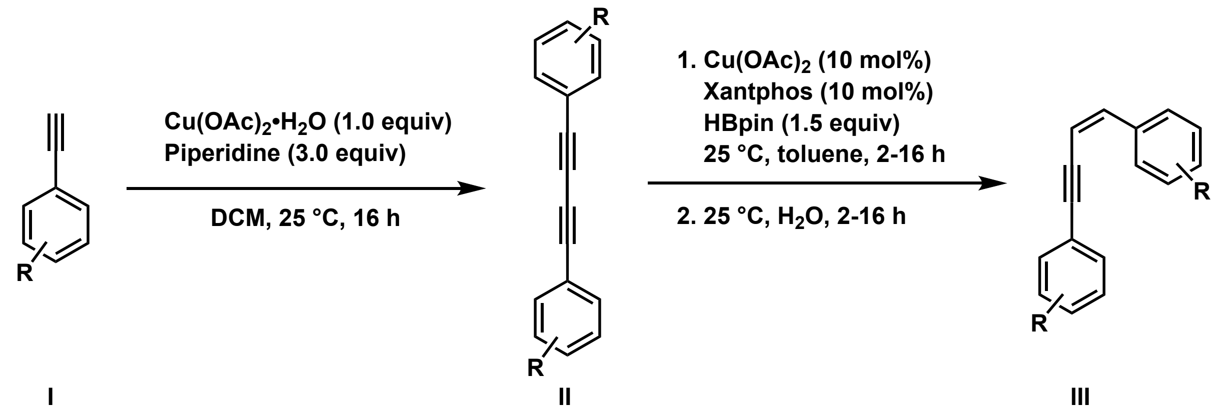

Scheme S1: Method for synthesis of enyne starting material.

2.1 General Procedure A

According to a modified procedure from Burgio *et al.*,[1] to a stirring suspension of $Cu(OAc)_2 \cdot H_2O$ (1.0 equiv, 0.300 - 2.00 mmol) in dichloromethane (0.25 M) was added acetylene (**I**) (1.0 equiv, 0.300 - 2.00 mmol) and piperidine (3.0 equiv, 0.900 - 6.00 mmol). The reaction was stirred at room temperature overnight. Reaction progress was followed via TLC. Upon completion, the reaction mixture was concentrated *in vacuo* and purified by flash column chromatography to afford the corresponding diyne (**II**).[1]

2.2 General Procedure B

According to a modified procedure from Burgio *et al.*,[1] to a stirring solution of diyne (**II**) (1.0 equiv, 0.25 - 2.00 mmol), Cu(OAc) (10 mol%, 0.03 - 0.20 mmol) and Xantphos (10 mol%, 0.03 - 0.20 mmol) in toluene (0.25 M) was added pinacolborane (1.5 equiv, 0.375 - 3.00 mmol) under a nitrogen atmosphere. The reaction was stirred at room temperature, and reaction progress was followed by TLC. Upon completion, the reaction was quenched with water (5.0 equiv, 1.25 - 30.0 mmol), extracted thrice with EtOAc, concentrated *in vacuo,* and purified by flash column chromatography to afford the corresponding enyne (**III**).

2.3 General Procedure C

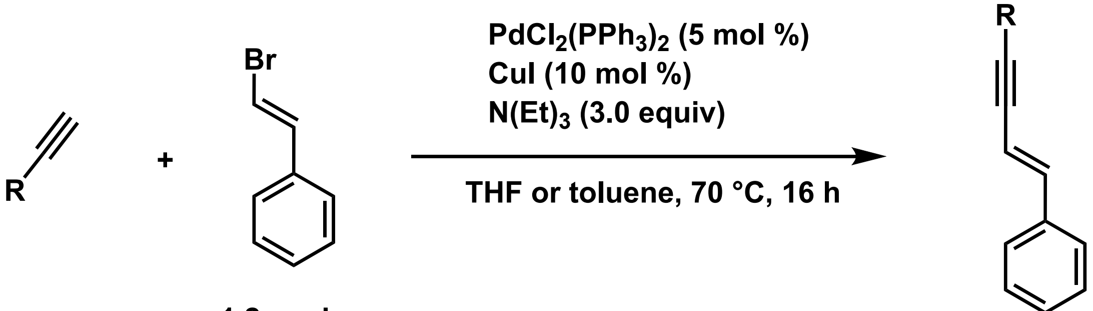

Scheme S2: Method (Sonogashira Cross-Coupling) for synthesis of enyne starting material.

According to a modified procedure from Wang *et al.*[2] To a 6-dram vial with screw on septa cap: a stir bar, acetylene (1.0 equiv, 0.30 - 1.09 mmol), $PdCl_2(PPh_3)_2$ (5 mol%,0.02 - 0.05 mmol) and CuI (10 mol%, 0.03 -0.10 mmol) were added. The reaction flask was put under inert atmosphere (N_2) using standard *Schlenk* technique. Toluene or THF (0.25 M), (*E*)-(2-bromovinyl)benzene (1.2 equiv, 0.36 - 1.30 mmol) and triethylamine (3.0 equiv, 0.9 - 3.27 mmol) were then added to the reaction flask. The reaction was heated to 70 °C and allowed to stir for 16 hours. The reaction mixture was reduced under negative pressure and purified via flash column chromatography to afford the corresponding enyne.[2]

2.4 General Procedure D

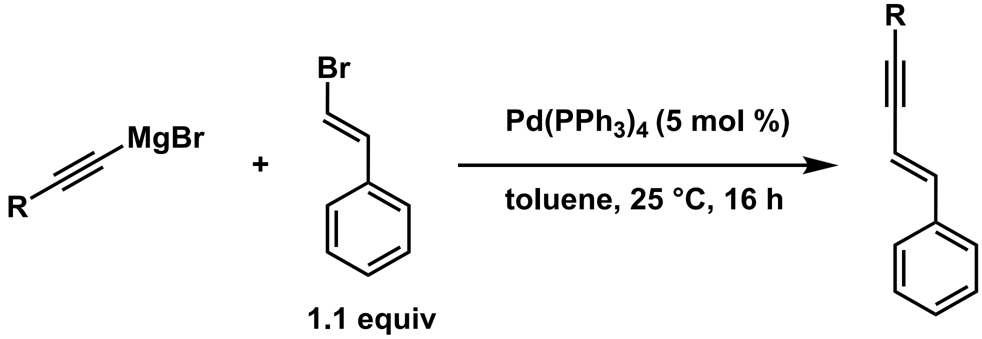

Scheme S3: Method (Kumada Cross-Coupling) for synthesis of enyne starting material.

According to a modified procedure from Wang *et al.*[2] To a 6-dram vial with screw on septa cap: a stir bar and Pd $(PPh_3)_4$ (5 mol%, 0.05 mmol) were added. The reaction flask was put under inert atmosphere (N_2) using standard *Schlenk* technique. Toluene (0.5 M), (*E*)-(2-bromovinyl)benzene (1.1 equiv, 1.20 mmol) and 1-propynylmagnesium bromide (0.5 M in THF) (1.0 equiv, 1.09 mmol) were then added to the reaction flask. The reaction was allowed to stir for 16 hours. The reaction mixture was reduced under negative pressure and purified via flash column chromatography to afford the corresponding enyne.[3]

2.5 General Procedure E

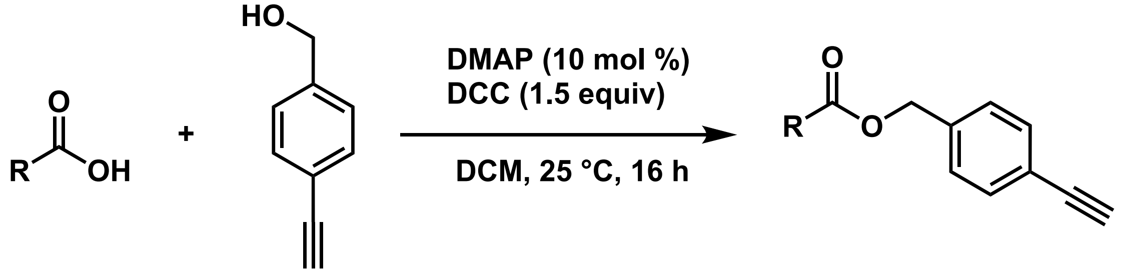

I II

Scheme S4: Method (DCC peptide coupling) for synthesis of alkyne starting material precursor.

To a solution of carboxylic acid **I** (1.0 equiv,1.51 mmol) in DCM (0.8 M), 4-iodobenzyl alcohol (1.0 equiv, 1.51 mmol), dicyclohexylcarbodiimide (DCC) (1.5 equiv, 2.27 mmol), dimethylaminopyridine (4-DMAP) (10 mol%, 0.151 mmol) was added. The reaction was stirred at room temperature until the reaction was complete. Upon completion, the mixture was concentrated under negative pressure and purified by chromatography on silica gel (eluent: EtOAc/Hex) to afford the desired ester compound **II**.

2.6 General Procedure F

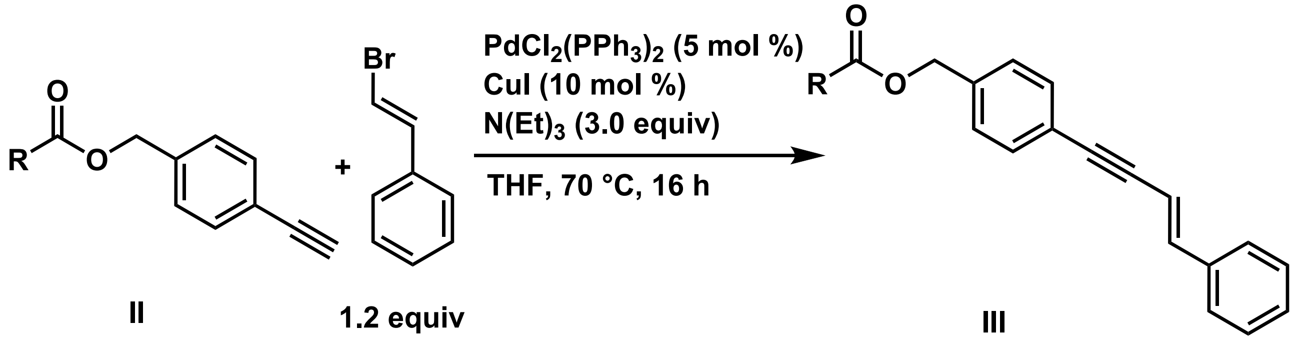

Scheme S5: Method (Sonogashira Cross-Coupling) for synthesis of enyne starting material.

According to a modified procedure from Wang *et al.*[2] To a 6-dram vial with screw on septa cap: a stir bar, **II** (1.0 equiv, 0.58 – 0.62 mmol), $PdCl_2(PPh_3)_2$ (5 mol%, 0.03 mmol) and CuI (10 mol%, 0.06 mmol) were added. The reaction flask was put under inert atmosphere (N_2) using standard *Schlenk* technique. Toluene (0.25 M), (*E*)-(2-bromovinyl)benzene (1.2 equiv, 0.70 – 0.74 mmol) and triethylamine (3.0 equiv, 1.74 – 1.86 mmol) were then added to the reaction flask. The reaction was heated to 70 °C and allowed to stir for 16 hours. The reaction mixture was reduced under negative pressure and purified via flash column chromatography to afford the corresponding enyne **III**.

2.7 General Procedure G:

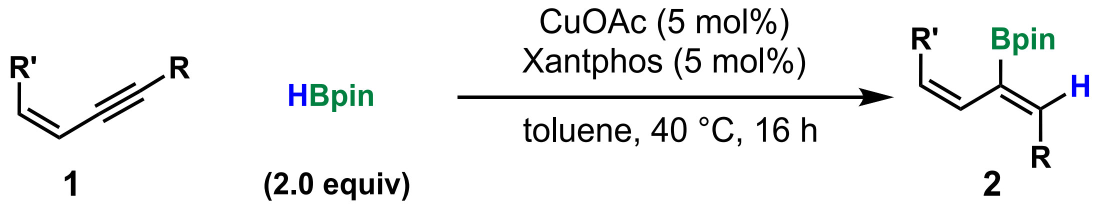

Scheme S6: Site-selective alkyne hydroboration of 1,3-enynes.

To a flame dried 2-dram vial with a septa lid: a stir bar, corresponding enyne (1.0 equiv, 0.250 mmol), copper(I)acetate (5 mol%, 0.013 mmol) and Xantphos (5 mol%, 0.013 mmol) were added. The reaction vial was put under inert atmosphere (N_2 or Ar) using standard *Schlenk* technique. Then, toluene (0.25 M) was added, and the mixture was allowed to complex at 40 °C. Once the mixture became a homogenous green solution, pinacolborane (2.0 equiv, 0.500 mmol) was added. The reaction was allowed to stir for 16 hours. Upon completion, volatile solvents were removed by rotary evaporator and the desired 2-boryl-1,3-diene was purified via flash column chromatography.

2.8 Characterization of substrates **S1a-1x**

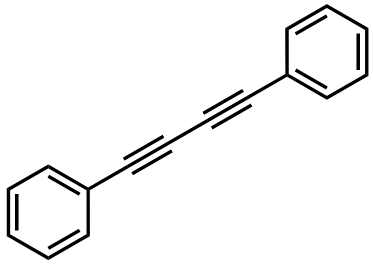

1,4-diphenylbuta-1,3-diyne (S1a): Following general procedure A, **S1a** (3.91 mmol) was isolated with 0% EtOAc:Hex in 79% yield using (9.79 mmol) ethynylbenzene. White solid. **^{1}H NMR** (400 MHz, $CDCl_3$) δ 7.54 (dd, *J* = 8.0, 1.6 Hz, 4H), 7.43 – 7.30 (m, 6H). **^{13}C NMR** (101 MHz, $CDCl_3$) δ 132.7, 129.4, 128.6, 122.0, 81.7, 74.1. Data were in accordance with literature values.[1]

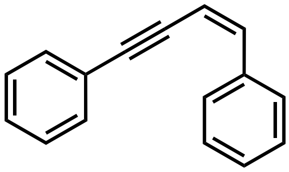

(*Z*)-but-1-en-3-yne-1,4-diyldibenze (1a): Following general procedure B, **1a** (994 µmol; >99:1, *Z*:*E*) was isolated using 0% EtOAc:Hex in 75% yield from **1a** (1.33 mmol). Yellow oil. **^{1}H NMR** (500 MHz, $CDCl_3$) δ 7.96 (d, *J* = 7.2 Hz, 2H), 7.52 (dd, *J* = 7.4, 2.3 Hz, 2H), 7.44 – 7.32 (m, 6H), 6.74 (d, *J* = 11.9 Hz, 1H), 5.96 (d, *J* = 11.9 Hz, 1H). **^{13}C NMR** (126 MHz, CDCl3) δ 138.8, 136.7, 131.6, 128.9, 128.7, 128.6, 128.5, 128.4, 123.6, 107.5, 96.0, 88.4. Data were in accordance with literature values.[1]

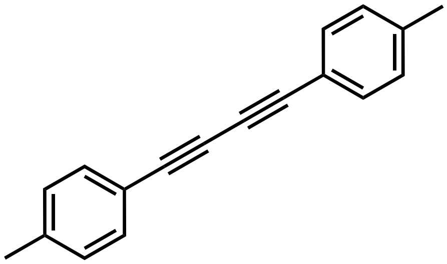

1,4-di-*p*-tolylbuta-1,3-diyne (S1b) Following general procedure A, **S1b** (1.54 mmol) was isolated with 0% EtOAc:Hex in 88% yield using (1.75 mmol) 1-ethynyl-4-methylbenzene. White solid. **^{1}H NMR** (400 MHz, $CDCl_3$) δ 7.42 (dd, *J* = 8.2, 0.7 Hz, 4H), 7.14 (dd, *J* = 8.6, 0.7 Hz, 4H), 2.37 (s, 6H). **^{13}C NMR** (101 MHz, CDCl3) δ 139.6, 132.5, 129.4, 118.9, 81.7, 73.6 21.8. Data were in accordance with literature values.[1]

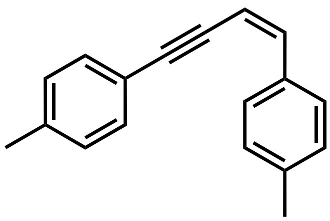

(*Z*)-4,4'-(but-1-en-3-yne-1,4-diyl)bis(methylbenzene) (1b) Following general procedure B, **1b** (395 µmol; >99:1, *Z*:*E*) was isolated using 0% EtOAc:Hex in 91% yield from **S1b** (434 µmol). Yellow oil. **^{1}H NMR** (500 MHz, $CDCl_3$) δ 7.84 (d, *J* = 8.1 Hz, 2H), 7.39 (d, *J* = 8.1 Hz, 2H), 7.19 (d, *J* = 8.0 Hz, 2H), 7.16 (d, *J* = 7.9 Hz, 2H), 6.65 (d, *J* = 11.9 Hz, 1H), 5.86 (d, *J* = 11.9

Hz, 1H), 2.37 (s, 6H). **^{13}C NMR** (126 MHz, $CDCl_3$) δ 138.6, 138.4, 134.1, 131.5, 129.3, 129.1, 128.8, 120.7, 106.6, 96.0, 88.0, 21.7, 21.6. Data were in accordance with literature values.[1]

1,4-di-*m*-tolylbuta-1,3-diyne (S1c) Following general procedure A, **S1c** (1.63 mmol) was isolated with 0% EtOAc:Hex in 93% yield using (1.75 mmol) 1-ethynyl-3-methylbenzene. White solid. **^{1}H NMR** (400 MHz, $CDCl_3$) δ 7.51 (d, J = 7.8 Hz, 2H), 7.25 (d, J = 1.0 Hz, 2H), 7.23 – 7.20 (m, 2H), 7.18 – 7.13 (m, 2H), 2.50 (s, 6H). **^{13}C NMR** (126 MHz, $CDCl_3$) δ 141.8, 133.1, 129.7, 129.3, 125.8, 121.9, 81.3, 77.6, 20.9. Data were in accordance with literature values.[1]

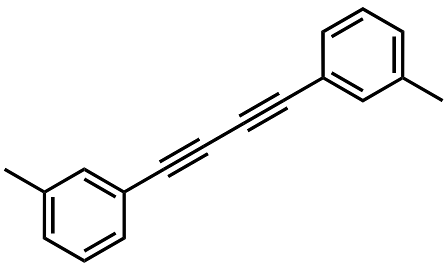

(*Z*)-3,3'-(but-1-en-3-yne-1,4-diyl)bis(methylbenzene) (1c) Following general procedure B, **1c** (360 µmol; >99:1, *Z*:*E*) was isolated using 0% EtOAc:Hex in 83% yield from **S1c** (434 µmol). Yellow oil. **^{1}H NMR** (400 MHz, $CDCl_3$) δ 7.82 (s, 1H), 7.71 (d, J = 7.7 Hz, 1H), 7.36 – 7.27 (m, 3H), 7.25 – 7.21 (m, 1H), 7.19 – 7.10 (m, 2H), 6.67 (d, J = 11.9 Hz, 1H), 5.90 (d, J = 11.9 Hz, 1H), 2.40 (s, 3H), 2.36 (s, 3H). **^{13}C NMR** (101 MHz, $CDCl_3$) δ 138.8, 138.2, 137.9, 136.7, 132.2, 129.5, 129.4, 129.4, 128.6, 128.5, 128.3, 126.2, 123.5, 107.4, 96.2, 88.3, 21.7, 21.4. Data were in accordance with literature values.[1]

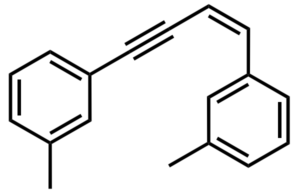

1,4-bis(4-(tert-butyl)phenyl)buta-1,3-diyne (S1d) Following general procedure A, **S1d** (1.54 mmol) was isolated with 0% EtOAc:Hex in 98% yield using (1.75 mmol) 1-(tert-butyl)-4-ethynylbenzene. White solid. **^{1}H NMR** (400 MHz, $CDCl_3$) δ 7.46 (d, J = 8.2 Hz, 4H), 7.35 (d, J = 8.2 Hz, 4H), 1.32 (s, 18H). **^{13}C NMR** (101 MHz, $CDCl_3$) δ 152.7, 132.4, 81.7, 73.6, 35.1, 31.3. Data were in accordance with literature values.[1]

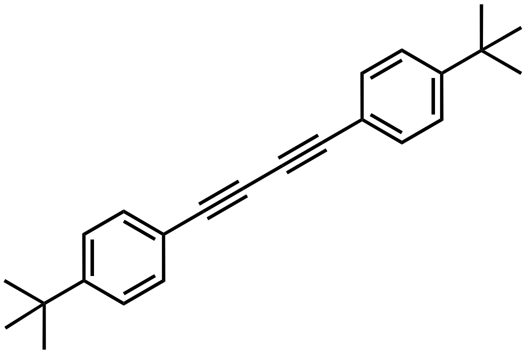

(*Z*)-4,4'-(but-1-en-3-yne-1,4-diyl)bis(tert-butylbenzene)(1d) Following general procedure B, **1d** (450 µmol; >99:1, *Z*:*E*) was isolated using 0% EtOAc:Hex in 83% yield from **S1d** (500 µmol). Yellow oil. **^{1}H NMR** (500 MHz, $CDCl_3$) δ 7.89 (d, *J* = 8.4 Hz, 2H), 7.45 (d, *J* = 8.5 Hz, 2H), 7.39 (dd, *J* = 9.6, 8.6 Hz, 4H), 6.65 (d, *J* = 11.9 Hz, 1H), 5.87 (d, *J* = 11.9 Hz, 1H), 1.34 (s, 9H), 1.33 (s, 9H). **^{13}C NMR** (126 MHz, $CDCl_3$) δ 151.8, 151.7, 138.2, 134.1, 131.3, 128.7, 125.6, 125.4, 120.7, 106.8, 96.1, 88.1, 35.0, 34.9, 31.4, 31.3. Data were in accordance with literature values.[1]

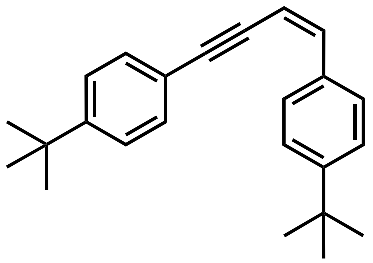

1,4-bis(4-propylphenyl)buta-1,3-diyne (S1e) Following general procedure A, **S1e** was isolated using flash column chromatography. **^{1}H NMR** (400 MHz, $CDCl_3$) δ 7.44 (d, *J* = 8.3 Hz, 4H), 7.14 (d, *J* = 8.3 Hz, 4H), 2.59 (dd, *J* = 8.5, 6.7 Hz, 4H), 1.64 (h, *J* = 7.4 Hz, 4H), 0.94 (t, *J* = 7.4 Hz, 6H). **^{13}C NMR** (101 MHz, $CDCl_3$) δ 144.3, 132.5, 128.7, 119.1, 81.7, 73.6, 38.1, 24.3, 13.8 Data were in accordance with literature values.[1]

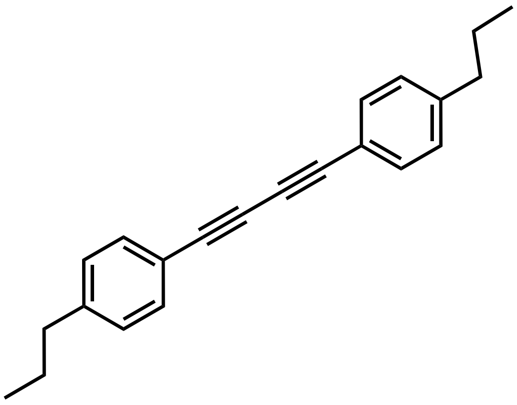

(*Z*)-4,4'-(but-1-en-3-yne-1,4-diyl)bis(propylbenzene) (1e) Following general procedure B, **1e** (290 µmol; >99:1, *Z*:*E*) was isolated using 0% EtOAc:Hex in 83% yield from **S1e** (349 µmol). Yellow oil.**^{1}H NMR** (400 MHz, $CDCl_3$) δ 7.86 (d, *J* = 8.2 Hz, 2H), 7.41 (d, *J* = 8.2 Hz, 2H), 7.20 (d, *J* = 9.0 Hz, 2H), 7.16 (d, *J* = 7.9 Hz, 2H), 6.65 (d, *J* = 11.9 Hz, 1H), 5.86 (d, *J* = 11.9 Hz, 1H), 2.60 (td, *J* = 7.6, 1.3 Hz, 4H), 1.66 (dqd, *J* = 14.8, 7.4, 2.9 Hz, 4H), 0.95 (td, *J* = 7.4, 1.1 Hz, 6H). ^{13}C NMR (101 MHz, $CDCl_3$) δ 143.5, 143.4, 138.4, 134.4, 131.5, 128.9, 128.7, 128.6, 120.9, 106.6, 96.1, 88.1, 38.1, 38.1, 24.6, 24.5, 14.0, 13.9. Data were in accordance with literature values.[1]

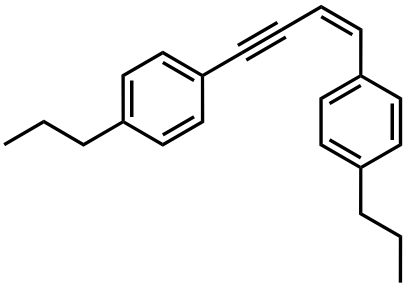

1,4-bis(4-methoxyphenyl)buta-1,3-diyne (S1f) Following general procedure A, **S1f** (3.18 mmol) was isolated with 0-5% EtOAc:Hex in 56% yield using (5.67 mmol) 1-1-ethynyl-4-methoxybenzene. White solid**.** **^{1}H NMR** (400 MHz, $CDCl_3$) δ 7.94 (d, *J* = 8.5 Hz, 4H), 7.62 (d, *J* = 8.4 Hz, 4H), 2.61 (s, 6H). **^{13}C NMR** (101 MHz, $CDCl_3$) δ 160.2, 134.0, 114.1, 113.9, 81.2, 72.9, 55.3. Data were in accordance with literature values.[1]

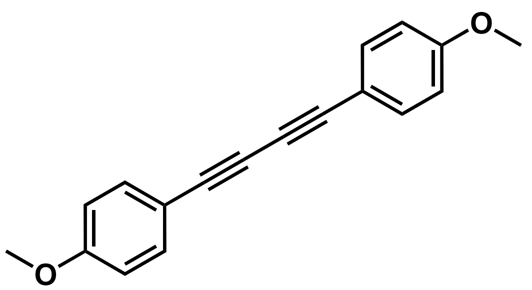

(*Z*)-4,4'-(but-1-en-3-yne-1,4-diyl)bis(methoxybenzene) (1f) Following general procedure B, **1f** (687 µmol; >99:1, *Z*:*E*) was isolated using 0-5% EtOAc:Hex in 53% yield from **S1f** (1.30 mmol). Yellow solid. **^{1}H NMR** (400 MHz, $CDCl_3$) δ 7.90 (d, *J* = 8.8 Hz, 2H), 7.43 (d, *J* = 9.0 Hz, 2H), 6.91 (d, *J* = 8.8 Hz, 2H), 6.88 (d, *J* = 8.9 Hz, 2H), 6.60 (d, *J* = 11.8 Hz, 1H), 5.79 (d, *J* = 11.9 Hz, 1H), 3.84 (s, 3H), 3.83 (s, 3H). **^{13}C NMR** (101 MHz, $CDCl_3$) δ 159.8, 159.8, 137.5, 133.0, 130.3, 129.9, 116.0, 114.3, 113.8, 105.3, 95.6, 87.6, 55.5, 55.5. Data were in accordance with literature values.[1]

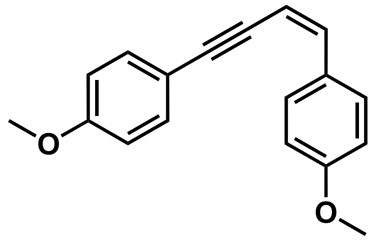

1,4-bis(3-methoxyphenyl)buta-1,3-diyne (S1g) Following general procedure A, **S1g** was isolated using flash column chromatography.**^{1}H NMR** (400 MHz, $CDCl_3$) δ 7.24 – 7.20 (m, 2H), 7.12 (dt, *J* = 7.6, 1.1 Hz, 2H), 7.04 (dd, *J* = 2.5, 1.3 Hz, 2H), 6.92 (ddd, *J* = 8.3, 2.6, 1.0 Hz, 2H), 3.80 (s, 6H). **^{13}C NMR** (101 MHz, $CDCl_3$) δ 159.4, 129.6, 125.2, 122.8, 117.2, 116.1, 81.6, 73.7, 55.4. Data were in accordance with literature values.[1]

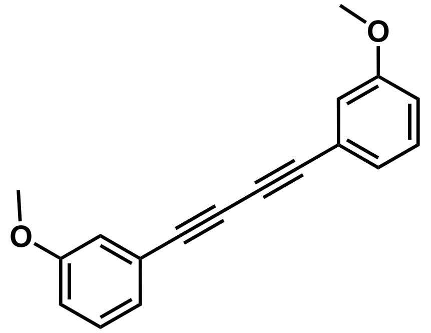

(*Z*)-3,3'-(but-1-en-3-yne-1,4-diyl)bis(methoxybenzene) (1g) Following general procedure B, **1g** (620 µmol; >99:1, *Z:E*) was isolated using 0-5% EtOAc:Hex in 62% yield from **S1g** (1.00 mmol). Yellow oil. **^{1}H NMR** (400 MHz, $CDCl_3$) δ 7.68 (s, 1H), 7.41 – 7.35 (m, 1H), 7.32 – 7.27 (m, 1H), 7.25 (s, 1H), 7.23 (s, 1H), 7.09 (dd, *J* = 7.6, 1.2 Hz, 1H), 7.03 (dt, *J* = 2.2, 1.0 Hz, 1H), 6.89 (ddd, *J* = 8.2, 7.3, 2.6 Hz, 1H), 6.69 (d, *J* = 11.9 Hz, 1H), 5.92 (d, *J* = 11.9 Hz, 1H), 3.82 (s, 3H), 3.81 (s, 3H). **^{13}C NMR** (101 MHz, $CDCl_3$) δ 159.5, 159.4, 138.7, 137.8, 129.5, 129.2, 124.4, 124.0, 121.8, 116.1, 115.1, 114.8, 113.2, 107.5, 96.1, 88.0, 55.3, 55.2. Data were in accordance with literature values.[1]

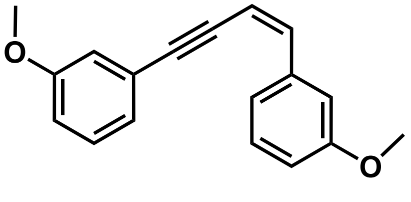

1,4-bis(4-(benzyloxy)phenyl)buta-1,3-diyne (S1h) Following general procedure A, **S1h** (823 μmol) was isolated with 0-10% EtOAc:Hex in a 47% yield from 1-(benzyloxy)-4-ethynylbenzene (1.75 mmol). White solid. **^{1}H NMR** (400 MHz, $CDCl_3$) δ 7.53 – 7.30 (m, 14H), 6.92 (dd, *J* = 9.0, 0.7 Hz, 4H), 5.08 (s, 4H). **^{13}C NMR** (101 MHz, $CDCl_3$) δ 159.4, 136.4, 134.0, 128.7, 128.1, 127.5, 115.0, 114.2, 81.2, 73.0, 70.1. Data were in accordance with literature values.[1]

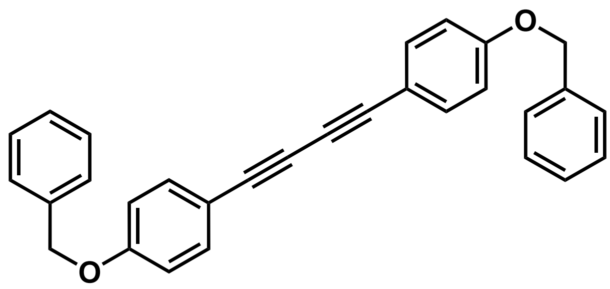

(*Z*)-4,4'-(but-1-en-3-yne-1,4-diyl)bis((benzyloxy)benzene) (1h) Following general procedure B, **1h** (311 μmol; >99:1, *Z:E*) was isolated using 0-5% EtOAc:Hex in 86% yield from **S1h** (362 μmol). Yellow solid. ^{1}H NMR (600 MHz, CDCl3) δ 7.91 – 7.86 (m, 2H), 7.45 – 7.41 (m, 5H), 7.41 – 7.37 (m, 5H), 7.35 – 7.31 (m, 2H), 6.99 – 6.96 (m, 2H), 6.96 – 6.93 (m, 2H), 6.59 (d, J = 11.9 Hz, 1H), 5.78 (d, J = 11.9 Hz, 1H), 5.09 (s, 2H), 5.08 (s, 2H). ^{13}C NMR (151 MHz, CDCl3) δ 158.94, 158.92, 137.5, 136.9, 136.7, 133.0, 130.3, 130.1, 128.8, 128.7, 128.3, 128.2, 127.62, 127.60, 116.2, 115.1, 114.7, 105.4, 95.6, 87.6, 70.2, 70.1. Data were in accordance with literature values.[1]

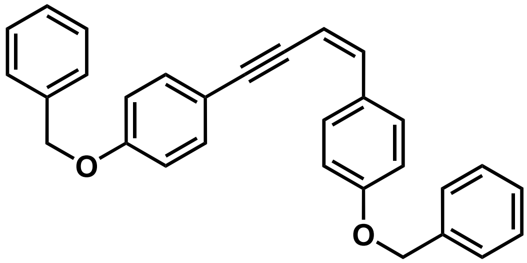

(Z)-(but-1-en-3-yne-1,4-diylbis(4,1-phenylene))bis(methylsulfane) (1i) Following general procedure B, **1i** (340 μmol; >99:1, *Z:E*) was isolated using 0-10% EtOAc:Hex in 77% yield from **S1i** (77 mg, 260 μmol). Yellow solid. ^{1}H NMR (400 MHz, $CDCl_3$) δ 7.85 (d, *J* = 8.5 Hz, 2H), 7.39 (d, *J* = 8.6 Hz, 2H), 7.23 – 7.19 (m, 3H), 6.63 (d, *J* = 11.9 Hz, 1H), 5.86 (d, *J* = 11.9 Hz, 1H), 2.51 (s, 3H), 2.50 (s, 3H). ^{13}C NMR (126 MHz, $CDCl_3$) δ 139.7, 139.3, 137.9, 133.6, 131.8, 129.2, 126.0, 126.0, 119.8, 106.8, 96.2, 88.7, 15.6, 15.5. Data were in accordance with literature values.[1]

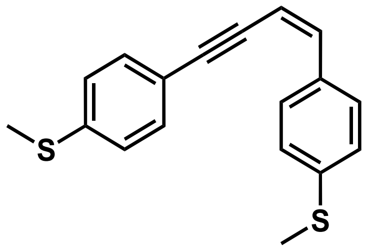

1,4-bis(4-(trifluoromethyl)phenyl)buta-1,3-diyne (S1j) Following general procedure A,

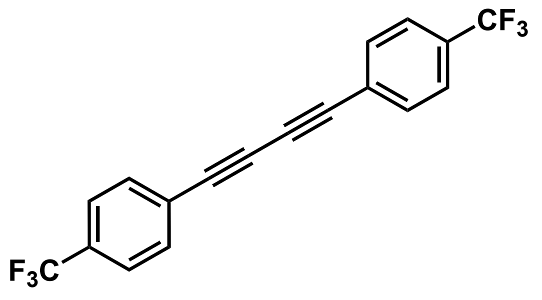

S1j (1.73 mmol) was isolated with 0-5% EtOAc:Hex in 99% yield using (1.75 mmol) 1-ethynyl-4-(trifluoromethyl)benzene. Yellow solid. **^{1}H NMR** (400 MHz, $CDCl_3$) δ 7.65 (d, *J* = 8.4 Hz, 4H), 7.61 (d, *J* = 8.9 Hz, 4H). **^{13}C NMR** (101 MHz, $CDCl_3$) δ 133.0, 131.3 (q, *J* = 32.9 Hz), 125.6 (q, *J* = 3.8 Hz), 123.8 (q, *J* = 272.5 Hz), 81.1, 75.8. Data were in accordance with literature values.[1]

(*Z*)-4,4'-(but-1-en-3-yne-1,4-diyl)bis((trifluoromethyl)benzene) (1j) Following general procedure B, **1j** (248 μmol; >99:1, *Z*:*E*) was isolated using 0-5% EtOAc:Hex in 56% yield

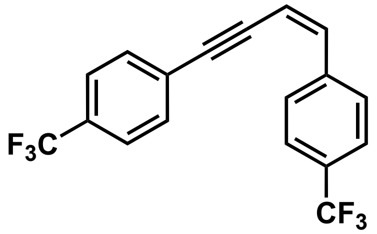

from **S1j** (443 μmol). Orange solid. **^{1}H NMR** (500 MHz, $CDCl_3$) δ 7.99 (dd, *J* = 8.1, 0.9 Hz, 2H), 7.65 (dd, *J* = 8.1, 1.2 Hz, 2H), 7.62 (d, *J* = 8.4 Hz, 2H), 7.58 (d, *J* = 8.2 Hz, 2H), 6.80 (d, *J* = 11.9 Hz, 1H), 6.06 (d, *J* = 11.9 Hz, 1H). **^{13}C NMR** (126 MHz, $CDCl_3$) δ 139.9, 138.6, 132.1, 130.8 (q, *J* = 33.5 Hz), 130.6 (q, *J* = 32.5 Hz), 129.3, 126.5, 125.9 (q, *J* = 3.9 Hz), 125.7 (q, *J* = 3.8 Hz), 125.5 (d, *J* = 272.1 Hz), 123.1 (q, *J* = 272.0 Hz), 109.9, 95.6, 90.0. Data were in accordance with literature values.[1]

1,4-bis(4-(trifluoromethoxy)phenyl)buta-1,3-diyne (S1k) Following general procedure A, **S1k** (4.06 mmol) was isolated with 0-5% EtOAc:Hex in 92% yield using (4.41 mmol) 1-

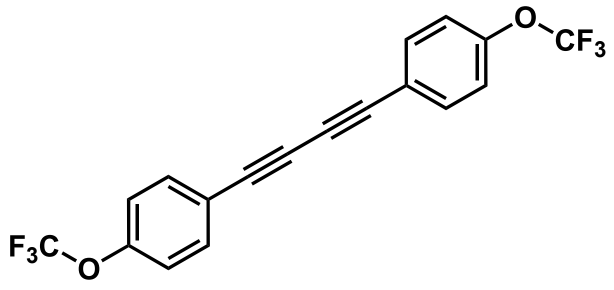

ethynyl-4-(trifluoromethoxy)benzene. Orange solid**. ^{1}H NMR** (400 MHz, $CDCl_3$) δ 7.64 (d, *J* = 8.6 Hz, 4H), 7.61 (d, *J* = 8.4 Hz, 4H). **^{13}C NMR** (101 MHz, $CDCl_3$) δ 133.0, 131.3 (q, *J* = 32.9 Hz), 125.6 (q, *J* = 3.8 Hz), 125.4, 123.8 (q, *J* = 272.5 Hz), 81.1, 75.8. Data were in accordance with literature values.[1]

(*Z*)-4,4'-(but-1-en-3-yne-1,4-diyl)bis((trifluoromethoxy)benzene) (1k) Following

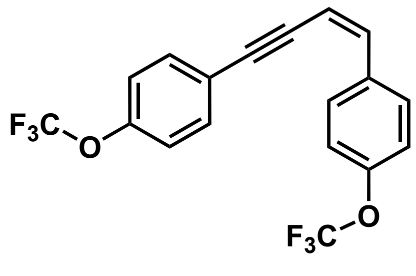

general procedure B, **1k** (297 μmol; 99:1, *Z*:*E*) was isolated using 0-5% EtOAc:Hex in 53% yield from **S1k** (443 μmol). Yellow solid.**^{1}H NMR** (500 MHz**, $CDCl_3$)** δ 7.99 (d, *J* = 8.1 Hz, 2H), 7.65 (d, *J* = 8.7 Hz, 2H), 7.62 (dd, *J* = 8.3, 0.6 Hz, 2H), 7.58 (dd, *J* = 8.7, 0.9 Hz, 2H), 6.80 (d, *J* = 11.9 Hz, 1H), 6.06 (d, *J* = 11.9 Hz, 1H). **^{13}C NMR** (126 MHz, $CDCl_3$) δ 139.9, 138.6, 132.1, 130.9 (q, *J* =

10.8 Hz), 130.6 (q, J = 10.5 Hz), 129.3, 126.9 (q, J = 84.4 Hz), 125.9 (q, J = 3.9 Hz), 125.7 (q, J = 3.8 Hz), 125.5 (q, J = 272.0 Hz), 123.1 (q, J = 272.1 Hz), 109.9, 95.6, 90.0. Data were in accordance with literature values.[1]

1,4-bis(4-fluorophenyl)buta-1,3-diyne (S1l) Following general procedure A, **S1l** was isolated with 0-5% EtOAc:Hex from 1-ethynyl-4-(trifluoromethoxy)benzene. Yellow solid. **^{1}H NMR** (400 MHz, $CDCl_3$) δ 7.50 (dd, J = 9.0, 5.4 Hz, 4H), 7.03 (dd, J = 8.7, 5.7 Hz, 3H). **^{13}C NMR** (101 MHz, $CDCl_3$) δ 163.2 (d, J = 251.4 Hz), 134.6 (d, J = 8.7 Hz), 117.9, 116.0 (d, J = 22.2 Hz), 80.5, 73.8. Data were in accordance with literature values.[1]

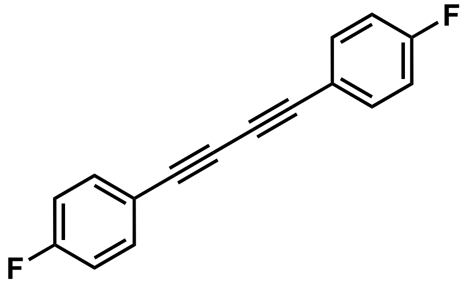

(Z)-4,4'-(but-1-en-3-yne-1,4-diyl)bis(fluorobenzene) (1l) Following general procedure B, **1l** (670 µmol; 98:2, Z:E) was isolated using 0-5% EtOAc:Hex in 67% yield from **S1l** (1.00 µmol). Yellow oil.**^{1}H NMR** (400 MHz, $CDCl_3$) δ 7.89 (dd, J = 9.1, 5.5 Hz, 2H), 7.45 (dd, J = 8.9, 5.3 Hz, 2H), 7.12 – 7.01 (m, 4H), 6.66 (d, J = 11.9 Hz, 1H), 5.88 (d, J = 11.9 Hz, 1H). **^{13}C NMR** (101 MHz, $CDCl_3$) δ 162.6 (d, J = 250.2 Hz), 161.3 (d, J = 248.9 Hz), 137.4, 133.3 (d, J = 8.2 Hz), 132.8 (d, J = 3.4 Hz), 130.5 (d, J = 8.1 Hz), 119.4 (d, J = 3.8 Hz), 115.8 (d, J = 22.1 Hz), 115.3 (d, J = 21.7 Hz), 106.8 (d, J = 2.3 Hz), 94.7, 87.6. Data were in accordance with literature values.[1]

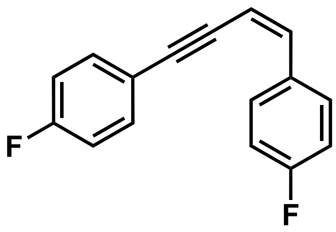

1,4-bis(3-fluorophenyl)buta-1,3-diyne (S1m) Following general procedure A, **S1m** was isolated using 0% EtOAc:Hex. Yellow solid. **^{1}H NMR** (500 MHz, $CDCl_3$) δ 7.34 – 7.30 (m, 4H), 7.25 – 7.18 (m, 2H), 7.13 – 7.07 (m, 2H). **^{13}C NMR** (126 MHz, $CDCl_3$) δ 162.4 (d, J = 247.5 Hz), 130.3 (d, J = 8.6 Hz), 128.6 (d, J = 3.2 Hz), 123.5 (d, J = 9.5 Hz), 119.4 (d, J = 23.1 Hz), 117.1 (d, J = 21.2 Hz), 80.8 (d, J = 3.4 Hz), 74.5. Data were in accordance with literature values.[1]

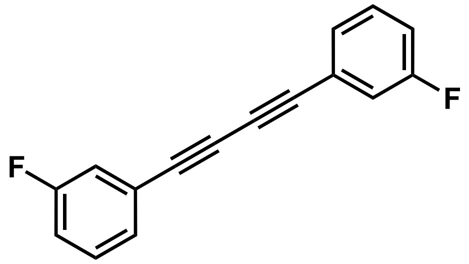

(*Z*)-3,3'-(but-1-en-3-yne-1,4-diyl)bis(fluorobenzene) (1m) Following general procedure B, **1m** (630 µmol; 96:4, *Z:E*) was isolated using 0-5% EtOAc:Hex in 63% yield from **S1m** (1.00 mmol). Yellow oil. **^{1}H NMR** (400 MHz, $CDCl_3$) δ 7.77 (ddd, *J* = 10.5, 2.7, 1.8 Hz, 1H), 7.52 (ddt, *J* = 7.7, 1.6, 0.7 Hz, 1H), 7.37 – 7.29 (m, 2H), 7.27 (t, *J* = 1.3 Hz, 1H), 7.17 (ddd, *J* = 9.4, 2.6, 1.4 Hz, 1H), 7.07 – 6.98 (m, 2H), 6.69 (d, *J* = 12.0 Hz, 1H), 5.94 (d, *J* = 12.0 Hz, 1H). **^{13}C NMR** (101 MHz, $CDCl_3$) δ 162.8 (d, J = 245 Hz), 162.6 (d, J = 247 Hz), 138.6 (d, J = 8 Hz), 138.1 (d, J = 3 Hz), 130.2 (d, J = 9 Hz), 129.9 (d, J = 8 Hz), 127.5 (d, J = 3 Hz), 125.1 (d, J = 10 Hz), 125.0 (d, J = Hz), 118.3 (d, J = 23 Hz), 116.1 (d, J = 21 Hz), 115.7 (d, J = 21 Hz), 115.1 (d, J = 23 Hz), 108.5, 95.5 (d, J = 4 Hz), 88.7. Data were in accordance with literature values.[1]

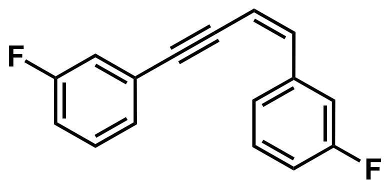

1,4-bis(4-chlorophenyl)buta-1,3-diyne (S1n) Following general procedure A, **S1n** (1.16 mmol) was isolated with 0% EtOAc:Hex in 66% yield using (1.75 mmol) 1-chloro-4-ethynylbenzene. Yellow solid. **^{1}H NMR** (400 MHz, $CDCl_3$) δ 7.45 (d, *J* = 8.7 Hz, 4H), 7.32 (d, *J* = 8.8 Hz, 4H). ^{13}C NMR (101 MHz, $CDCl_3$) δ 135.6, 133.7, 128.9, 120.1, 80.8, 74.6. Data were in accordance with literature values.[1]

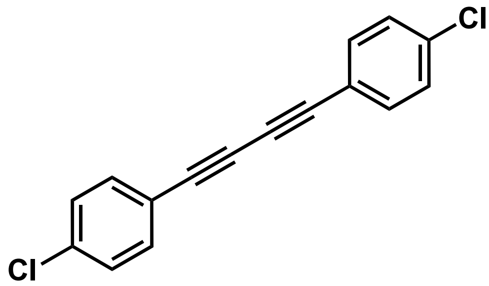

(*Z*)-4,4'-(but-1-en-3-yne-1,4-diyl)bis(chlorobenzene) (1n) Following general procedure B, **1n** (325 µmol; >99:1, *Z:E*) was isolated using 0-5% EtOAc:Hex in 65% yield from **S1n** (500 µmol). Yellow oil. **^{1}H NMR** (400 MHz, $CDCl_3$) δ 7.82 (dd, *J* = 8.5, 1.9 Hz, 2H), 7.40 (dd, *J* = 8.7, 2.1 Hz, 2H), 7.37 – 7.31 (m, 4H), 6.67 (d, *J* = 11.9 Hz, 1H), 5.92 (d, *J* = 11.9 Hz, 1H). **^{13}C NMR** (101 MHz, $CDCl_3$) δ 137.7, 134.9, 134.6, 134.2, 133.7, 132.6, 129.9, 129.04, 129.0, 128.5, 121.7, 107.8, 95.2, 88.8. Data were in accordance with literature values.[1]

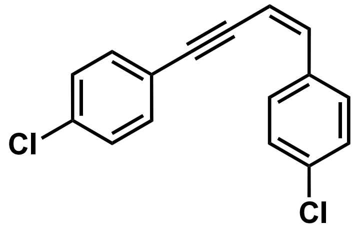

1,4-bis(3-chlorophenyl)buta-1,3-diyne (S1o) Following general procedure A, **S1o** (1.70 mmol) was isolated with 0% EtOAc:Hex in 97% yield using (1.75 mmol) 1-chloro-3-ethynylbenzene. Yellow solid. **^{1}H NMR** (400 MHz, $CDCl_3$) δ 7.51 (ddd, *J* = 2.2, 1.6, 0.5 Hz, 2H), 7.41 (ddd, *J* = 7.6, 1.9, 0.9 Hz, 2H), 7.38 – 7.35 (m, 2H), 7.29 (dd, *J* = 7.6, 0.5 Hz, 2H). **^{13}C NMR** (101 MHz, $CDCl_3$) δ 134.5, 132.4, 130.8, 129.9, 129.9, 123.4, 80.7, 74.8. Data were in accordance with literature values.[1]

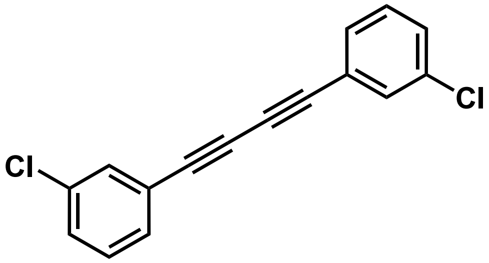

(*Z*)-3,3'-(but-1-en-3-yne-1,4-diyl)bis(chlorobenzene) (1o) Following general procedure B, **1p** (325 μmol; 98:2, *Z*:*E*) was isolated using 0-5% EtOAc:Hex in 65% yield from **S1p** (500 μmol). Yellow oil. **^{1}H NMR** (500 MHz, $CDCl_3$) δ 8.11 (d, *J* = 2.0 Hz, 1H), 7.63 (dt, *J* = 7.0, 1.8 Hz, 1H), 7.50 (t, *J* = 1.8 Hz, 1H), 7.39 (dt, *J* = 7.3, 1.5 Hz, 1H), 7.35 – 7.27 (m, 4H), 6.68 (d, *J* = 11.8 Hz, 1H), 5.95 (d, *J* = 11.9 Hz, 1H). **^{13}C NMR** (126 MHz, $CDCl_3$) δ 138.2, 138.0, 134.5, 134.4, 131.5, 129.9, 129.8, 129.7, 129.0, 128.7, 128.5, 127.3, 124.9, 108.7, 95.4, 88.9. Data were in accordance with literature values.[1]

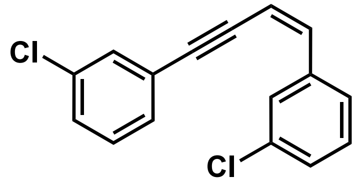

1,4-bis(2-chlorophenyl)buta-1,3-diyne (S1p) Following general procedure A, **S1o** (1.70 mmol) was isolated with 0-5% EtOAc:Hex in 97% yield using (1.75 mmol) 1-chloro-2-ethynylbenzene. Yellow solid. **^{1}H NMR** (400 MHz, $CDCl_3$) δ 7.57 (ddd, *J* = 7.6, 1.8, 0.5 Hz, 2H), 7.42 (ddd, *J* = 8.1, 1.3, 0.5 Hz, 2H), 7.31 (ddd, *J* = 8.0, 7.4, 1.8 Hz, 2H), 7.23 (dd, *J* = 7.5, 1.3 Hz, 2H). **^{13}C NMR** (101 MHz, $CDCl_3$) δ 137.1, 134.5, 130.4, 129.6, 126.7, 122.0, 79.6, 78.5. Data were in accordance with literature values.[1]

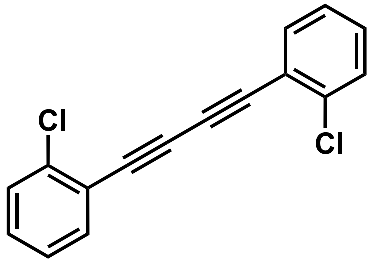

(*Z*)-2,2'-(but-1-en-3-yne-1,4-diyl)bis(chlorobenzene) (1p) Following general procedure B, **1o** (375 μmol; >99:1, *Z*:*E*) was isolated using 0-7% EtOAc:Hex in 75% yield from **S1o** (500 μmol). Yellow oil. **^{1}H NMR** (400 MHz, $CDCl_3$) δ 8.59 (ddd, *J* = 7.7, 1.7, 0.4 Hz, 1H), 7.46 (ddd, *J* = 7.2, 1.8, 0.5 Hz, 1H), 7.43 – 7.38 (m, 2H), 7.32 – 7.26 (m, 2H), 7.25 – 7.19 (m, 2H), 7.15 (d, *J* = 12.0 Hz, 1H), 6.11 (d, *J* = 12.0 Hz, 1H). **^{13}C NMR** (101 MHz, $CDCl_3$) δ 136.0, 135.5, 134.1, 133.9, 133.7, 129.9, 129.8, 129.7, 129.6, 129.5, 126.7, 126.6, 123.3, 109.3, 93.0, 92.3. Data were in accordance with literature values.[1]

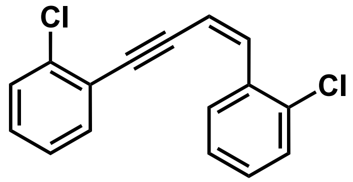

1,4-di(thiophen-2-yl)buta-1,3-diyne (S1q) Following general procedure A, **S1q** (1.49 mmol) was isolated with 0% EtOAc:Hex in 85% yield using (1.75 mmol) 2-ethynylthiophene. Off-white solid**. ^{1}H NMR** (400 MHz, $CDCl_3$) δ 7.35 (dd, *J* = 3.6, 1.2 Hz, 2H), 7.33 (dd, *J* = 5.2, 1.2 Hz, 2H), 7.00 (dd, *J* = 5.2, 3.7 Hz, 2H). **^{13}C NMR** (101 MHz, $CDCl_3$) δ 134.6, 129.1, 127.4, 122.1, 77.9, 76.8. Data were in accordance with literature values.[1]

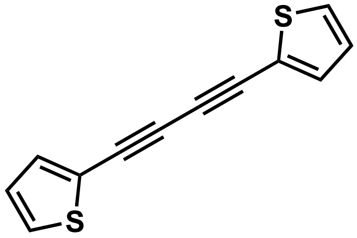

(*Z*)-2,2'-(but-1-en-3-yne-1,4-diyl)dithiophene (1q) Following general procedure B, **1q** (719 µmol; 98:2, *Z:E*) was isolated using 0-5% EtOAc:Hex in 77% yield from **S1q** (933 µmol). Yellow oil. **^{1}H NMR** (400 MHz, $CDCl_3$) δ 7.37 (ddd, *J* = 5.9, 1.3, 0.6 Hz, 1H), 7.35 – 7.31 (m, 2H), 7.28 (ddd, *J* = 3.6, 1.2, 0.6 Hz, 1H), 7.04 (ddd, *J* = 4.9, 3.7, 1.2 Hz, 2H), 6.96 (d, *J* = 11.3 Hz, 1H), 5.76 (d, *J* = 11.3 Hz, 1H). **^{13}C NMR** (101 MHz, $CDCl_3$) δ 141.0, 132.4, 131.9, 129.9, 128.0, 127.4, 127.4, 126.6, 123.7, 104.3, 92.6, 92.3. Data were in accordance with literature values.[1]

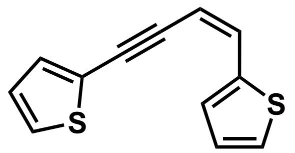

(*E*)-1-methoxy-4-(4-phenylbut-3-en-1-yn-1-yl)benzene (1s) Following general procedure C: **1s** (853.2 µmol; 15:85, *Z:E*) was isolated using 0-10% EtOAc:Hex in 78% yield from 1-ethynyl-4-methoxybenzene (1.09 mmol). White solid. **^{1}H NMR** (400 MHz, $CDCl_3$) δ 7.46 – 7.27 (m, 7H), 7.01 (d, *J* = 16.2 Hz, 1H), 6.87 (d, *J* = 8.8 Hz, 2H), 6.38 (d, *J* = 16.2 Hz, 1H), 3.83 (s, 3H). **^{13}C NMR** (101 MHz, $CDCl_3$) δ 159.7, 140.6, 136.6, 133.1, 128.9, 128.6, 126.4, 115.7, 114.2, 108.5, 92.0, 87.8, 55.5. Data were in accordance with literature values.[3]

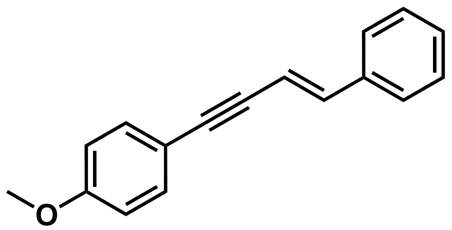

(*E*)-N,N-dimethyl-4-(4-phenylbut-3-en-1-yn-1-yl)aniline (1t) Following general procedure C: **1t** (1.088 mmol; <1:99, *Z:E*) was isolated using 0-10% EtOAc:Hex in 99% yield from 4-ethynyl-N,N-dimethylaniline (1.09 mmol). Yellow solid. **^{1}H NMR** (400 MHz, $CDCl_3$) δ 7.42 – 7.39 (m, 2H), 7.37 – 7.30 (m, 5H), 6.96 (d, *J* = 16.2 Hz, 1H), 6.65 (d, *J* = 9.1 Hz, 2H), 6.40 (d, *J* = 16.2 Hz, 1H), 2.99 (s, 6H). **^{13}C NMR** (101 MHz, $CDCl_3$) δ 152.2, 139.5, 136.9, 132.8, 128.8, 128.3, 126.3, 112.0, 110.3, 109.0, 93.9, 87.2, 40.4 Data were in accordance with literature values.[3]

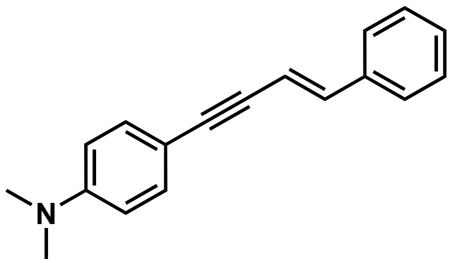

(*E*)-trimethyl(4-phenylbut-3-en-1-yn-1-yl)silane (1u) Following general procedure C: **1u** (1.08 mmol; 6:94, *Z:E*) was isolated using 0% EtOAc:Hex in 99% yield from 4- ethynyltrimethylsilane (1.09 mmol). Yellow oil. **^{1}H NMR** (600 MHz, $CDCl_3$) δ 7.37 (dd, *J* = 8.8, 2.3 Hz, 2H), 7.32 (td, *J* = 8.3, 1.9 Hz, 2H), 7.30 – 7.27 (m, 1H), 7.01 (d, *J* = 16.3 Hz, 1H), 6.18 (d, *J* = 16.3 Hz, 1H), 0.23 (s, 9H). **^{13}C NMR** (101 MHz, $CDCl_3$) δ 142.5, 140.0, Data were in accordance with literature values.[4]

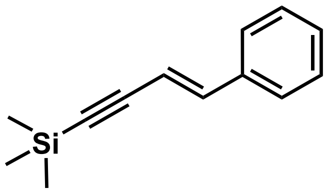

(*E*)-pent-1-en-3-yn-1-ylbenzene (1v) Following general procedure D: **1v** (590 μmol; 10:90, *Z:E*) was isolated using 0% EtOAc:Hex in 54% yield from prop-1-yn-1-ylmagnesium bromide (1.09 mmol). Colorless oil. **^{1}H NMR** (400 MHz, $CDCl_3$) δ 7.38 – 7. 26 (m, 5H), 6.14 (dq, *J* = 16.2, 2.4 Hz, 1H), 2.02 (dd, *J* = 2.4, 0.7 Hz, 3H). **^{13}C NMR** (101 MHz, $CDCl_3$) δ 140.2, 136.7, 128.8, 128.4, 126.2, 109.0, 88.5, 79.1, 4.7. Data were in accordance with literature values.[5]

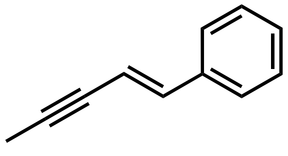

4-ethynylbenzyl (*R*)-2-(6-methoxynaphthalen-2-yl)propanoate (S1w) Following general procedure E, compound **S1w** (1.32 mmol) was isolated using (*R*)-2-(6-methoxynaphthalen-2-yl)propanoic acid (1.51 mmol) by flash column chromatography, 5-20% EtOAc:Hex in an 87% yield. White solid. **^{1}H NMR** (400 MHz, $CDCl_3$) δ 7.71 – 7.66 (m, 2H), 7.64 – 7.63 (m, 1H), 7.39 (m, 3H), 7.18 (d, *J* = 0.6 Hz, 1H), 7.16 – 7.11 (m, 3H), 5.10 (d, *J* = 12.8 Hz 1H), 5.08 (d, *J* = 12.8 Hz, 1H), 3.92 (s, 3H), 3.89 (q, *J* = 7.1 Hz, 1H), 3.06 (s, 1H), 1.59 (d, *J* = 7.2 Hz, 3H). **^{13}C NMR** (101 MHz, $CDCl_3$) δ 174.5, 157.8, 136.9, 135.5, 133.9, 132.3, 129.4, 129.1, 127.8, 127.3, 126.4, 126.1, 122.0, 119.2, 105.7, 83.4, 77.6, 66.0, 55.5, 45.6, 18.6. HRMS calcd for $C_{23}H_{21}O_3$ $[M+H]^+$ 345.1491 m/z, found 345.1490 m/z. Δ -0.2897. Data were in accordance with literature values.[6]

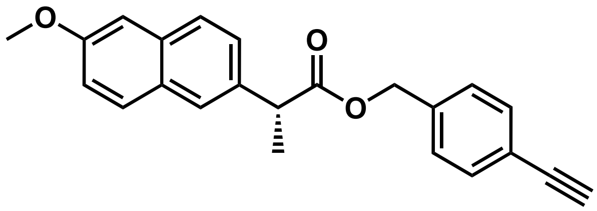

4-ethynylbenzyl 5-(2,4-dimethylphenoxy)-2,2-dimethylpentanoate (S1x) Following general procedure E, compound **S1x** (1.41 mmol) was isolated using 5-(2,5-dimethylphenoxy)-2,2-dimethylpentanoic acid (1.51 mmol) by flash column chromatography, 0-25% EtOAc:Hex in an 93% yield. White solid.**^{1}H NMR** (400 MHz, $CDCl_3$) δ 7.46 (d, *J* = 8.5 Hz, 2H), 7.28 (dd, *J* = 7.9, 0.7 Hz, 2H), 6.97 (d, *J* = 0.9 Hz, 1H), 6.64 (d, *J* = 7.5 Hz, 1H), 6.58 (s, 1H), 5.08 (s, 2H), 3.87 (t, *J* = 5.7 Hz, 2H), 3.07 (s, 1H), 2.29 (s, 3H), 2.14 (s, 3H), 1.73 – 1.70 (m, 4H), 1.23 (s, 6H). **^{13}C NMR** (101 MHz, $CDCl_3$) δ 177.6, 157.0, 137.1, 136.5, 132.4, 130.4, 127.8, 123.7, 121.9, 120.9, 120.8, 112.0, 83.4, 77.6, 67.9, 65.7, 42.3, 37.2, 24.8, 21.5, 15.8. HRMS calcd for $C_{24}H_{29}O_3^+$ [M+H]$^+$ 365.2117 m/z, found 365.2130 m/z. Δ 3.5596.

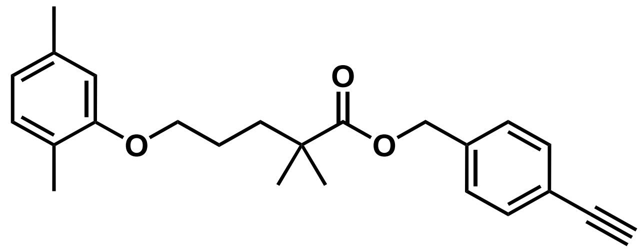

(*E*)-4-(4-phenylbut-3-en-1-yn-1-yl)benzyl (*R*)-2-(6-methoxynaphthalen-2-yl)propanoate (1w) Following general procedure F, compound **1w** (360 μmol) was isolated using **S1w** (581 μmol) by flash column chromatography, 0-20% EtOAc:Hex in a 62% yield. Colorless oil.**^{1}H NMR** (400 MHz, $CDCl_3$) δ 7.69 (dd, *J* = 8.6, 5.5 Hz, 2H), 7.65 – 7.64 (m, 1H), 7.44 – 7.39 (m, 3H), 7.39 – 7.34 (m, 4H), 7.33 – 7.29 (m, 1H), 7.19 (d, *J* = 0.6 Hz, 1H), 7.18 – 7.10 (m, 3H), 7.04 (d, *J* = 16.3 Hz, 1H), 6.37 (d, *J* = 16.2 Hz, 1H), 5.13 (d, *J* = 12.8 Hz, 1H), 5.08 (d, *J* = 12.8 Hz, 1H), 3.92 (s, 3H), 3.90 (q, *J* = 7.1 Hz, 1H), 1.60 (d, *J* = 7.2 Hz, 3H). **^{13}C NMR** (101 MHz, $CDCl_3$) δ 174.4, 147.0, 141.4, 136.0, 133.7, 131.5, 129.3, 128.9, 128.7, 128.7, 128.3, 127.8, 127.2, 126.3, 126.2, 126.0, 123.9, 123.1, 119.0, 108.0, 105.6, 91.3, 77.2, 66.0, 55.3, 45.4, 18.5. HRMS calcd for $C_{31}H_{27}O_3^+$ [M+H]$^+$ 447.1960 m/z, found 447.1974 m/z. Δ 3.1306.

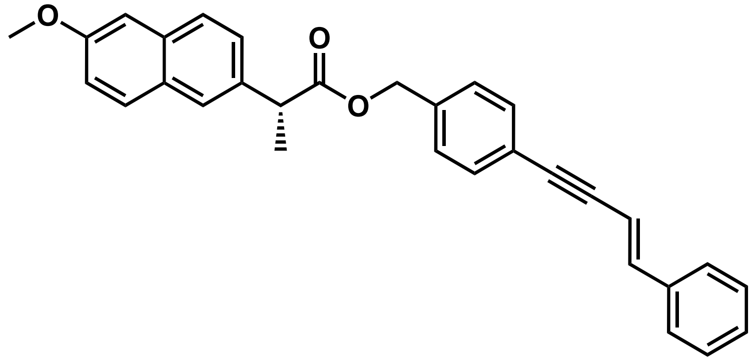

(*E*)-4-(4-phenylbut-3-en-1-yn-1-yl)benzyl-5-(2,4-dimethylphenoxy)-2,2-dimethylpentanoate (1x) Following general procedure F, compound **1x** (222 µmol) was isolated using **S1x** (617 µmol) by flash column chromatography, 0-20% EtOAc:Hex in a

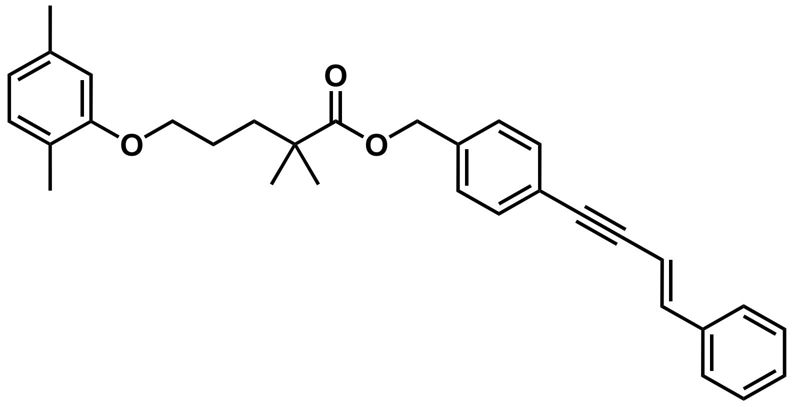

36% yield. Colorless oil. **^{1}H NMR** (400 MHz, $CDCl_3$) δ 7.47 – 7.41 (m, 4H), 7.38 – 7.33 (m, 2H), 7.32 – 7.28 (m, 3H), 7.05 (d, *J* = 16.2 Hz, 1H), 7.00 (dd, *J* = 7.4, 0.9 Hz, 1H), 6.68 – 6.64 (m, 1H), 6.60 (d, *J* = 16.2 Hz, 1H), 6.38 (d, *J* = 16.2 Hz, 1H), 5.11 (s, 2H), 3.89 (t, *J* = 5.6 Hz, 2H), 2.31 (s, 3H), 2.16 (s, 3H), 1.75 – 1.72 (m, 4H), 1.25 (s, 6H). **^{13}C NMR** (101 MHz, $CDCl_3$) δ 177.7, 157.1, 141.6, 136.6, 136.5, 136.5, 131.8, 130.4, 128.8, 128.0, 126.5, 123.8, 123.3, 120.9, 112.1, 108.2, 91.5, 89.4, 68.0, 65.9, 43.7, 42.3, 37.3, 25.3, 24.7, 21.5, 15.9. HRMS calcd for $C_{32}H_{35}O_3^+$ $[M+H]^+$ 467.2586 m/z, found 467.2589 m/z. Δ 0.6420.

3. Synthesis of Substrate **1r**

3.1 Synthesis of 2-(3-(4-iodophenoxy)propyl)isoindoline-1,3-dione (**S1r**)

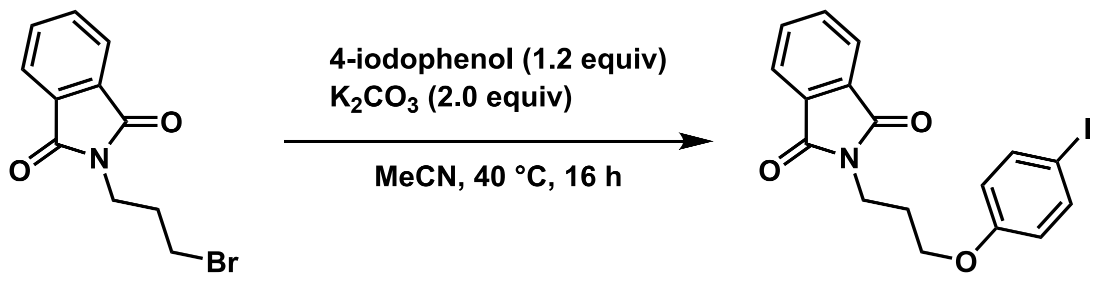

S1r

Scheme S7: Synthesis of   via SN_2 conditions.

Procedure: To a 6-dram vial: 2-(3-bromopropyl)isoindoline-1,3-dione (2.27 mmol), 4-iodophenol (2.73 mmol), potassium carbonate (4.55 mmol) and MeCN (5 mL) were added. The vial was heated to 40 °C for 16 hours. Water (5 mL) and EtOAc (5 mL) were added. Two additional aliquots of EtOAc were added and extracted, then washed with brine. The organic phase was reduced under negative pressure and purified via column chromatography to afford 2-(3-(4-iodophenoxy)propyl)isoindoline-1,3-dione **21a** (923 mg, 2.27 mmol, 87%) as a white amorphous solid. **^{1}H NMR** (400 MHz, $CDCl_3$) δ 7.84 (dd, *J* = 5.5, 3.0 Hz, 2H), 7.72 (dd, *J* = 5.5, 3.0 Hz, 2H), 7.50 (d, *J* = 9.0 Hz, 2H), 6.57 (d, *J* = 9.1 Hz, 2H), 3.98 (t, *J* = 6.0 Hz, 2H), 3.90 (t, *J* = 6.8 Hz, 2H), 2.22 – 2.12 (m, 2H). **^{13}C NMR** (101 MHz, $CDCl_3$) δ 168.3, 158.6, 138.1, 134.0, 132.1, 123.3, 116.8, 82.9, 65.8, 35.4, 28.2. HRMS calcd for $C_{17}H_{14}INNaO_3^+$ $[M+Na]^+$ 429.9911 m/z, found 429.9891 m/z. Δ -4.6513.

3.2 Synthesis of 2-(3-(4-((trimethylsilyl)ethynyl)phenoxy)propyl)isoindoline-1,3-dione (**S2r**)

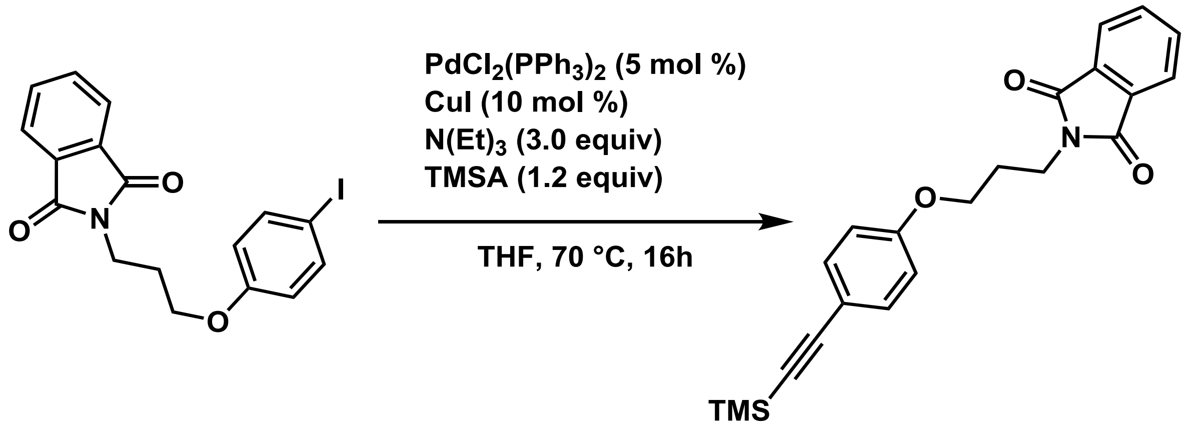

S1r **S2r**

Scheme S8: Synthesis of **S2r** via Sonogashira cross-coupling conditions.

Procedure: To a 6-dram vial with screw on septa cap: a stir bar, 2-(3-(4-iodophenoxy)propyl)isoindoline-1,3-dione **S1r** (1.11 mmol), $PdCl_2(PPh_3)_2$ (55.3 µmol) and CuI (111 µmol) were added. The reaction flask was put under inert atmosphere (N_2) using standard *Schlenk* technique. THF (5 mL), TMSA (1.33 mmol) and triethylamine (3.32 mmol) were then added to the reaction flask. The reaction was heated to 70 °C and allowed to stir for 16 hours. Water (5 mL) and EtOAc (5 mL) were added. Two additional aliquots of EtOAc were added and extracted, then washed with brine. The organic phase was reduced under negative pressure and purified via column chromatography to afford 2-(3-(4-((trimethylsilyl)ethynyl)phenoxy)propyl)isoindoline-1,3-dione **S2r** (298.1 mg, 790.4 µmol, 71%) as a white amorphous. **^{1}H NMR** (400 MHz, $CDCl_3$) δ 7.84 (dd, *J* = 5.5, 2.9 Hz, 2H), 7.71 (dd, *J* = 5.5, 3.1 Hz, 2H), 7.34 (d, *J* = 8.9 Hz, 2H), 6.70 (d, *J* = 8.9 Hz, 2H), 4.02 (t, *J* = 6.0 Hz, 2H), 3.90 (t, *J* = 6.8 Hz, 2H), 2.22 – 2.14 (m, 2H), 0.23 (s, 9H). **^{13}C NMR** (101 MHz, $CDCl_3$) δ 168.5, 159.0, 138.3, 134.1, 133.6, 123.4, 117.0, 114.4, 105.4, 92.6, 65.8, 35.6, 28.4, 0.2. HRMS calcd for $C_{22}H_{24}NO_3Si^+$ $[M+H]^+$ 378.1525 m/z, found 378.1529 m/z. Δ 1.0578.

3.3 Synthesis of 2-(3-(4-ethynylphenoxy)propyl)isoindoline-1,3-dione (**S3r**)

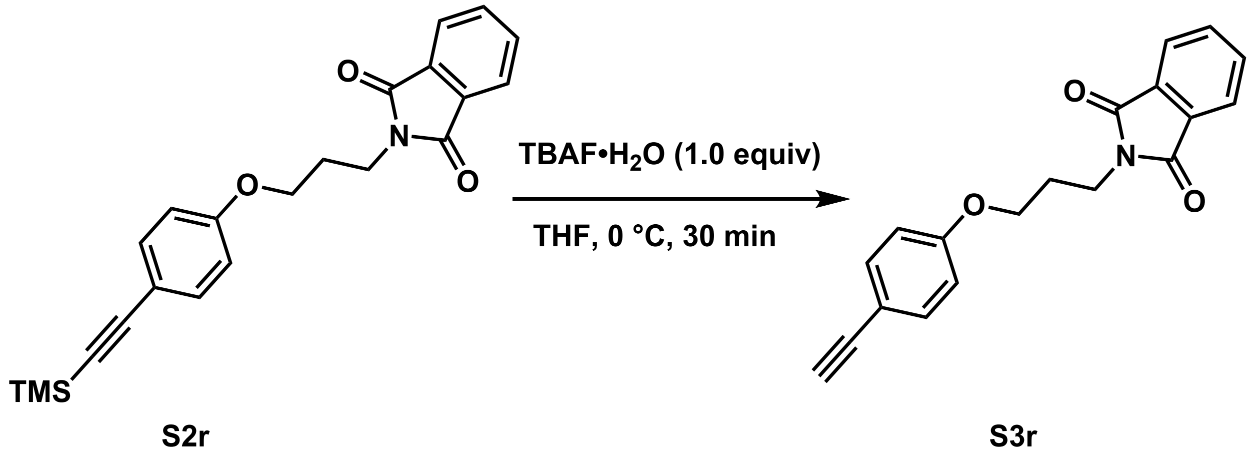

Scheme S9: Synthesis of **S3r** via TMS deprotection.

Procedure: To a 6 dram vial, 2-(3-(4-((trimethylsilyl)ethynyl)phenoxy)propyl)isoindoline-1,3-dione **S2r** (609 µmol) was dissolved in THF (6 mL). Then, TBAF trihydrate (731 µmol) was added slowly at 0 °C. The reaction was stirred at 0 °C until TLC analysis showed the complete consumption of **S2r** (about 0.5 h). The solution was quenched with NH_4Cl (aq) (10 mL). Then Water (5 mL) and EtOAc (5 mL) were added. Two additional aliquots of EtOAc were added and extracted, then washed with brine. The organic phase was reduced under negative pressure and purified via column chromatography to afford 2-(3-(4-ethynylphenoxy)propyl)isoindoline-1,3-dione **S3r** (167.23 mg, 548.1 µmol, 90%) as a white amorphous solid. **^{1}H NMR** (400 MHz, $CDCl_3$) δ 7.84 (dd, *J* = 5.5, 3.1 Hz, 2H), 7.72 (dd, *J* = 5.5, 3.0 Hz, 2H), 7.37 (d, *J* = 8.9 Hz, 2H), 6.73 (d, *J* = 8.9 Hz, 2H), 4.03 (t, *J* = 6.1 Hz, 2H), 3.91 (t, *J* = 6.8 Hz, 2H), 2.98 (s, 1H), 2.22 – 2.15 (m, 2H). **^{13}C NMR** (101 MHz, CDCl3) δ 168.5, 159.2, 134.1, 133.7, 132.3, 123.4, 114.6, 114.4, 83.8, 75.9, 65.9, 35.6, 28.4. HRMS calcd for $C_{19}H_{15}NNaO_3^+$ $[M+Na]^+$ 328.0944 m/z, found 328.0959 m/z. Δ 4.5719.

3.4 Synthesis of (*E*)-2-(3-(4-(4-phenylbut-3-en-1-yn-1-yl)phenoxy)propyl)isoindoline-1,3-dione (**1r**)

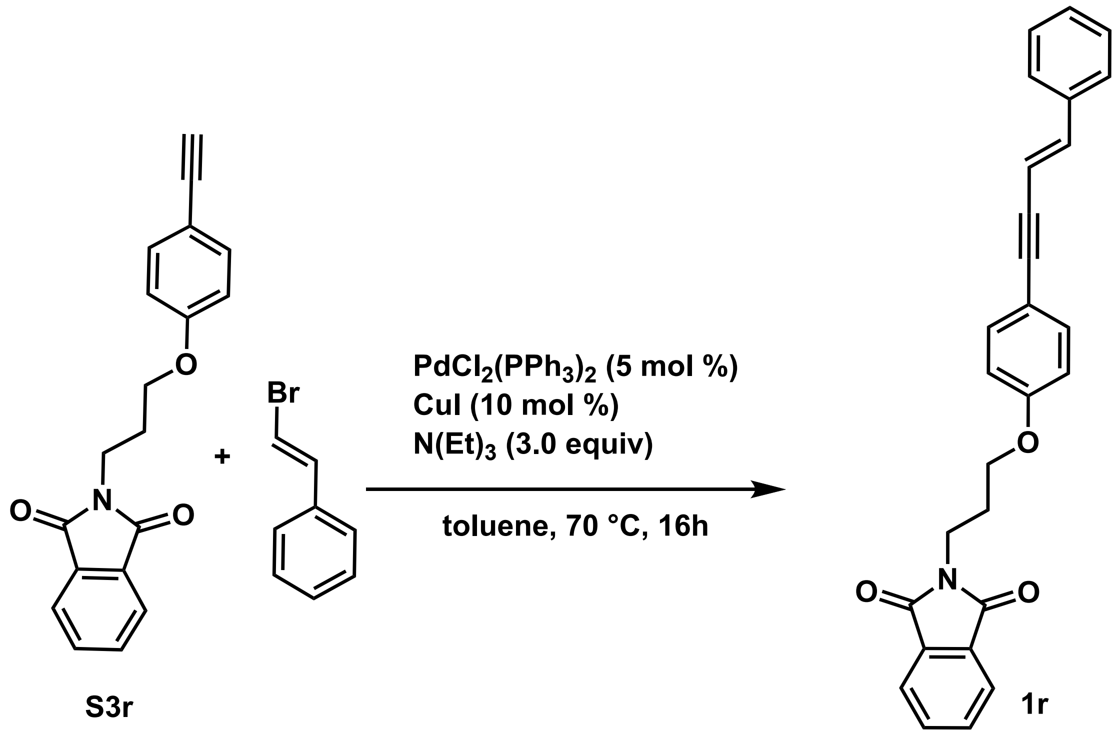

S3r **1r**

Scheme S10: Synthesis of 1r via Sonogashira cross-coupling conditions.

To a 6-dram vial with screw on septa cap: a stir bar, 2-(3-(4-ethynylphenoxy)propyl)isoindoline-1,3-dione **S3r** (1.0 equiv), $PdCl_2(PPh_3)_2$ (5 mol%) and CuI (10 mol%) were added. The reaction flask was put under inert atmosphere (N_2) using standard *Schlenk* technique. Toluene (5 mL), (*E*)-(2-bromovinyl)benzene (1.2 equiv) and triethylamine (3.0 equiv) were then added to the reaction flask. The reaction was heated to 70 °C and allowed to stir for 16 hours. Water (5 mL) and EtOAc (5 mL) were added. Two additional aliquots of EtOAc were added and extracted, then washed with brine. The organic phase was reduced under negative pressure and purified via column chromatography to afford (*E*)-2-(3-(4-(4-phenylbut-3-en-1-yn-1-yl)phenoxy)propyl)isoindoline-1,3-dione (**1r**) (107 mg, 263 µmol, 50%)) as a white amorphous solid. **^{1}H NMR** (400 MHz, $CDCl_3$) δ 7.83 (dd, *J* = 6.0, 3.2 Hz, 2H), 7.71 (dd, *J* = 5.6, 2.9 Hz, 2H), 7.40 (d, *J* = 7.4 Hz, 2H), 7.38 – 7.26 (m, 5H), 6.98 (d, *J* = 16.2 Hz, 1H), 6.74 (d, *J* = 8.3 Hz, 2H), 6.36 (d, *J* = 16.2 Hz, 1H), 4.03 (t, *J* = 6.1 Hz, 2H), 3.90 (t, *J* = 6.8 Hz, 2H), 2.18 (p, *J* = 6.5 Hz, 2H). **^{13}C NMR** (101 MHz, $CDCl_3$) δ 168.5, 158.8, 140.5, 136.6, 134.1, 133.0, 132.3, 129.3, 128.8, 127.0, 126.3, 123.4, 114.6, 108.5, 91.4, 86.1, 65.8, 35.5, 28.3. HRMS calcd for $C_{27}H_{22}NO_3^+$ $[M+H]^+$ 408.1600 m/z, found 408.1607 m/z. Δ 1.7150.

4. Optimization Table

Entry	HBpin loading	Catalyst	Cat. Loading	Ligand	Solvent	Temp °C	Isolated Yield
1	1.2 equiv	PBu_3	0.1 equiv	-	THF	25	0%
2	1.2 equiv	$Cu(OAc)_2$	0.1 equiv	Xantphos	THF	25	14%
3	1.2 equiv	$Cu(OAc)_2$	0.1 equiv	Xantphos	THF	40	31%
4	1.2 equiv	$Cu(OAc)_2$	0.1 equiv	Xantphos	Toluene	40	42%
5	1.2 equiv	$Cu(OAc)_2$	0.1 equiv	Xantphos	Toluene	50	49%
6	1.2 equiv	$Cu(OAc)_2$	0.1 equiv	Xantphos	Toluene	70	14%
7[b]	1.2 equiv	$Cu(OAc)_2$	0.1 equiv	IMes	Toluene	50	7%
8	1.2 equiv	$Cu(OAc)_2$	0.1 equiv	dppp	Toluene	50	trace
9	1.2 equiv	$Cu(OAc)_2$	0.1 equiv	DPEPhos	Toluene	50	9%
10	1.2 equiv	$Cu(OAc)_2$	0.1 equiv	dppbz	Toluene	50	25%
11[a]	1.2 equiv	$Cu(OAc)_2$	0.1 equiv	$P(NEt_2)_3$	Toluene	50	trace
12[a]	1.2 equiv	$Cu(OAc)_2$	0.1 equiv	$P(Bu)_3$	Toluene	50	5%
13[a]	1.2 equiv	$Cu(OAc)_2$	0.1 equiv	$P(Ph)_3$	Toluene	50	trace
14	1.4 equiv	$Cu(OAc)_2$	0.1 equiv	Xantphos	Toluene	50	25%
15	1.6 equiv	$Cu(OAc)_2$	0.1 equiv	Xantphos	Toluene	50	40%
16	2.0 equiv	$Cu(OAc)_2$	0.1 equiv	Xantphos	Toluene	50	55%
18	2.0 equiv	$Cu(OAc)_2$	0.2 equiv	Xantphos	Toluene	50	38%
19	2.0 equiv	$Cu(OAc)_2$	0.3 equiv	Xantphos	Toluene	50	46%
21	2.0 equiv	$CuCl_2$	0.1 equiv	Xantphos	Toluene	50	4%
22	2.0 equiv	CuOAc	0.1 equiv	Xantphos	Toluene	50	75%
23	2.0 equiv	CuI	0.1 equiv	Xantphos	Toluene	50	0%
24	2.0 equiv	CuCN	0.1 equiv	Xantphos	Toluene	50	8%
25	2.0 equiv	CuCl	0.1 equiv	Xantphos	Toluene	50	4%
26	1.2 equiv	CuOAc	0.1 equiv	Xantphos	Toluene	50	68%
28	2.0 equiv	CuOAc	0.1 equiv	Xantphos	THF	50	53%
29	2.0 equiv	CuOAc	0.1 equiv	Xantphos	MeCN	50	13%
30	2.0 equiv	CuOAc	0.1 equiv	Xantphos	1,4-dioxane	50	72%
31	2.0 equiv	CuOAc	0.1 equiv	Xantphos	Toluene	40	55%
32	2.0 equiv	CuOAc	0.1 equiv	Xantphos	Toluene	25	76%
33	2.0 equiv	CuOAc	0.1 equiv	PPh_3	Toluene	50	25%

34	2.0 equiv	CuOAc	0.1 equiv	DPEphos	Toluene	50	13%
35	2.0 equiv	CuOAc	0.05 equiv	Xantphos	Toluene	50	80%
36	2.0 equiv	CuOAc	0.05 equiv	Xantphos	Toluene	60	80%
37	2.0 equiv	CuOAc	0.05 equiv	Xantphos	Toluene	40	89%
38	2.0 equiv	CuOAc	0.05 equiv	Xantphos	Toluene	25	80%

Table S1: Full optimization table. a: 0.20 equiv of ligand used. b: 0.10 equiv of NaO*t*Bu used.

5. Characterization of 2-Boryl-1,3-Dienes

2-((1*Z*,3*Z*)-1,4-diphenylbuta-1,3-dien-2-yl)-4,4,5,5-tetrayl-1,3,2-dioxaborolane (2a).

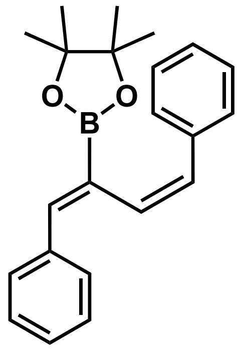

Synthesized according to general procedure G**.** Isolated in 0-5% EtOAc:Hex as a yellow oil in 73% yield (60.8 mg, 0.183 mmol). **^{1}H NMR** (400 MHz, $CDCl_3$) δ 7.64 – 7.57 (m, 2H), 7.44 – 7.40 (m, 2H), 7.39 – 7.33 (m, 2H), 7.32 – 7.27 (m, 3H), 7.25 – 7.23 (m, 1H), 7.23 – 7.17 (m, 1H), 6.72 (d, *J* = 11.6 Hz, 1H), 6.61 (dd, *J* = 11.7, 2.0 Hz, 1H), 0.99 (s, 12H). **^{13}C NMR** (101 MHz, $CDCl_3$) δ 143.4, 139.1, 138.0, 131.6, 130.6, 129.7, 128.9, 128.4, 128.3, 128.3, 127.1, 83.6, 24.6, (C-B not observed). **^{11}B NMR** (128 MHz, $CDCl_3$) δ 30.3. HRMS calcd for $C_{22}H_{26}BO_2^+$ $[M+H]^+$ 333.2026 m/z, found 333.2026 m/z. Δ 0.0000.

2-((1*Z*,3*Z*)-1,4-di-p-tolylbuta-1,3-dien-2-yl)-4,4,5,5-tetramethyl-1,3,2-dioxaborolane

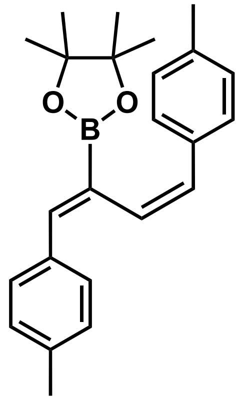

(2b) Synthesized according to general procedure G**.** Isolated in 0-5% EtOAc:Hex as a yellow oil in 78% yield (70.2 mg, 0.195 mmol). **^{1}H NMR** (400 MHz, $CDCl_3$) δ 7.51 (d, *J* = 8.4 Hz, 2H), 7.30 (d, *J* = 8.3 Hz, 2H), 7.19 – 7.14 (m, 3H), 7.10 (d, *J* = 0.9 Hz, 2H), 6.67 (d, *J* = 11.6 Hz, 1H), 6.56 (dd, *J* = 11.6, 2.0 Hz, 1H), 2.36 (s, 3H), 2.31 (s, 3H), 0.99 (s, 12H). **^{13}C NMR** (101 MHz, $CDCl_3$) δ 142.7, 137.9, 136.5, 136.0, 135.0, 131.0, 129.7, 129.4, 128.7, 128.7, 128.5, 24.3, 21.2, 21.0, (*C*-B not observed). **^{11}B NMR** (128 MHz, $CDCl_3$) δ 30.7. HRMS calcd for $C_{24}H_{30}BO_2^+$ $[M+H]^+$ 361.2339 m/z, found 361.2338 m/z. Δ -0.2768.

2-((1*Z*,3*Z*)-1,4-di-m-tolylbuta-1,3-dien-2-yl)-4,4,5,5-tetramethyl-1,3,2-dioxaborolane (2c). Synthesized according to general procedure G. Isolated in 0-5% EtOAc:Hex as a

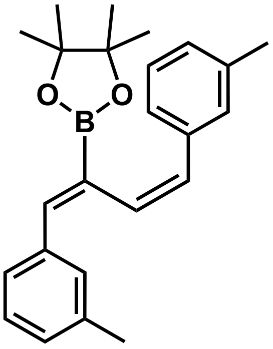

yellow oil in 69% yield (44.3 mg, 0.123 mmol). **^{1}H NMR** (400 MHz, $CDCl_3$) δ 7.46 – 7.38 (m, 2H), 7.25 – 7.22 (m, 2H), 7.21 – 7.14 (m, 3H), 7.11 (d, *J* = 7.6 Hz, 1H), 7.01 (dt, *J* = 7.2, 2.0 Hz, 1H), 6.70 – 6.65 (m, 1H), 6.59 (dd, *J* = 11.6, 2.0 Hz, 1H), 2.36 (s, 3H), 2.32 (s, 3H), 0.98 (s, 12H). **^{13}C NMR** (101 MHz, $CDCl_3$) δ 142.9, 138.8, 137.7, 137.6, 137.5, 131.2, 130.2, 130.1, 129.2, 128.7, 128.1, 127.9, 127.6, 126.6, 125.8, 83.2, 24.3, 21.3, 21.2, (*C*-B not observed). **^{11}B NMR** (128 MHz, $CDCl_3$) δ 31.5. HRMS calcd for $C_{24}H_{30}BO_2^+$ $[M+H]^+$ 361.2339 m/z, found 361.2345 m/z. Δ 1.6610.

2-((1*Z*,3*Z*)-1,4-bis(4-(tert-butyl)phenyl)buta-1,3-dien-2-yl)-4,4,5,5-tetramethyl-1,3,2-dioxaborolane (2d). Synthesized according to general procedure G**.** Isolated in 0-5% EtOAc:Hex as a yellow oil in 64% yield (71.0 mg, 0.160 mmol). **^{1}H NMR** (400 MHz, $CDCl_3$) δ 7.62 – 7.51 (m, 2H), 7.39 – 7.35 (m, 2H), 7.35 – 7.29 (m, 4H), 7.17 (s, 1H), 6.69 (d, *J* = 11.3 Hz, 1H), 6.60 (dd, *J* = 11.5, 2.0 Hz, 1H), 1.33 (s, 9H), 1.29 (s, 9H), 0.96 (s, 12H). **^{13}C NMR** (101 MHz, $CDCl_3$) δ 151.3, 150.1, 142.9, 136.5, 135.4, 131.2, 130.4, 129.5, 128.6, 125.3, 125.2, 83.5, 34.8, 34.7, 31.4, 31.4, 24.6, (*C*-B not observed). **^{11}B NMR** (128 MHz, $CDCl_3$) δ 31.3. HRMS calcd for $C_{30}H_{41}BO_2^+$ $[M+H]^+$ 445.3278 m/z, found 445.3269 m/z. Δ -2.0210.

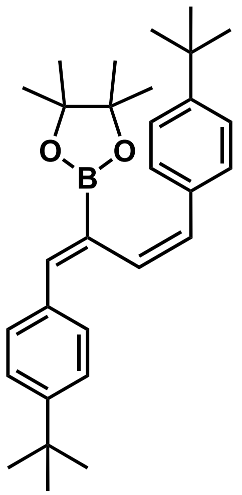

2-((1*Z*,3*Z*)-1,4-bis(4-propylphenyl)buta-1,3-dien-2-yl)-4,4,5,5-tetramethyl-1,3,2-dioxaborolane (2e). Synthesized according to general procedure G**.** Isolated in 0-5% EtOAc:Hex as a yellow oil in 69% yield (72.0 mg, 0.173 mmol). **^{1}H NMR** (400 MHz, $CDCl_3$) δ 7.54 – 7.51 (m, 2H), 7.32 (dd, *J* = 7.9, 0.5 Hz, 2H), 7.19 – 7.14 (m, 3H), 7.09 (d, *J* = 8.0 Hz, 2H), 6.67 (d, *J* = 11.8 Hz, 1H), 6.57 (dd, *J* = 11.5, 2.1 Hz, 1H), 2.56 (dt, *J* = 19.8, 7.9 Hz, 4H), 1.70 – 1.55 (m, 4H), 0.98 (s, 12H), 0.97 – 0.91 (m, 6H). **^{13}C NMR** (126 MHz, $CDCl_3$) δ 143.0, 143.0, 141.8, 136.7, 135.6, 131.3, 130.1, 129.7, 128.8, 128.5, 128.4, 83.5, 38.1, 38.0, 24.9, 24.6, 24. 6, 14.0, 14.0, (*C*-B not observed). **^{11}B NMR** (128 MHz, $CDCl_3$) δ 30.5. HRMS calcd for $C_{28}H_{38}BO_2^+$ $[M+H]^+$ 417.2965 m/z, found 417.2974 m/z. Δ 2.1567.

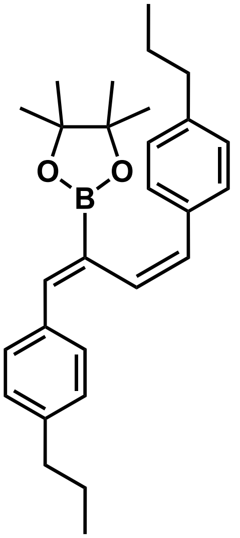

2-((1*Z*,3*Z*)-1,4-bis(4-methoxyphenyl)buta-1,3-dien-2-yl)-4,4,5,5-tetramethyl-1,3,2-dioxaborolane (2f), Synthesized according to general procedure G. Isolated from 0-10% EtOAc:Hex as a yellow oil in 92% yield (90.2 mg, 0.230 mmol), **^{1}H NMR** (400 MHz, $CDCl_3$) δ 7.58 (d, *J* = 8.8 Hz, 2H), 7.35 (d, *J* = 8.8 Hz, 2H), 7.15 (d, *J* = 1.5 Hz, 1H), 6.88 (d, *J* = 8.8 Hz, 2H), 6.83 (d, *J* = 8.7 Hz, 2H), 6.64 (d, *J* = 11.5 Hz, 1H), 6.51 (dd, *J* = 11.5, 2.0 Hz, 1H), 3.82 (s, 3H), 3.78 (s, 3H), 1.01 (s, 12H). **^{13}C NMR** (101 MHz, $CDCl_3$) δ 159.6, 158.9, 142.6, 132.1, 131.2, 131.0, 130.6, 130.1, 129.3, 113.8, 113.7, 83.5, 55.5, 55.4, 24.7, (*C*-B not observed). **^{11}B NMR** (128 MHz, $CDCl_3$) δ 29. 8. HRMS calcd for $C_{24}H_{30}BO_4^+$ $[M+K]^+$ 431.1796 m/z, found 431.1788 m/z. Δ -1.8554.

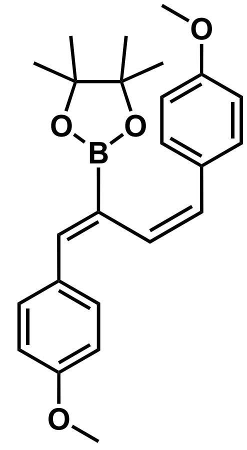

2-((1*Z*,3*Z*)-1,4-bis(3-methoxyphenyl)buta-1,3-dien-2-yl)-4,4,5,5-tetramethyl-1,3,2-dioxaborolane (2g), Synthesized according to general procedure G. Isolated from 0-10% EtOAc:Hex as a yellow oil in 89% yield (87.3 mg, 0.223 mmol). **^{1}H NMR** (400 MHz, $CDCl_3$) δ 7.30 – 7.23 (m, 1H), 7.22 – 7.14 (m, 4H), 7.02 – 6.95 (m, 2H), 6.89 – 6.81 (m, 1H), 6.80 – 6.72 (m, 1H), 6.69 (d, *J* = 11.7 Hz, 1H), 6.63 (dd, *J* = 11.7, 1.5 Hz, 1H), 3.80 (s, 3H), 3.78 (s, 3H), 1.00 (s, 12H). **^{13}C NMR** (101 MHz, $CDCl_3$) δ 159.7, 159.5, 142.9, 140.4, 139.3, 131.4, 130.9, 129.5, 129.3, 122.4, 121.6, 114.8, 114.1, 113.7, 113.4, 83.6, 55.4, 55.2, 24.6, (*C*-B not observed). **^{11}B NMR** (128 MHz, $CDCl_3$) δ 30.2. HRMS calcd for $C_{24}H_{30}BO_4^+$ $[M+H]^+$ 393.2237 m/z, found 393.2243 m/z. Δ 1.5259.

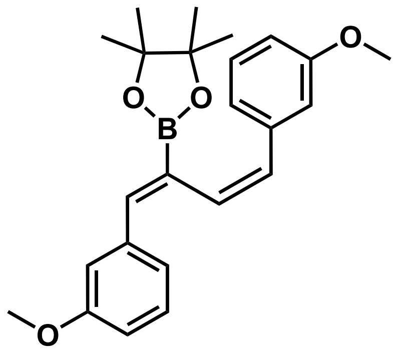

2-((1*Z*,3*Z*)-1,4-bis(4-(benzyloxy)phenyl)buta-1,3-dien-2-yl)-4,4,5,5-tetramethyl-1,3,2-dioxaborolane (2h) - Synthesized according to general procedure G. Isolated from 0-10% EtOAc:Hex as a yellow oil in 61% yield (83.0 mg, 0.153 mmol). **^{1}H NMR** (400 MHz, $CDCl_3$) δ 7.57 (d, *J* = 8.8 Hz, 2H), 7.47 – 7.29 (m, 12H), 7.14 (d, *J* = 1.7 Hz, 1H), 6.95 (d, *J* = 8.8 Hz, 2H), 6.89 (d, *J* = 8.7 Hz, 2H), 6.63 (d, *J* = 11.6 Hz, 1H), 6.51 (dd, *J* = 11.6, 2.1 Hz, 1H), 5.08 (s, 2H), 5.06 (s, 2H), 0.99 (s, 12H). **^{13}C NMR** (101 MHz, $CDCl_3$) δ 158.9, 158.0, 142.6, 137.0, 132.3, 131.3, 131.2, 130.7, 130.1, 129.3, 128.9, 128.8, 128.7, 128.2, 128.0, 127.6, 127.4, 114.9, 114.6, 83.5, 70.1, 24.7, (*C*-B not observed). **^{11}B NMR** (128 MHz, $CDCl_3$) δ 28.1. HRMS calcd for $C_{36}H_{38}BO_4^+$ [M+H]$^+$ 545.2863 m/z, found 545.2864 m/z. Δ 0.1834.

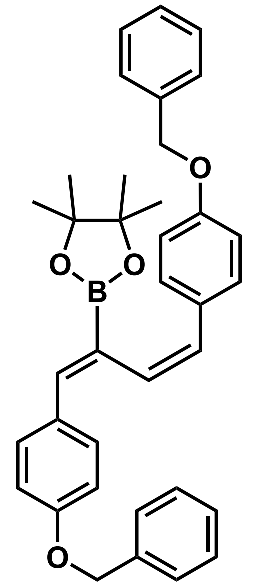

2-((1*Z*,3*Z*)-1,4-bis(4-(methylthio)phenyl)buta-1,3-dien-2-yl)-4,4,5,5-tetramethyl-1,3,2-dioxaborolane (2i), Synthesized according to general procedure G. Isolated from 0-10% EtOAc:Hex as a yellow oil in 58% yield (61.7 mg, 0.145 mmol). ^{1}H NMR (400 MHz, $CDCl_3$) δ 7.56 – 7.48 (m, 2H), 7.36 – 7.29 (m, 2H), 7.23 – 7.17 (m, 4H), 7.16 (d, *J* = 1.6 Hz, 1H), 6.65 (d, *J* = 11.6 Hz, 1H), 6.56 (dd, *J* = 11.6, 2.0 Hz, 1H), 2.49 (s, 3H), 2.45 (s, 3H), 1.01 (s, 12H). ^{13}C NMR (101 MHz, $CDCl_3$) δ 142.8, 139.0, 137.2, 136.3, 134.7, 130.9, 130.4, 130.1, 129.3, 126.9, 125.9, 83.7, 24.6, 16.3, 15.6 (*C*-B not observed).^{11}B NMR (128 MHz, $CDCl_3$) δ 31.7. HRMS calcd for $C_{24}H_{30}BO_2S_2^+$ [M+H]$^+$ 425.1780 m/z, found 425.1768 m/z. Δ -2.8224.

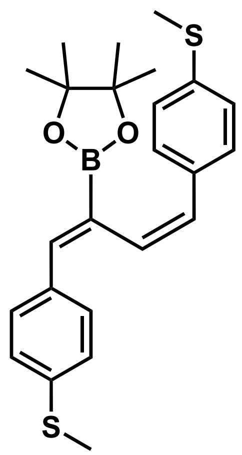

2-((1*Z*,3*Z*)-1,4-bis(4-(trifluoromethyl)phenyl)buta-1,3-dien-2-yl)-4,4,5,5-tetramethyl-1,3,2-dioxaborolane (2j), Synthesized according to general procedure G. Isolated from 0-10% EtOAc:Hex as a yellow solid in 49% yield (57.6 mg, 0.123 mmol). **^{1}H NMR** (400 MHz, $CDCl_3$) δ 7.67 (d, *J* = 8.2 Hz, 2H), 7.61 (d, *J* = 8.2 Hz, 2H), 7.56 (d, *J* = 8.2 Hz, 2H), 7.48 (d, *J* = 8.2 Hz, 2H), 7.29 (s, 1H), 6.76 (d, *J* = 11.7 Hz, 1H), 6.65 (dd, *J* = 11.8, 2.0 Hz, 1H), 0.99 (s, 12H). **^{13}C NMR** (126 MHz, $CDCl_3$) δ 142.7, 140.8, 136.5, 131.9, 130.9, 130.1, 129.6, 128.8, 127.1, 125.3 (q, *J* = 5.4 Hz), 125.2 (q, *J* = 5.6 Hz), 123.1 (q, *J* = 271.4 Hz), 123.0 (q, *J* = 271.4 Hz), 83.8, 24.4 (*C*-B not observed).**^{11}B NMR** (128 MHz, $CDCl_3$) δ 30.0. **^{19}F NMR** (376 MHz, $CDCl_3$) δ -62.53, -62.54. HRMS calcd for $C_{24}H_{24}BF_6O_2^+$ $[M+NH_4]^+$ 486.2039 m/z, found 486.2020 m/z. Δ -3.9078.

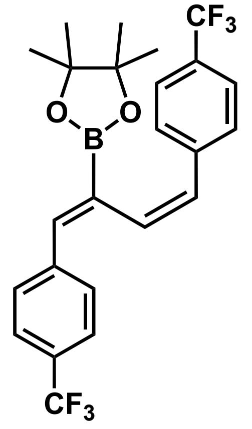

2-((1*Z*,3*Z*)-1,4-bis(4-(trifluoromethoxy)phenyl)buta-1,3-dien-2-yl)-4,4,5,5-tetramethyl-1,3,2-dioxaborolane (2k), Synthesized according to general procedure G. Isolated from 0-10% EtOAc:Hex as a yellow oil in 67% yield (82.8 mg, 0.168 mmol). **^{1}H NMR** (400 MHz, $CDCl_3$) δ 7.60 (d, *J* = 8.7 Hz, 2H), 7.40 (d, *J* = 8.6 Hz, 2H), 7.21 (s, 1H), 7.19 (d, *J* = 7.9 Hz, 2H), 7.15 (d, *J* = 7.8 Hz, 2H), 6.69 (d, *J* = 11.7 Hz, 1H), 6.58 (dd, *J* = 11.7, 2.0 Hz, 1H), 1.00 (s, 12H). **^{13}C NMR** (126 MHz, $CDCl_3$) δ 148.9, 148.2, 142.3, 137.6, 136.2, 130.9, 130.8, 130.5, 130.0, 121.5 (q, *J* = 256.7 Hz), 121.1, 120.6, 119.4 (q, *J* = 257.6 Hz), 83.7, 24.4(*C*-B not observed). **^{11}B NMR** (128 MHz, $CDCl_3$) δ 28.4. **^{19}F NMR** (376 MHz, $CDCl_3$) δ -57.7, -58.0. HRMS calcd for $C_{24}H_{24}BF_6O_4^+$ $[M+NH_4]^+$, 518.1937 m/z found 518.1950 m/z. Δ 2.5087.

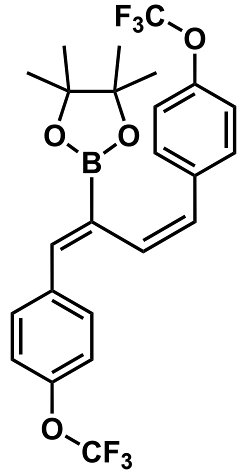

2-((1*Z*,3*Z*)-1,4-bis(4-fluorophenyl)buta-1,3-dien-2-yl)-4,4,5,5-tetramethyl-1,3,2-dioxaborolane (2l) – Modified from general procedure G: 1.2 equivalence pinacolborane, reaction time 1 hour. Isolated from 0-10% EtOAc:Hex as a yellow oil in 71% yield (65.3 mg, 0.178 mmol). **^{1}H NMR** (500 MHz, $CDCl_3$) δ 7.57 (dd, *J* = 8.7, 5.6 Hz, 2H), 7.36 (dd, *J* = 8.6, 5.6 Hz, 2H), 7.19 (d, *J* = 1.6 Hz, 1H), 7.04 (dd, *J* = 9.1, 8.7 Hz, 2H), 6.98 (dd, *J* = 9.0, 8.8 Hz, 2H), 6.66 (d, *J* = 11.6 Hz, 1H), 6.53 (dd, *J* = 11.6, 2.1 Hz, 1H), 1.02 (s, 12H). **^{13}C NMR** (126 MHz, $CDCl_3$) δ 162.6 (d, *J* = 248.9 Hz),162.1 (d, *J* = 246.6 Hz), 142.6, 135.1 (d, *J* = 3.2 Hz), 134.0 (d, *J* = 3.4 Hz), 131.4 (d, *J* = 8.1 Hz), 130.6, 130.4 (d, *J* = 7.8 Hz), 130.1 (d, *J* = 1.6 Hz), 115.4 (d, *J* = 9.5 Hz), 115.2 (d, *J* = 9.5 Hz), 83.7, 24.6, (*C*-B not observed). **^{11}B NMR** (128 MHz, $CDCl_3$) δ

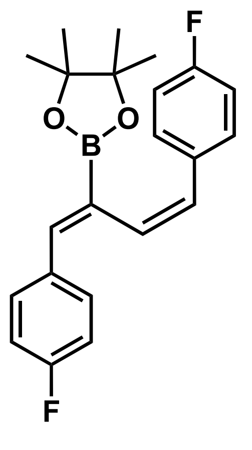

30.3. **^{19}F NMR** (376 MHz, $CDCl_3$) δ -112.62, -114.97. HRMS calcd for $C_{22}H_{24}BF_2O_2^+$ [M+H]$^+$ 369.1837 m/z, found 369.1825 m/z. Δ -3.2504.

2-((1*Z*,3*Z*)-1,4-bis(3-fluorophenyl)buta-1,3-dien-2-yl)-4,4,5,5-tetramethyl-1,3,2-dioxaborolane (2m) – Modified from general procedure G: 1.2 equivalence pinacolborane, reaction time 1 hour. Isolated from 0-10% EtOAc:Hex as a yellow oil in 92% yield (84.6 mg, 0.230 mmol). **^{1}H NMR** (400 MHz, $CDCl_3$) δ 7.36 – 7.27 (m, 3H), 7.25 – 7.22 (m, 1H), 7.21 (d, *J* = 2.9 Hz, 1H), 7.16 (dt, *J* = 7.9, 1.5 Hz, 1H), 7.09 (ddd, *J* = 10.3, 2.6, 1.6 Hz, 1H), 7.03 – 6.96 (m, 1H), 6.91 (tdd, *J* = 8.4, 2.6, 1.1 Hz, 1H), 6.68 (d, *J* = 11.8 Hz, 1H), 6.60 (dd, *J* = 11.8, 2.0 Hz, 1H), 1.02 (s, 12H). **^{13}C NMR** (101 MHz, $CDCl_3$) δ 163.1 (d, *J* = 245.2 Hz), 162.8 (d, *J* = 245.1 Hz), 142.6 (d, *J* = 2.5 Hz), 141.1 (d, *J* = 7.7 Hz), 139.9 (d, *J* = 7.7 Hz), 131.2, 130.9 (d, *J* = 2.4 Hz), 129.9 (d, *J* = 8.2 Hz), 129.8 (d, *J* = 8.3 Hz) , 125.6 (d, *J* = 2.8 Hz), 124. 7 (d, *J* = 2.8 Hz), 116.1 (d, *J* = 21.7 Hz), 115.5 (d, *J* = 21.6 Hz), 115.3 (d, *J* = 21.4 Hz), 114.1 (d, *J* = 21.2 Hz), 83.8, 24.6, (*C*-B not observed). **^{11}B NMR** (128 MHz, $CDCl_3$) δ 29.35. **^{19}F NMR** (376 MHz, $CDCl_3$) δ -113.27, -113.68. HRMS calcd for $C_{22}H_{24}BF_2O_2^+$ [M+H]$^+$ 369.1837 m/z, found 369.1841 m/z. Δ 1.0835.

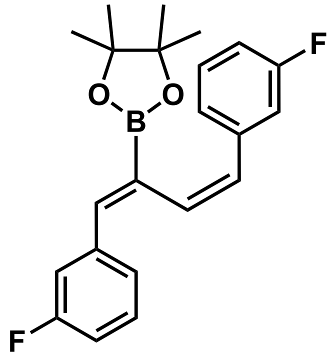

2-((1*Z*,3*Z*)-1,4-bis(4-chlorophenyl)buta-1,3-dien-2-yl)-4,4,5,5-tetramethyl-1,3,2-dioxaborolane (2n), Synthesized according to general procedure G. Isolated from 0-10% EtOAc:Hex as a yellow oil in 65% yield (65.0 mg, 0.163 mmol). **^{1}H NMR** (400 MHz, $CDCl_3$) δ 7.51 (d, *J* = 8.5 Hz, 2H), 7.31 (d, *J* = 9.2 Hz, 4H), 7.28 – 7.23 (m, 2H), 7.18 (d, *J* = 2.0 Hz, 1H), 6.65 (d, *J* = 11.7 Hz, 1H), 6.55 (dd, *J* = 11.7, 2.0 Hz, 1H), 1.02 (s, 12H). **^{13}C NMR** (101 MHz, $CDCl_3$) δ 142.6, 137.4, 136.2, 134.2, 133.0, 130.9, 130.7, 130.7, 130.1, 128.6, 128.6, 83.8, 24.6, (*C*-B not observed). **^{11}B NMR** (128 MHz, $CDCl_3$) δ 29.9. HRMS calcd for $C_{22}H_{24}BCl_2O_2^+$ [M+CH_3OH+H]$^+$ 433.1509 m/z, found 433.1491 m/z. Δ -4.1556.

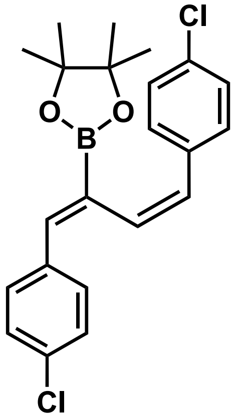

2-((1*Z*,3*Z*)-1,4-bis(3-chlorophenyl)buta-1,3-dien-2-yl)-4,4,5,5-tetramethyl-1,3,2-dioxaborolane (2o), Synthesized according to general procedure G. Isolated from 0-10% EtOAc:Hex as a yellow oil in 55**%** yield. (55.0 mg, 0.138 mmol) **,** ^{1}**H NMR** (400 MHz, $CDCl_3$) δ 7.56 (s, 1H), 7.45 – 7.42 (m, 1H), 7.37 (s, 1H), 7.29 – 7.27 (m, 2H), 7.25 – 7.24 (m, 1H), 7.22 (s, 1H), 7.20 – 7.17 (m, 2H), 6.66 (d, *J* = 11.8 Hz, 1H), 6.59 (dd, *J* = 11.7, 2.0 Hz, 1H), 1.02 (s, 12H). ^{13}**C NMR** (101 MHz, $CDCl_3$) δ 142.5, 140.7, 139.4, 134.5, 134.3, 131.2, 130.8, 129.7, 129.6, 129.4, 128.7, 128.4, 127.9, 127.3, 127.1, 83.9, 24.6, (*C*-B not observed). ^{11}**B NMR** (128 MHz, $CDCl_3$) δ 30.2. HRMS calcd for $C_{45}H_{51}B_2Cl_4O_5^+$ $[2M+MeOH+H]^+$ 833.2671 m/z, found 833.2650 m/z. Δ -2.5202.

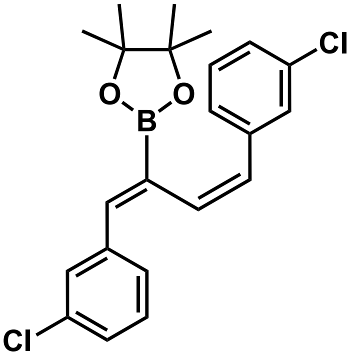

2-((1*Z*,3*Z*)-1,4-bis(2-chlorophenyl)buta-1,3-dien-2-yl)-4,4,5,5-tetramethyl-1,3,2-dioxaborolane (2p), Synthesized according to general procedure G. Isolated from 0-10% EtOAc:Hex as a yellow oil in 34**%** yield (34.1 mg ,0.085 mmol). ^{1}**H NMR** (400 MHz, $CDCl_3$) δ 7.71 – 7.62 (m, 1H), 7.45 – 7.34 (m, 4H), 7.25 – 7.22 (m, 2H), 7.20 – 7.16 (m, 2H), 6.78 (d, *J* = 11.4 Hz, 1H), 6.54 (dd, *J* = 11.6, 1.9 Hz, 1H), 0.97 (s, 12H). ^{13}**C NMR** (101 MHz, $CDCl_3$) δ 140.3, 137.4, 135.7, 134.0, 131.2, 130.9, 130.6, 129.7, 129.6, 129.5, 129.4, 128.6, 126.7, 126.3, 83.7, 24.6, (*C*-B not observed). ^{11}**B NMR** (128 MHz, $CDCl_3$) δ 30.1. HRMS calcd for $C_{22}H_{23}BCl_2NaO_2^+$ $[M+Na]^+$ 423.1066 m/z, found 423.1053 m/z. Δ -3.0725.

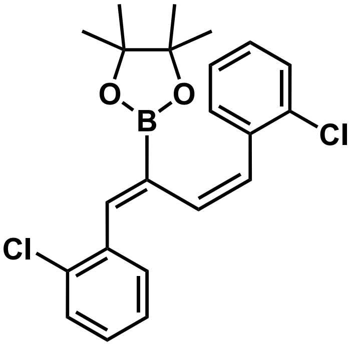

2-((1*Z*,3*Z*)-1,4-di(thiophen-2-yl)buta-1,3-dien-2-yl)-4,4,5,5-tetramethyl-1,3,2-dioxaborolane (2q), Synthesized according to general procedure G. Isolated from 0-10% EtOAc:Hex as a yellow oil in 76% yield (65.4 mg, 0.190 mmol). ^{1}**H NMR** (400 MHz, $CDCl_3$) δ 7.50 (dd, *J* = 2.2, 1.0 Hz, 1H), 7.34 (d, *J* = 5.1 Hz, 1H), 7.25 – 7.23 (m, 1H), 7.17 (dd, *J* = 5.1, 1.2 Hz, 1H), 7.06 – 7.01 (m, 2H), 6.95 (dd, *J* = 5.1, 3.6 Hz, 1H), 6.89 – 6.85 (m, 1H), 6.52 (dd, *J* = 11.4, 2.2 Hz, 1H), 1.15 (s, 12H). ^{13}**C NMR** (101 MHz, $CDCl_3$) δ 141.6, 141.4, 137.3, 130.0, 128.6, 128.4, 127.5, 127.1, 127.1, 125.9, 125.2, 83.8, 24.8, (*C*-B not observed). ^{11}**B NMR** (128 MHz, $CDCl_3$) δ 30.1. HRMS calcd for $C_{18}H_{22}BO_2S_2^+$ $[M+H]^+$ 345.1154 m/z, found 345.1158 m/z. Δ 1.1590.

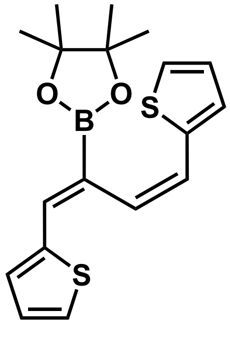

2-(3-(4-((1*Z*,3*E*)-4-phenyl-2-(4,4,5,5-tetramethyl-1,3,2-dioxaborolan-2-yl)buta-1,3-dien-1-yl)phenoxy)propyl)isoindoline-1,3-dione

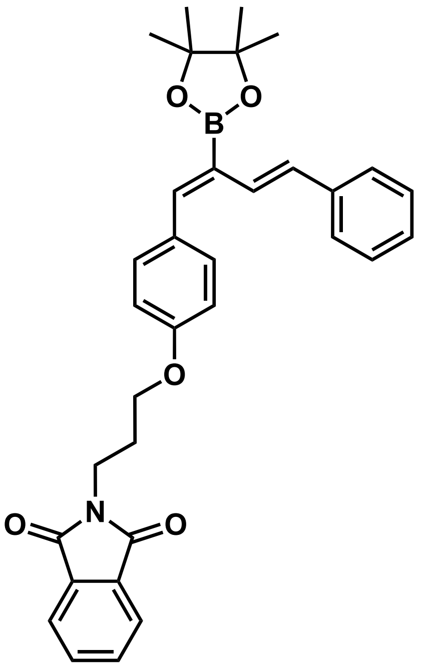

(2r) – Synthesized according to general procedure G. Isolated from 5-30% EtOAc:Hex as a white solid in 55% yield (73.6 mg, 0.138 mmol). **^{1}H NMR** (400 MHz, $CDCl_3$) δ 7.81 (dd, *J* = 5.5, 3.0 Hz, 2H), 7.69 (dd, *J* = 5.5, 3.0 Hz, 2H), 7.38 (d, *J* = 7.2 Hz, 2H), 7.37 – 7.25 (m, 5H), 7.25 (d, *J* = 7.1 Hz, 1H), 6.96 (d, *J* = 16.2 Hz, 1H), 6.72 (d, *J* = 8.7 Hz, 2H), 6.34 (d, *J* = 16.2 Hz, 1H), 4.01 (t, *J* = 6.0 Hz, 2H), 3.88 (t, *J* = 6.8 Hz, 2H), 2.16 (p, *J* = 6.4 Hz, 2H), 1.24 (s, 12H). **^{13}C NMR** (101 MHz, $CDCl_3$) δ 168.5, 158.8, 140.5, 136.6, 134.1, 133.0, 132.2, 128.8, 128.5, 126.3, 115.7, 114.6, 108.5, 92.0, 87.7, 65.8, 35.5, 28.3, 24.6, 24.2, (*C*-B not observed). **^{11}B NMR** (128 MHz, $CDCl_3$) δ 22.4. HRMS calcd for $C_{33}H_{35}BNO_5^+$ $[M+H]^+$ 536.2608 m/z, found 536.2617 m/z. Δ 1.6783.

2-((1*Z*,3*E*)-1-(4-methoxyphenyl)-4-phenylbuta-1,3-dien-2-yl)-4,4,5,5-tetramethyl-1,3,2-dioxaborolane (2s) – Synthesized according to general procedure G. Isolated from 0-15% EtOAc:Hex as a white solid in 60% yield (54.3 mg, 0.150 mmol). **^{1}H NMR** (400 MHz, $CDCl_3$) δ 7.45 – 7.27 (m, 8H), 7.24 – 7.17 (m, 2H), 6.91 (d, *J* = 8.8 Hz, 2H), 3.84 (s, 3H), 1.37 (s, 12H). **^{13}C NMR** (101 MHz, $CDCl_3$) δ 159.3, 143.3, 138.7, 133.1, 131.5, 130.6, 128.6, 128.1, 127.2, 126.6, 113.8, 83.7, 55.4, 25.0. (*C*-B not observed). **^{11}B NMR** (128 MHz, $CDCl_3$) δ 30.9. HRMS calcd for $C_{23}H_{28}BO_3^+$ $[M+H]^+$ 363.2131 m/z, found 363.2132 m/z. Δ 0.2753.

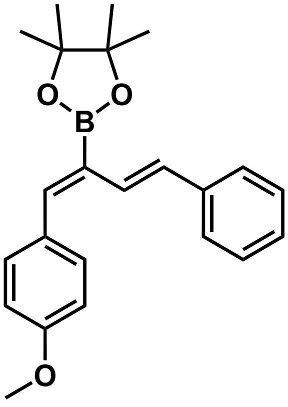

N,N-dimethyl-4-((1*Z*,3*E*)-4-phenyl-2-(4,4,5,5-tetramethyl-1,3,2-dioxaborolan-2-yl)buta-1,3-dien-1-yl)aniline (2t) – Synthesized according to general procedure G. Isolated from 0-15% EtOAc:Hex as a yellow solid in 32% yield (30.0 mg, 0.080 mmol). **^{1}H NMR** (400 MHz, $CDCl_3$) δ 7.45 – 7.34 (m, 5H), 7.32 – 7.27 (m, 2H), 7.25 – 7.19 (m, 2H), 7.19 – 7.14 (m, 1H), 6.71 (d, *J* = 8.8 Hz, 2H), 3.00 (s, 6H), 1.36 (s, 12H). **^{13}C NMR** (101 MHz, $CDCl_3$) δ 150.0, 144.3, 139.0, 132.1, 131.6, 128.9, 128.6, 126.9, 126.5, 123.5, 111.8, 83.5, 40.4, 25.0, (*C*-B not observed). **^{11}B NMR** (128 MHz, $CDCl_3$) δ 30.7. HRMS calcd for $C_{24}H_{31}BNO_2^+$ $[M+H]^+$ 376.2448 m/z, found 376.2447 m/z. Δ -0.2658.

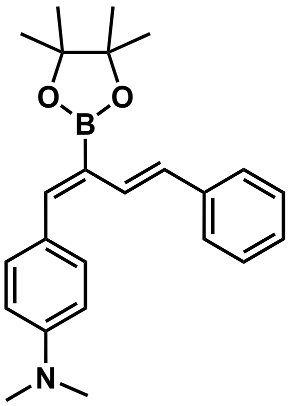

trimethyl((1*Z*,3*E*)-4-phenyl-2-(4,4,5,5-tetramethyl-1,3,2-dioxaborolan-2-yl)buta-1,3-dien-1-yl)silane (2u) – Synthesized according to general procedure G. Isolated from 0-5% EtOAc:Hex as a colorless oil in 52% yield (42.6 mg, 0.130 mmol). **^{1}H NMR** (400 MHz, $CDCl_3$) δ 7.60 (d, *J* = 10.8 Hz, 1H), 7.43 (d, *J* = 7.7 Hz, 2H), 7.40 – 7.32 (m, 2H), 7.28 (d, *J* = 7.3 Hz, 1H), 7.24 – 7.16 (m, 1H), 6.74 (d, *J* = 15.2 Hz, 1H), 1.30 (s, 12H), 0.30 (s, 9H). **^{13}C NMR** (101 MHz, $CDCl_3$) δ 156.1, 137.9, 137.2, 129.7, 128.8, 128.3, 127.0, 83.1, 24.9, 1.5, (*C*-B not observed). **^{11}B NMR** (128 MHz, $CDCl_3$) δ 31.6. HRMS calcd for $C_{19}H_{30}BO_2Si^+$ $[M+H]^+$ 329.2108 m/z, found 329.2116 m/z. Δ 2.4301.

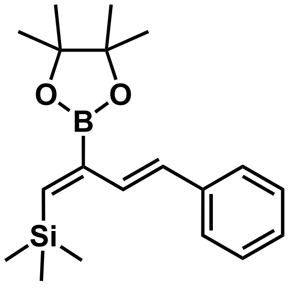

4,4,5,5-tetramethyl-2-((1*E*,3*Z*)-1-phenylpenta-1,3-dien-3-yl)-1,3,2-dioxaborolane (2v) – Synthesized according to general procedure G. Isolated from 0-10% EtOAc:Hex as a colorless oil in 14% yield (9.45 mg, 0.035 mmol). **^{1}H NMR** (400 MHz, $CDCl_3$) δ 7.45 (d, *J* = 6.8 Hz, 2H), 7.31 (t, *J* = 7.5 Hz, 2H), 7.22 (d, *J* = 13.7 Hz, 1H), 7.18 (d, *J* = 12.3 Hz, 1H), 7.14 (d, *J* = 6.7 Hz, 1H), 6.51 (q, *J* = 7.2 Hz, 1H), 1.94 (d, *J* = 7.1 Hz, 3H), 1.32 (s, 12H). **^{13}C NMR** (101 MHz, $CDCl_3$) δ 142.7, 138.7, 132.1, 128.6, 127.2, 126.5, 126.2, 83.4, 25.0, 14.9. (*C*-B not observed). **^{11}B NMR** (128 MHz, $CDCl_3$) δ 30.5. HRMS calcd for $C_{17}H_{24}BO_2^+$ $[M+H]^+$ 271.1869 m/z, found 271.1873 m/z. Δ 1.4750.

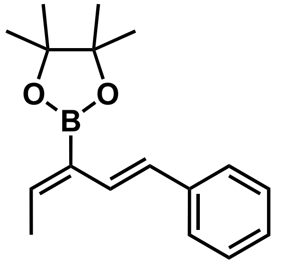

4-((1*Z*,3*E*)-4-phenyl-2-(4,4,5,5-tetramethyl-1,3,2-dioxaborolan-2-yl)buta-1,3-dien-1-yl)benzyl (R)-2-(6-methoxynaphthalen-2-yl)propanoate (2w) – Synthesized according to general procedure G. Isolated from 0-20% EtOAc:Hex as a yellow oil in 41% yield (58.8 mg, 0.103 mmol). **^{1}H NMR** (400 MHz, $CDCl_3$) δ 7.71 – 7.65 (m, 3H), 7.41 (d, *J* = 1.9 Hz, 1H), 7.39 (dt, *J* = 6.6, 1.3 Hz, 2H), 7.32 (d, *J* = 8.0 Hz, 2H), 7.28 (d, *J* = 8.0 Hz, 2H), 7.29 – 7.17 (m, 6H), 7.12 (dd, *J* = 8.8, 2.5 Hz, 1H), 7.09 (d, *J* = 2.5 Hz, 1H), 5.16 (d, *J* = 12.7 Hz, 1H), 5.10 (d, *J* = 12.7 Hz, 1H), 3.93 (q, *J* = 7.5 Hz, 1H), 3.89 (s, 3H), 1.61 (d, *J* = 7.1 Hz, 3H), 1.37 (s, 12H). **^{13}C NMR** (101 MHz, $CDCl_3$) δ 174.6, 157.8, 142.8, 138.4, 137.7, 135.7, 135.4, 134.0, 133.9, 130.0, 129.4, 129.1, 128.6, 127.6, 127.4, 127.3, 126.7, 126.4, 126.2, 119.1, 105.7, 83.8, 66.3, 60.5, 45.6, 25.0, 18.7, 14.3, (*C*-B not observed). **^{11}B NMR** (128 MHz, $CDCl_3$) δ 28.2. HRMS calcd for $C_{37}H_{39}BNaO_5^+$ $[M+Na]^+$ 597.2788 m/z, found 597.2792 m/z. Δ 0.6697.

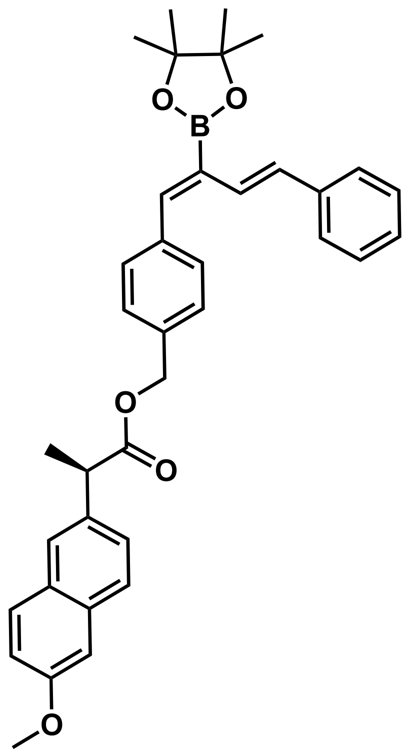

4-((1*Z*,3*E*)-4-phenyl-2-(4,4,5,5-tetramethyl-1,3,2-dioxaborolan-2-yl)buta-1,3-dien-1-yl)benzyl 5-(2,5-dimethylphenoxy)-2,2-dimethylpentanoate (2x) – Synthesized according to general procedure G. Isolated from 0-20% EtOAc:Hex as a yellow oil in 49% yield (72.8 mg, 0.123 mmol). **^{1}H NMR** (400 MHz, $CDCl_3$) δ 7.41 (d, *J* = 2.2 Hz, 2H), 7.39 (d, *J* = 3.4 Hz, 2H), 7.35 (s, 1H), 7.33 (d, *J* = 6.5 Hz, 2H), 7.29 (dd, *J* = 6.3, 1.9 Hz, 4H), 7.23 (d, *J* = 7.2 Hz, 1H), 7.20 (d, *J* = 7.3 Hz, 1H), 6.65 (d, *J* = 7.4 Hz, 1H), 6.60 (s, 1H), 5.14 (s, 2H), 3.90 (t, *J* = 5.5 Hz, 2H), 2.30 (s, 3H), 2.16 (s, 3H), 1.76 (m, 4H), 1.39 (s, 12H), 1.28 (s, 6H). **^{13}C NMR** (101 MHz, $CDCl_3$) δ 177.8, 157.1, 142.8, 138.4, 137.7, 136.6, 135.8, 134.0, 130.4, 130.0, 128.6, 127.7, 127.6, 127.4, 126.7, 123.7, 120.8, 112.1, 83.8, 68.0, 66.0, 42.3, 37.3, 25.3, 25.0, 24.9, 21.5, 15.9, (*C*-B not observed). **^{11}B NMR** (128 MHz, $CDCl_3$) δ 29.2. HRMS calcd for $C_{38}H_{48}BO_5^+$ $[M+H]^+$ 595.3595 m/z, found 595.3584 m/z. Δ -1.8476.

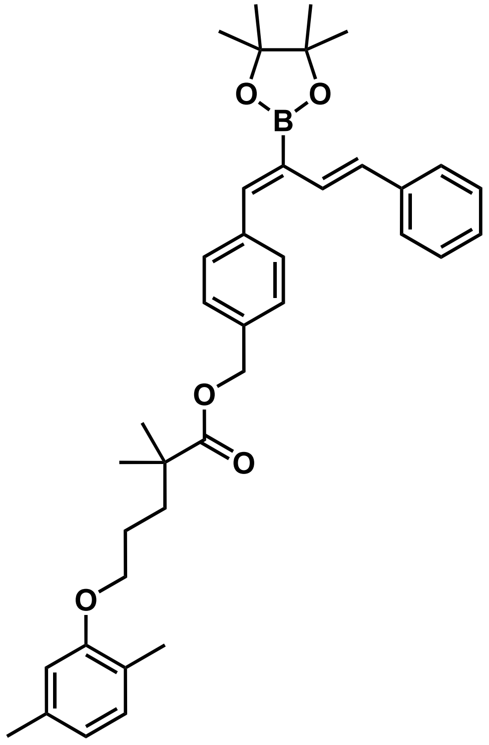

6. Experimental for Applications of 2-boryl-1,3-dienes

6.1 Synthesis of (1*Z*,3*Z*)-1,4-bis(4-methoxyphenyl)buta-1,3-diene

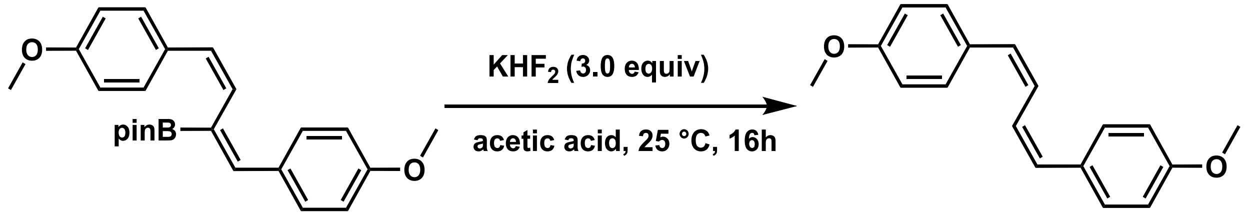

2f

3

Scheme S11: Protodeboration of 2-boryl-1,3-diene **3**.

To a two-dram vial: a stir bar, 2-((1*Z*,3*Z*)-1,4-bis(4-methoxyphenyl)buta-1,3-dien-2-yl)-4,4,5,5-tetramethyl-1,3,2-dioxaborolane, **2f** (0.125 mmol, 1.0 equiv, 49.0 mg), KHF_2 (0.375 mmol, 3.0 equiv, 29.3 mg) and 0.50 mL of acetic acids were added. The reaction was allowed to stir overnight. The reaction was quenched with sodium carbonate and extracted with ethyl acetate (5.0 mL x 3). The organic layer was reduced under vacuum and compound (1*Z*,3*Z*)-1,4-bis(4-methoxyphenyl)buta-1,3-diene **3** was isolated via flash chromatography (0-10% EtOAc:Hex) as a white solid in 54% yield (18.0 mg).

^{1}H NMR (400 MHz, $CDCl_3$) δ 7.37 (dd, *J* = 8.8, 2.1 Hz, 4H), 6.90 (dd, *J* = 8.7, 2.3 Hz, 4H), 6.62 (ddd, *J* = 10.5, 1.7, 0.59 Hz, 2H), 6.49 (ddd, *J* = 10.1, 8.0, 2.0 Hz, 2H), 3.84 (s, 6H). **^{13}C NMR** (101 MHz, $CDCl_3$) δ 158.9, 131.1, 130.6, 125.4, 122.3, 113.8, 55.5. HRMS calcd for $C_{18}H_{19}O_2^+$ $[M+H]^+$ 267.1385 m/z, found 267.1377 m/z. Δ -2.9947.

6.2 Synthesis of (*E*)-1,4-bis(4-methoxyphenyl)but-3-en-2-one

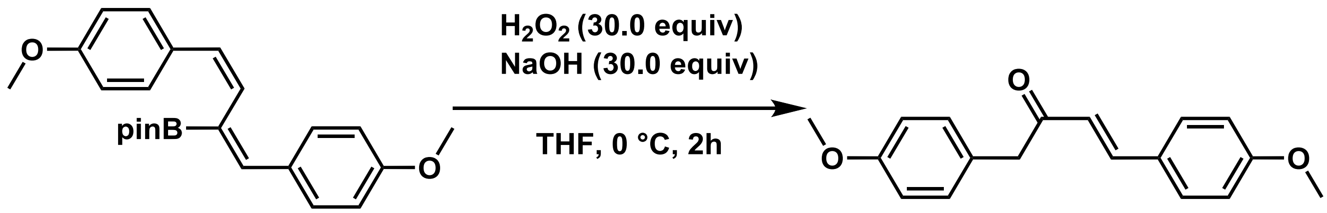

2f
>99:1 (*Z:E*)

5
1:>99 (*Z:E*)

Scheme S12: Basic peroxide oxidation of 2-boryl-1,3-diene **5**.

To a two-dram vial: a stir bar, 2-((1*Z*,3*Z*)-1,4-bis(4-methoxyphenyl)buta-1,3-dien-2-yl)-4,4,5,5-tetramethyl-1,3,2-dioxaborolane **2f** (0.125 mmol, 1.0 equiv, 49.0 mg) and THF (0.5 mL) were added. The reaction vial was put into an ice bath and allowed to cool to 0 °C. Then, 35 wt% hydrogen peroxide (3.75 mmol, 30.0 equiv, 0.25 mL) and 3M NaOH (3.75 mmol, 30.0 equiv, 1.25 mL) were added. The reaction was allowed to stir for 2 hours. Upon completion the reaction was quenched with saturated hydrogen sulfide (5.0 mL x 3) and extracted with ethyl acetate (5.0 mL x 3). The organic layer was reduced under vacuum and compound (*E*)-1,4-bis(4-methoxyphenyl)but-3-en-2-one, **5** was isolated via flash chromatography (0-30% EtOAc:Hex) as a white solid in 54% yield (15.0 mg).

^{1}H NMR (400 MHz, $CDCl_3$) δ 7.59 (d, *J* = 16.1 Hz, 1H), 7.47 (dd, *J* = 8.8, 1.3 Hz, 2H), 7.18 (d, *J* = 8.6 Hz, 2H), 6.89 (dd, *J* = 8.8, 5.4 Hz, 4H), 6.66 (d, *J* = 16.0 Hz, 1H), 3.85 (s, 2H), 3.84 (s, 3H), 3.80 (s, 3H). **^{13}C NMR** (126 MHz, $CDCl_3$) δ 197.8, 161.8, 158.7, 143.2, 130.6, 130.2, 127.3, 126.8, 123.1, 114.5, 114.3, 55.5, 55.4, 47.6. HRMS calcd for $C_{18}H_{19}O_3^+$ $[M+H]^+$ 283.1334 m/z, found 283.1332 m/z. Δ -0.7064.

6.3 Synthesis of (*Z*)-2-((*E*)-4-methoxybenzylidene)-4-(4-methoxyphenyl)but-3-en-1-ol

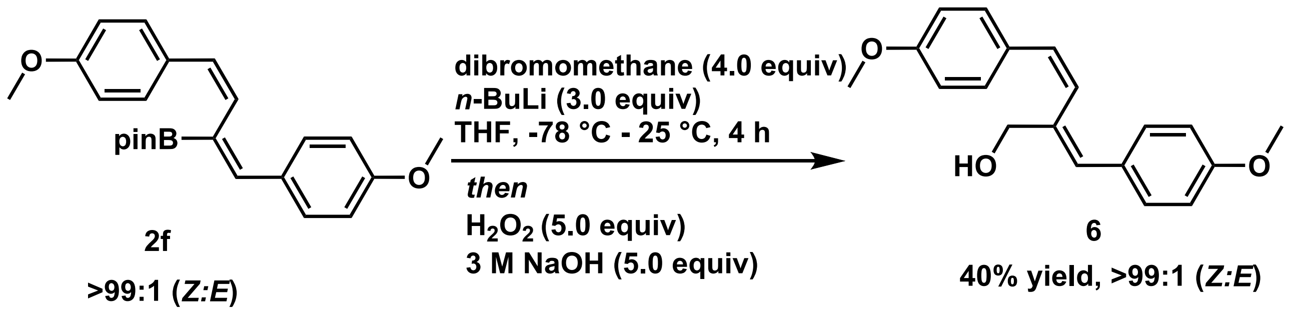

Scheme S13: Homologation-oxidation of 2-boryl-1,3-diene **6**.

This procedure was modified from literature. To a flame dried two-dram vial: a stir bar, dibromomethane (0.50 mmol, 4.0 equiv, 86.9 mg) and anhydrous THF (1 mL) were added. The vial was put under inert atmosphere using standard *Schlenk* technique. The reaction was put into a dry ice/acetone bath and allowed to cool to -78 °C. Then, 2.5 M *n*-BuLi in hexanes (0.375 mmol, 3.0 equiv, 150.0 µL) was added and the reaction was allowed to stir for 30 minutes. Then, 2-((1*Z*,3*Z*)-1,4-bis(4-methoxyphenyl)buta-1,3-dien-2-yl)-4,4,5,5-tetramethyl-1,3,2-dioxaborolane **2f** (0.125 mmol, 1.0 equiv, 49.0 mg) was added and the reaction was allowed to react for 1 hour. The reaction vessel was removed from the dry ice/acetone bath and allowed to stir at room temperature for 3 hours. After that, 35 wt% hydrogen peroxide (0.625 mmol, 5.0 equiv, 63.8 µL) and 3M NaOH (0.625 mmol, 5.0 equiv, 208.0 µL) were added and the reaction was allowed to stir at room temperature, overnight. Upon completion the reaction was quenched with saturated hydrogen sulfide (5.0 mL x 3) and extracted with ethyl acetate (5.0 mL x 3). The organic layer was reduced under vacuum and compound (*Z*)-2-((*E*)-4-methoxybenzylidene)-4-(4-methoxyphenyl)but-3-en-1-ol **6** was isolated via flash chromatography (0-25% EtOAc:Hex) as a yellow oil in 40% yield (14.8 mg).

^{1}H NMR (500 MHz, $CDCl_3$) δ 7.72 (d, *J* = 8.8 Hz, 2H), 7.12 (d, *J* = 8.6 Hz, 2H), 6.89 – 6.84 (m, 5H), 6.72 (d, *J* = 12.8 Hz, 1H), 6.13 (d, *J* = 12.8 Hz, 1H), 3.83 (s, 3H), 3.79 (s, 3H), 3.73 (s, 2H). **^{13}C NMR** (126 MHz, $CDCl_3$) δ 199.7, 161.2, 159.0, 142.1, 132.8, 130.9, 130.5, 128.1, 127.0, 124.9, 114.5, 113.9, 55.7, 55.6, 50.5. HRMS calcd for $C_{38}H_{41}O_6^+$ $[2M+H]^+$ 593.2898 m/z, found 593.2889 m/z. Δ -1.5170.

6.4 Synthesis of 4-((1*E*,3*Z*)-1,4-bis(4-methoxyphenyl)buta-1,3-dien-2-yl)benzonitrile

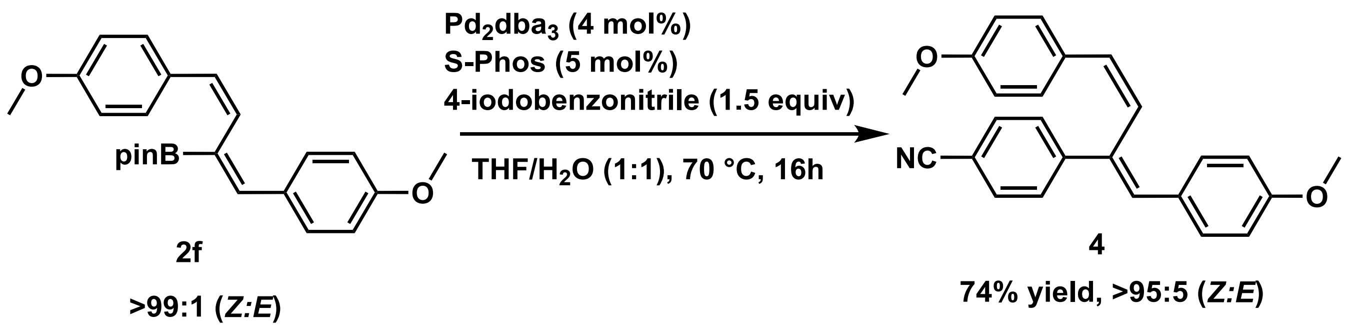

Scheme S14: Suzuki-Miyaura cross-coupling of 2-boryl-1,3-diene **2f** and 4-iodobenzonitrile.

To a two-dram vial: a stir bar, 2-((1*Z*,3*Z*)-1,4-bis(4-methoxyphenyl)buta-1,3-dien-2-yl)-4,4,5,5-tetramethyl-1,3,2-dioxaborolane **2f** (0.125 mmol, 1.0 equiv, 49.0 mg), Pd_2dba_3 (5.28 μmol, 4 mol%, 2.17 mg), S-Phos (4.22 μmol, 5 mol%, 3.87 mg) and 4-iodobenzonitrile (0.158 mmol, 1.5 equiv, 36.3 mg) were added. The vial was sealed and put under inert atmosphere using standard *Schlenk* technique. Then THF : 3M NaOH (2.4 mL : 0.8 mL) were added and the reaction was heated to 60°C and allowed to react overnight. Ethyl acetate (5.0 mL x 3) was added to the mixture followed by extraction, treatment with sodium sulfate and concentration under vacuum. 4-((1*E*,3*Z*)-1,4-bis(4-methoxyphenyl)buta-1,3-dien-2-yl)benzonitrile **4** was isolated via flash chromatography (0-15% EtOAc:Hex) as a yellow solid in 74% yield (28.8 mg).

^{1}H NMR (500 MHz, $CDCl_3$) δ 7.56 (d, *J* = 8.8 Hz, 2H), 7.49 (d, *J* = 8.0 Hz, 2H), 7.40 (d, *J* = 8.2 Hz, 2H), 7.08 (d, *J* = 8.7 Hz, 2H), 6.90 (d, *J* = 8.8 Hz, 2H), 6.86 (s, 1H), 6.75 (d, *J* = 12.0 Hz, 1H), 6.58 (d, *J* = 8.8 Hz, 2H), 6.49 (dd, *J* = 11.9, 1.9 Hz, 1H), 3.84 (s, 3H), 3.70 (s, 3H). **^{13}C NMR** (126 MHz, $CDCl_3$) δ 159.6, 159.0, 146.3, 135.6, 133.6, 133.1, 131.9, 131.3, 130.0, 129.4, 127.8, 126.0, 119.3, 113.9, 113.3, 110.0, 103.5, 55.5, 55.3. HRMS calcd for $C_{50}H_{42}N_2NaO_4^+$ $[2M+Na]^+$ 757.3037 m/z, found 757.3039 m/z. Δ 0.2641.

7. Hidden Borane Catalysis Experiment

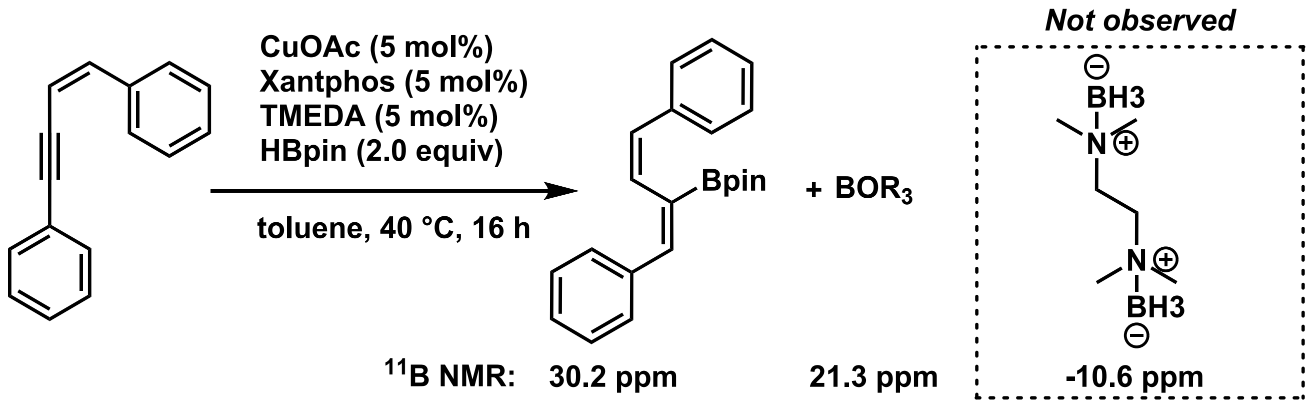

Scheme S15: Hidden borane catalysis experiments.

To a flame dried 2-dram vial: a stir bar, (*Z*)-but-1-en-3-yne-1,4-diyldibenzene (1.0 equiv, 0.125 mmol, 25.5 mg), copper acetate (5 mol%, 0.006 mmol, 0.77 mg), Xantphos (5 mol%, 0.006 mmol, 3.62 mg) and TMEDA (5 mol%, 0.006 mmol, 0.726 mg) were added. The vial was put under inert atmosphere using standard *Schlenk* technique. Then toluene (0.5 mL) was added, and the reaction was allowed to stir until the mixture turned green. The temperature was set to 40 °C. Pinacolborane (2.0 equiv, 0.250 mmol, 32.0 mg) was then added and the reaction was allowed to stir overnight. Next, the reaction vial was opened to air and the mixture was transferred to an NMR tube. A ^{11}B NMR spectra was collected. No borane-TMEDA adduct was observed.

8. Crystallographic Data

A large colorless prism was cut (0.24 × 0.38 × 0.45 mm^3) and centered on the goniometer of a Rigaku Oxford Diffraction Synergy-S diffractometer equipped with a HyPix6000HE detector and operating with MoKα radiation. The data collection routine, unit cell refinement, and data processing were carried out with the program CrysAlisPro.[1] The Laue symmetry and systematic absences were consistent with the monoclinic space group $P2_1/n$. The structure was solved using SHELXT[2] and refined using SHELXL[3] via Olex2.[4] The final refinement model involved anisotropic displacement parameters for non-hydrogen atoms and a riding model for all hydrogen atoms. Mercury[5] was used for molecular graphics generation.

Crystal Growth Procedure: Compound **2b** (yellow oil) was left in the freezer (0 °C) for approximately 3 months. During which time large colorless crystals formed which were sufficient for X-ray crystallography.

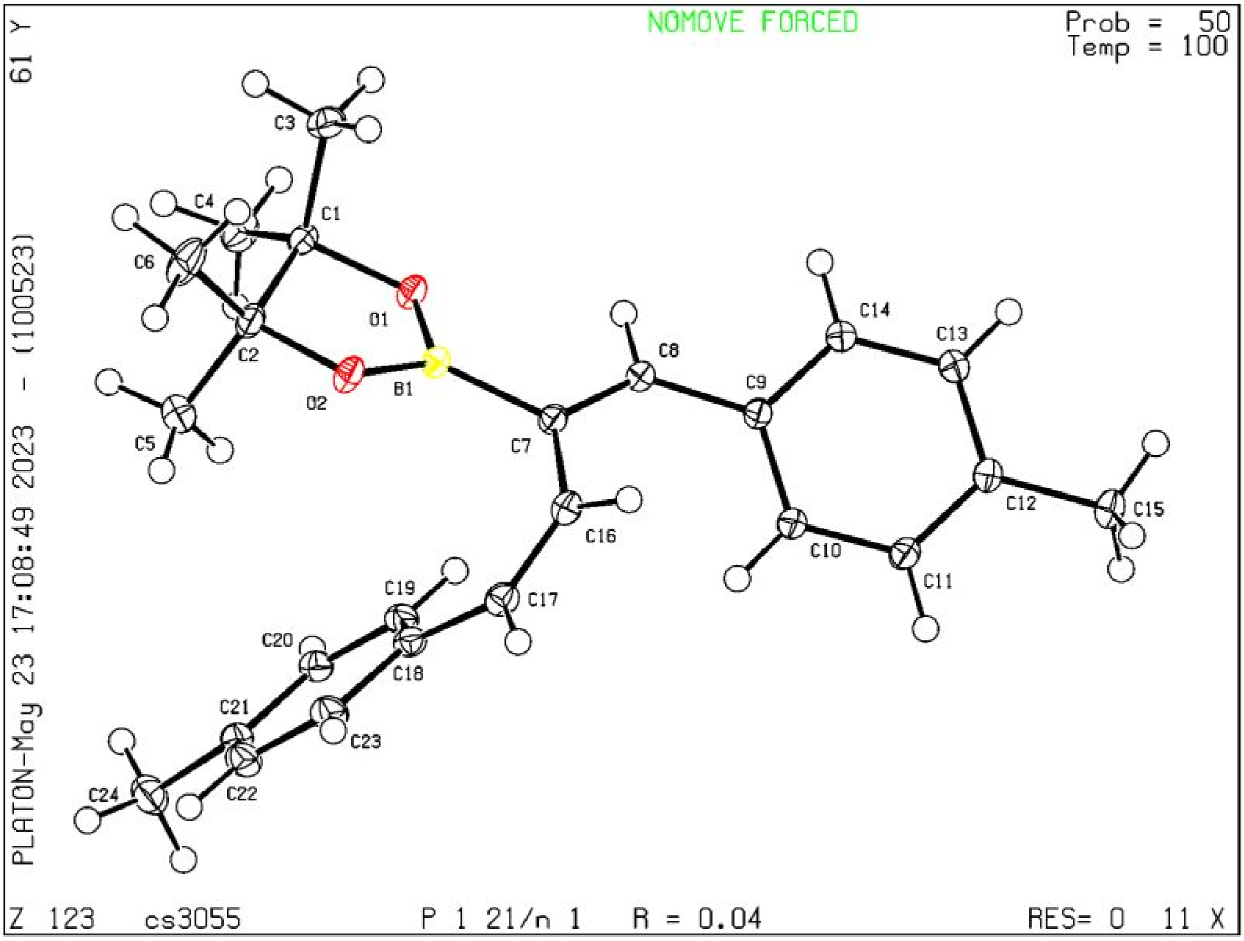

Figure S1: ORTEP diagram of **2b** with thermal ellipsoids at 50% probability level.

Table S2: Crystal data and structure refinement for cs3055 (2b).

Identification code	cs3055
Empirical formula	$C_{24}H_{29}BO_2$
Formula weight	360.28
Temperature/K	100
Crystal system	monoclinic
Space group	$P2_1/n$
a/Å	10.3788(2)
b/Å	16.4472(2)
c/Å	12.6082(2)
α/°	90
β/°	108.355(2)
γ/°	90
Volume/Å^3	2042.75(6)
Z	4
ρ_{calc}g/cm^3	1.171
μ/mm^{-1}	0.072
F(000)	776.0
Crystal size/mm^3	0.45 × 0.38 × 0.24
Radiation	Mo Kα (λ = 0.71073)
2Θ range for data collection/°	6.454 to 76.528
Index ranges	-17 ≤ h ≤ 17, -28 ≤ k ≤ 28, -21 ≤ l ≤ 21
Reflections collected	93612
Independent reflections	10844 [R_{int} = 0.0379, R_{sigma} = 0.0214]
Data/restraints/parameters	10844/0/250
Goodness-of-fit on F^2	1.047
Final R indexes [I>=2σ (I)]	R_1 = 0.0408, wR_2 = 0.1171
Final R indexes [all data]	R_1 = 0.0498, wR_2 = 0.1220
Largest diff. peak/hole / e Å^{-3}	0.58/-0.32

Crystal data as a footnote:

Crystal Data for cs3055 (2b). $C_{24}H_{29}BO_2$ (*M* =360.28 g/mol): monoclinic, space group $P2_1/n$ (no. 14), *a* = 10.3788(2) Å, *b* = 16.4472(2) Å, *c* = 12.6082(2) Å, *β* = 108.355(2)°, *V* = 2042.75(6) Å^3, *Z* = 4, *T* = 100 K, μ(Mo Kα) = 0.072 mm^{-1}, *Dcalc* = 1.171 g/cm^3, 93612 reflections measured (6.454° ≤ 2Θ ≤ 76.528°), 10844 unique (R_{int} = 0.0379, R_{sigma} = 0.0214) which were used in all calculations. The final R_1 was 0.0408 (I > 2σ(I)) and wR_2 was 0.1220 (all data).

Table S3 Bond Lengths for cs3055 (2b)

Atom	Atom	Length/Å	Atom	Atom	Length/Å
B1	O1	1.3723(7)	C10	C11	1.3913(8)
B1	O2	1.3651(7)	C11	C12	1.3980(8)
B1	C7	1.5616(8)	C12	C13	1.3937(8)
O1	C1	1.4617(7)	C12	C15	1.5073(8)
O2	C2	1.4602(7)	C13	C14	1.3918(8)
C1	C2	1.5666(8)	C16	C17	1.3470(8)
C1	C3	1.5224(8)	C17	C18	1.4677(8)
C1	C4	1.5202(8)	C18	C19	1.4025(8)
C2	C5	1.5225(9)	C18	C23	1.4030(8)
C2	C6	1.5170(9)	C19	C20	1.3898(8)
C7	C8	1.3590(8)	C20	C21	1.3977(8)
C7	C16	1.4772(8)	C21	C22	1.3960(9)
C8	C9	1.4667(7)	C21	C24	1.5044(9)
C9	C10	1.4025(8)	C22	C23	1.3924(9)
C9	C14	1.4012(8)			

Table S4 Bond Angles for cs3055 (2b)

Atom	Atom	Atom	Angle/˚	Atom	Atom	Atom	Angle/˚
O1	B1	C7	122.95(5)	C14	C9	C8	118.65(5)
O2	B1	O1	113.34(5)	C14	C9	C10	117.53(5)
O2	B1	C7	123.68(5)	C11	C10	C9	120.88(5)
B1	O1	C1	107.55(4)	C10	C11	C12	121.37(5)
B1	O2	C2	107.79(4)	C11	C12	C15	120.72(5)
O1	C1	C2	102.78(4)	C13	C12	C11	117.79(5)
O1	C1	C3	106.88(5)	C13	C12	C15	121.48(5)
O1	C1	C4	107.94(5)	C14	C13	C12	121.11(5)
C3	C1	C2	112.85(5)	C13	C14	C9	121.24(5)
C4	C1	C2	115.35(5)	C17	C16	C7	128.04(5)
C4	C1	C3	110.32(5)	C16	C17	C18	128.19(5)
O2	C2	C1	102.70(4)	C19	C18	C17	122.95(5)
O2	C2	C5	107.18(5)	C19	C18	C23	117.56(5)
O2	C2	C6	108.09(5)	C23	C18	C17	119.40(5)
C5	C2	C1	113.71(5)	C20	C19	C18	121.08(5)
C6	C2	C1	114.33(5)	C19	C20	C21	121.15(5)
C6	C2	C5	110.17(6)	C20	C21	C24	120.70(6)
C8	C7	B1	117.46(5)	C22	C21	C20	118.07(5)
C8	C7	C16	122.62(5)	C22	C21	C24	121.17(5)
C16	C7	B1	119.58(5)	C23	C22	C21	120.92(5)
C7	C8	C9	128.62(5)	C22	C23	C18	121.20(5)
C10	C9	C8	123.77(5)				

Table S5 Torsion Angles for cs3055 (2b).

A	B	C	D	Angle/˚	A	B	C	D	Angle/˚
B1	O1	C1	C2	-19.47(6)	C7	C8	C9	C14	-156.71(6)
B1	O1	C1	C3	99.54(5)	C7	C16	C17	C18	8.44(10)
B1	O1	C1	C4	-141.80(5)	C8	C7	C16	C17	-137.59(6)
B1	O2	C2	C1	-20.05(6)	C8	C9	C10	C11	179.79(5)
B1	O2	C2	C5	100.03(6)	C8	C9	C14	C13	179.09(5)
B1	O2	C2	C6	-141.24(6)	C9	C10	C11	C12	0.30(9)
B1	C7	C8	C9	-179.83(5)	C10	C9	C14	C13	-3.14(9)
B1	C7	C16	C17	49.24(8)	C10	C11	C12	C13	-1.80(9)
O1	B1	O2	C2	8.77(7)	C10	C11	C12	C15	179.46(6)
O1	B1	C7	C8	16.53(8)	C11	C12	C13	C14	0.81(9)
O1	B1	C7	C16	-169.95(5)	C12	C13	C14	C9	1.70(9)
O1	C1	C2	O2	23.70(5)	C14	C9	C10	C11	2.15(9)
O1	C1	C2	C5	-91.75(5)	C15	C12	C13	C14	179.54(5)
O1	C1	C2	C6	140.52(5)	C16	C7	C8	C9	6.86(9)
O2	B1	O1	C1	7.79(6)	C16	C17	C18	C19	24.64(9)
O2	B1	C7	C8	-161.53(5)	C16	C17	C18	C23	-158.85(6)
O2	B1	C7	C16	11.99(8)	C17	C18	C19	C20	176.44(5)
C3	C1	C2	O2	-91.04(5)	C17	C18	C23	C22	-176.98(6)
C3	C1	C2	C5	153.50(5)	C18	C19	C20	C21	-0.40(9)
C3	C1	C2	C6	25.78(7)	C19	C18	C23	C22	-0.28(9)
C4	C1	C2	O2	140.89(5)	C19	C20	C21	C22	1.32(9)
C4	C1	C2	C5	25.43(7)	C19	C20	C21	C24	-175.82(6)
C4	C1	C2	C6	-102.30(6)	C20	C21	C22	C23	-1.73(9)
C7	B1	O1	C1	-170.46(5)	C21	C22	C23	C18	1.24(10)
C7	B1	O2	C2	-173.00(5)	C23	C18	C19	C20	-0.13(8)
C7	C8	C9	C10	25.67(9)	C24	C21	C22	C23	175.39(6)

This report has been created with Olex2, compiled on 2023.03.06 svn.rbb2c1857 for OlexSys.

9. Failed Substrates

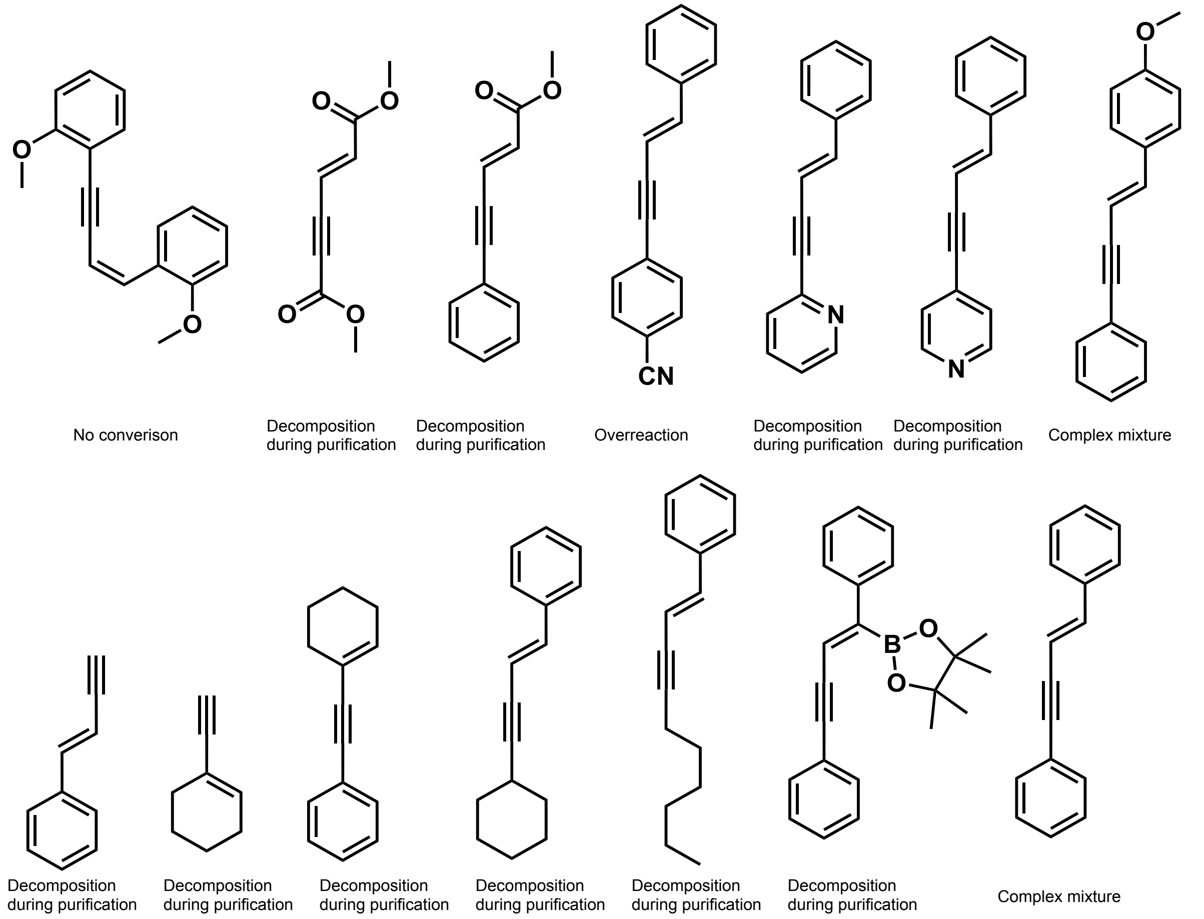

Figure S2: Substrates which failed to furnish 2-boryl-1,3-diene under the optimized conditions.

10. Spectra

^{1}H NMR of **2a** ($CDCl_3$, 400 MHz)

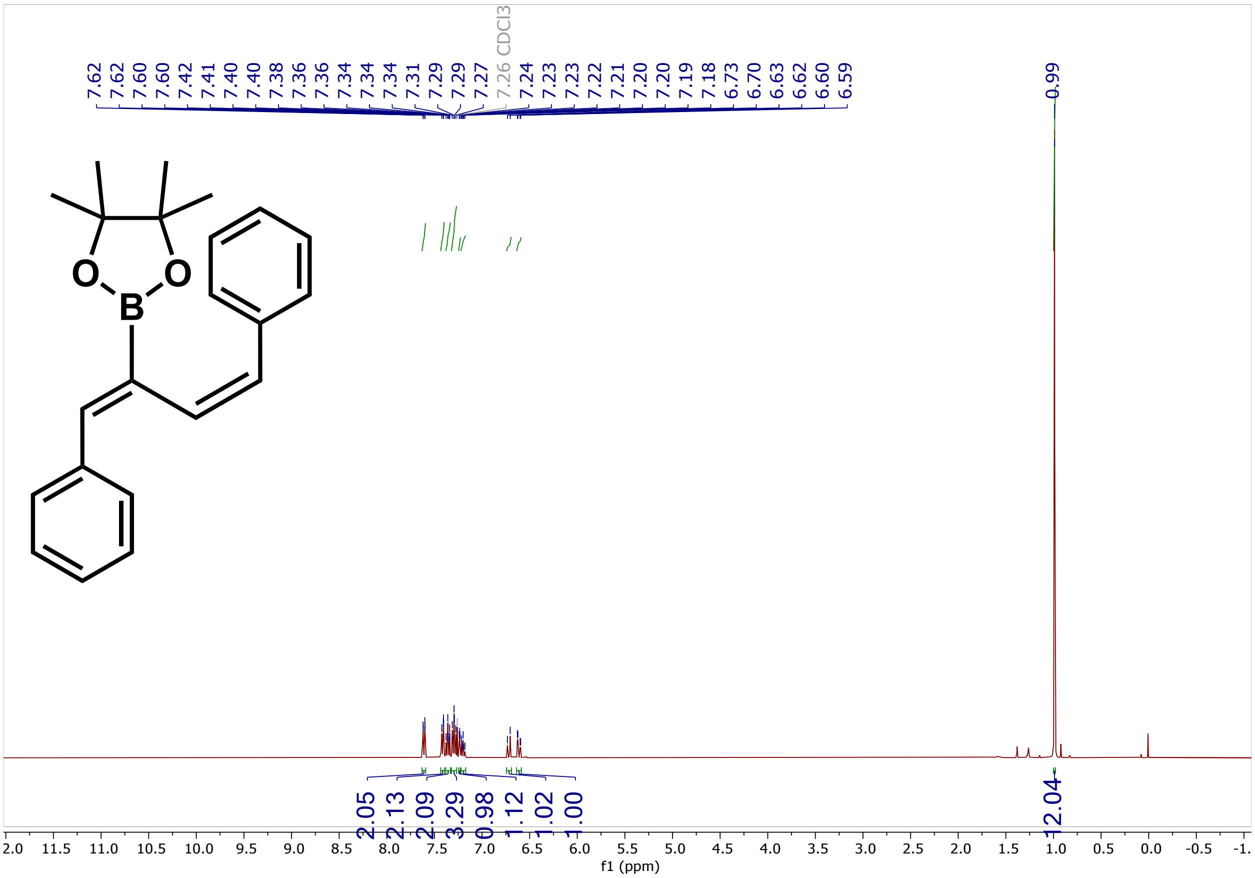

^{13}C NMR of **2a** ($CDCl_3$, 101 MHz)

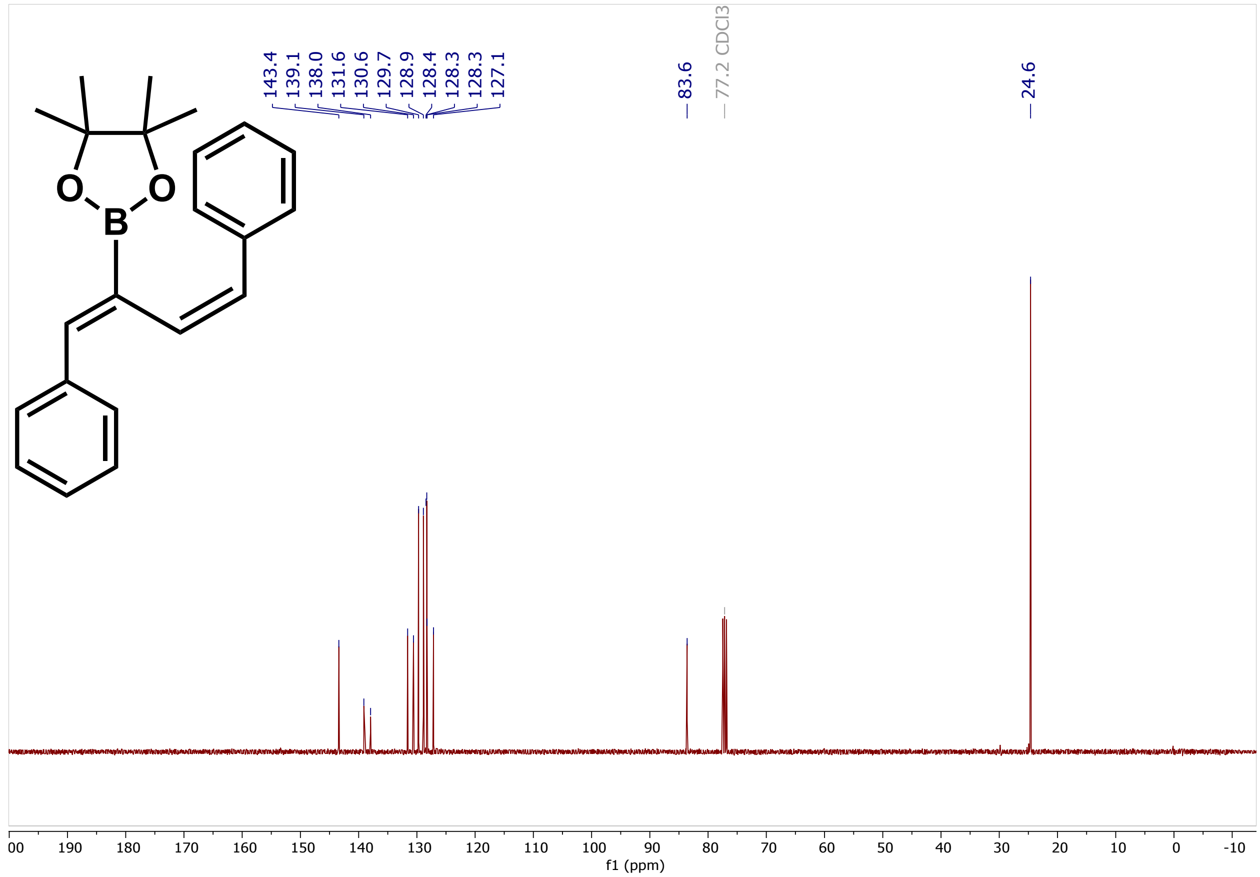

^{11}B NMR of **2a** ($CDCl_3$, 128 MHz)

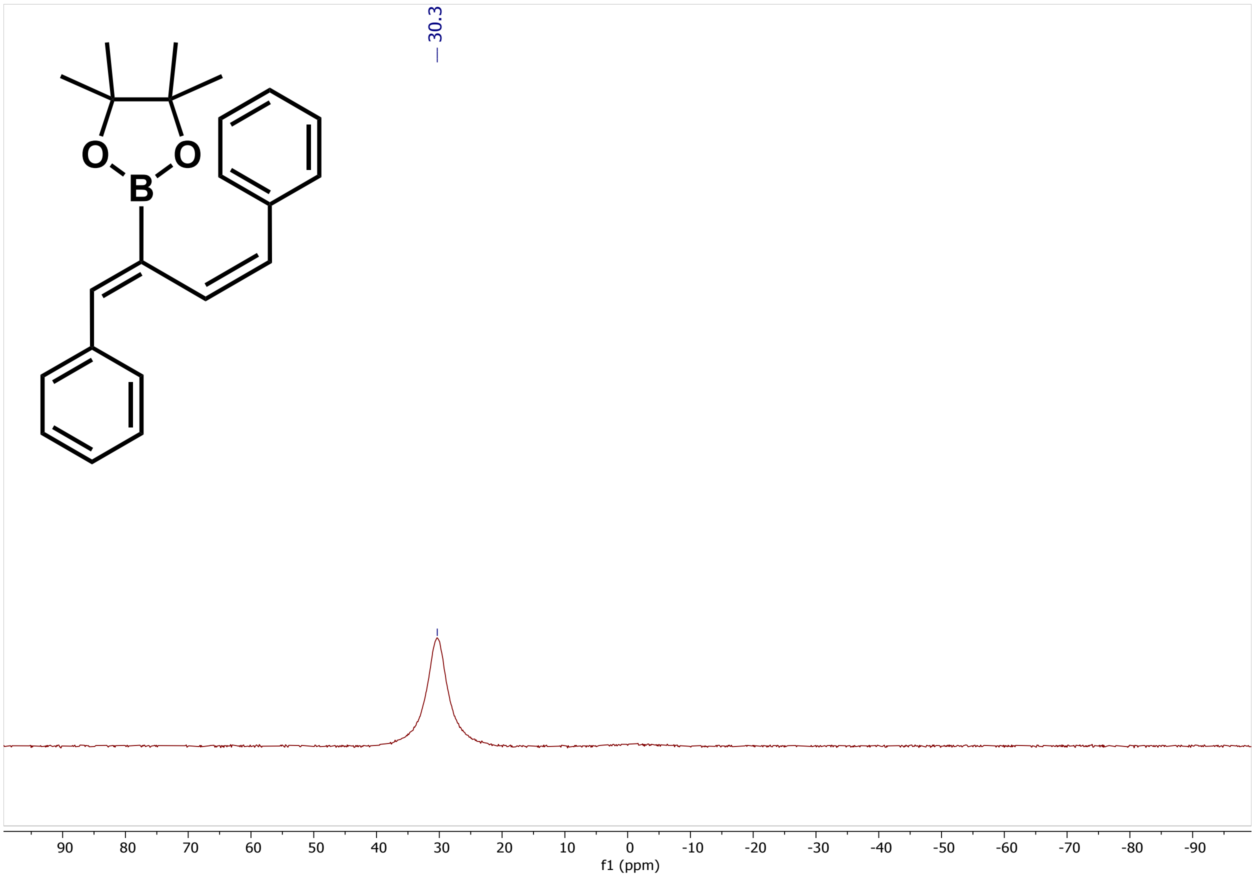

^{1}H NMR of **2b** ($CDCl_3$, 400 MHz)

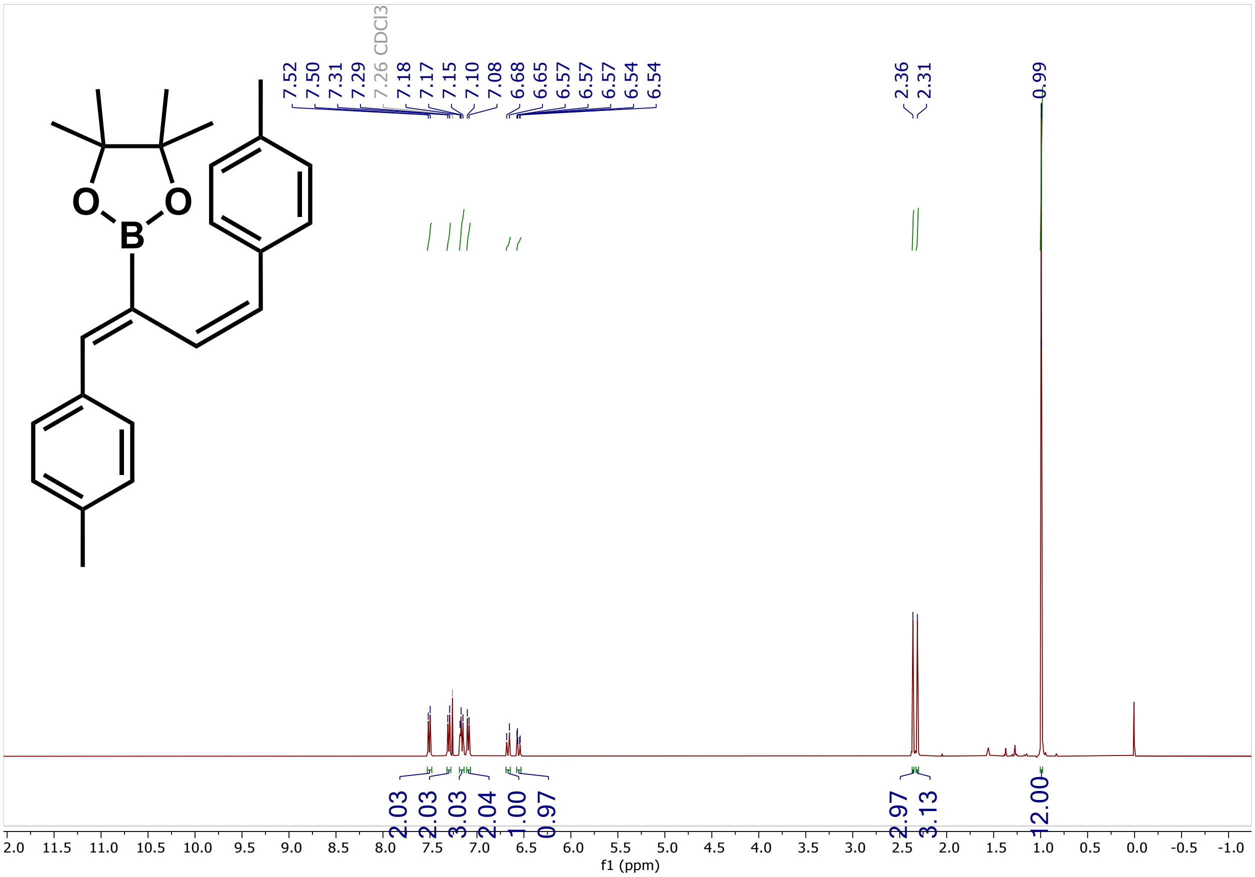

^{13}C NMR of **2b** ($CDCl_3$, 101 MHz)

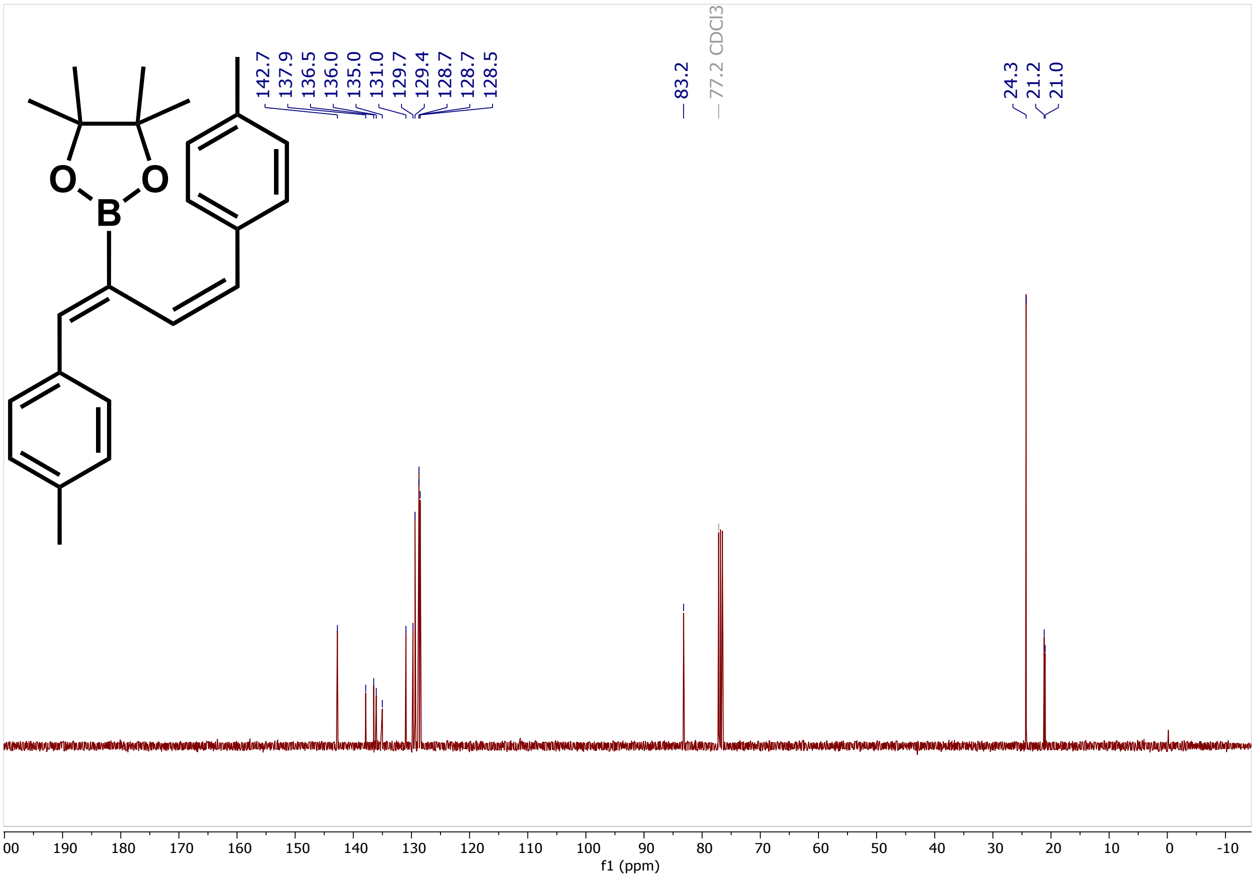

^{11}B NMR of **2b** ($CDCl_3$, 128 MHz)

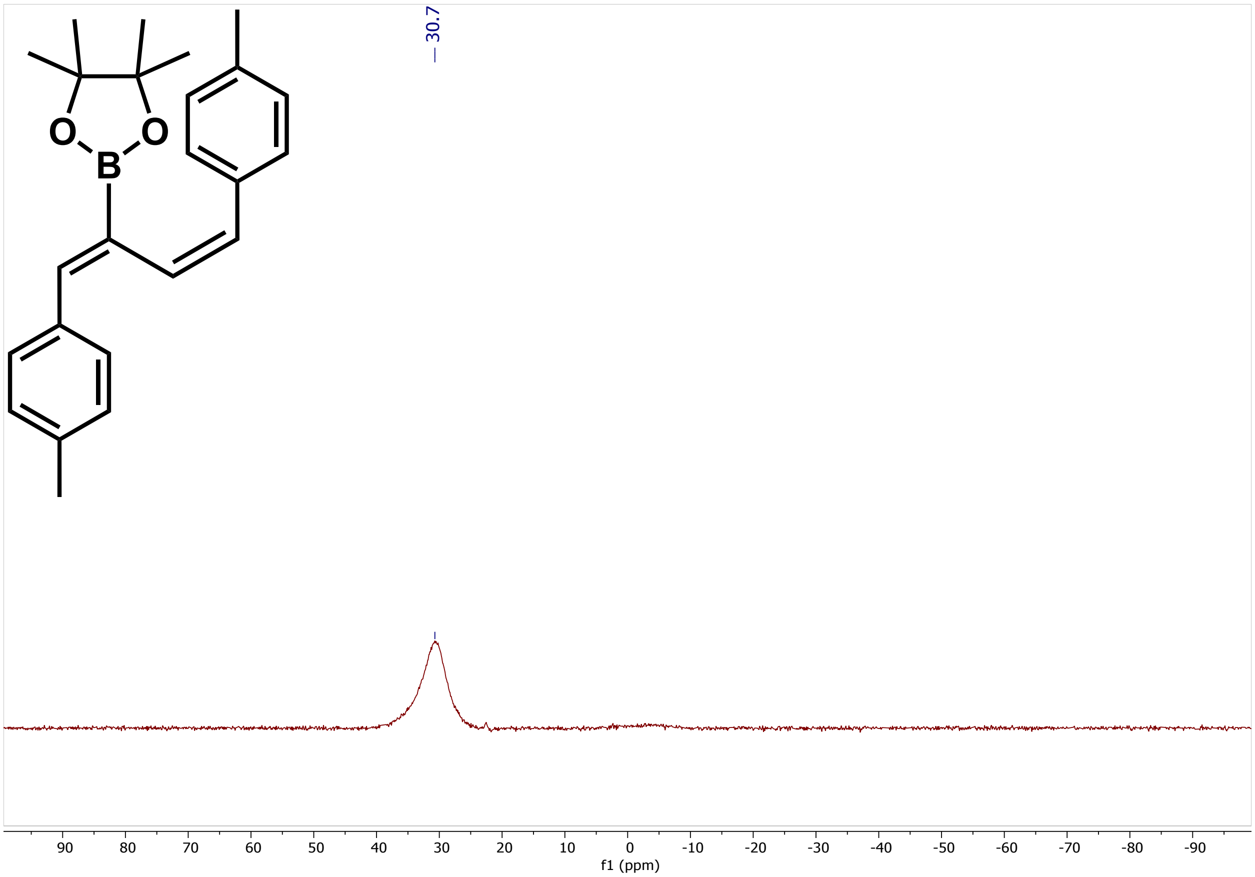

^{1}H NMR of **2c** ($CDCl_3$, 400 MHz)

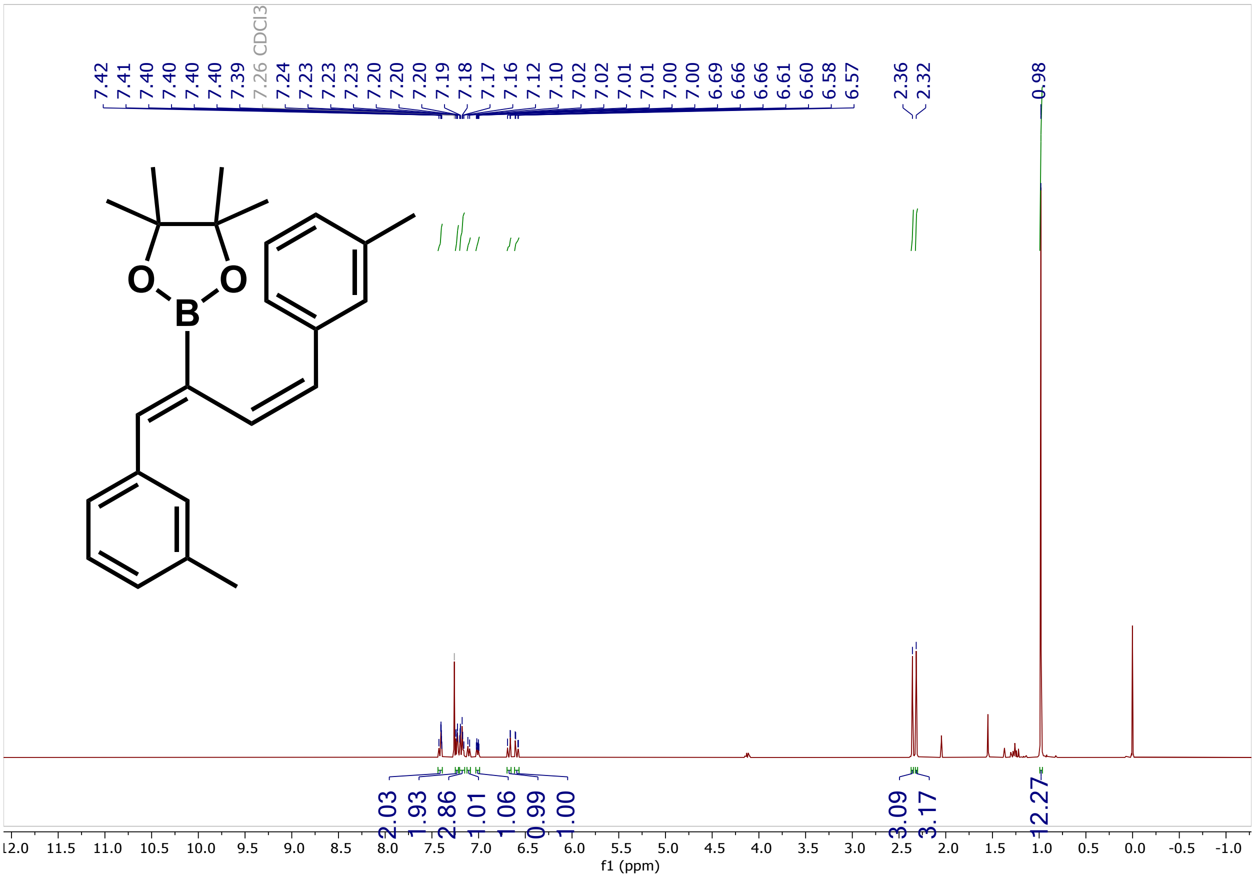

^{13}C NMR of **2c** ($CDCl_3$, 101 MHz)

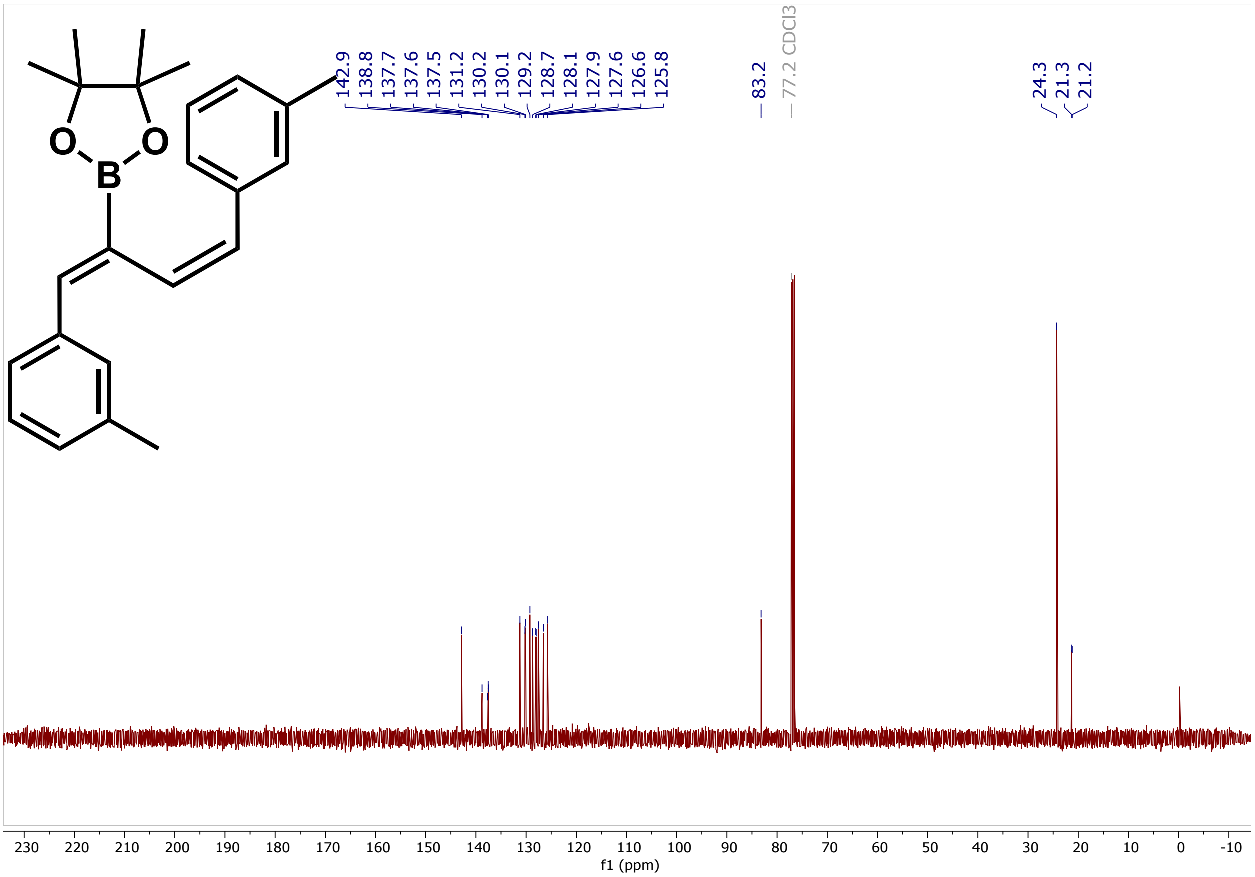

^{11}B NMR of **2c** ($CDCl_3$, 128 MHz)

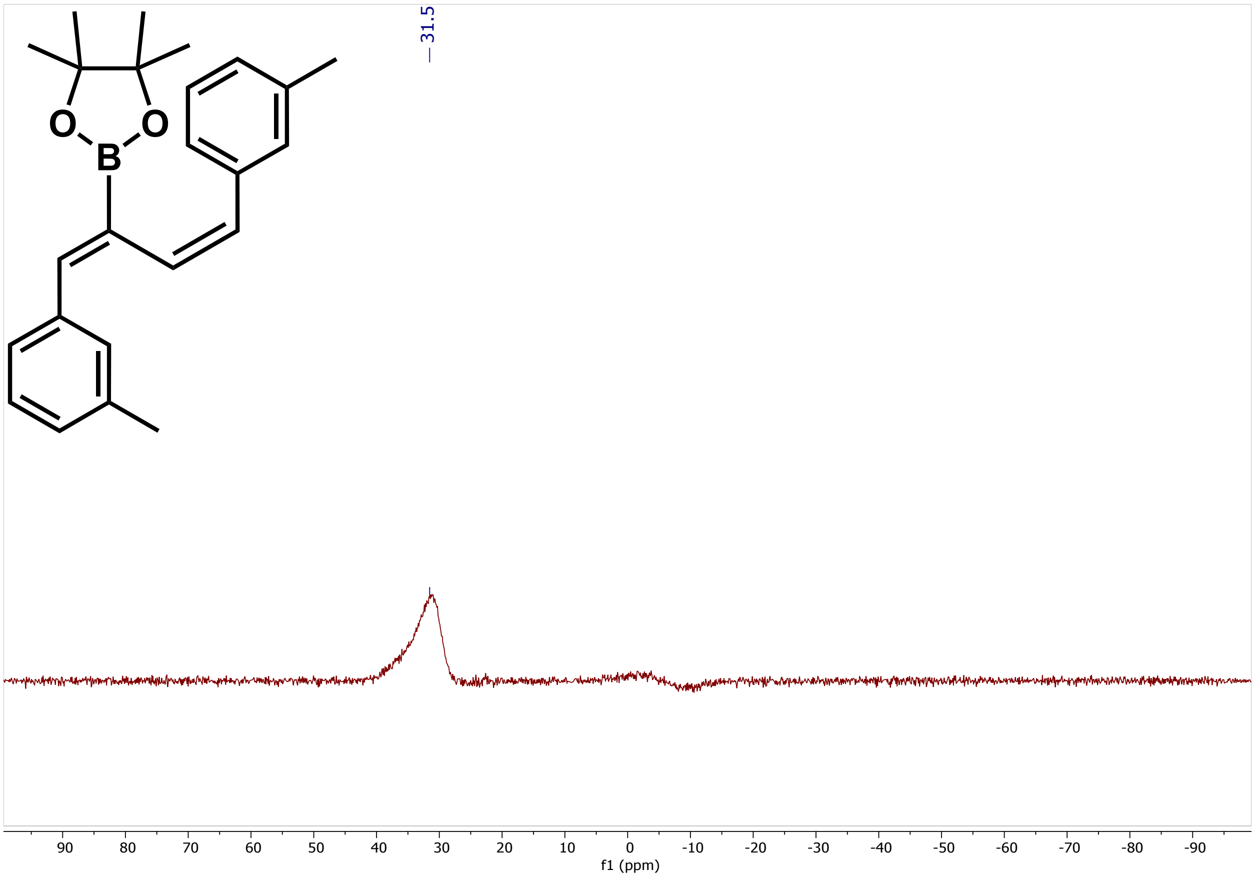

^{1}H NMR of **2d** ($CDCl_3$, 400 MHz)

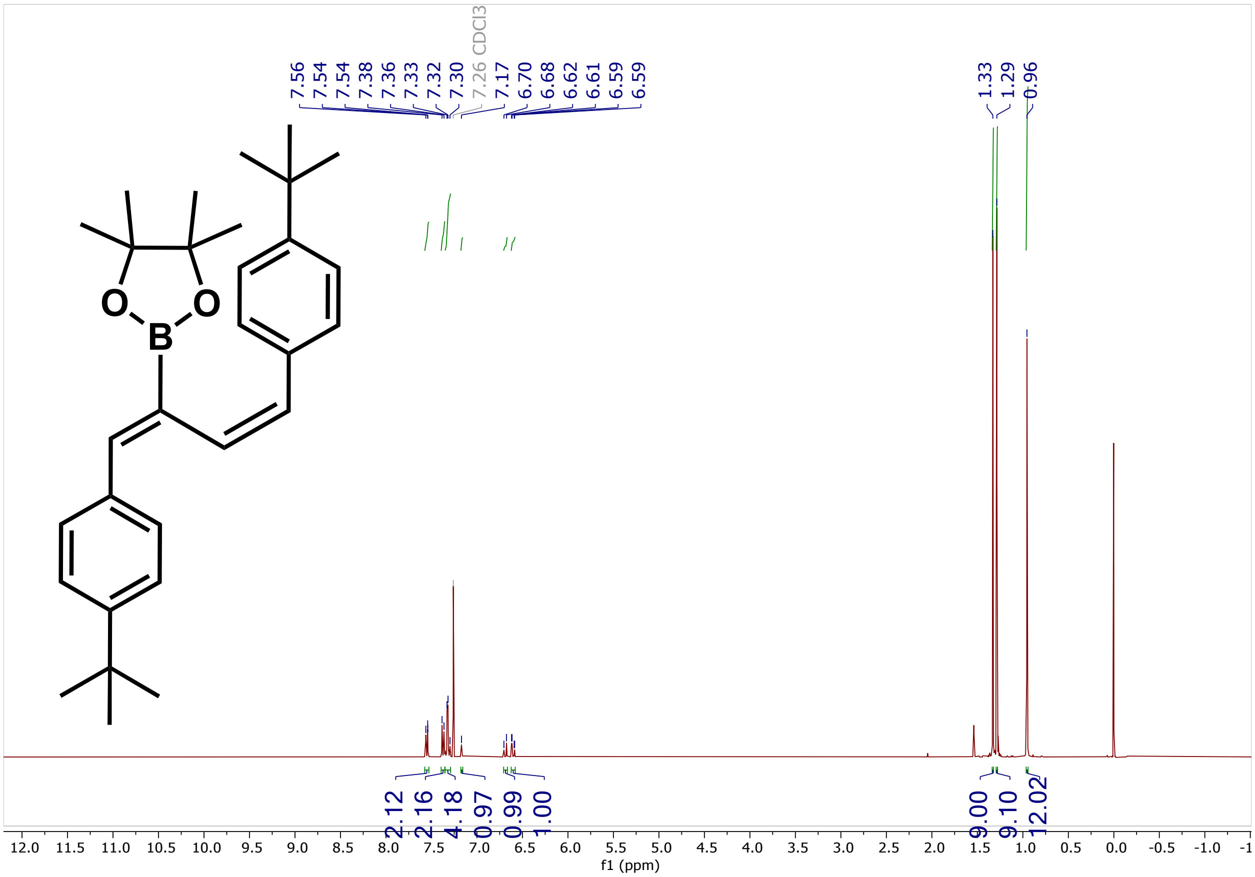

^{13}C NMR of **2d** ($CDCl_3$, 101 MHz)

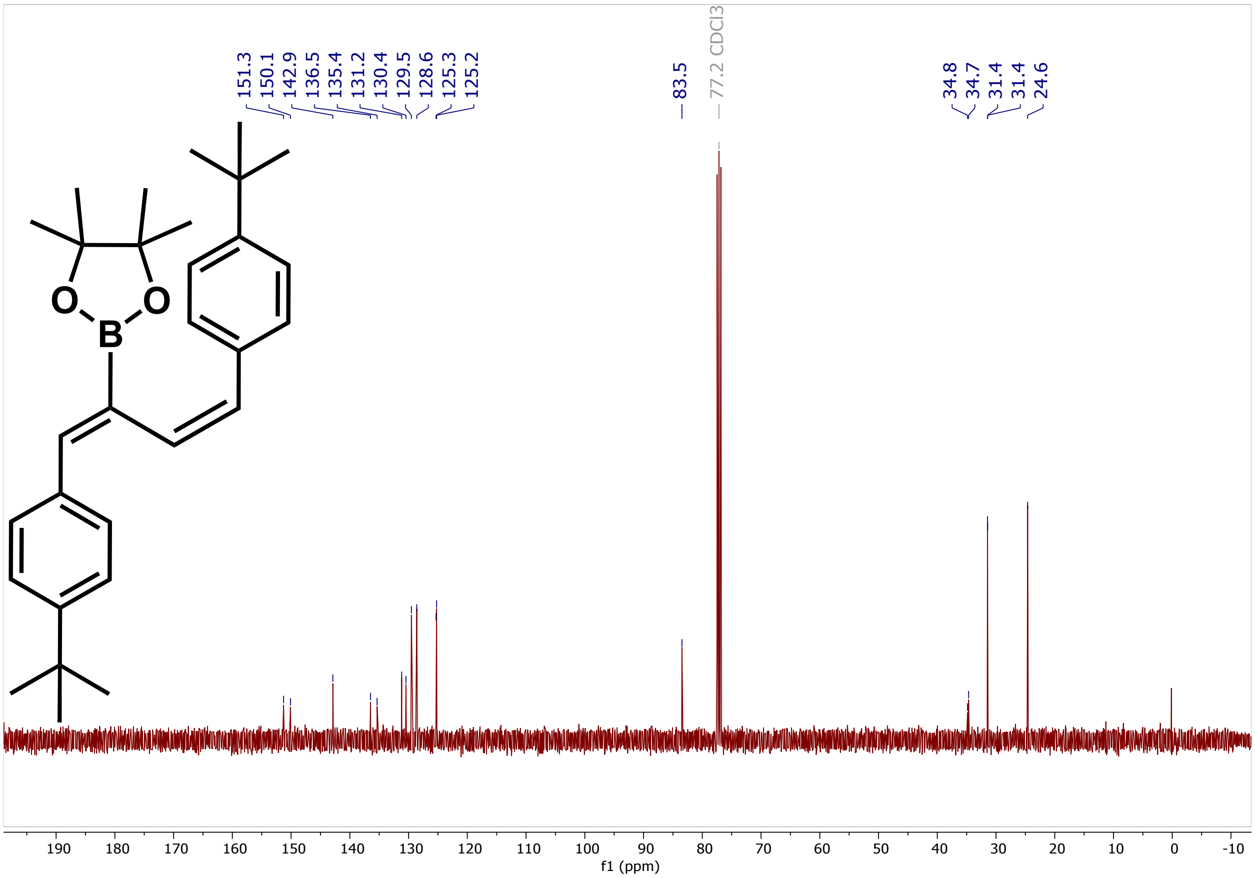

^{11}B NMR of **2d** ($CDCl_3$, 128 MHz)

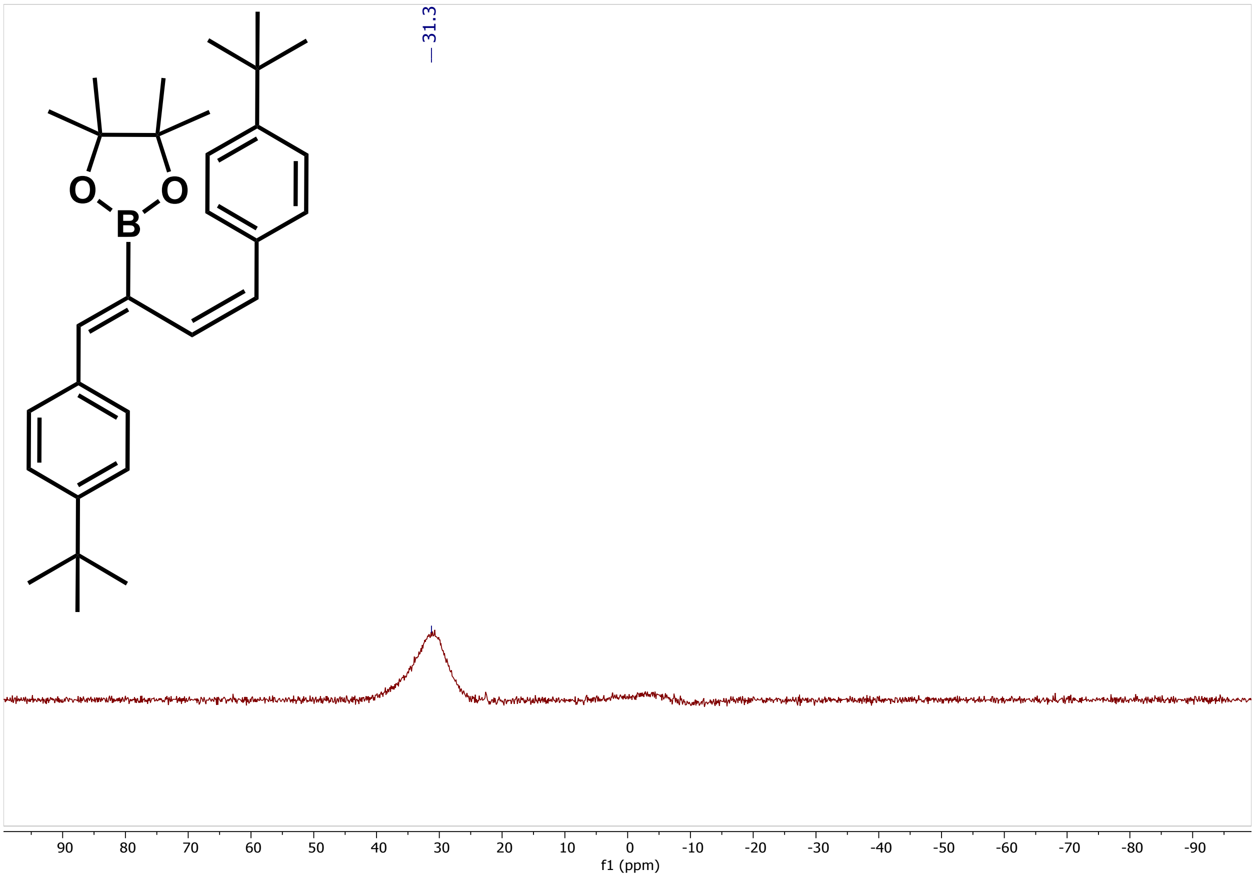

^{1}H NMR of **2e** ($CDCl_3$, 400 MHz)

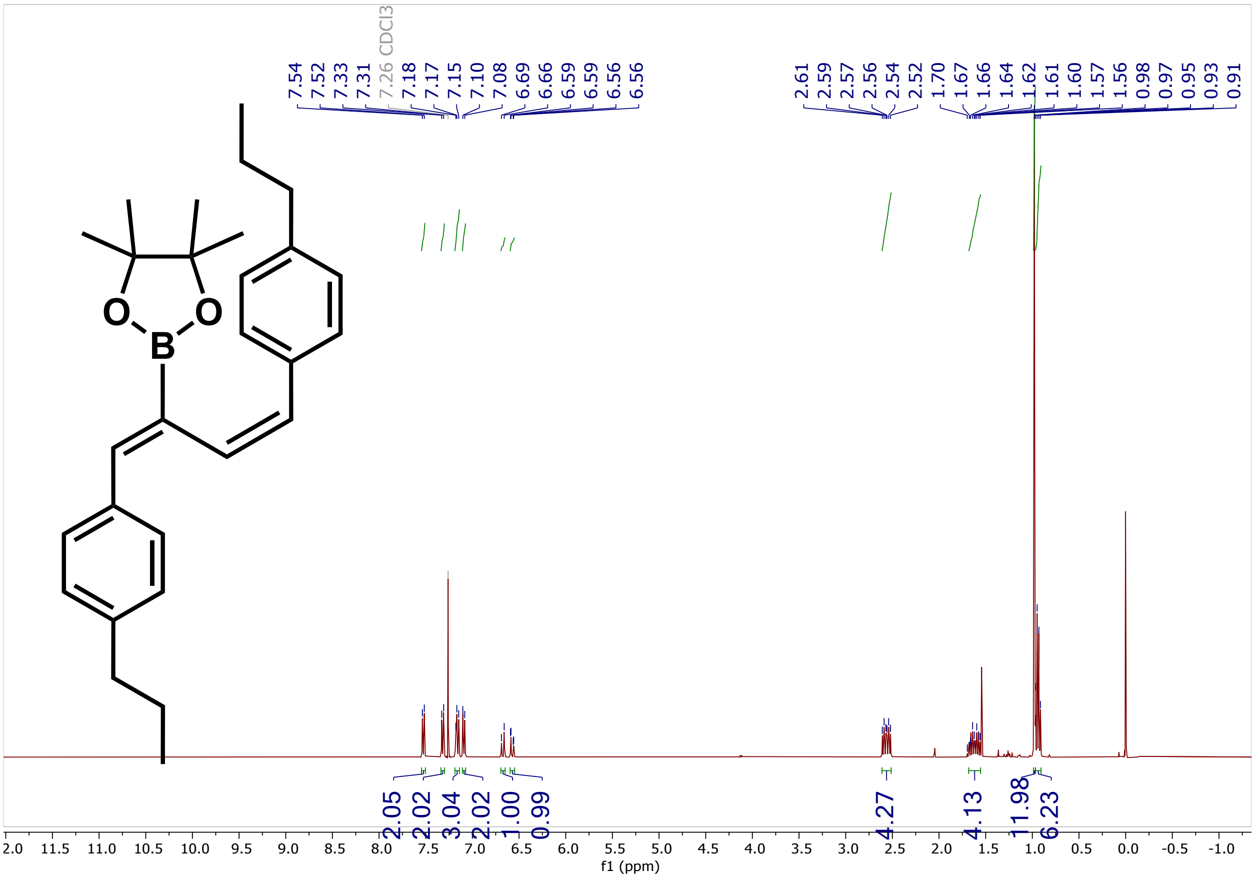

^{13}C NMR of **2e** ($CDCl_3$, 101 MHz)

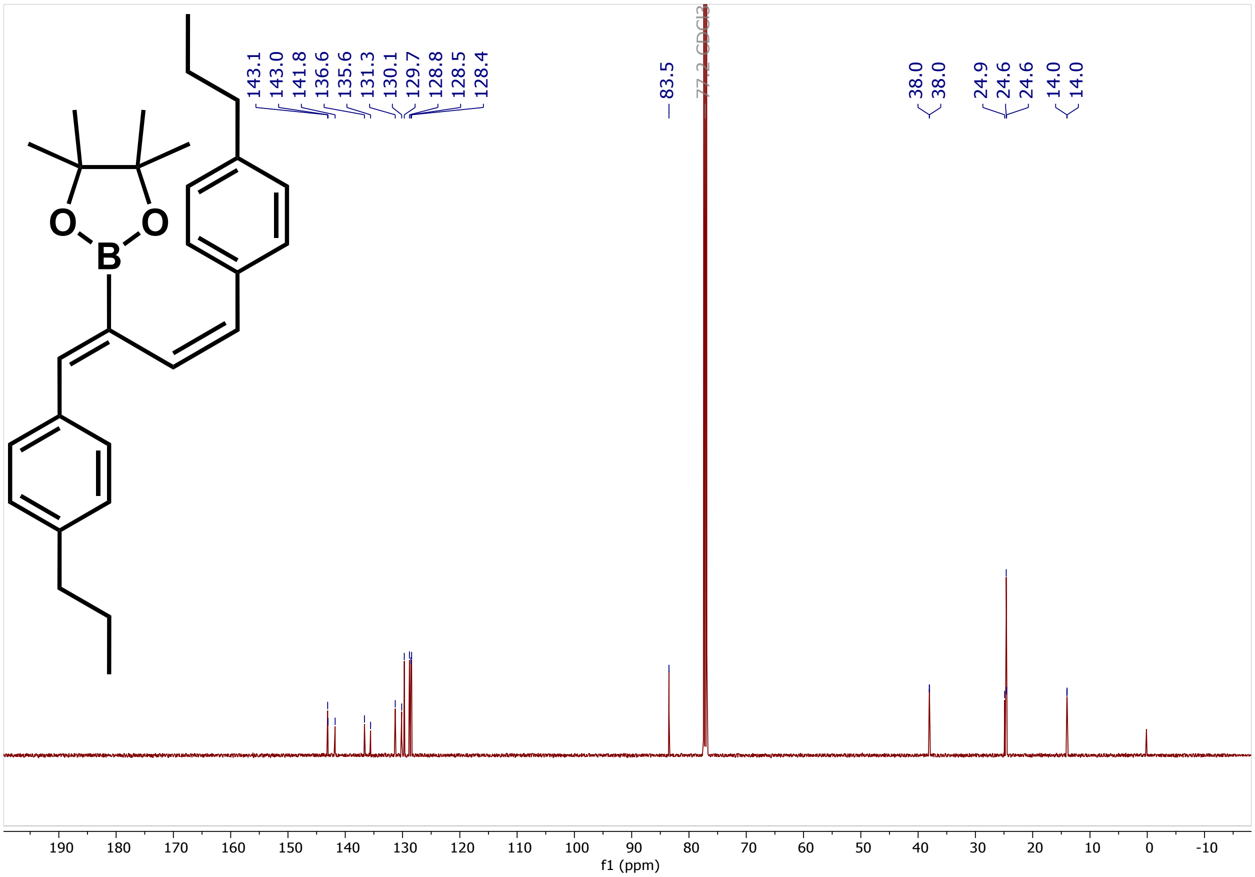

^{11}B NMR of **2e** ($CDCl_3$, 128 MHz)

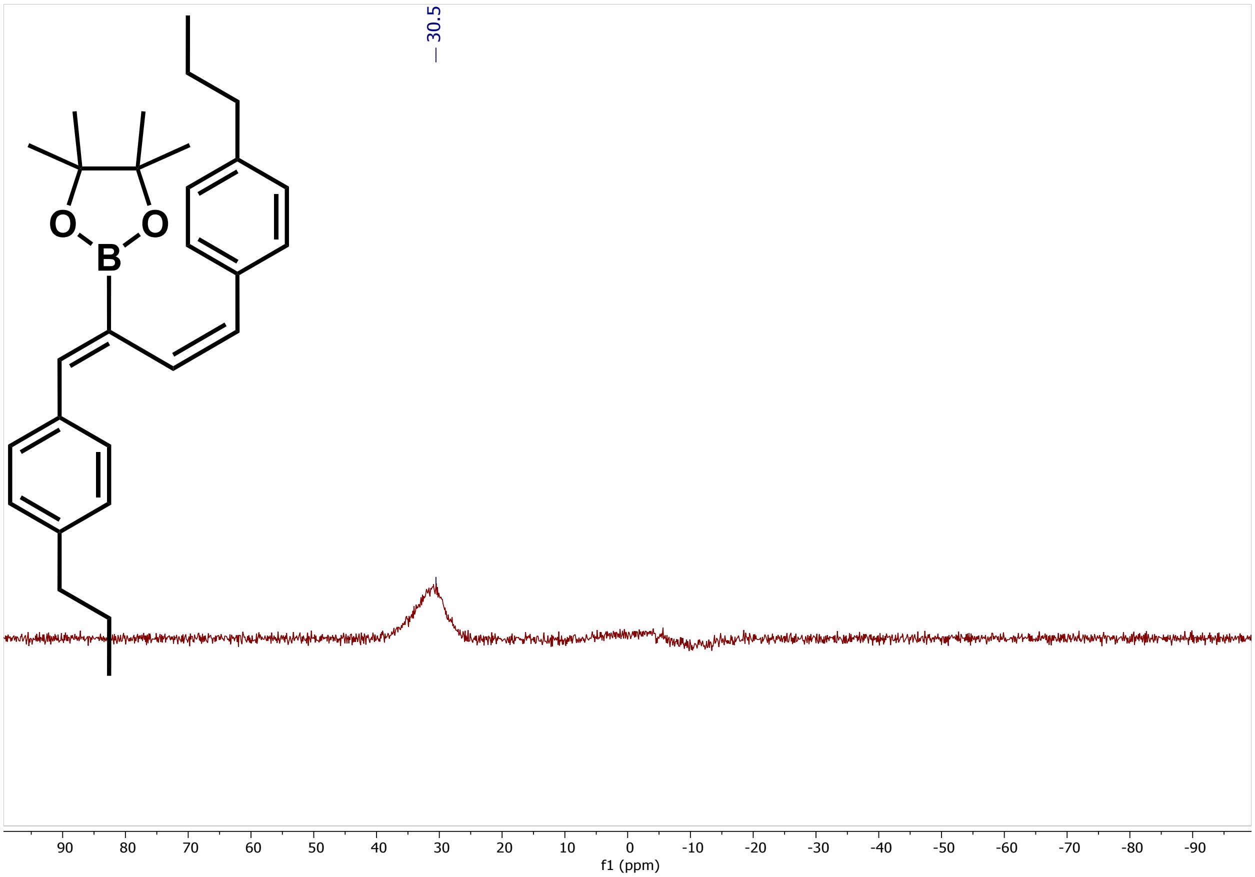

^{1}H NMR of **2f** ($CDCl_3$, 400 MHz)

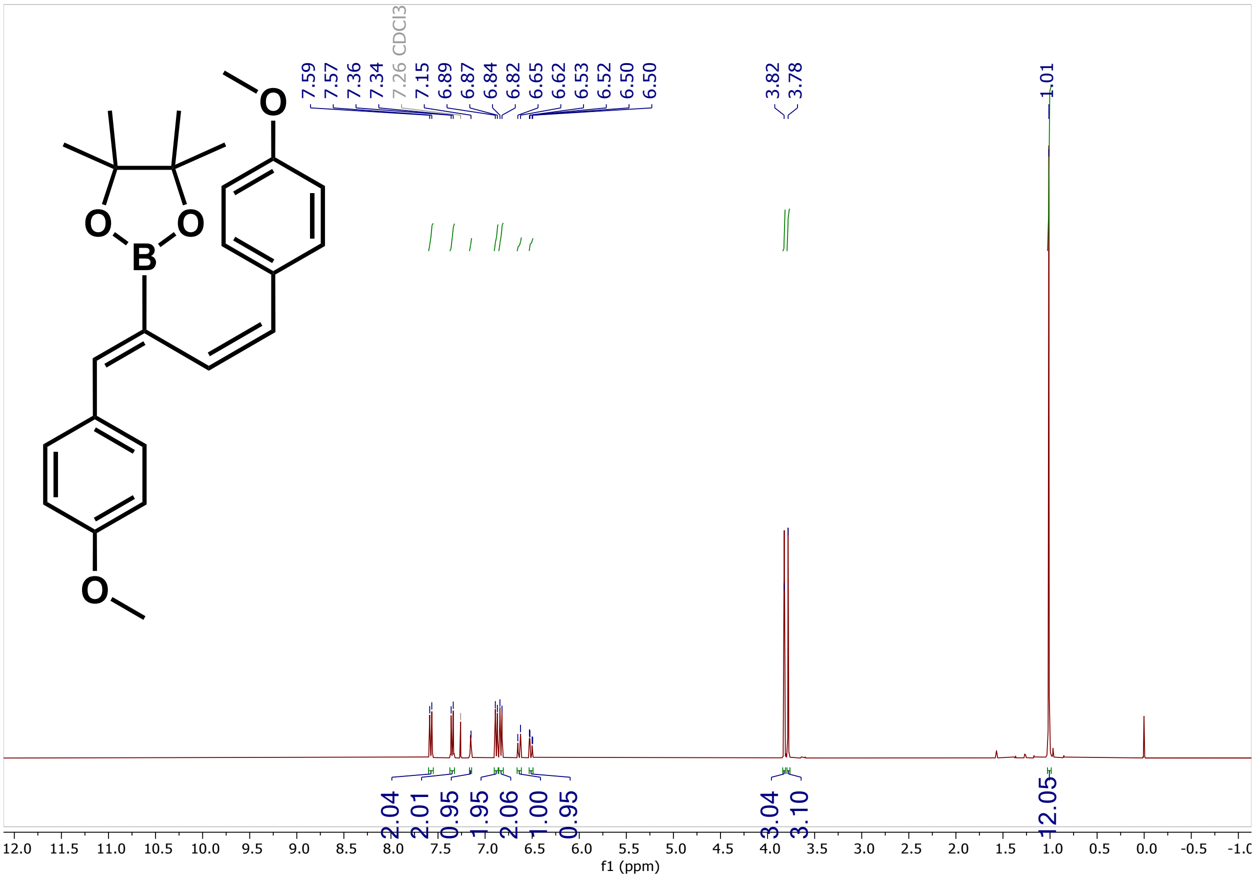

^{13}C NMR of **2f** ($CDCl_3$, 101 MHz)

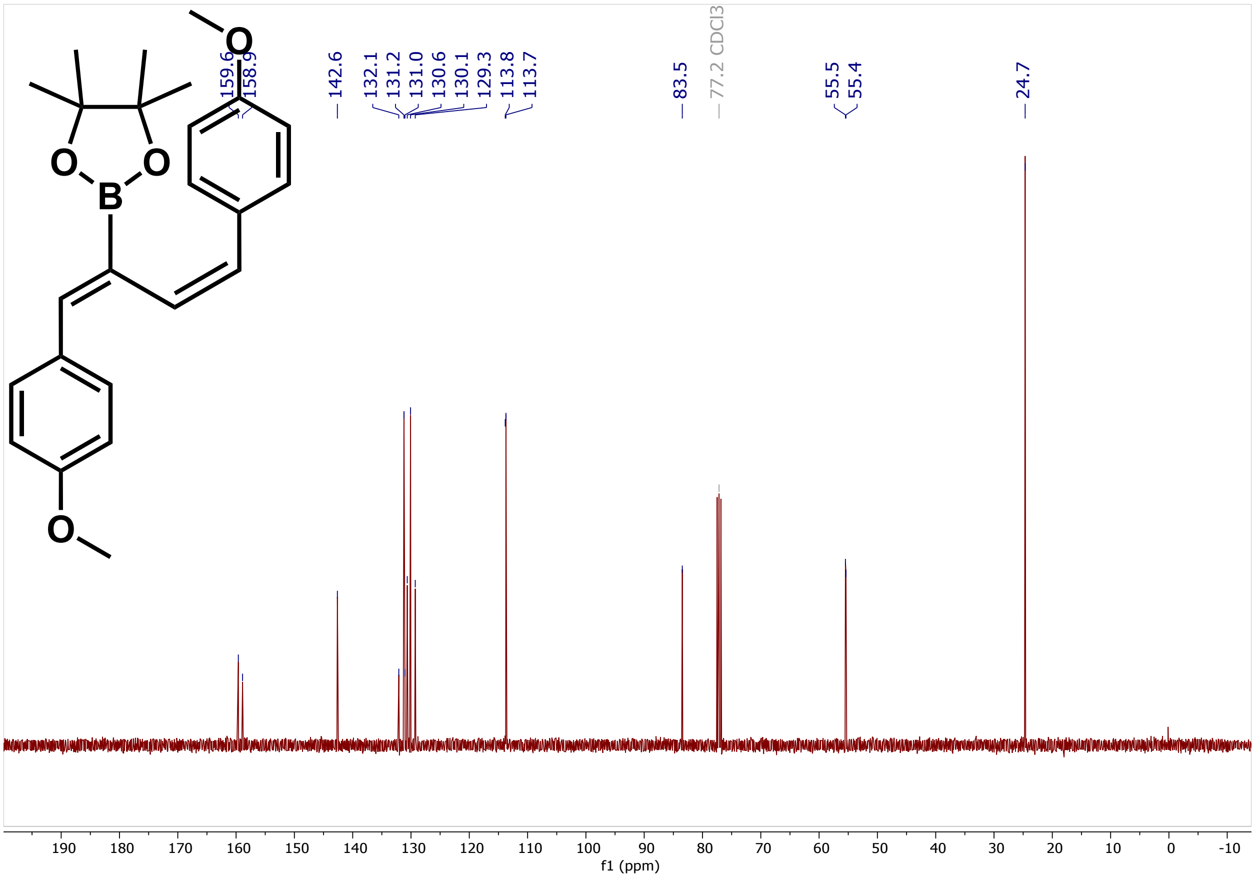

^{11}B NMR of **2f** ($CDCl_3$, 128 MHz)

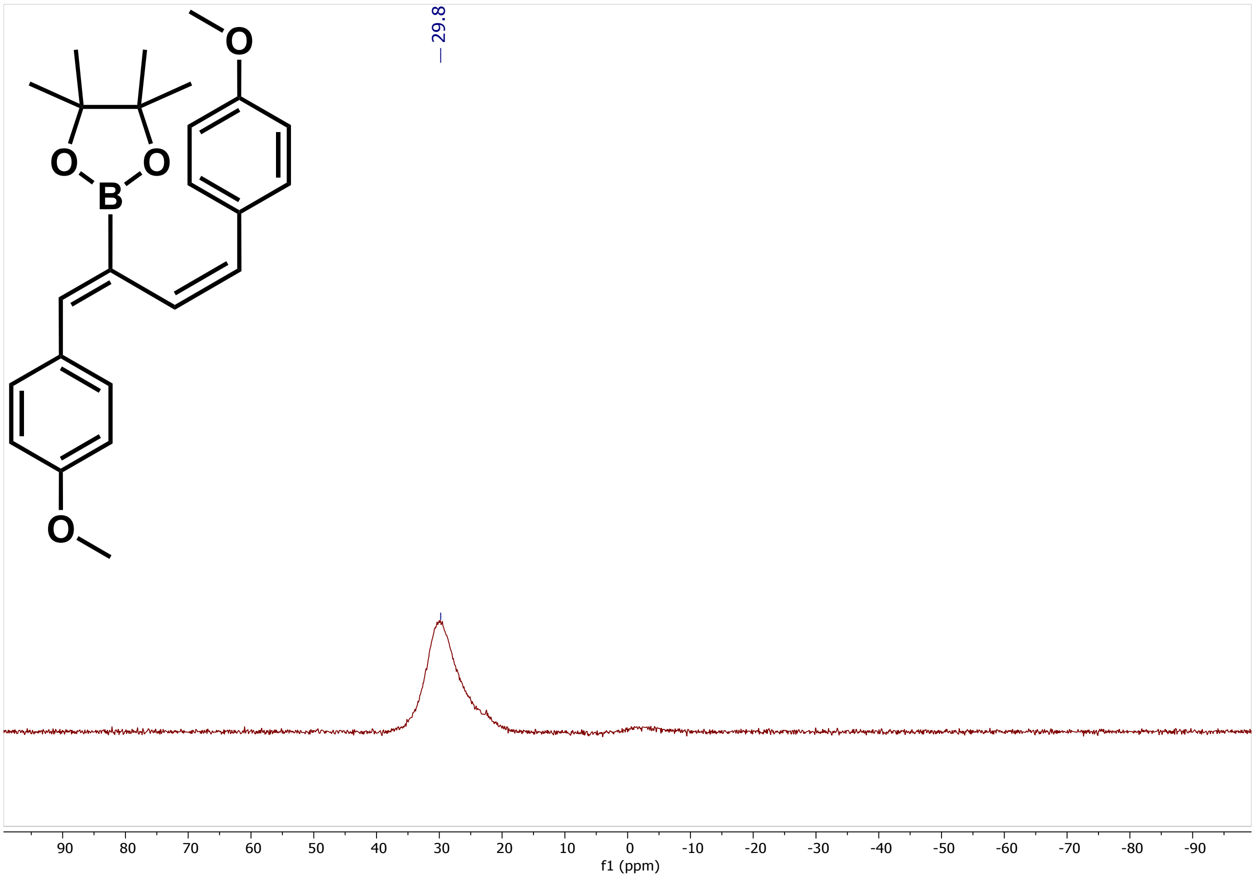

^{1}H NMR of **2g** ($CDCl_3$, 400 MHz)

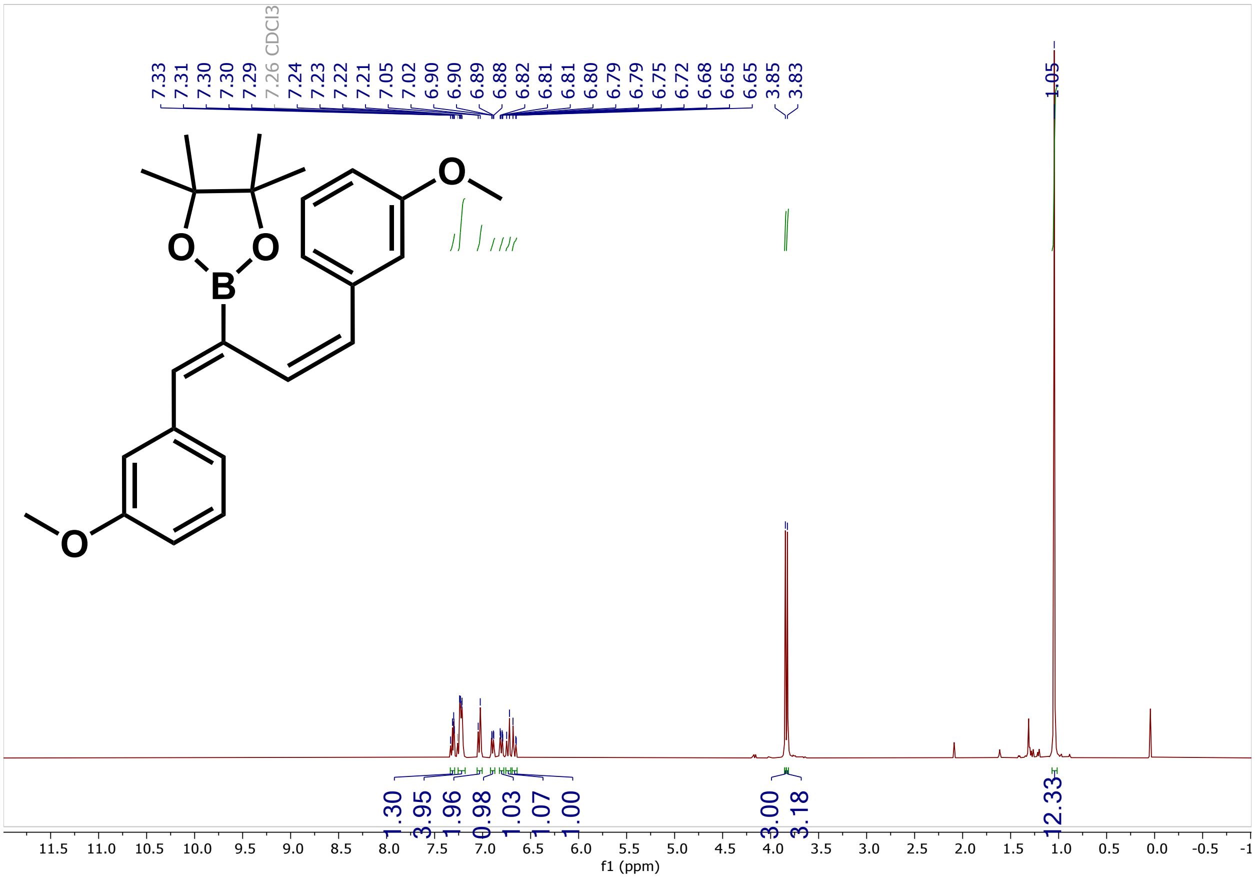

^{13}C NMR of **2g** ($CDCl_3$, 101 MHz)

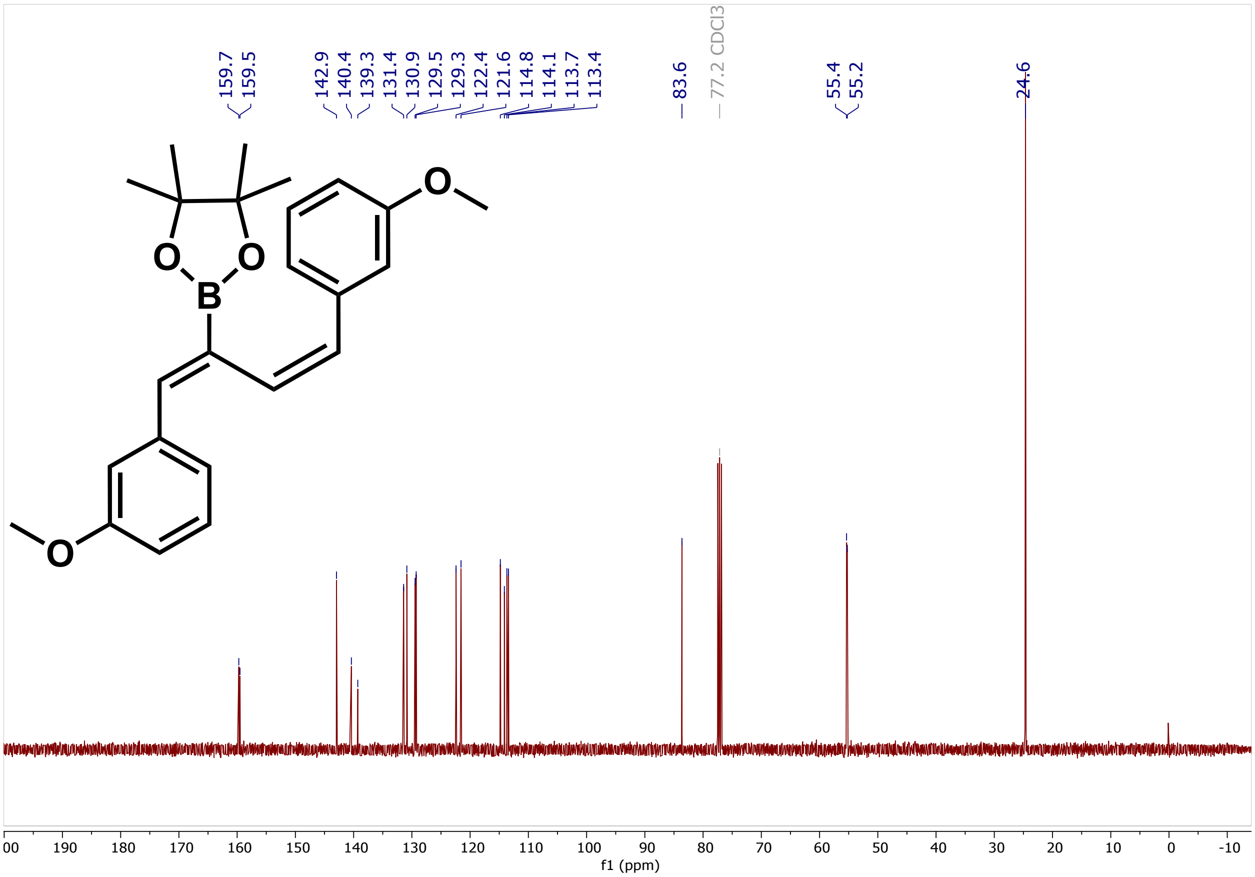

^{11}B NMR of **2g** ($CDCl_3$, 128 MHz)

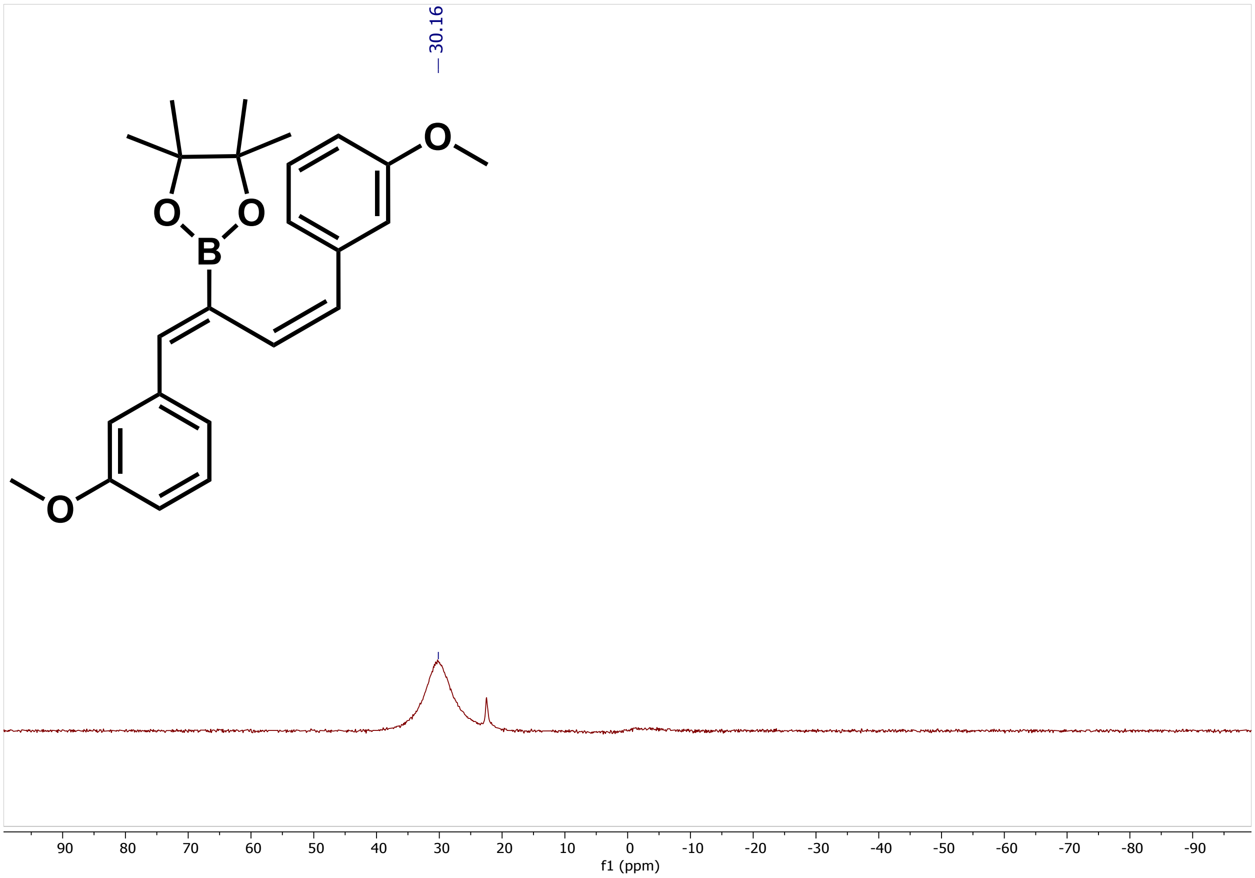

^{1}H NMR of **2h** ($CDCl_3$, 400 MHz)

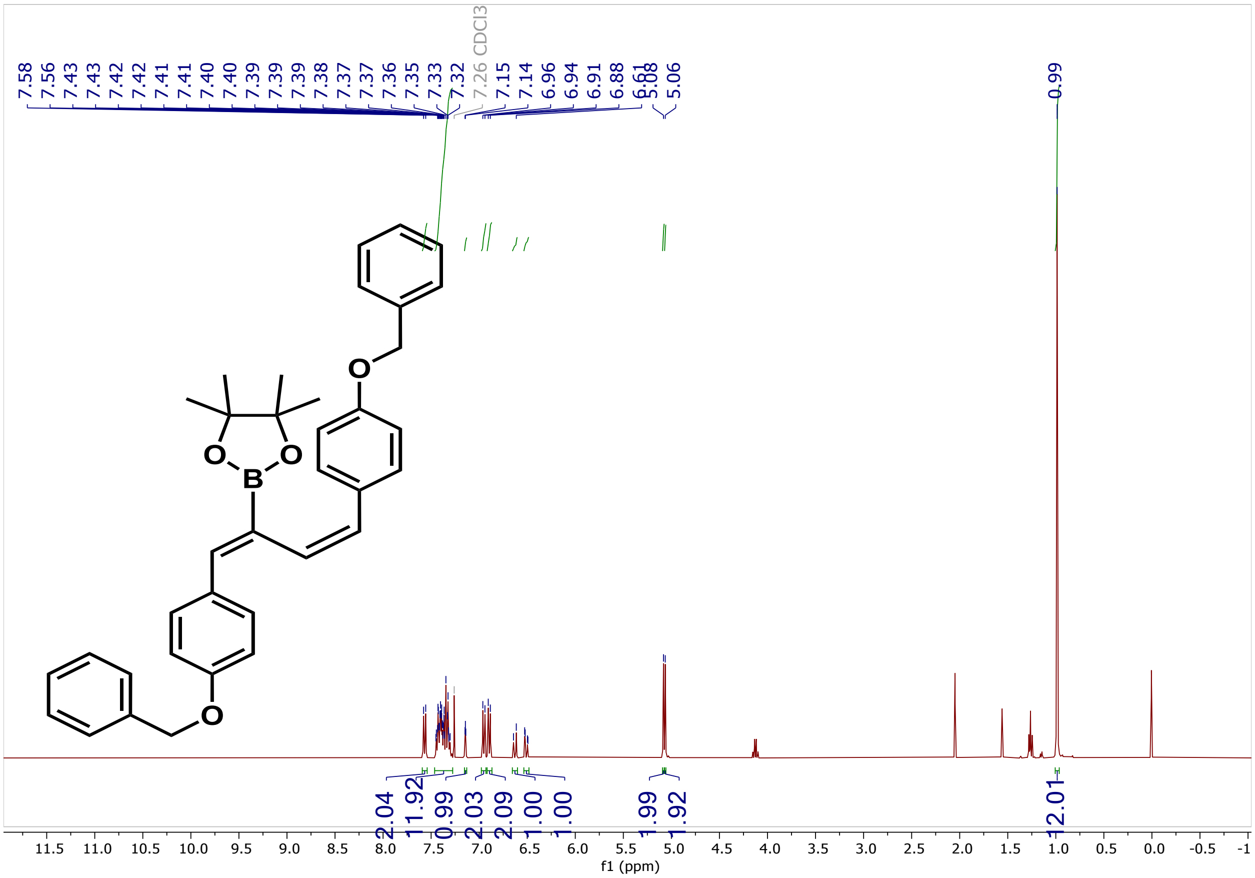

^{13}C NMR of **2h** ($CDCl_3$, 101 MHz)

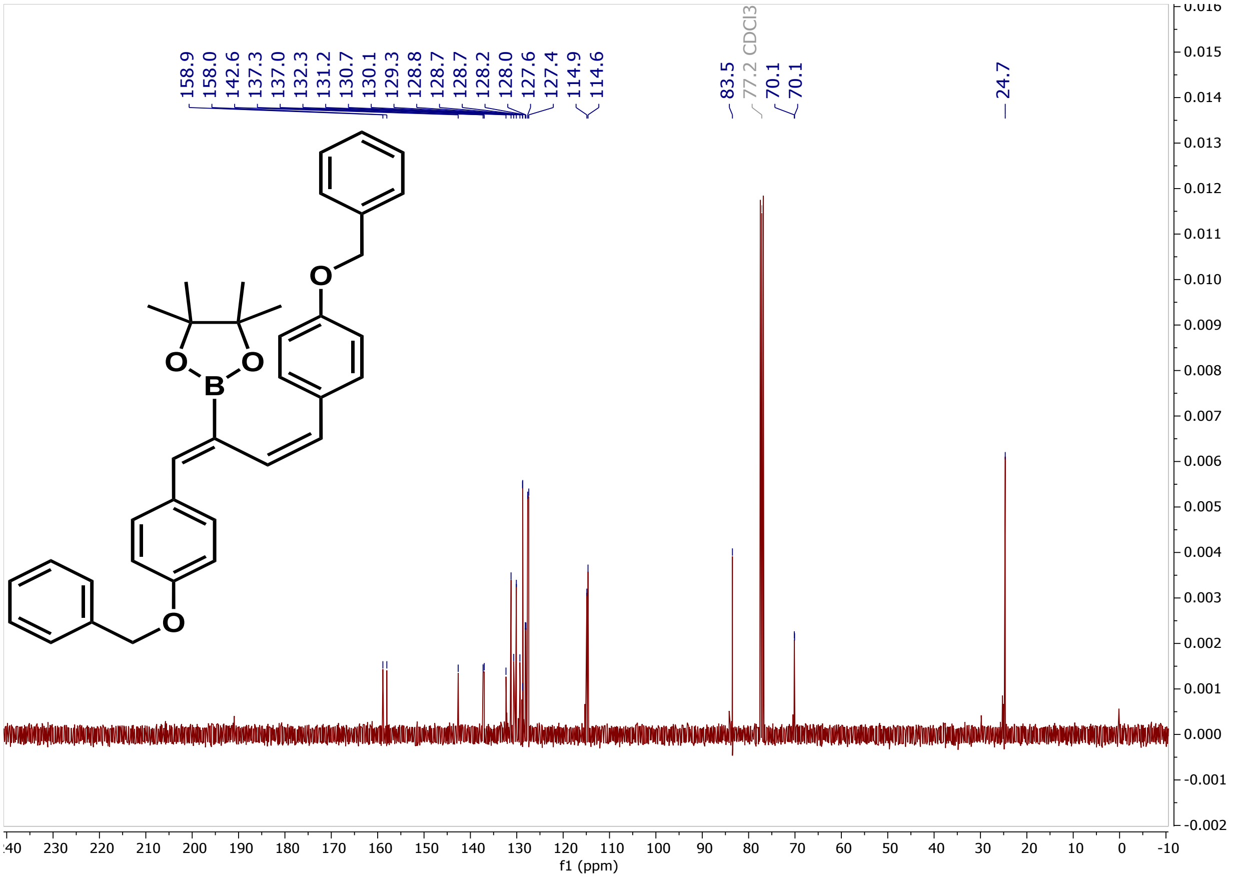

^{11}B NMR of **2h** ($CDCl_3$, 128 MHz)

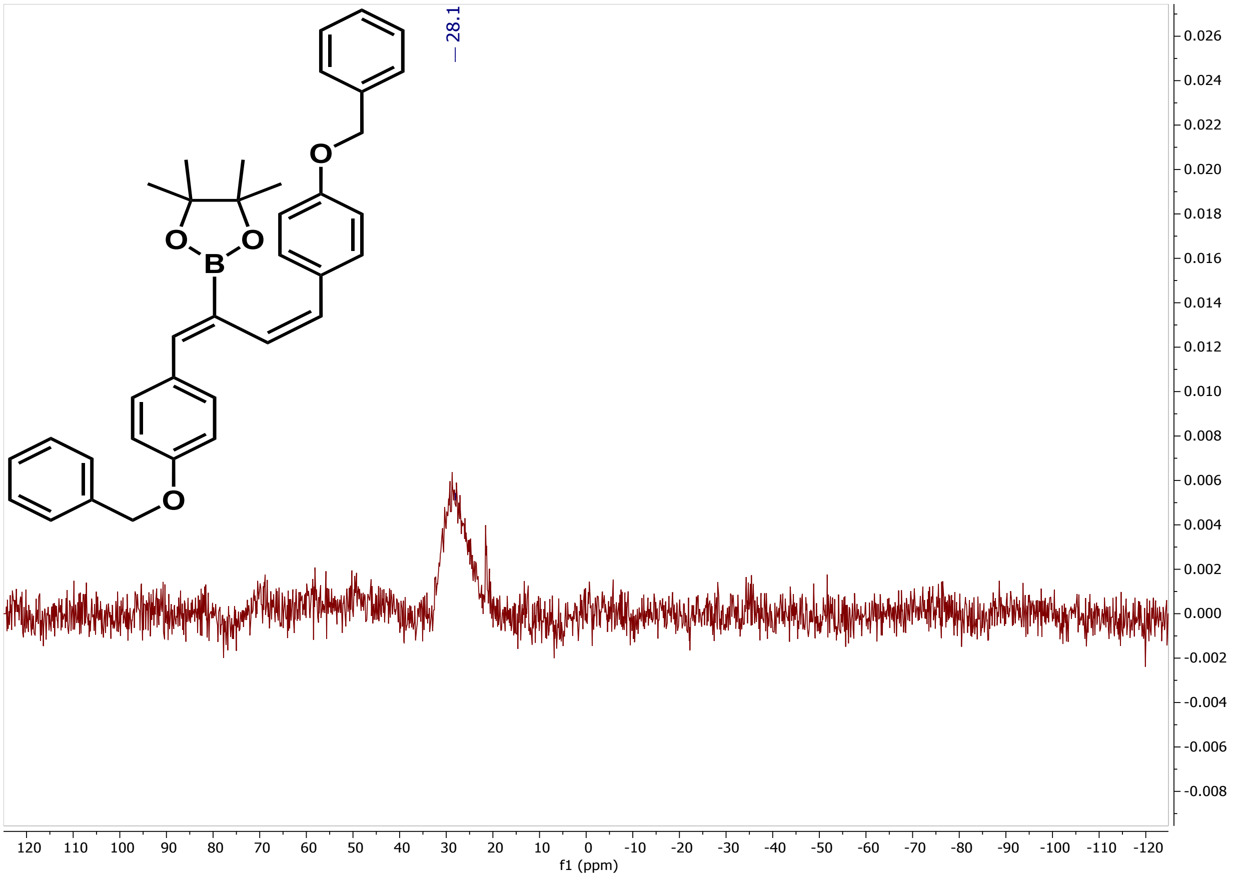

^{1}H NMR of **2i** ($CDCl_3$, 400 MHz)

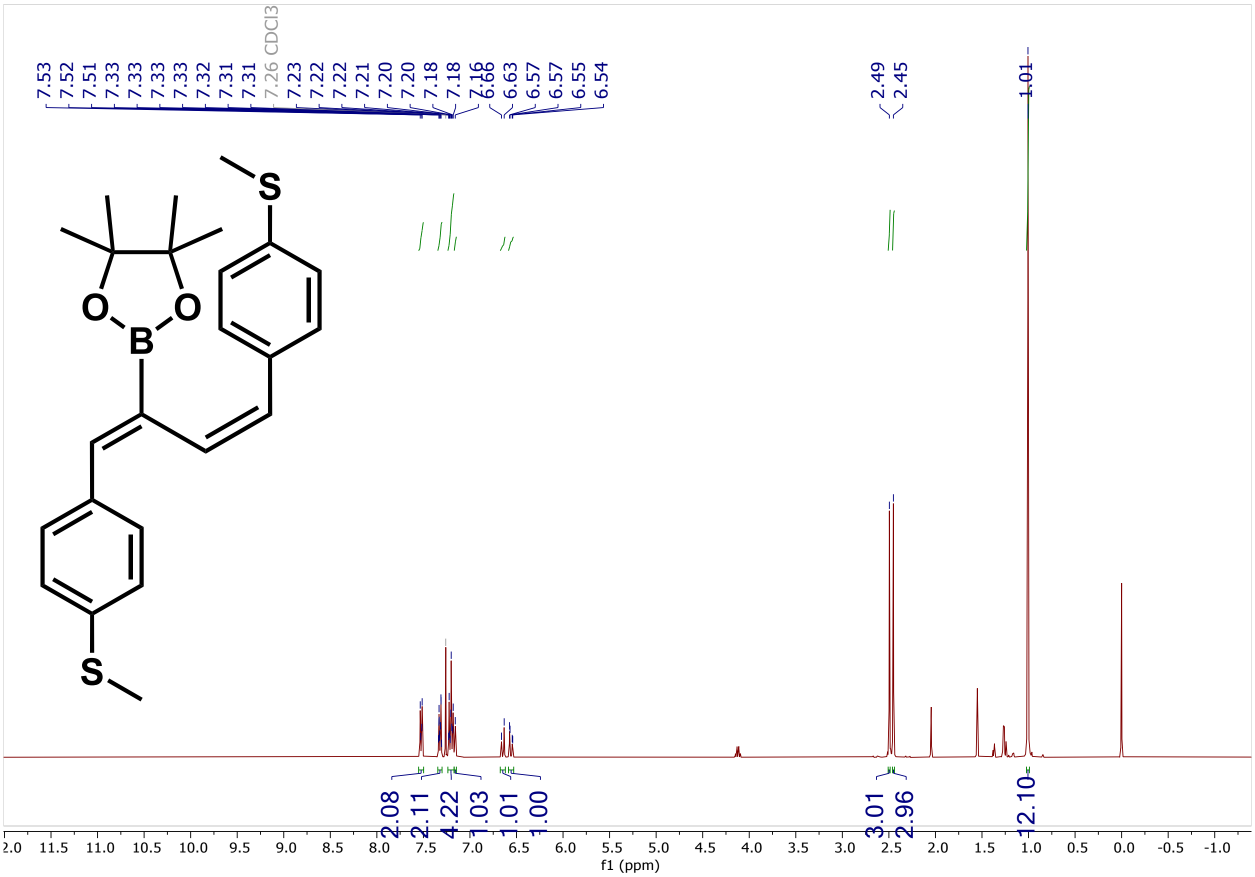

^{13}C NMR of **2i** ($CDCl_3$, 101 MHz)

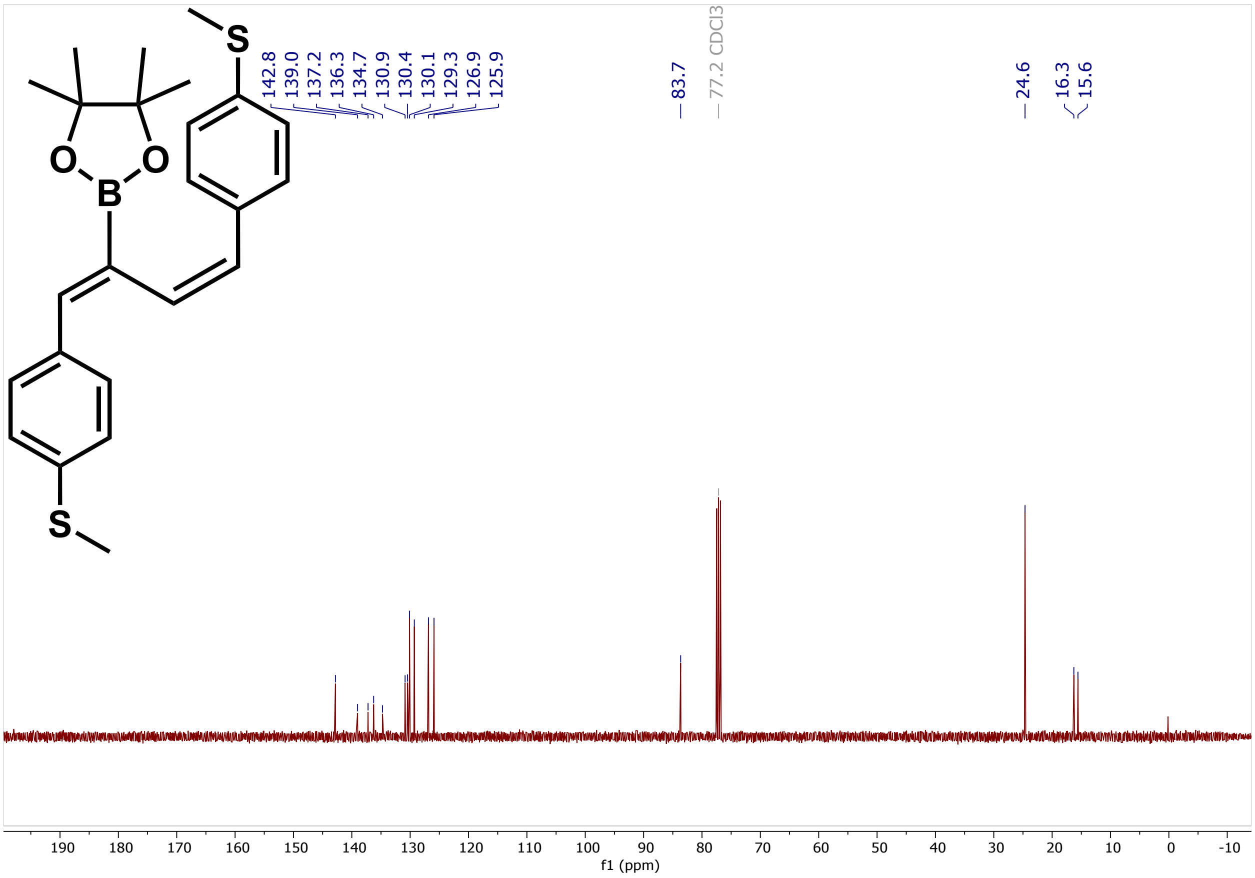

^{11}B NMR of **2i** ($CDCl_3$, 128 MHz)

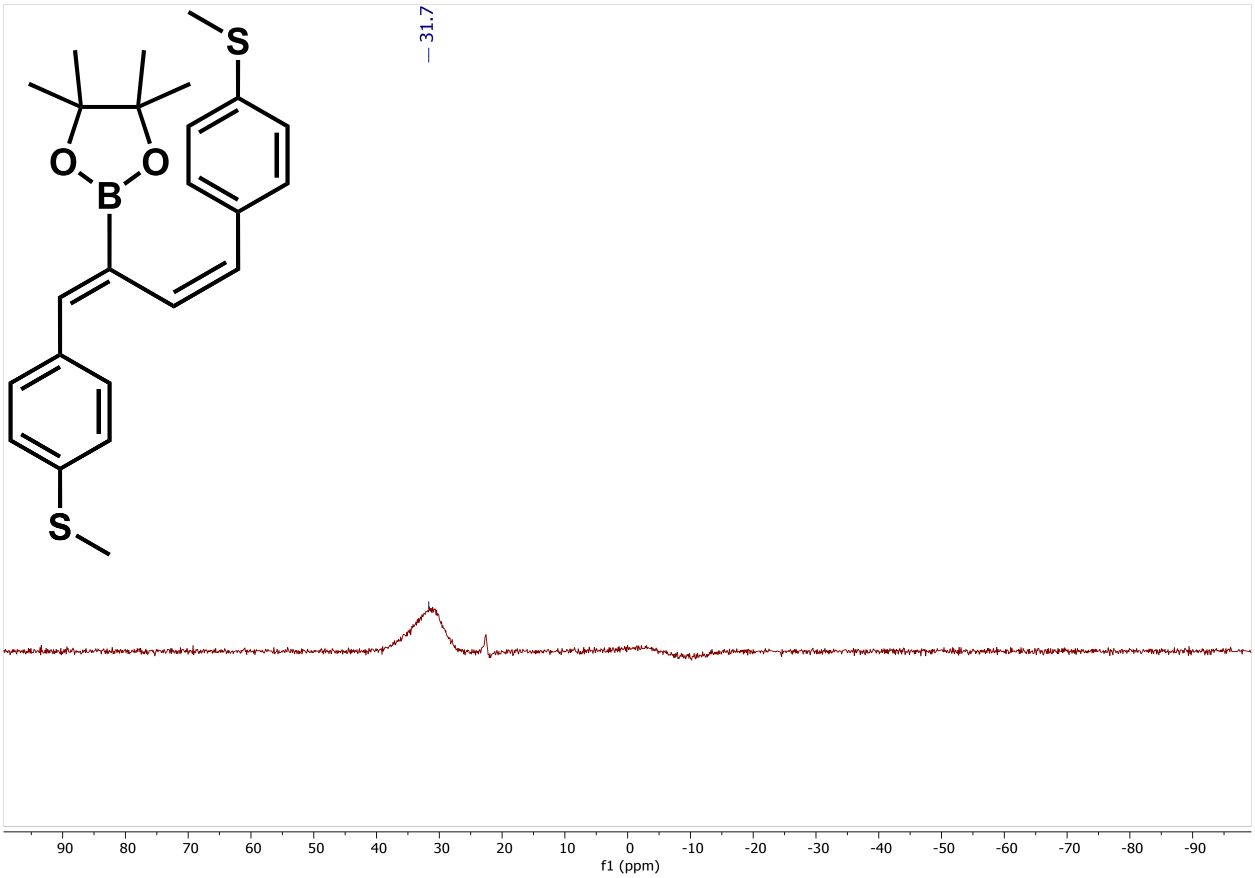

^{1}H NMR of **2j** ($CDCl_3$, 400 MHz)

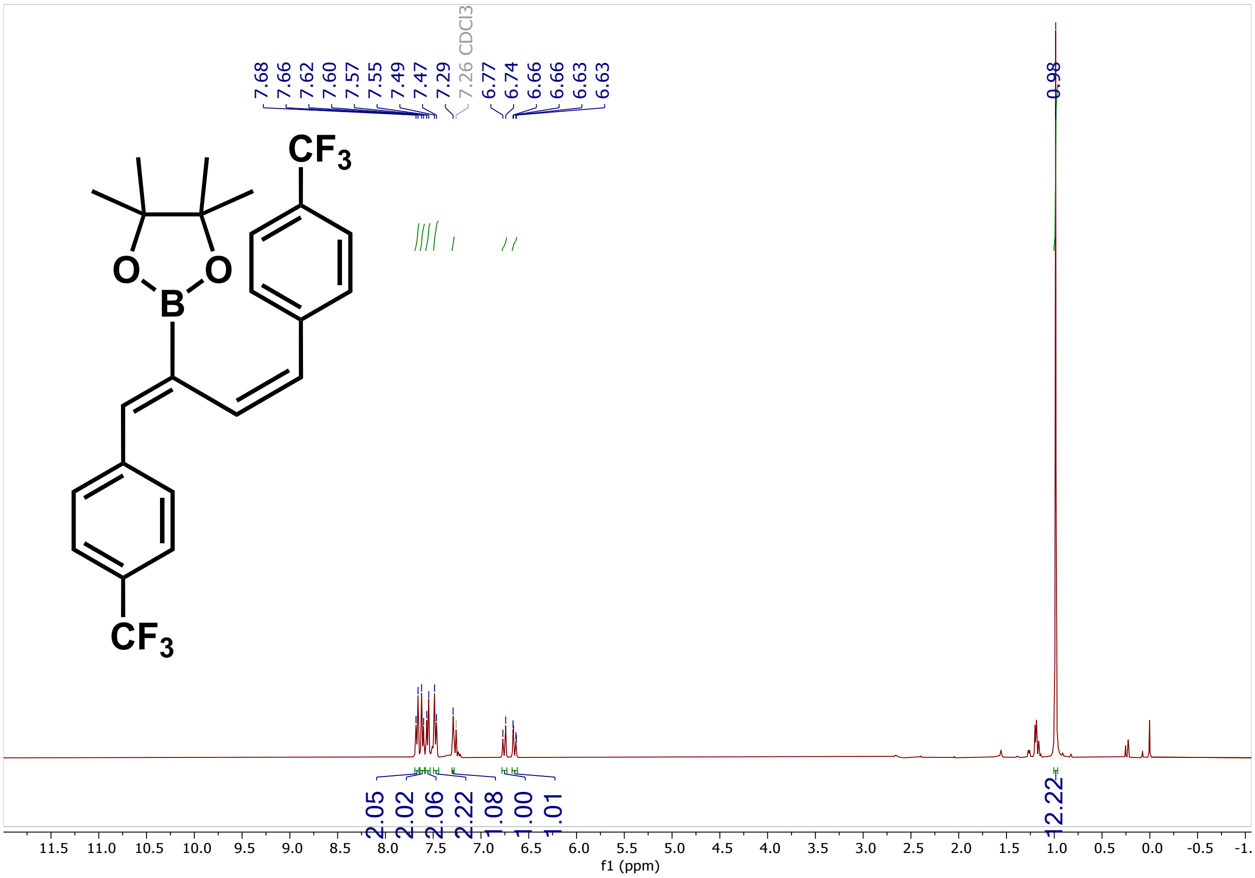

^{13}C NMR of **2j** ($CDCl_3$, 101 MHz)

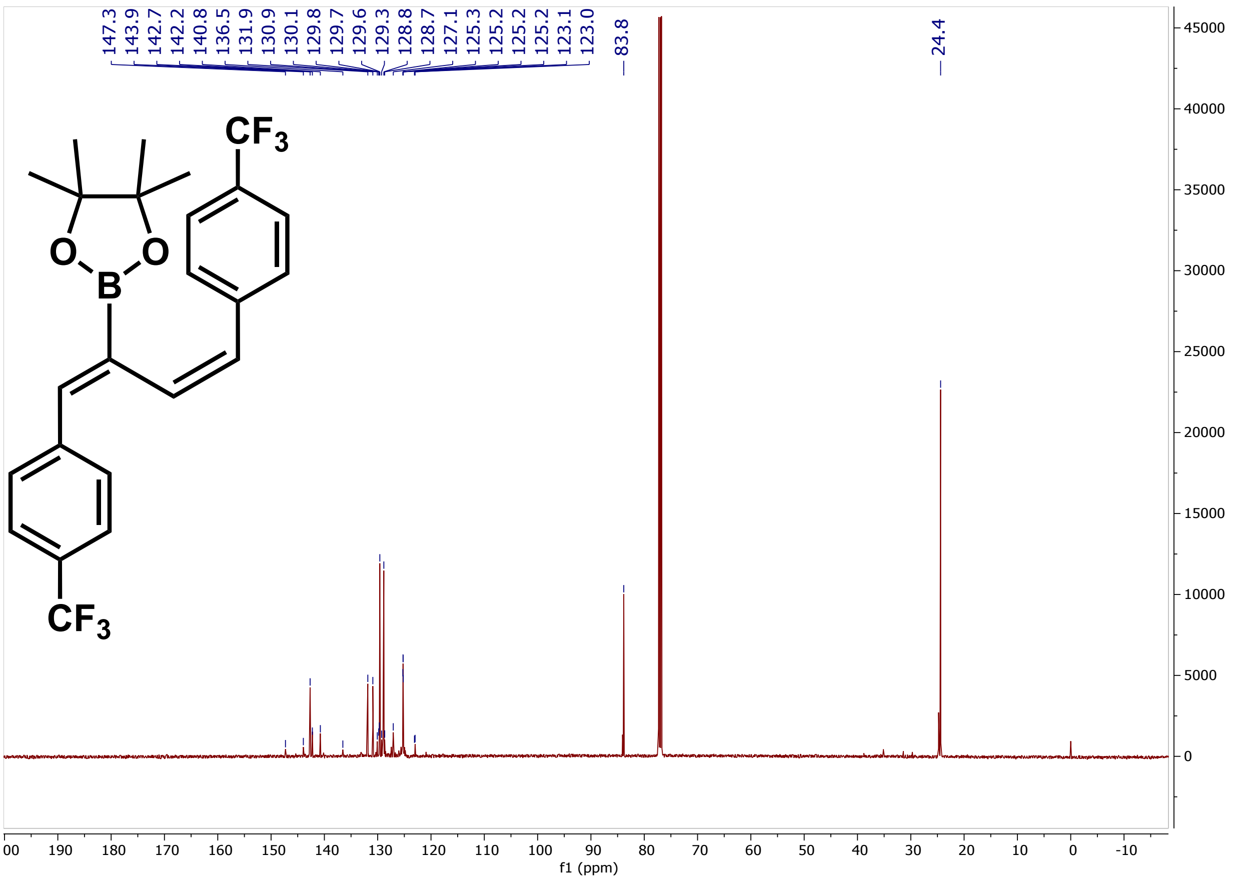

^{11}B NMR of **2j** ($CDCl_3$, 128 MHz)

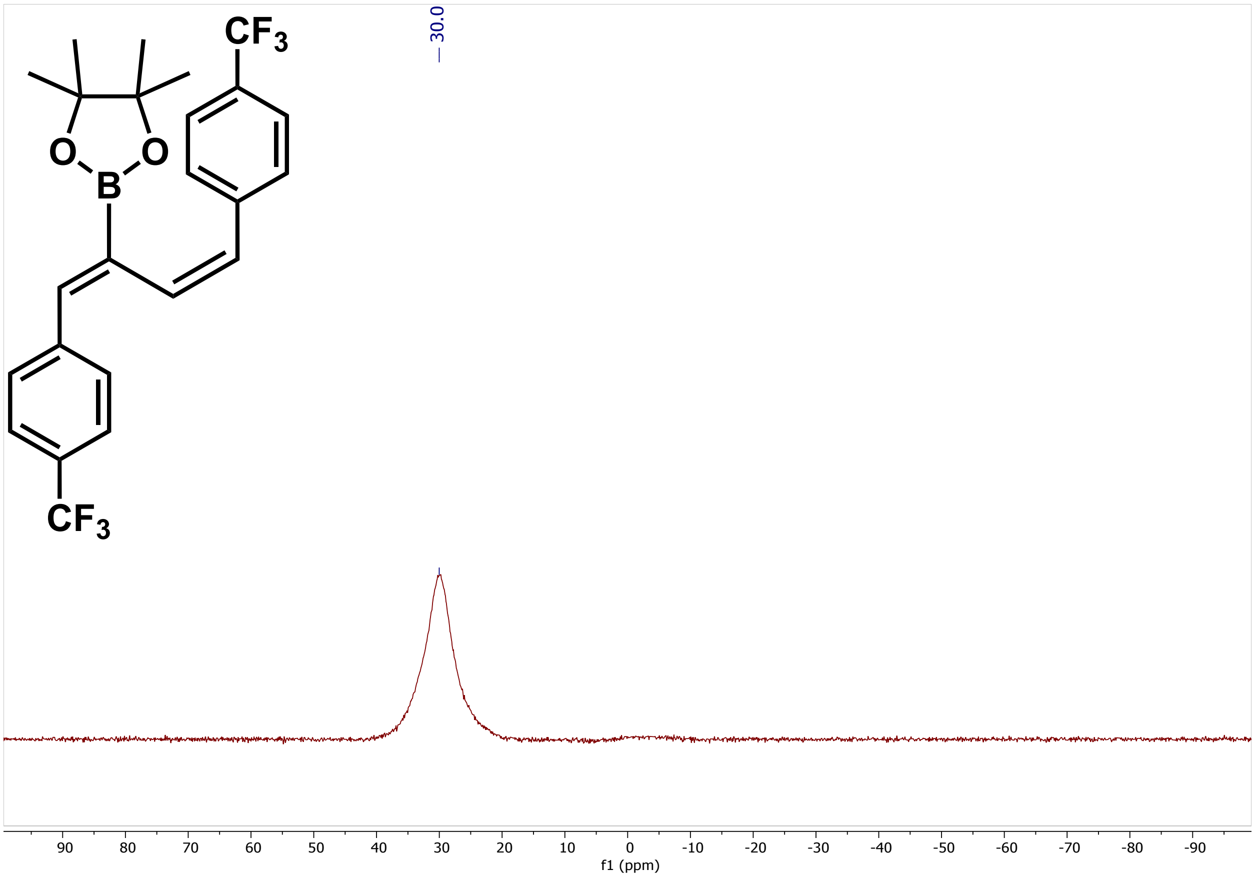

^{19}F NMR of **2j** ($CDCl_3$, 376 MHz)

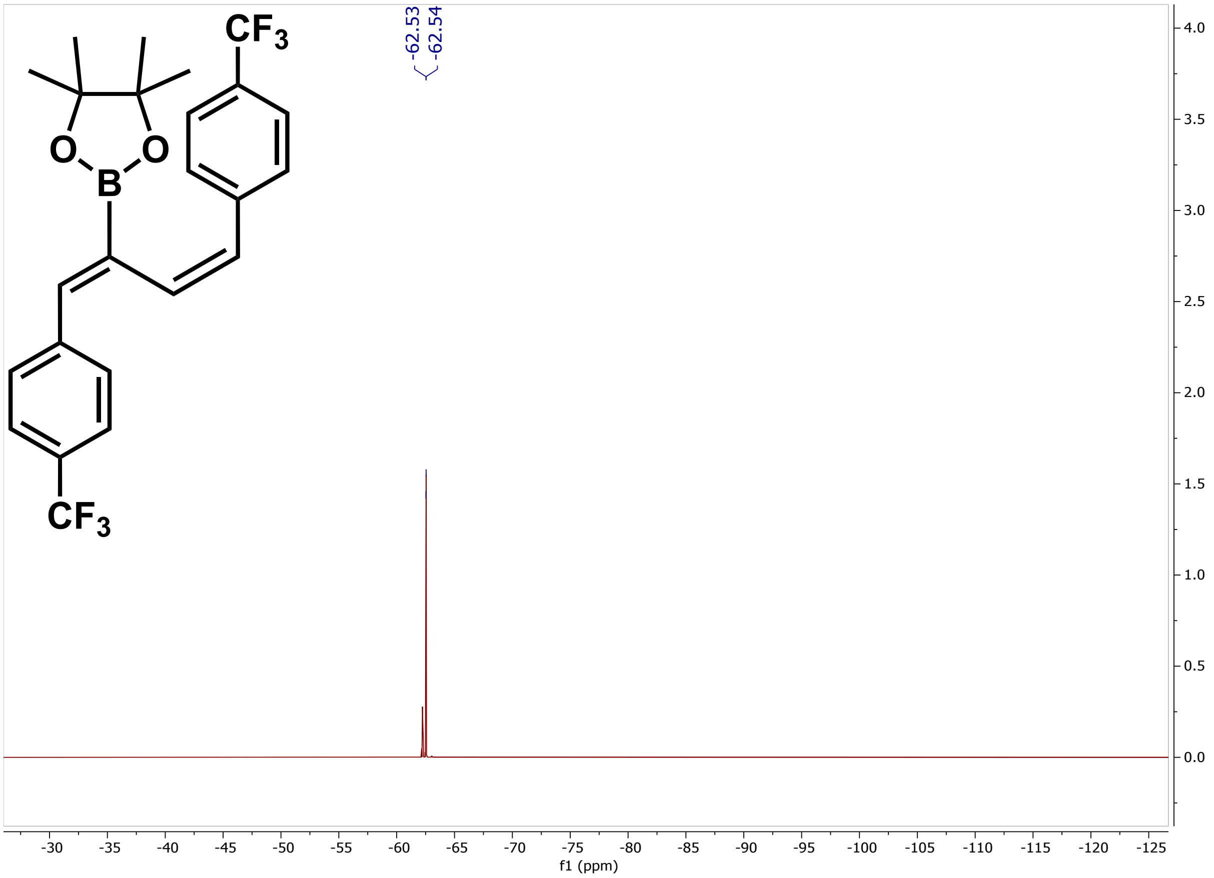

^{1}H NMR of **2k** ($CDCl_3$, 400 MHz)

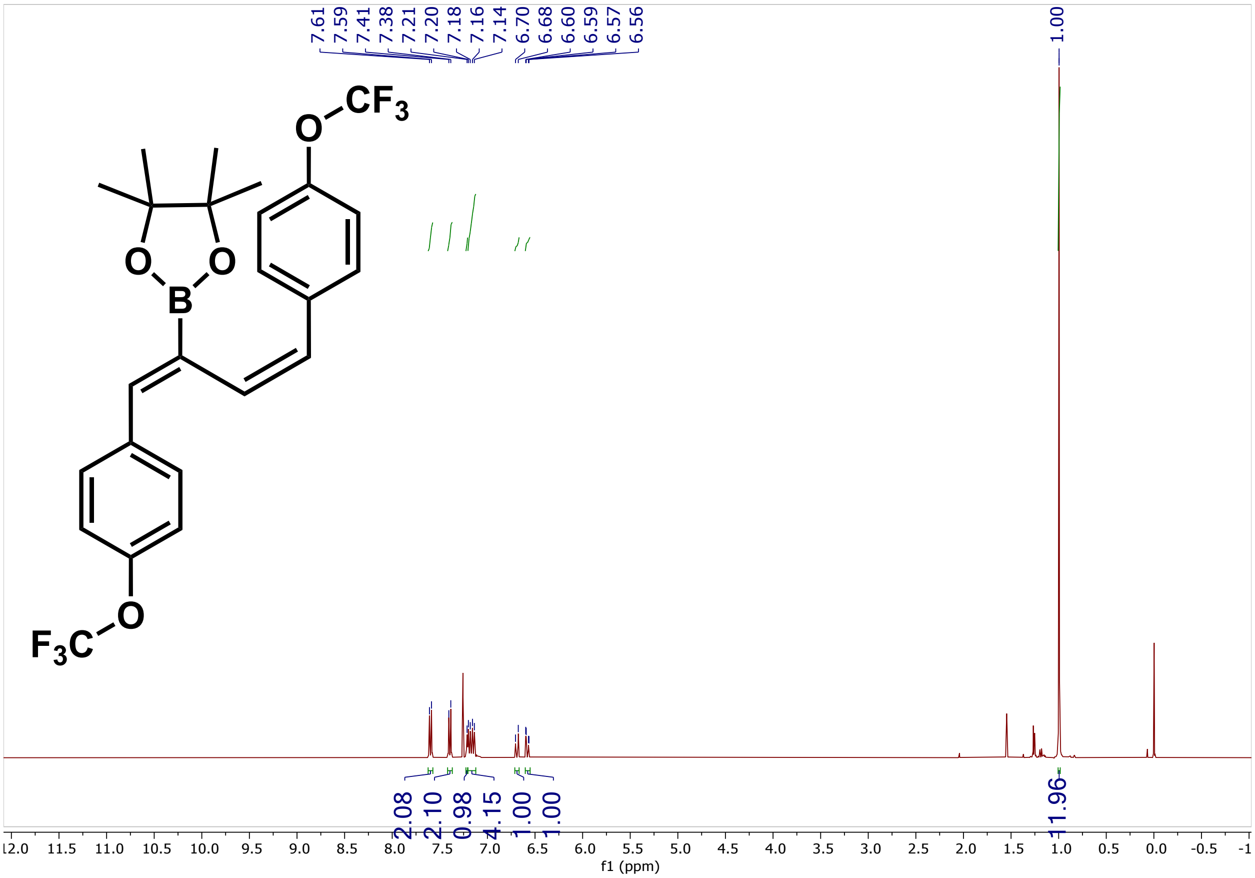

^{13}C NMR of **2k** ($CDCl_3$, 101 MHz)

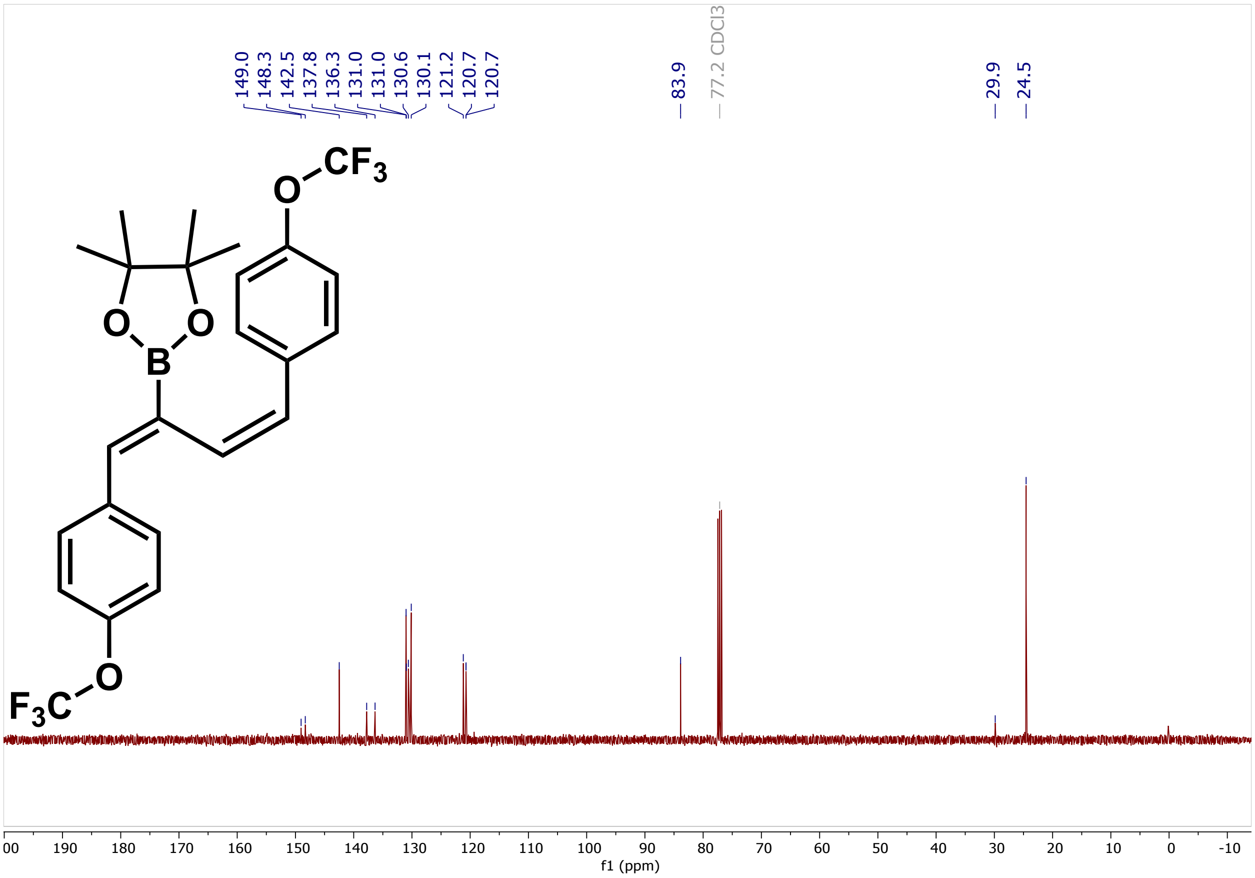

^{11}B NMR of **2k** ($CDCl_3$, 128 MHz)

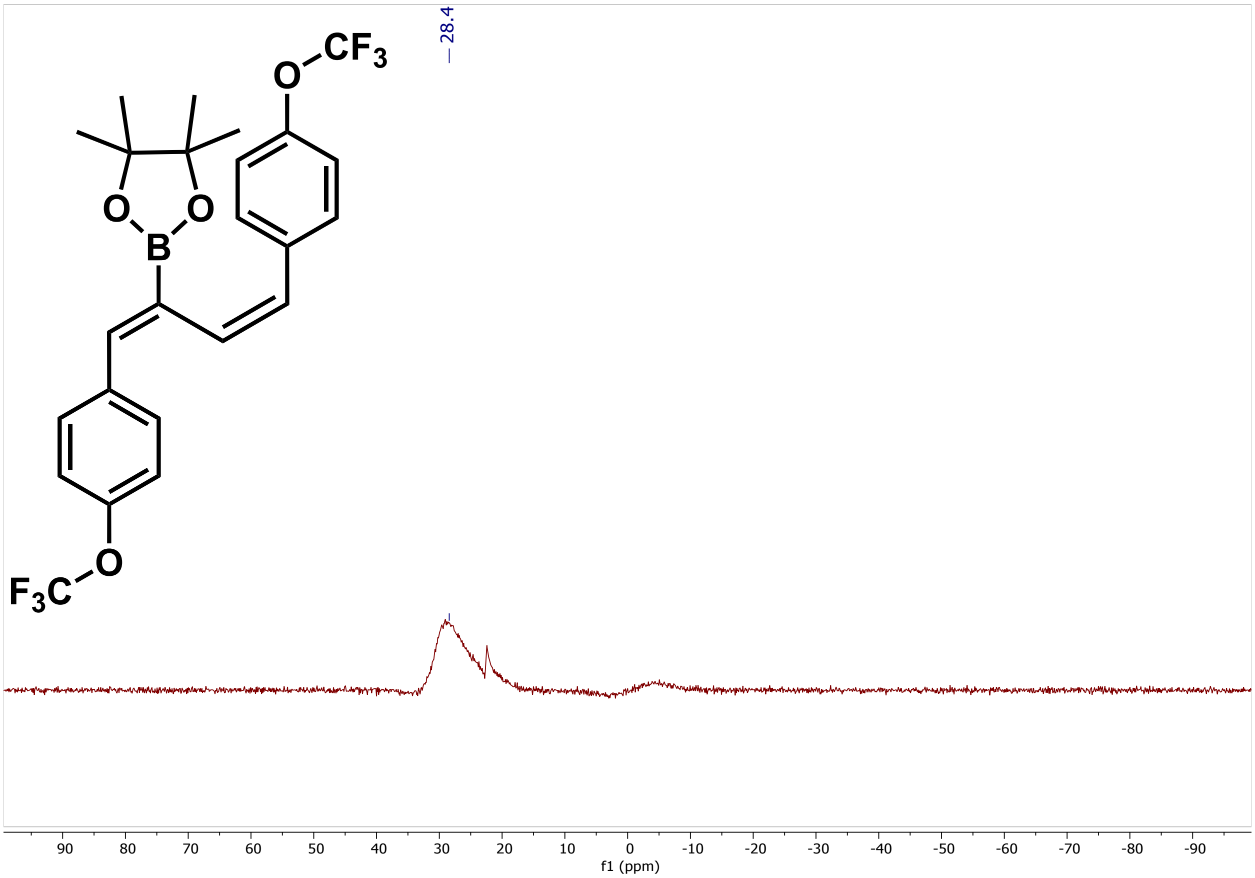

^{19}F NMR of **2k** ($CDCl_3$, 376 MHz)

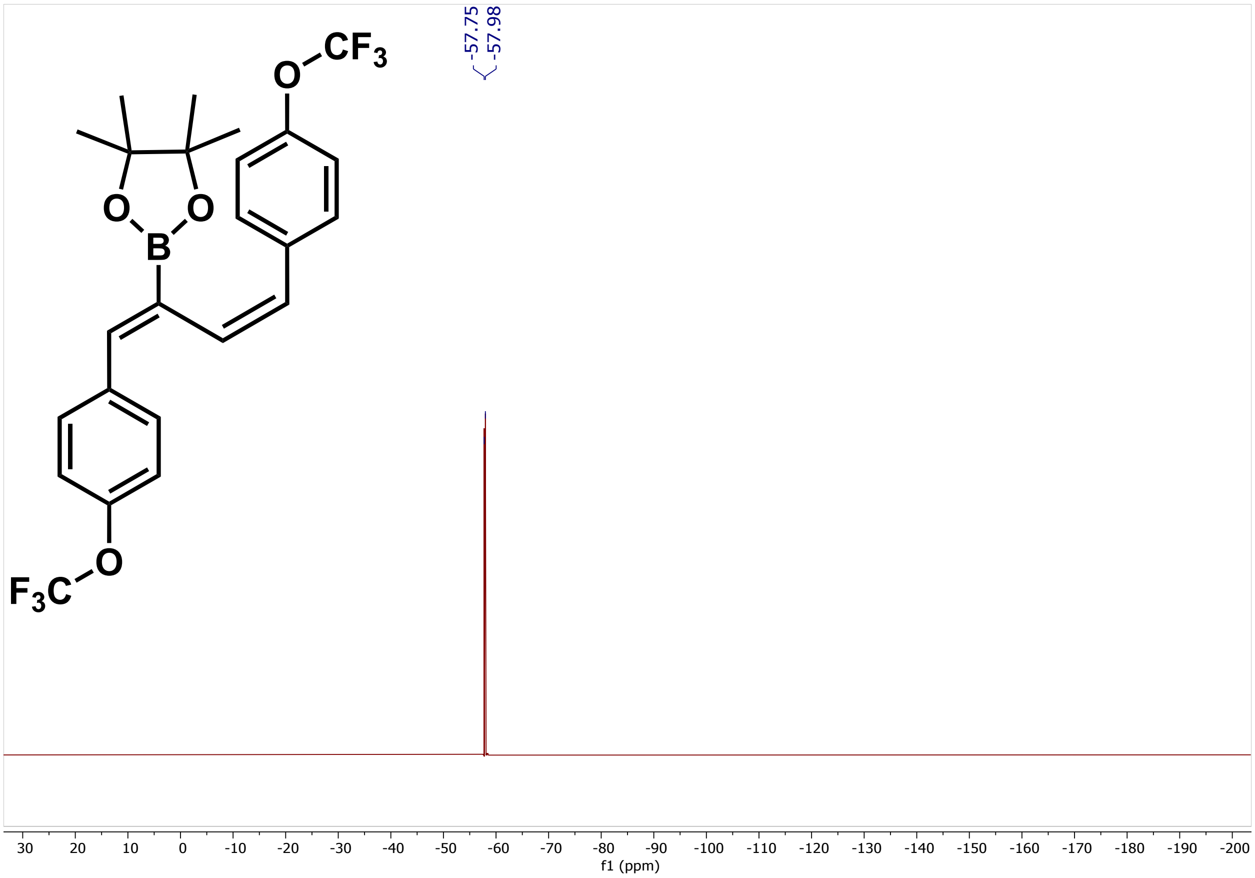

^{1}H NMR of **2l** ($CDCl_3$, 400 MHz)

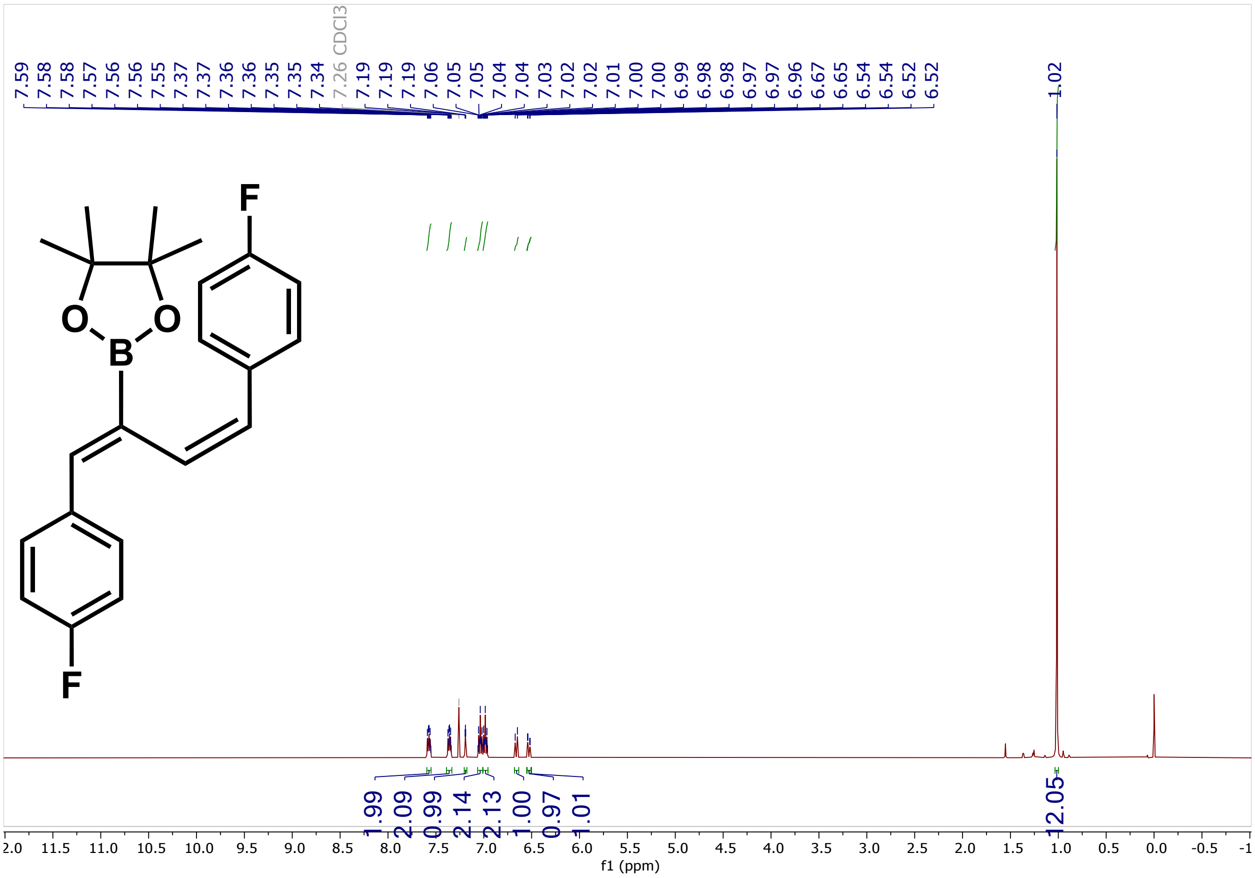

^{13}C NMR of **2l** ($CDCl_3$, 101 MHz)

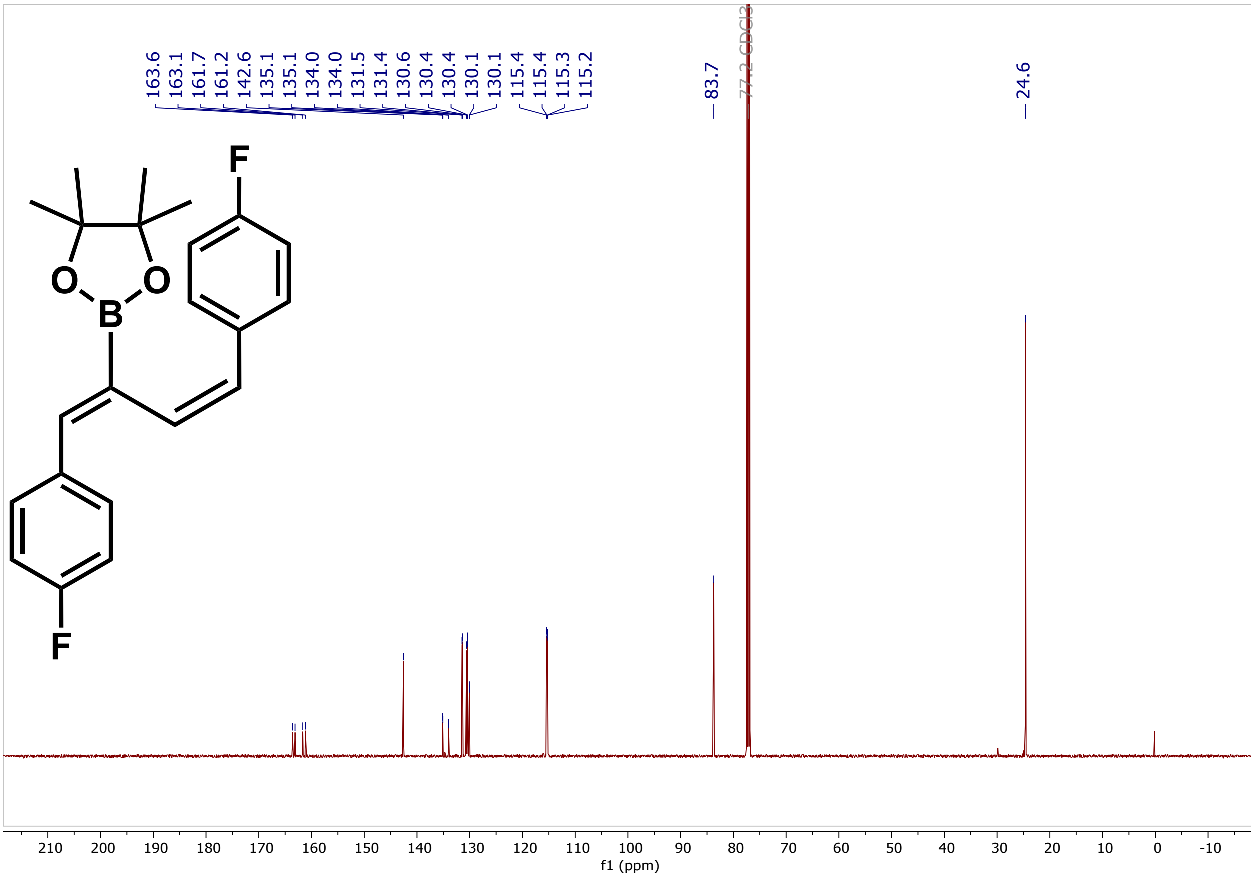

^{11}B NMR of **2l** ($CDCl_3$, 128 MHz)

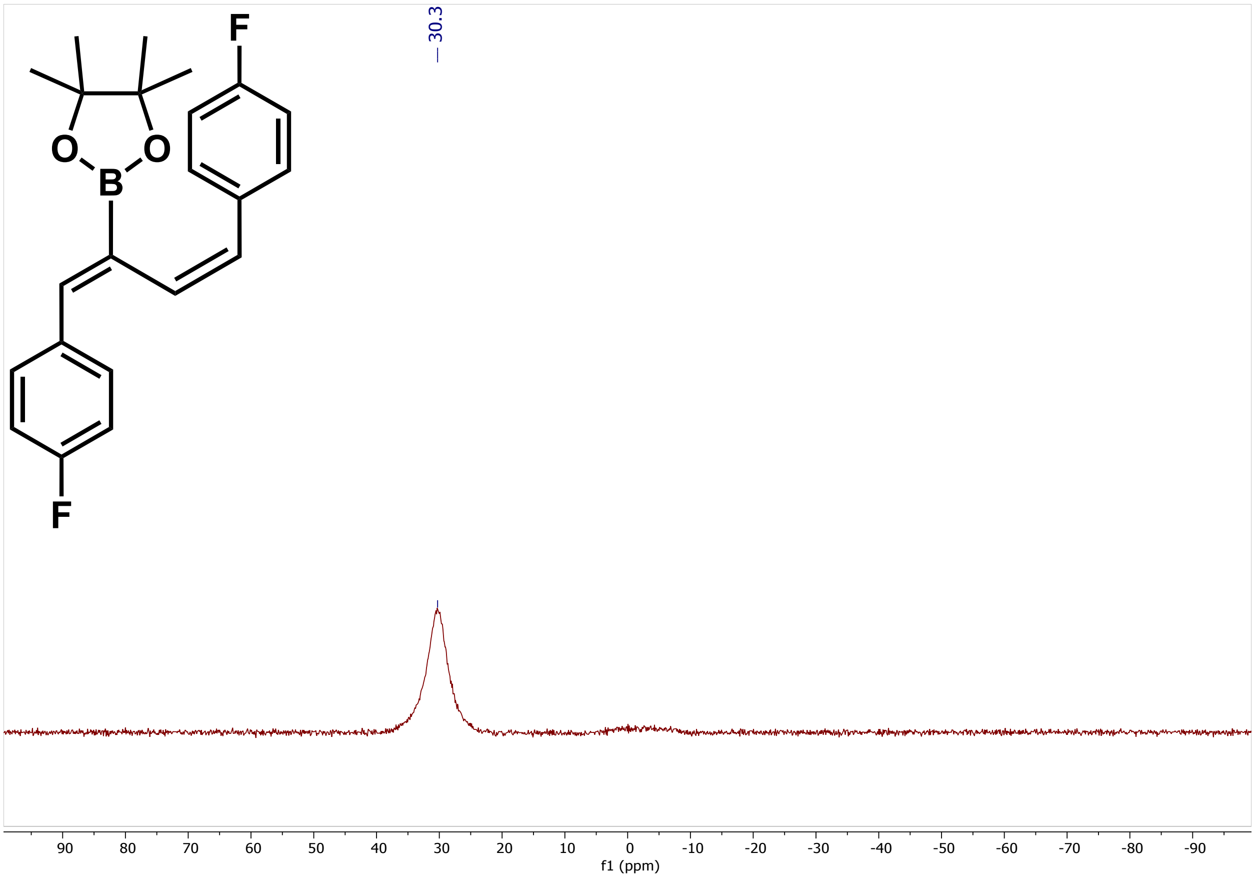

^{19}F NMR of **2l** ($CDCl_3$, 376 MHz)

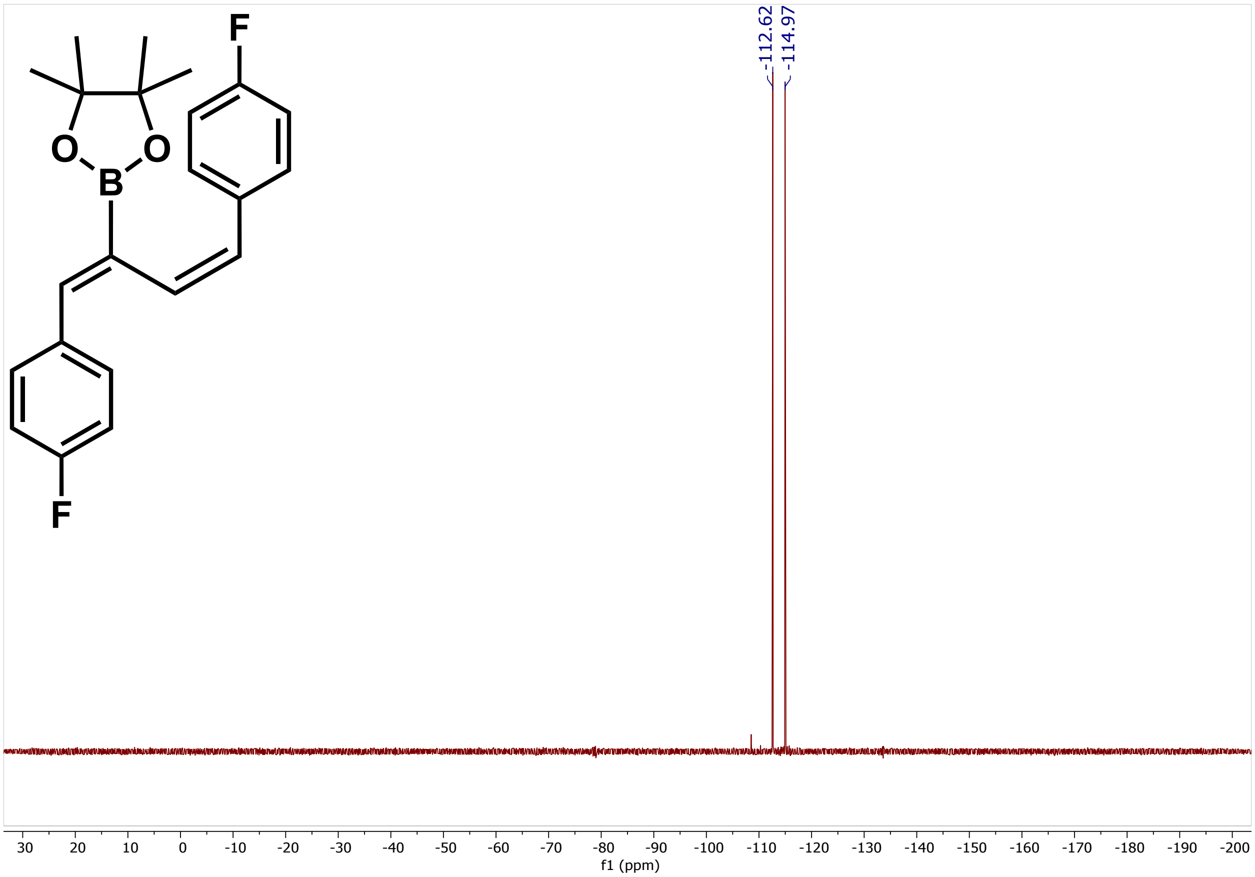

^{1}H NMR of **2m** ($CDCl_3$, 400 MHz)

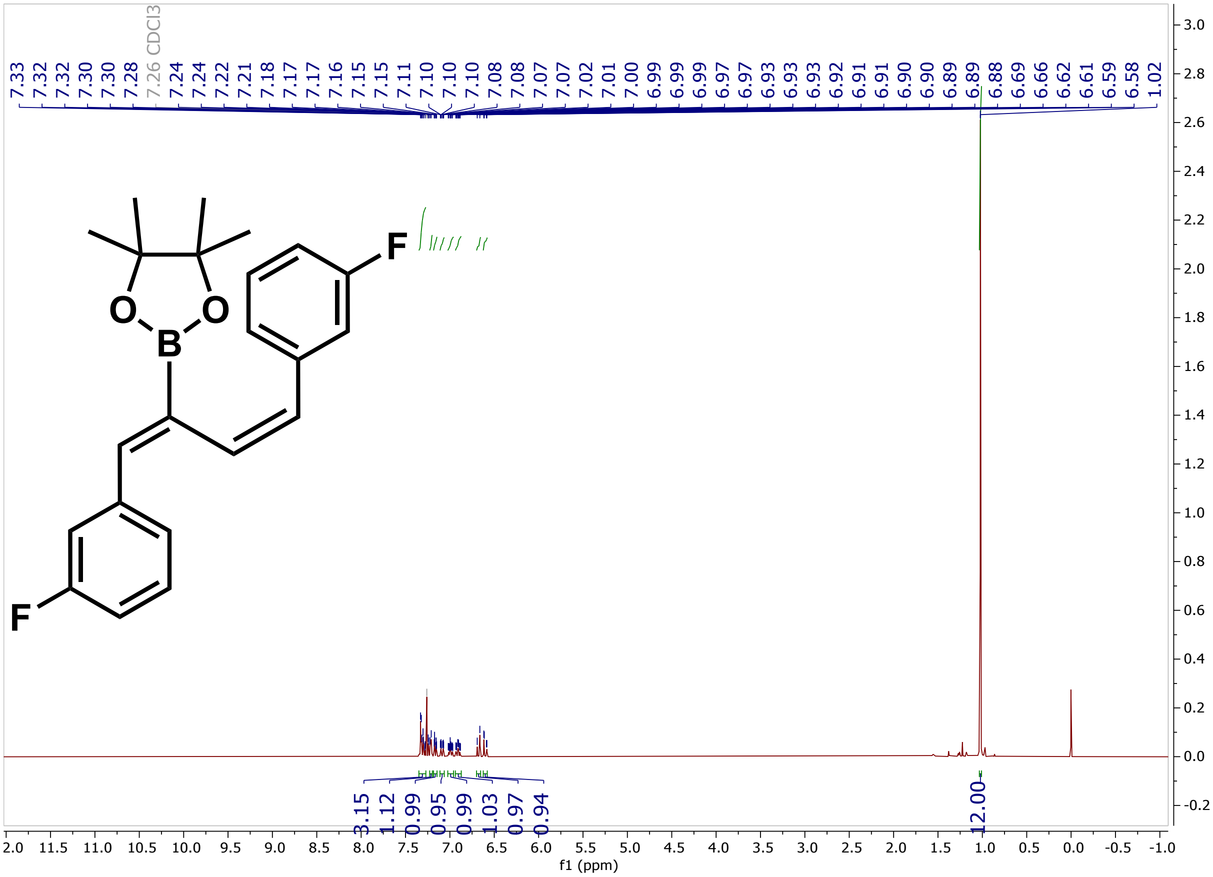

^{13}C NMR of **2m** ($CDCl_3$, 101 MHz)

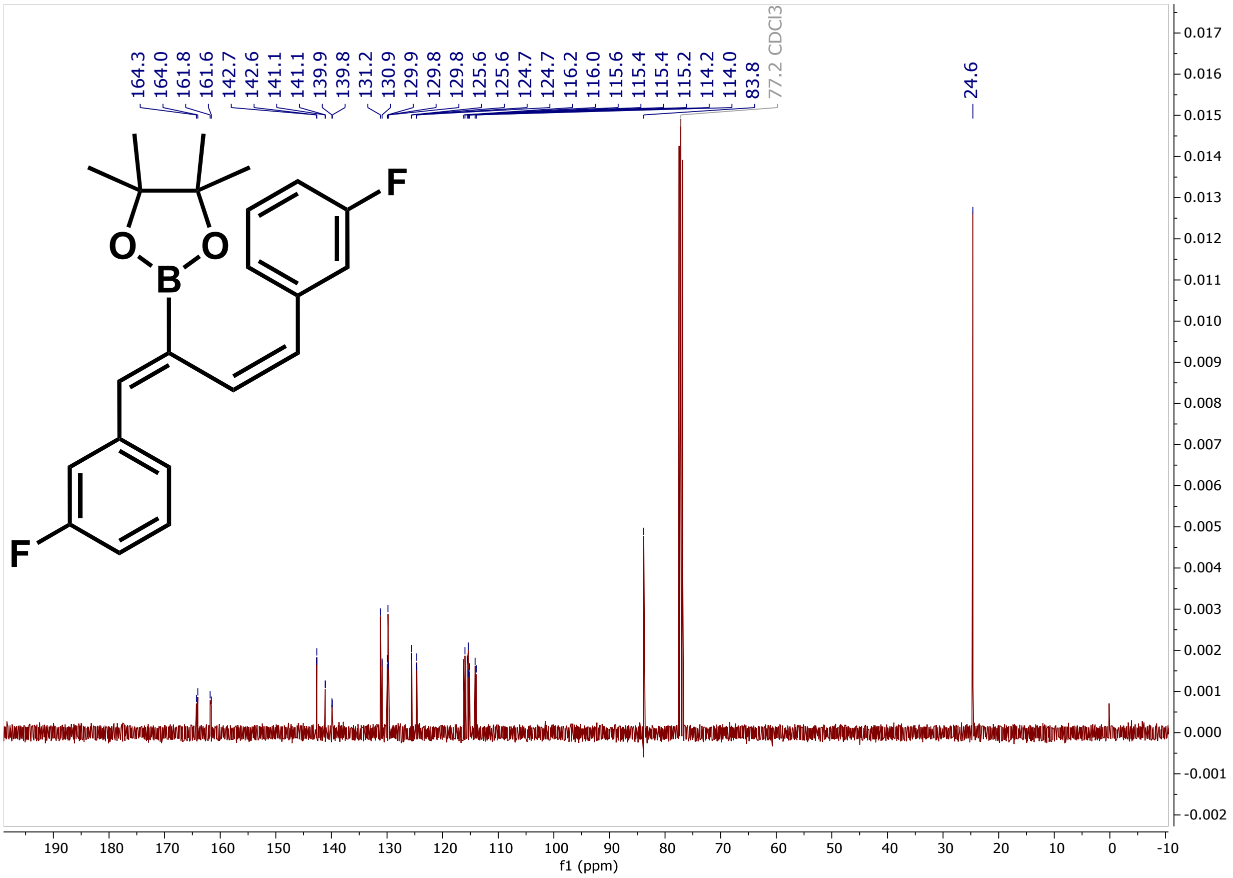

^{11}B NMR of **2m** ($CDCl_3$, 128 MHz)

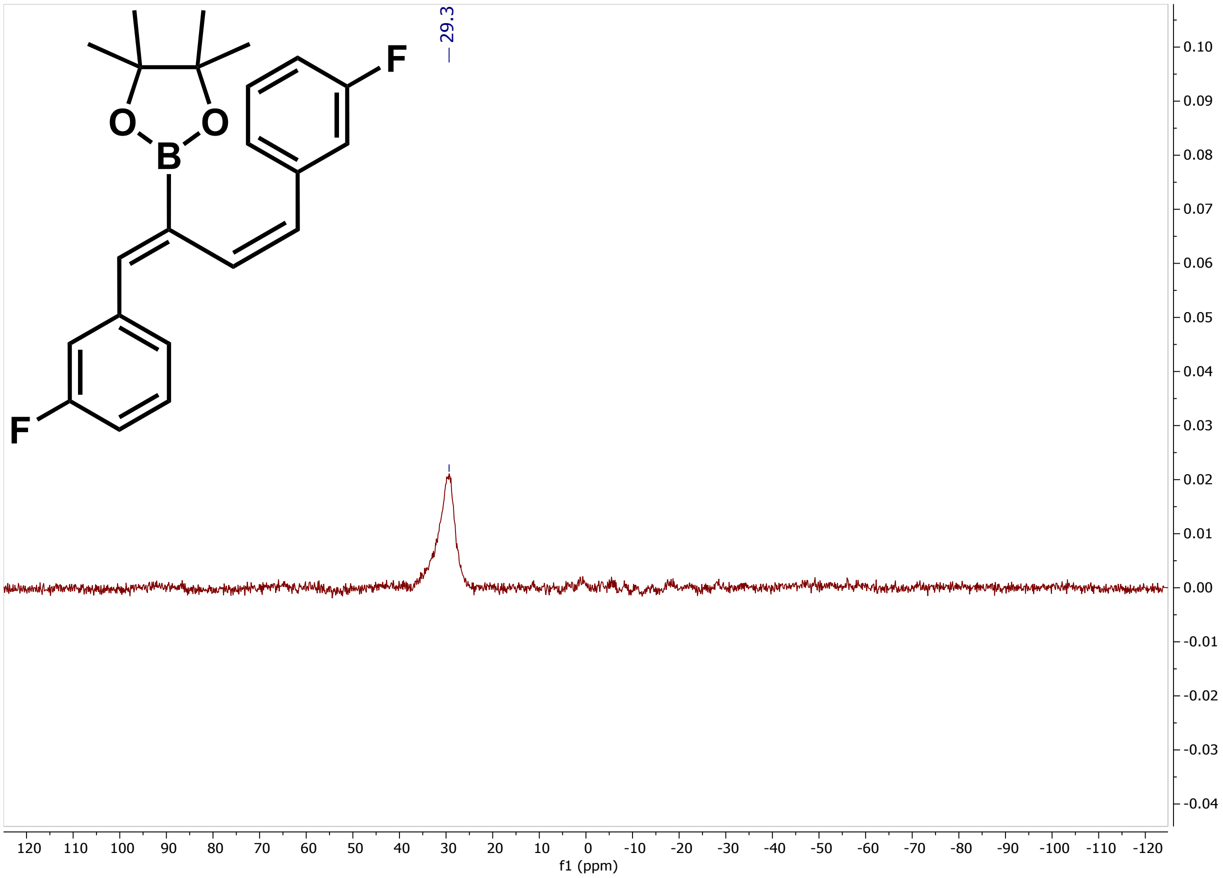

^{19}F NMR of **2m** ($CDCl_3$, 376 MHz)

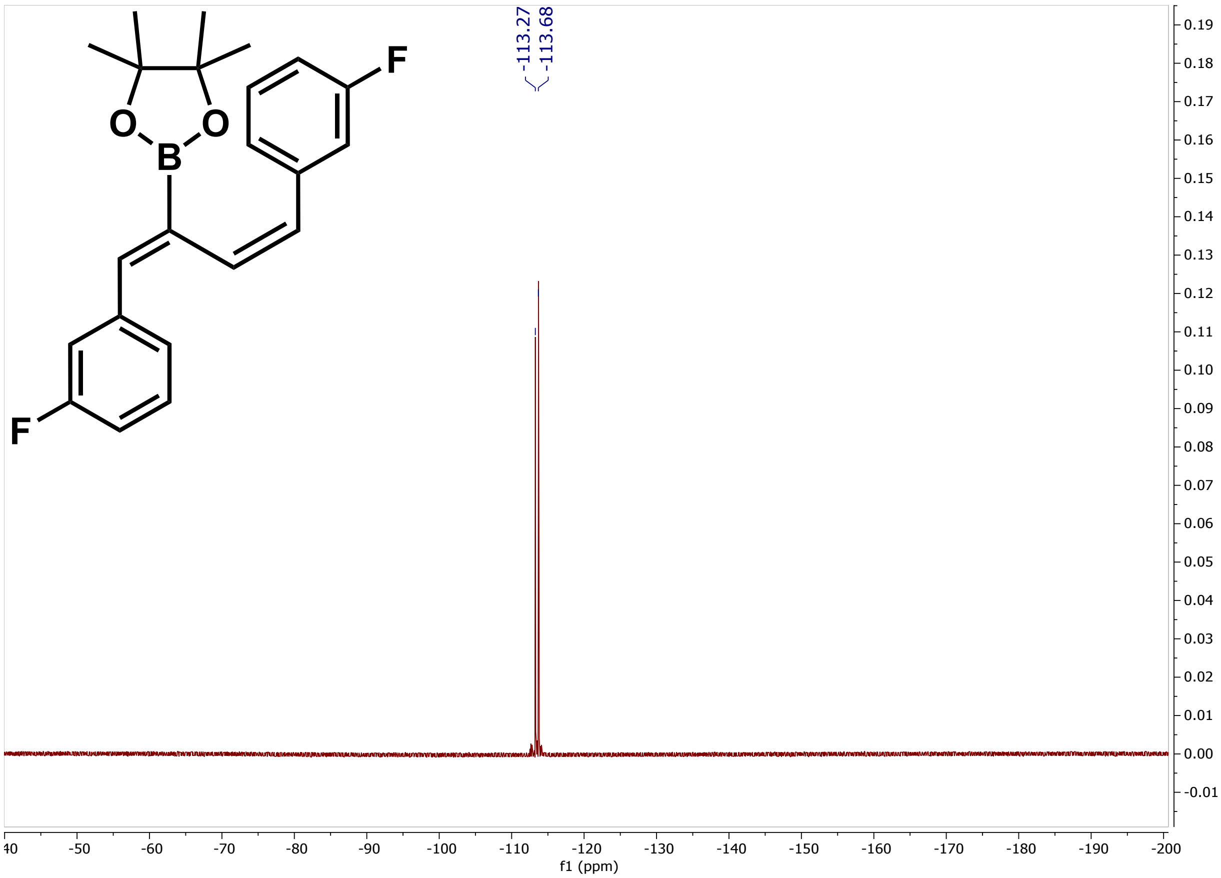

^{1}H NMR of **2n** ($CDCl_3$, 400 MHz)

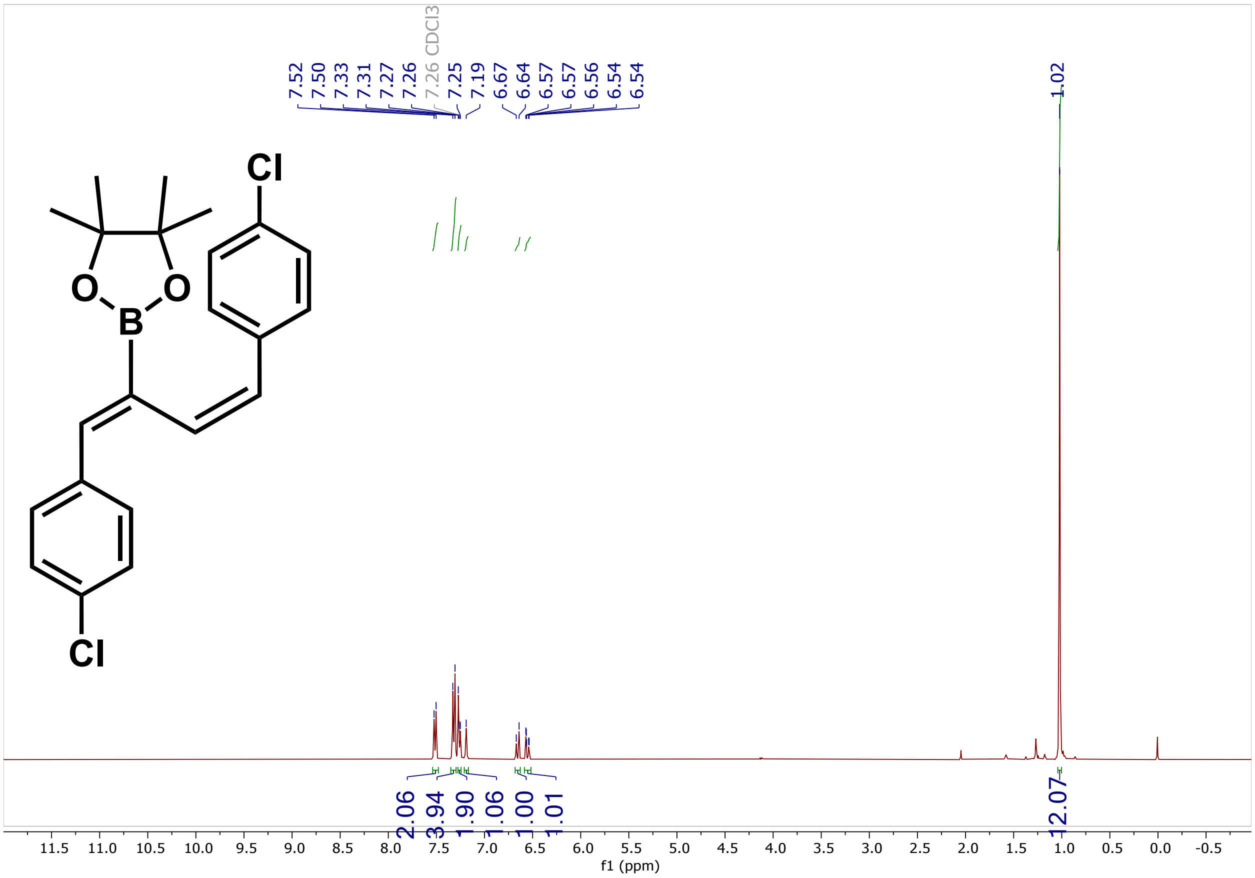

^{13}C NMR of **2n** ($CDCl_3$, 101 MHz)

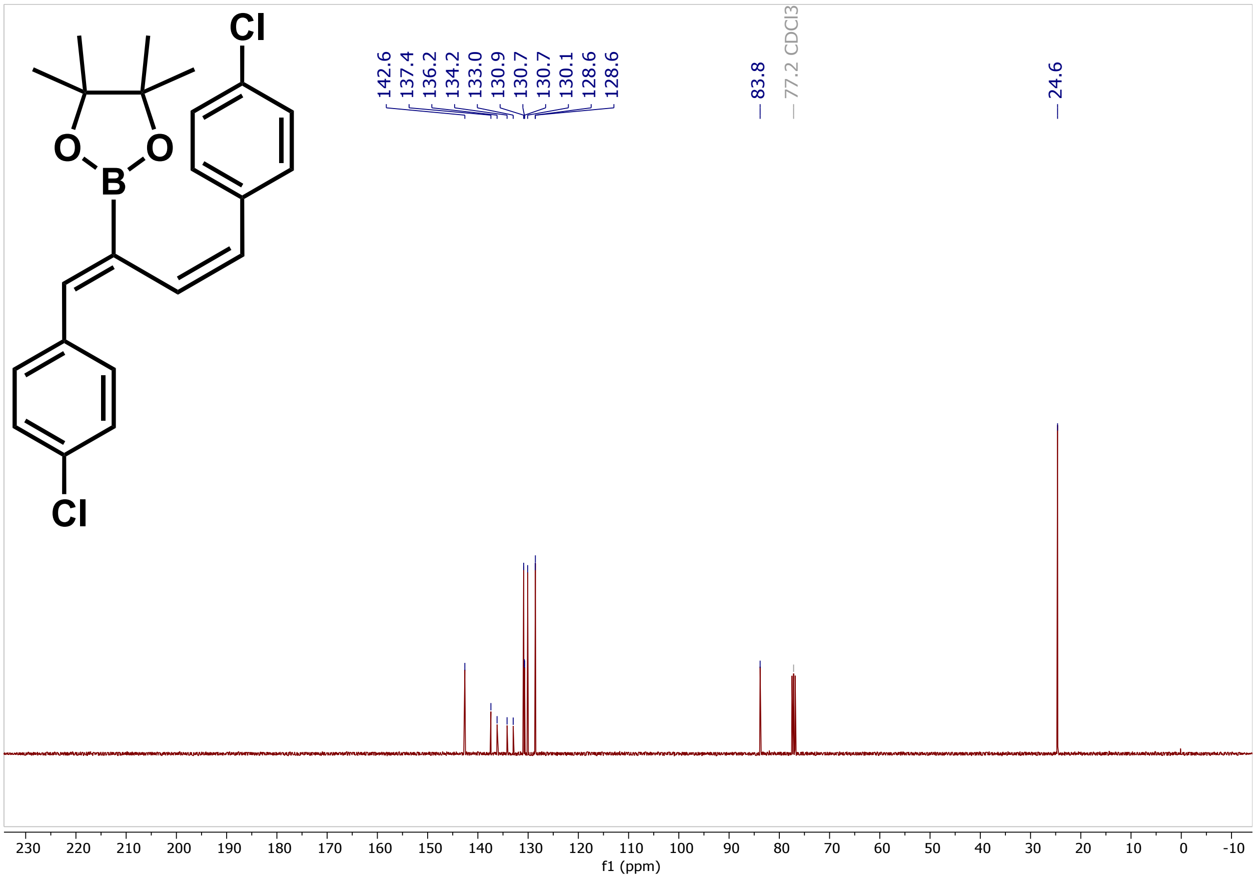

^{11}B NMR of **2n** ($CDCl_3$, 128 MHz)

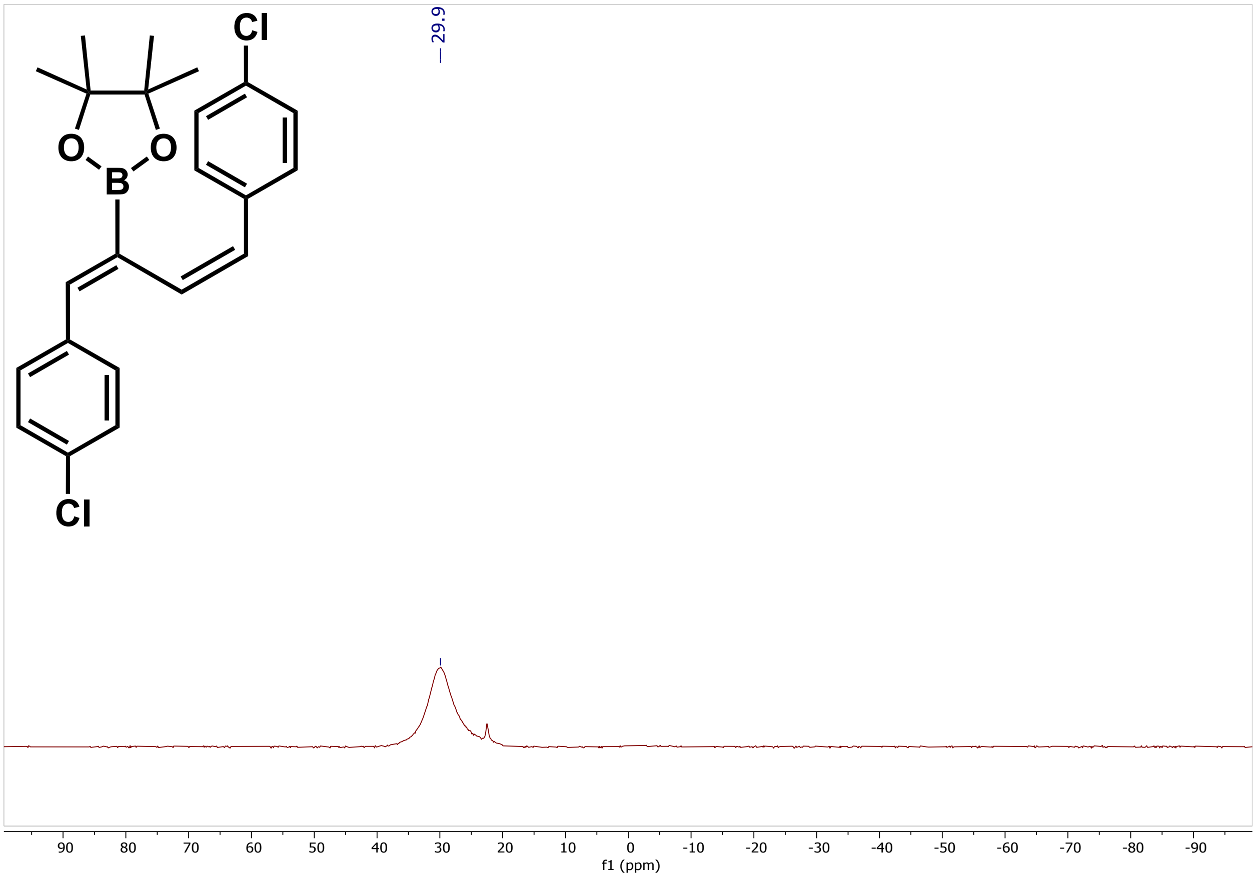

^{1}H NMR of **2o** ($CDCl_3$, 400 MHz)

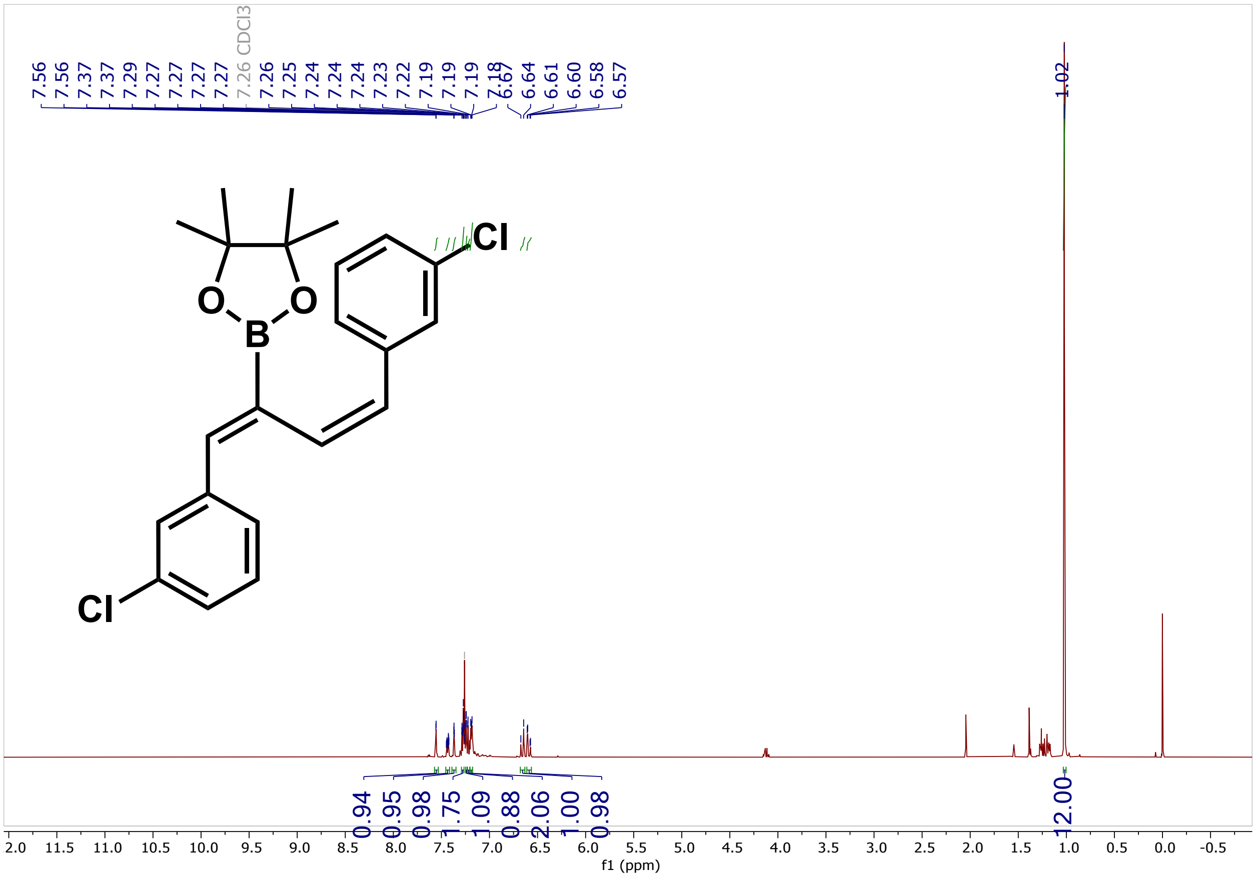

^{13}C NMR of **2o** ($CDCl_3$, 101 MHz)

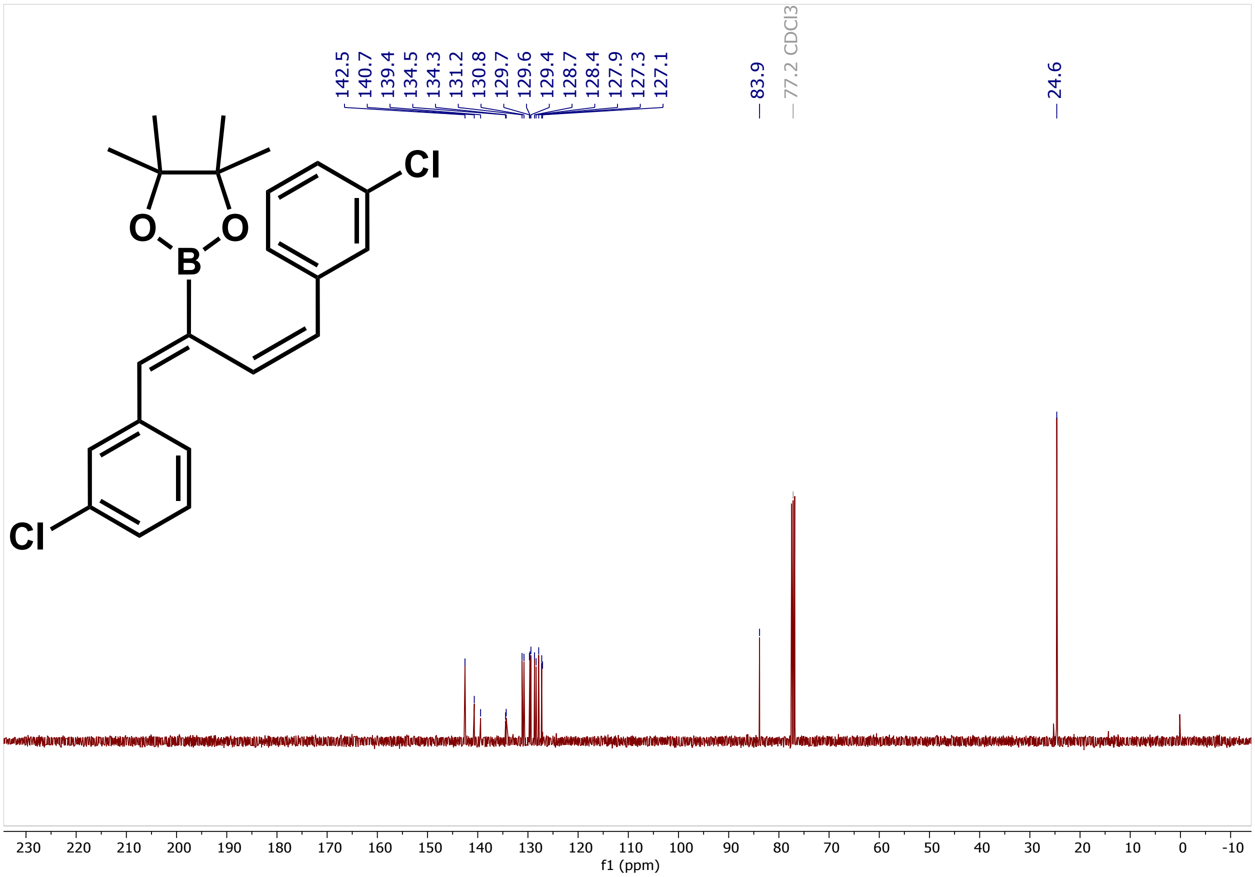

^{11}B NMR of **2o** ($CDCl_3$, 128 MHz)

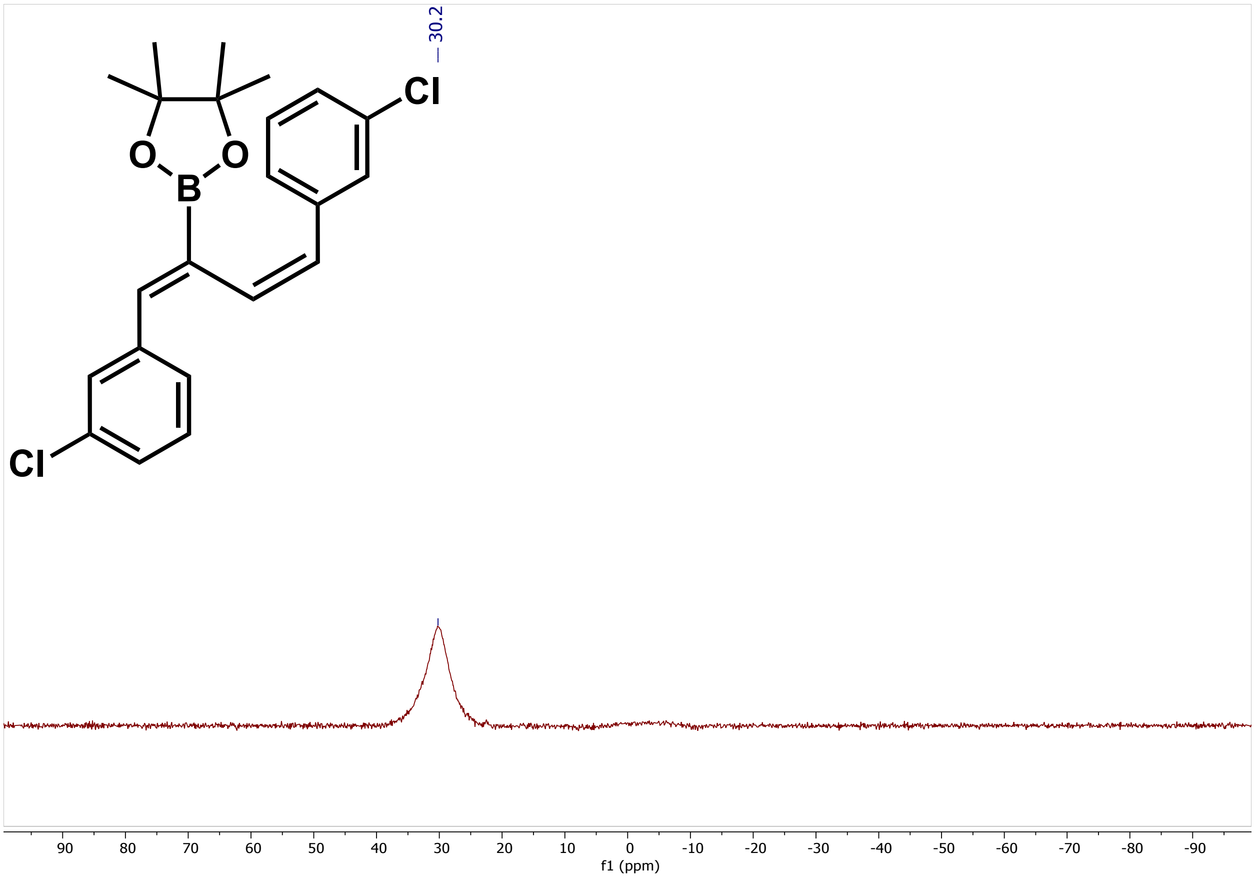

^{1}H NMR of **2p** ($CDCl_3$, 400 MHz)

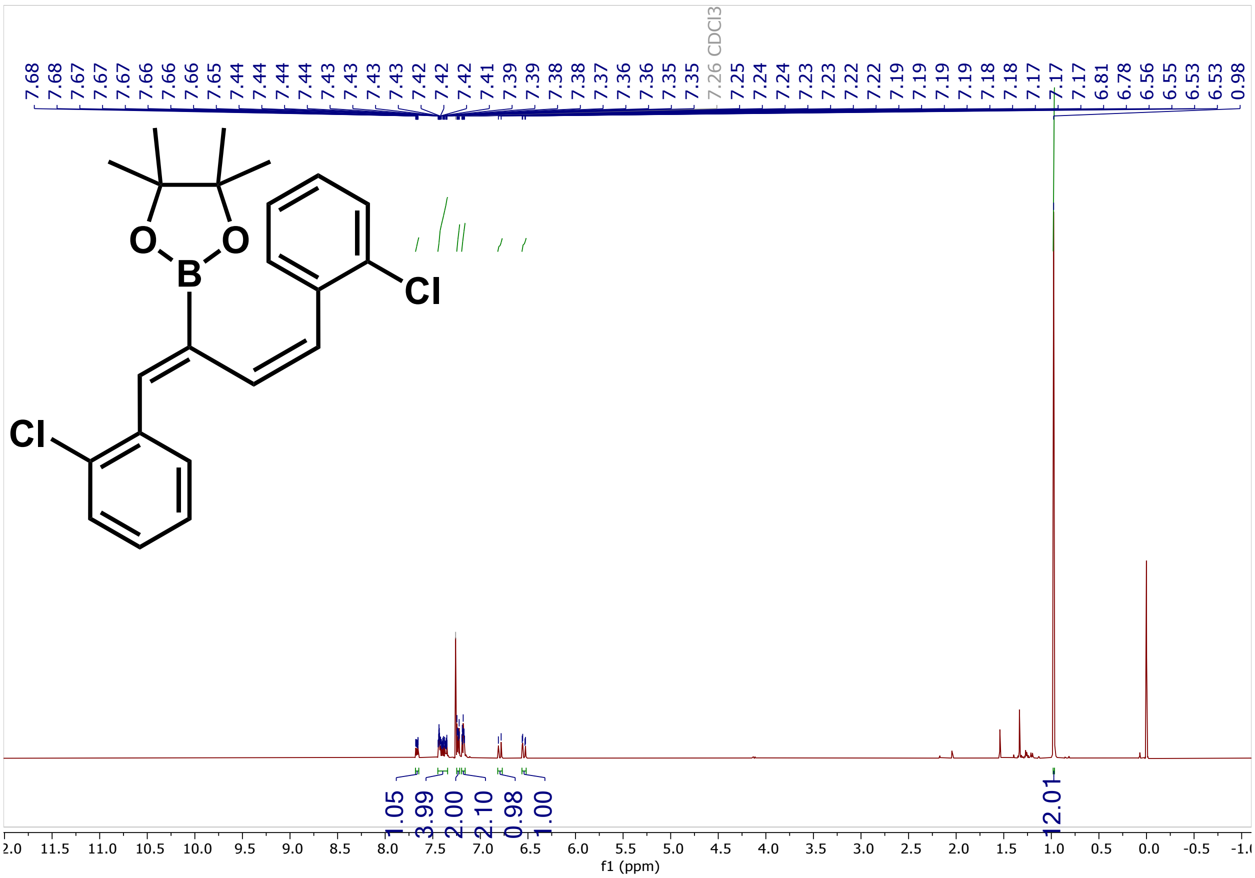

^{13}C NMR of **2p** ($CDCl_3$, 101 MHz)

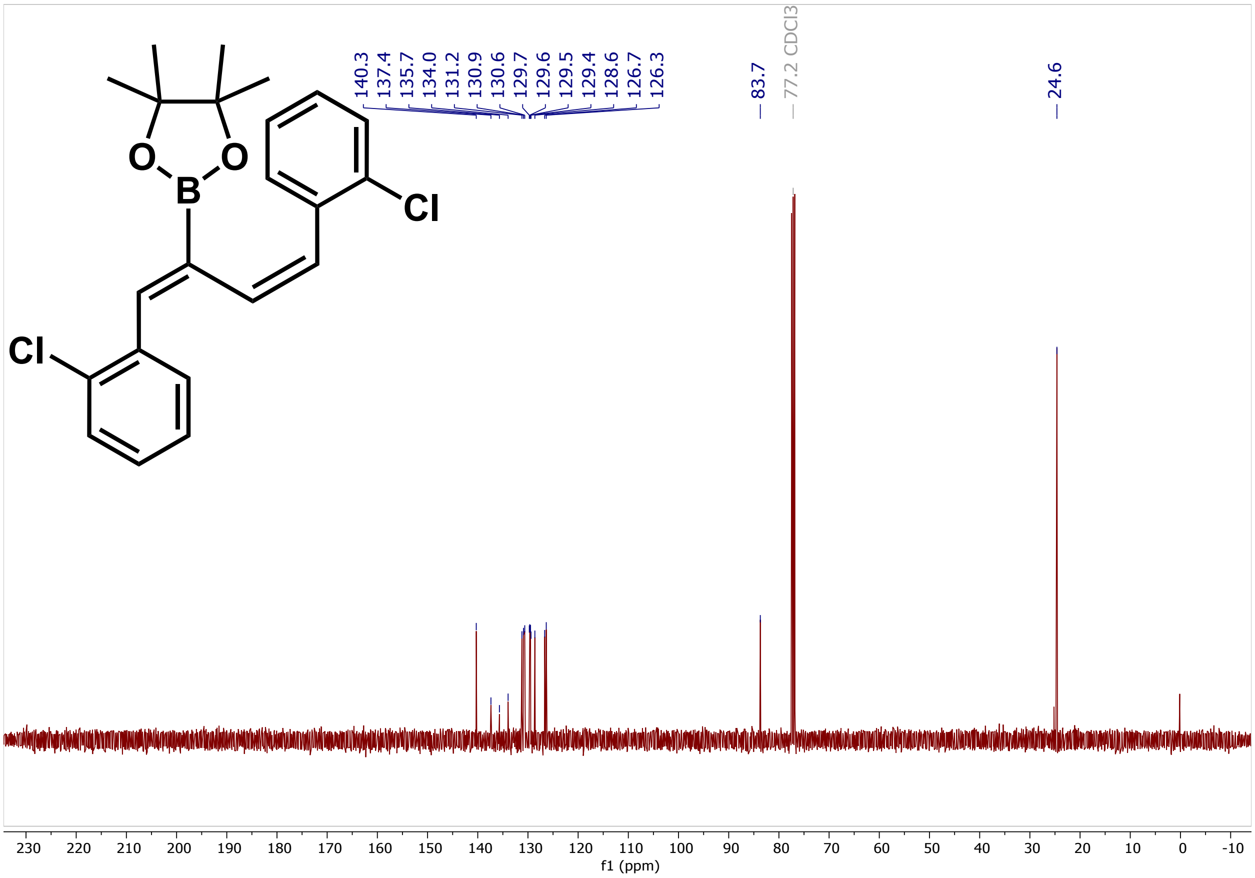

^{11}B NMR of **2p** ($CDCl_3$, 128 MHz)

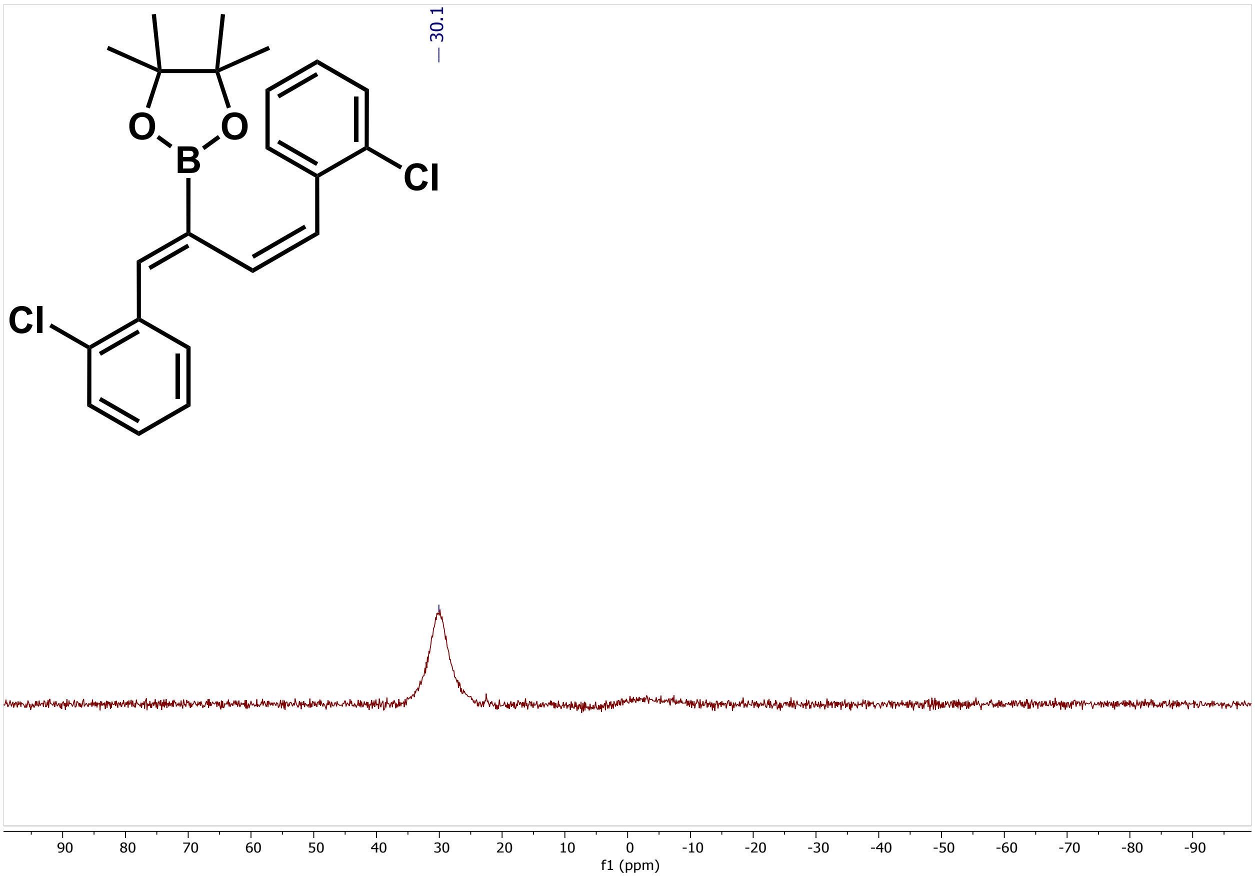

^{1}H NMR of **2q** ($CDCl_3$, 400 MHz)

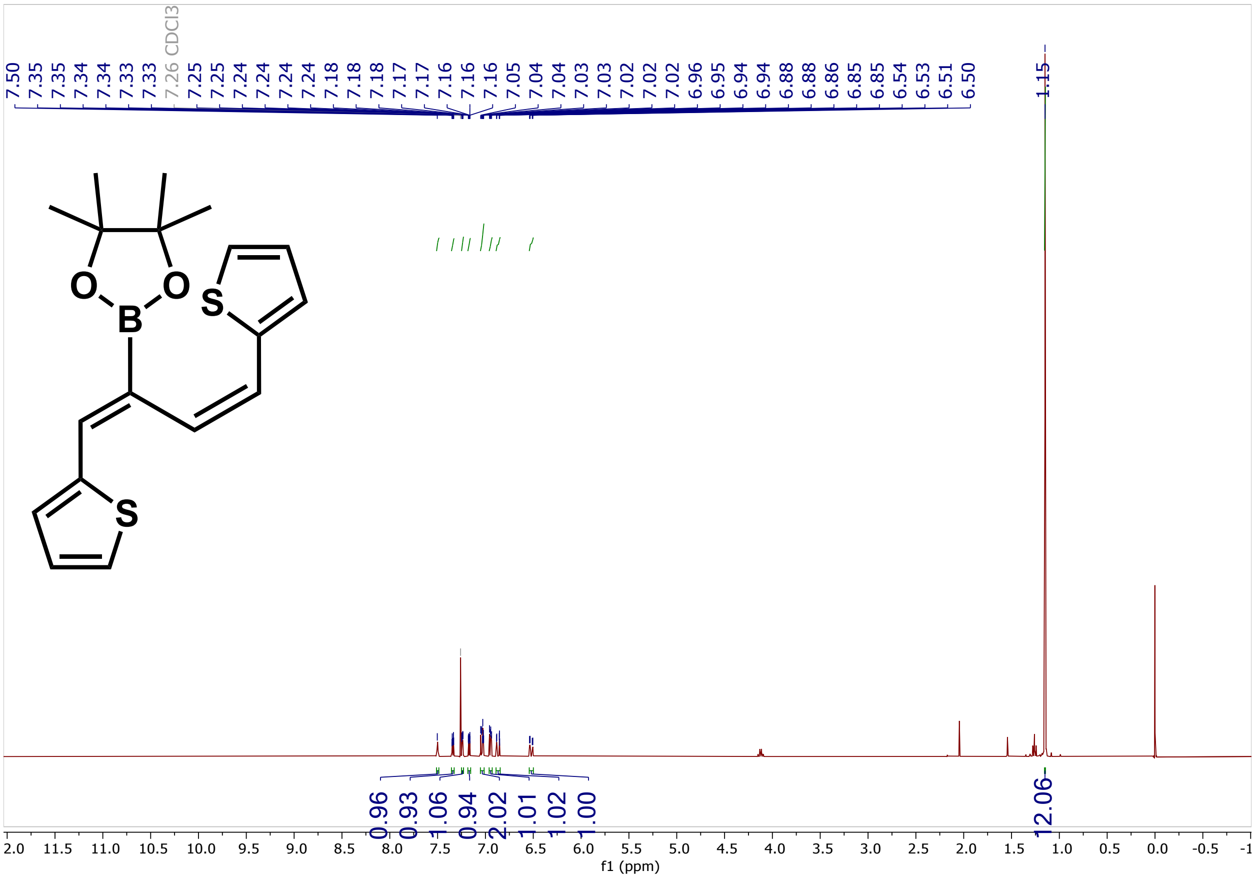

^{13}C NMR of **2q** ($CDCl_3$, 101 MHz)

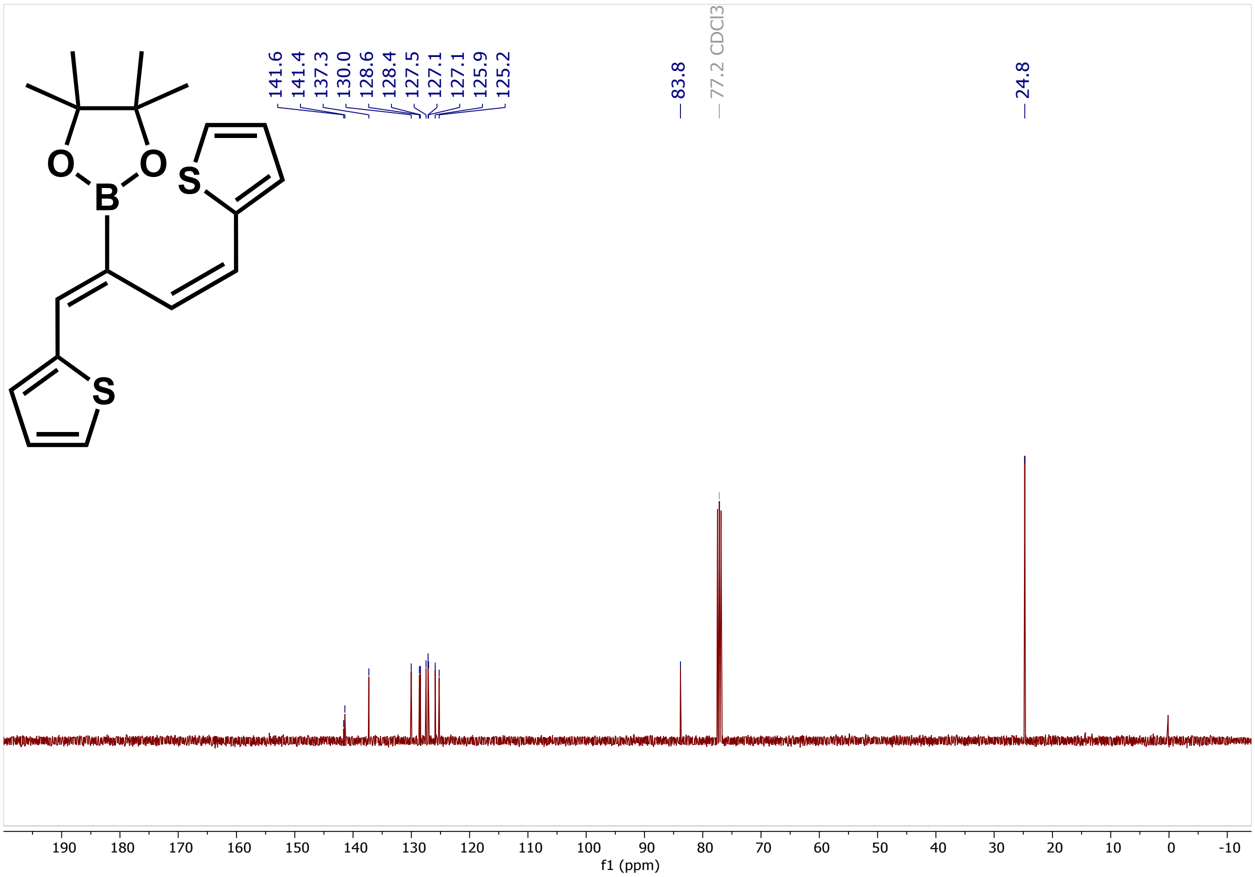

^{11}B NMR of **2q** ($CDCl_3$, 128 MHz)

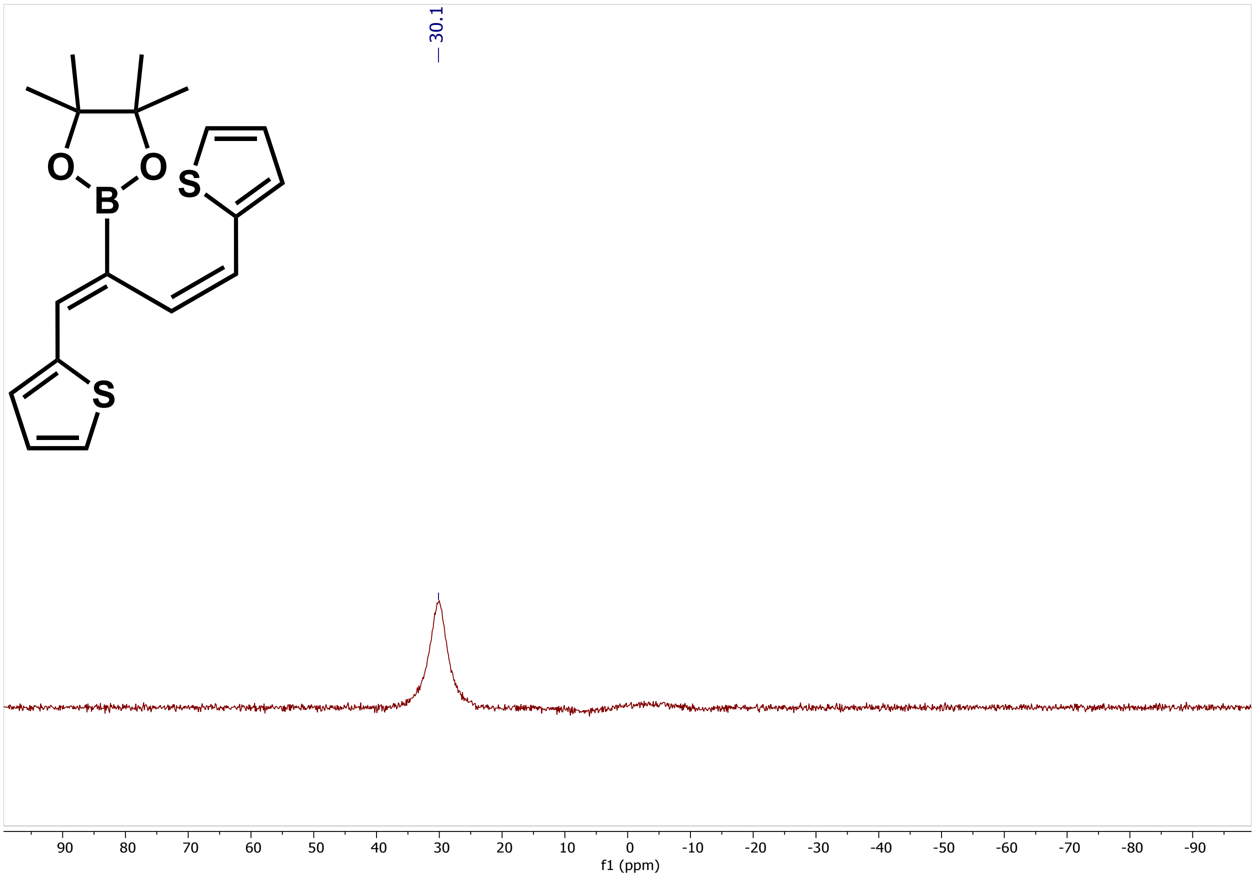

^{1}H NMR of **2r** ($CDCl_3$, 400 MHz)

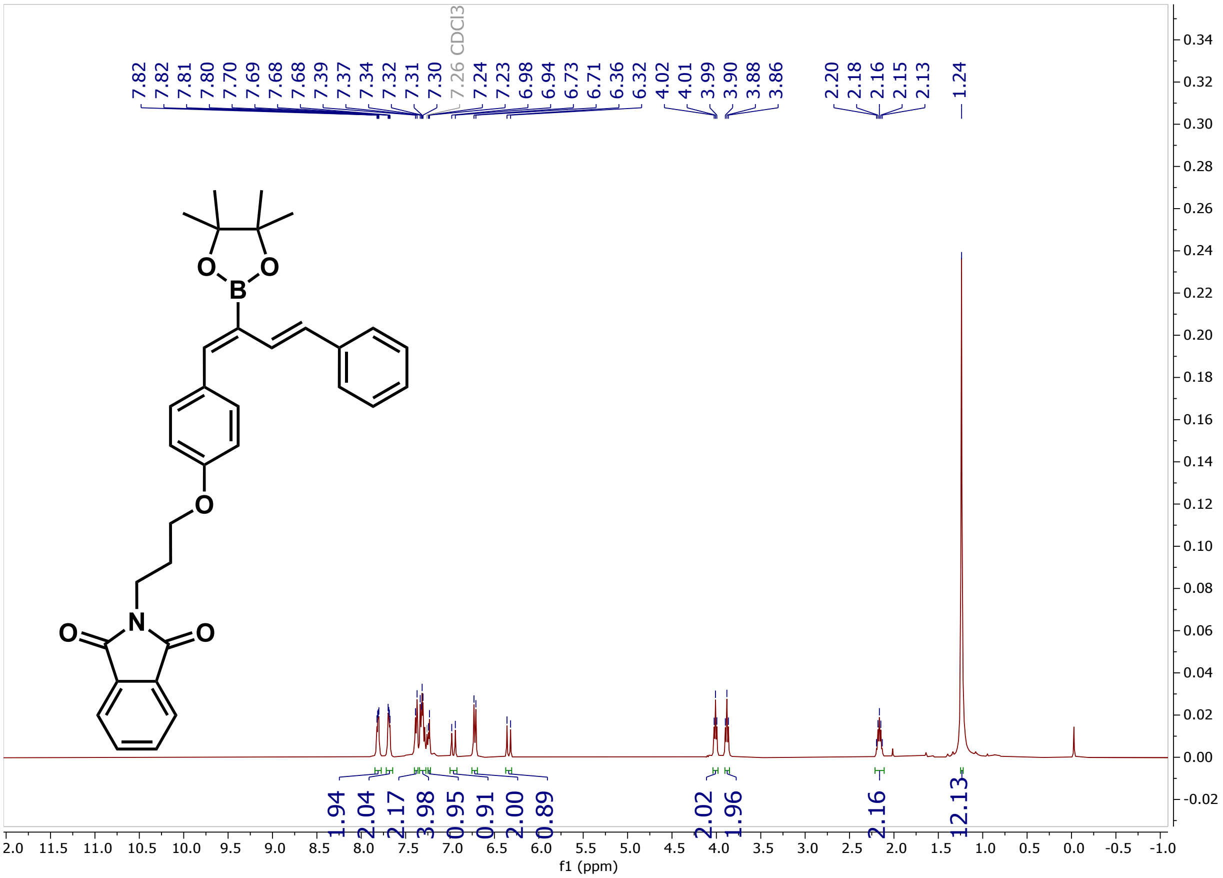

^{13}C NMR of **2r** ($CDCl_3$, 101 MHz)

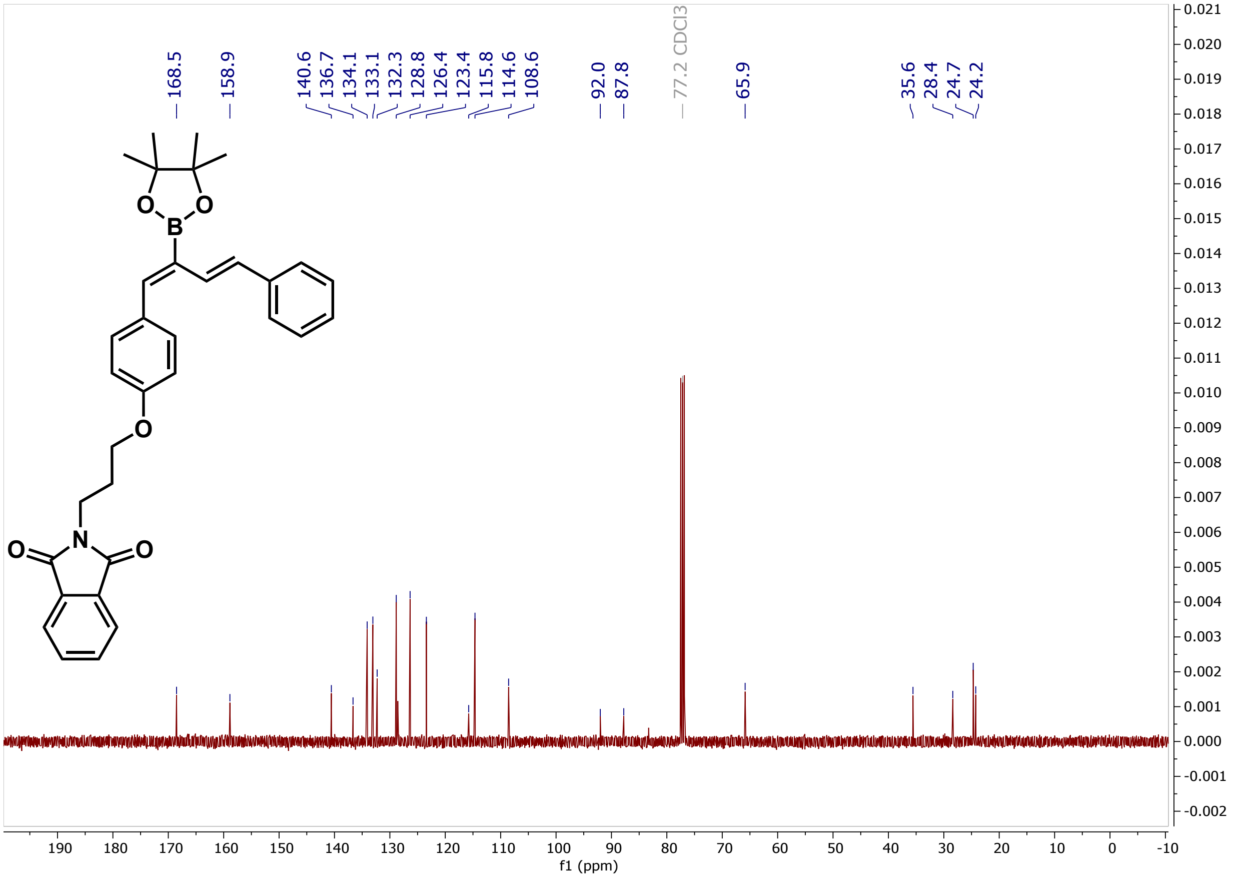

^{11}B NMR of **2r** ($CDCl_3$, 128 MHz)

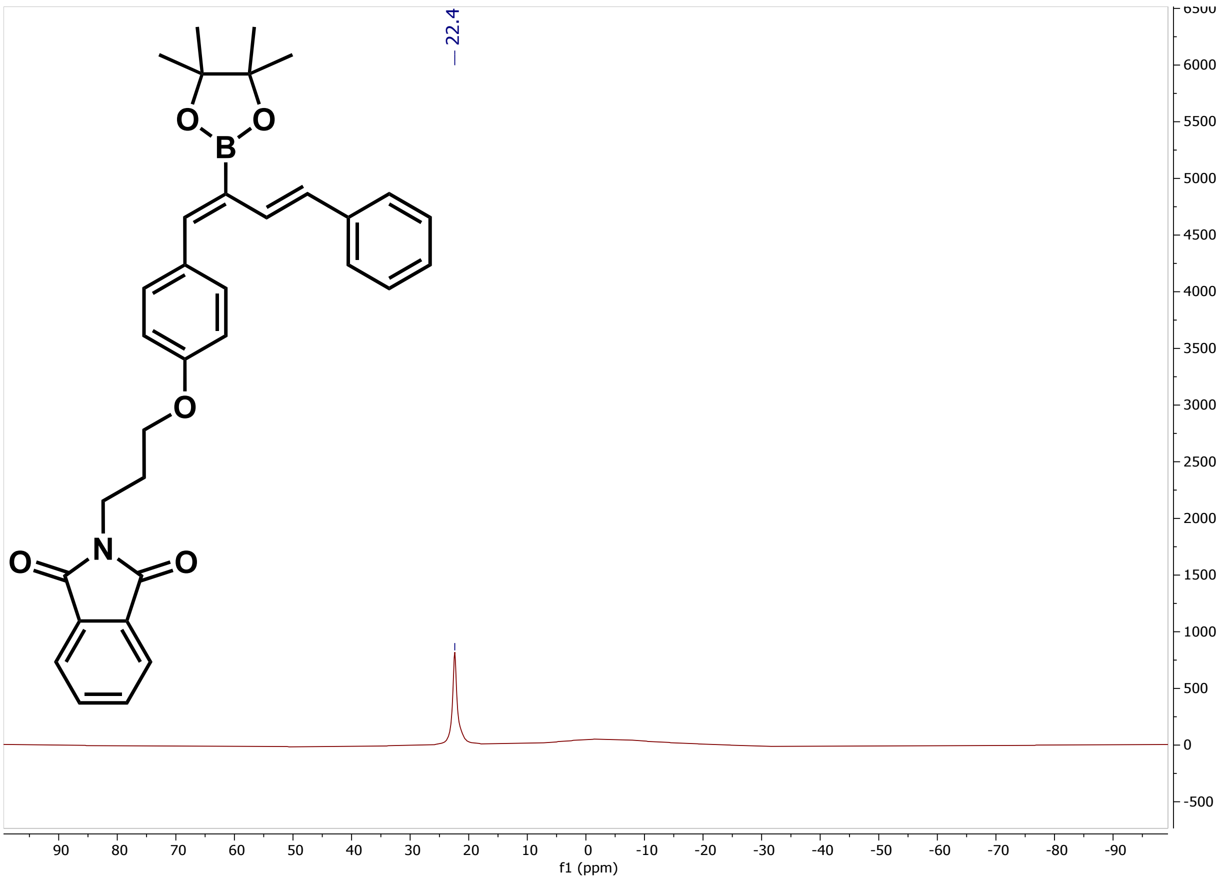

^{1}H NMR of **2s** ($CDCl_3$, 400 MHz)

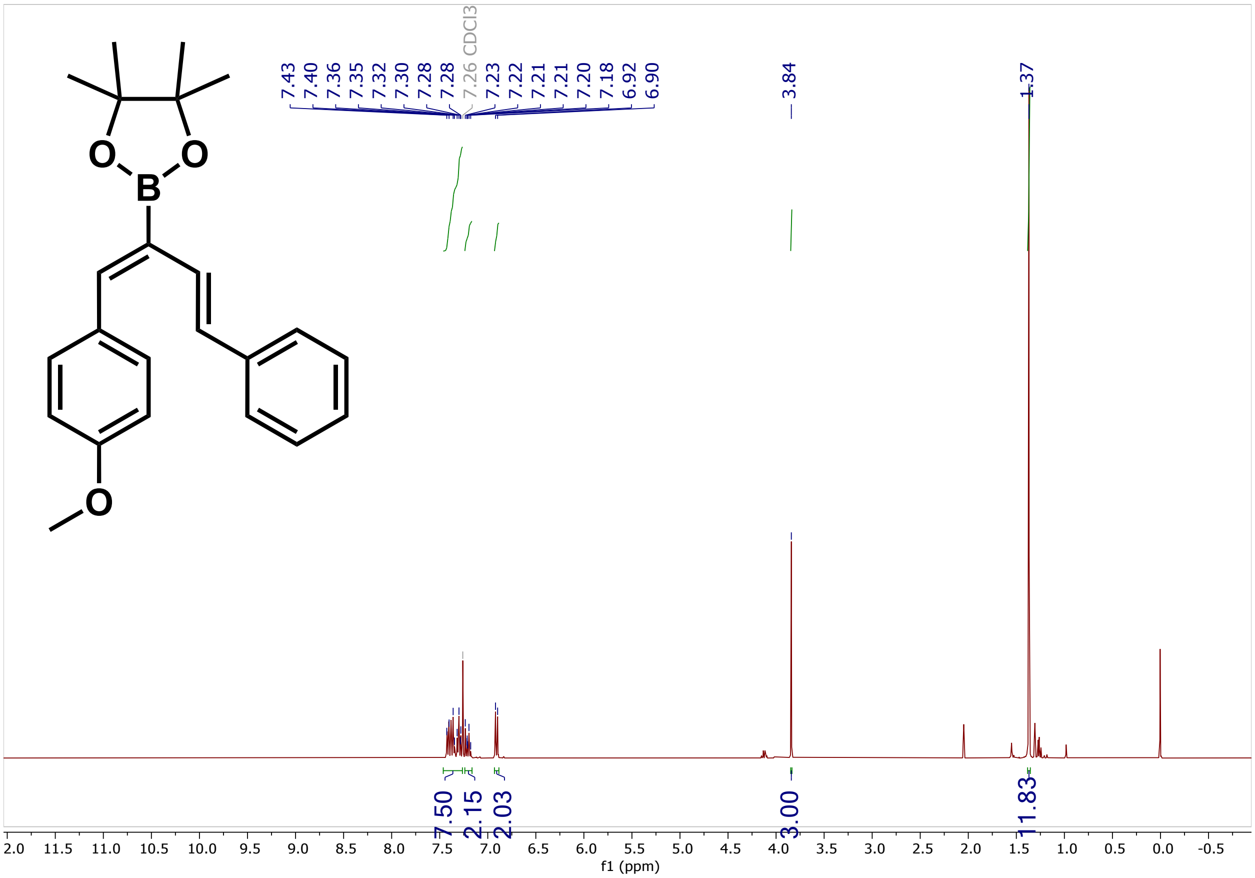

^{13}C NMR of **2s** ($CDCl_3$, 101 MHz)

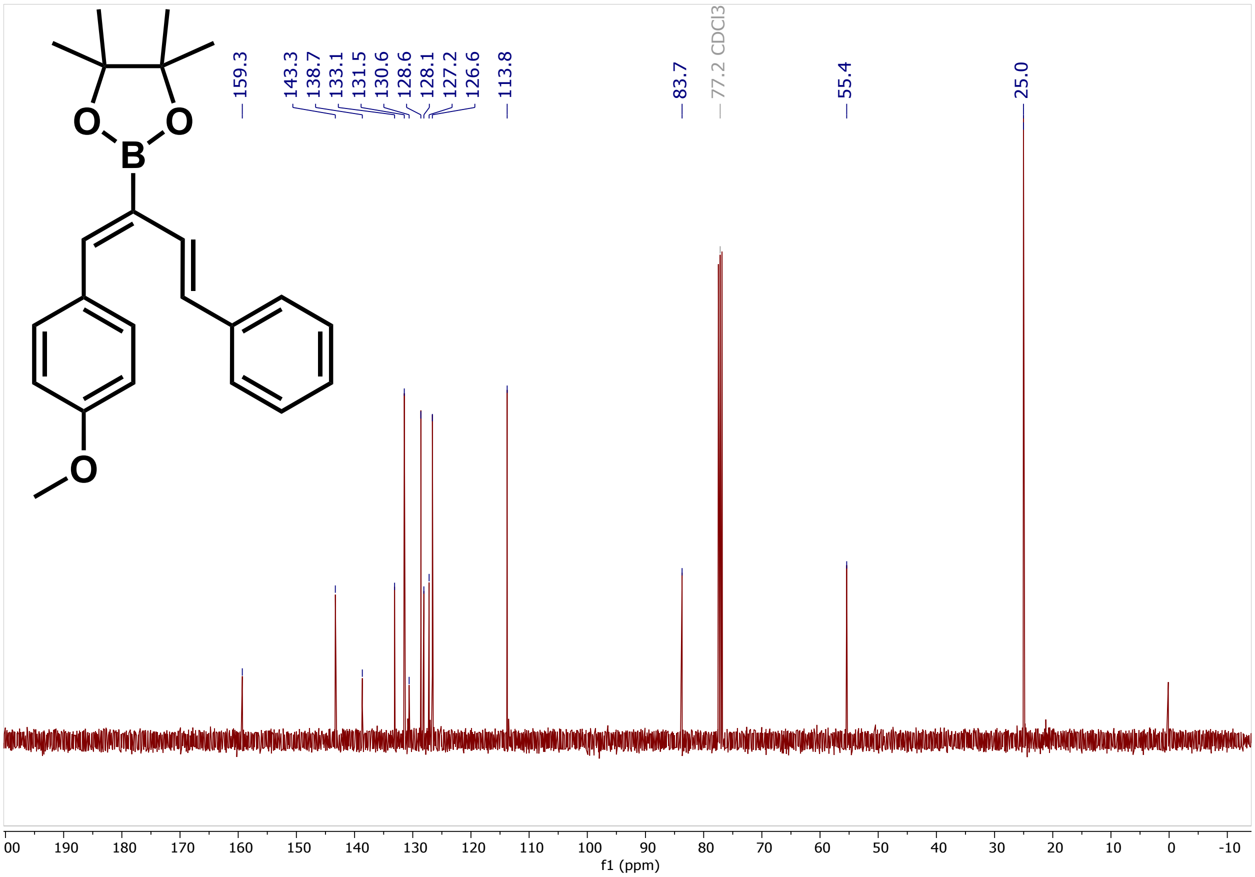

^{11}B NMR of **2s** ($CDCl_3$, 128 MHz)

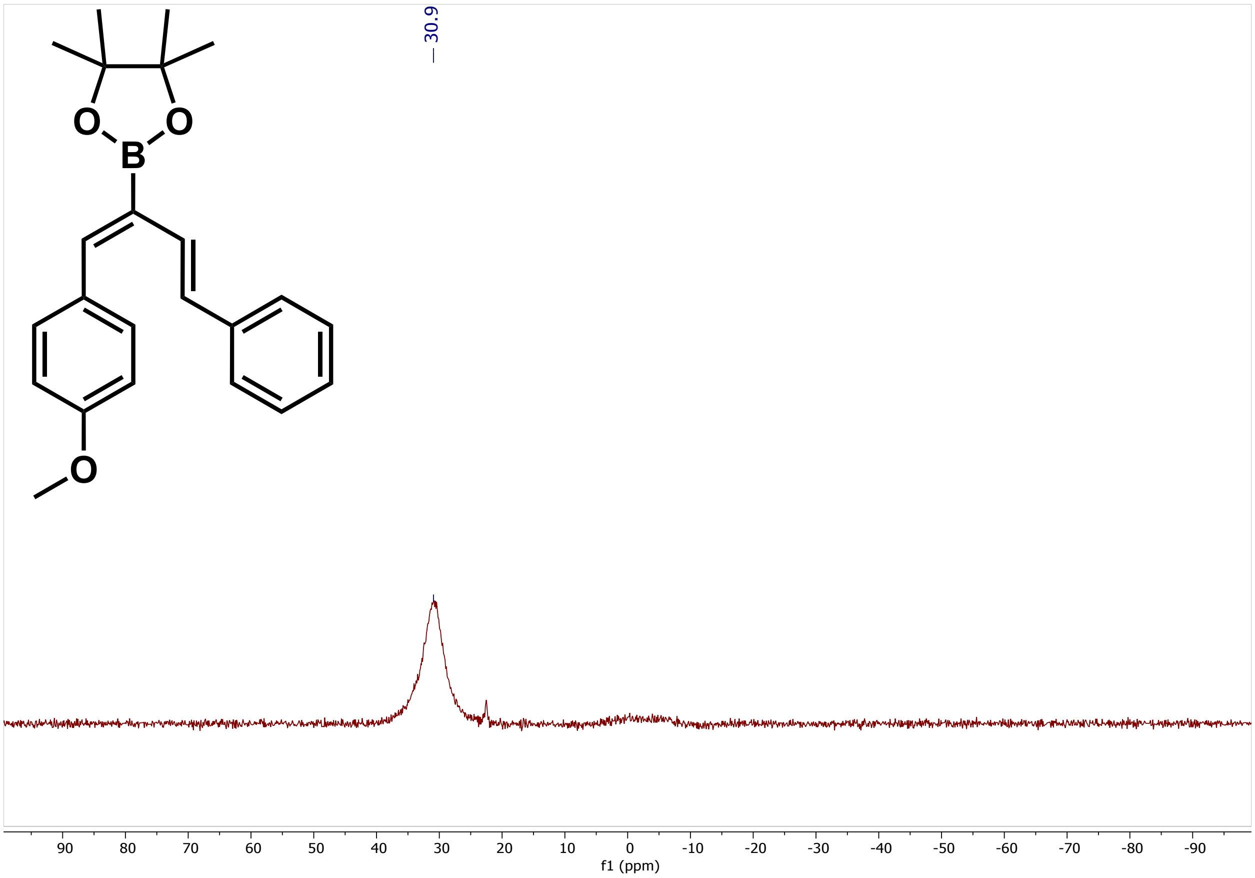

^{1}H NMR of **2t** ($CDCl_3$, 400 MHz)

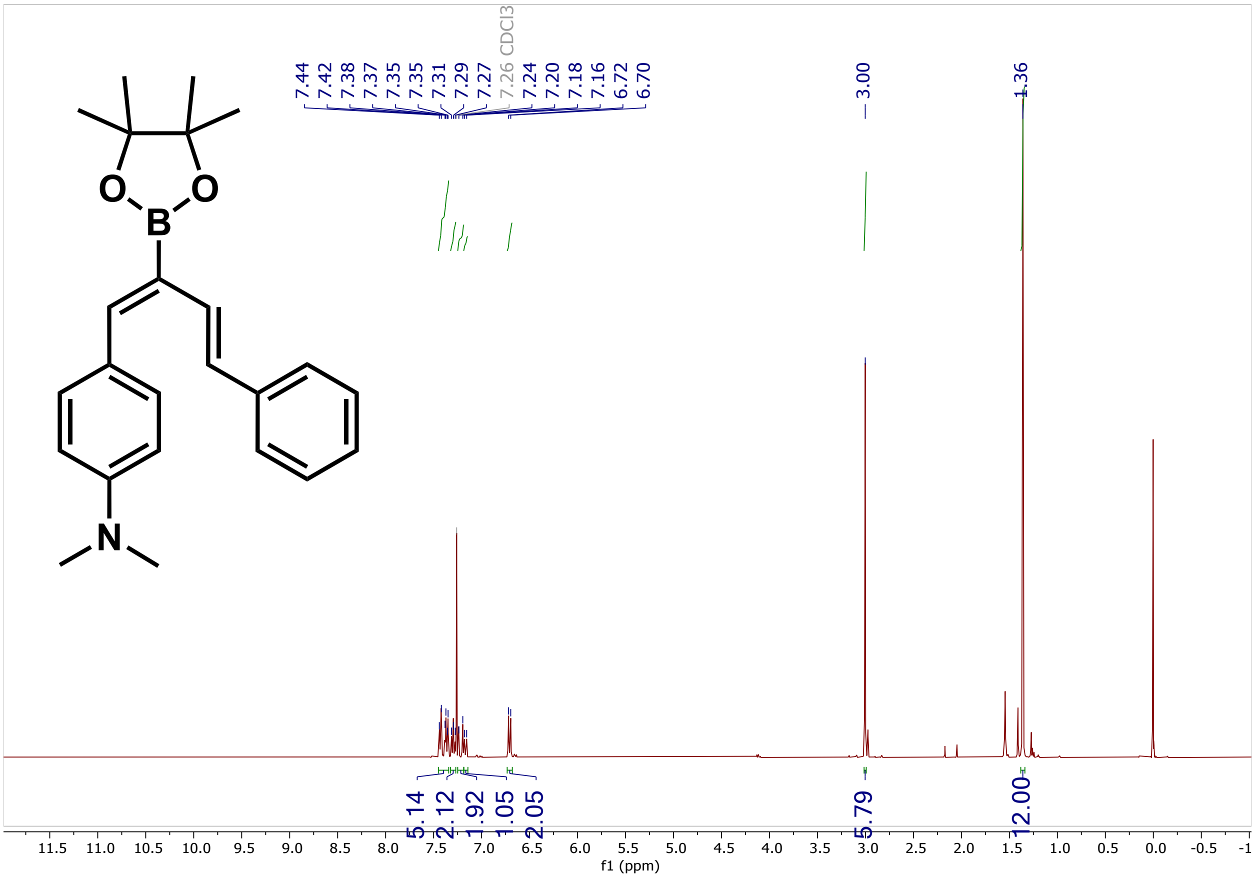

^{13}C NMR of **2t** ($CDCl_3$, 101 MHz)

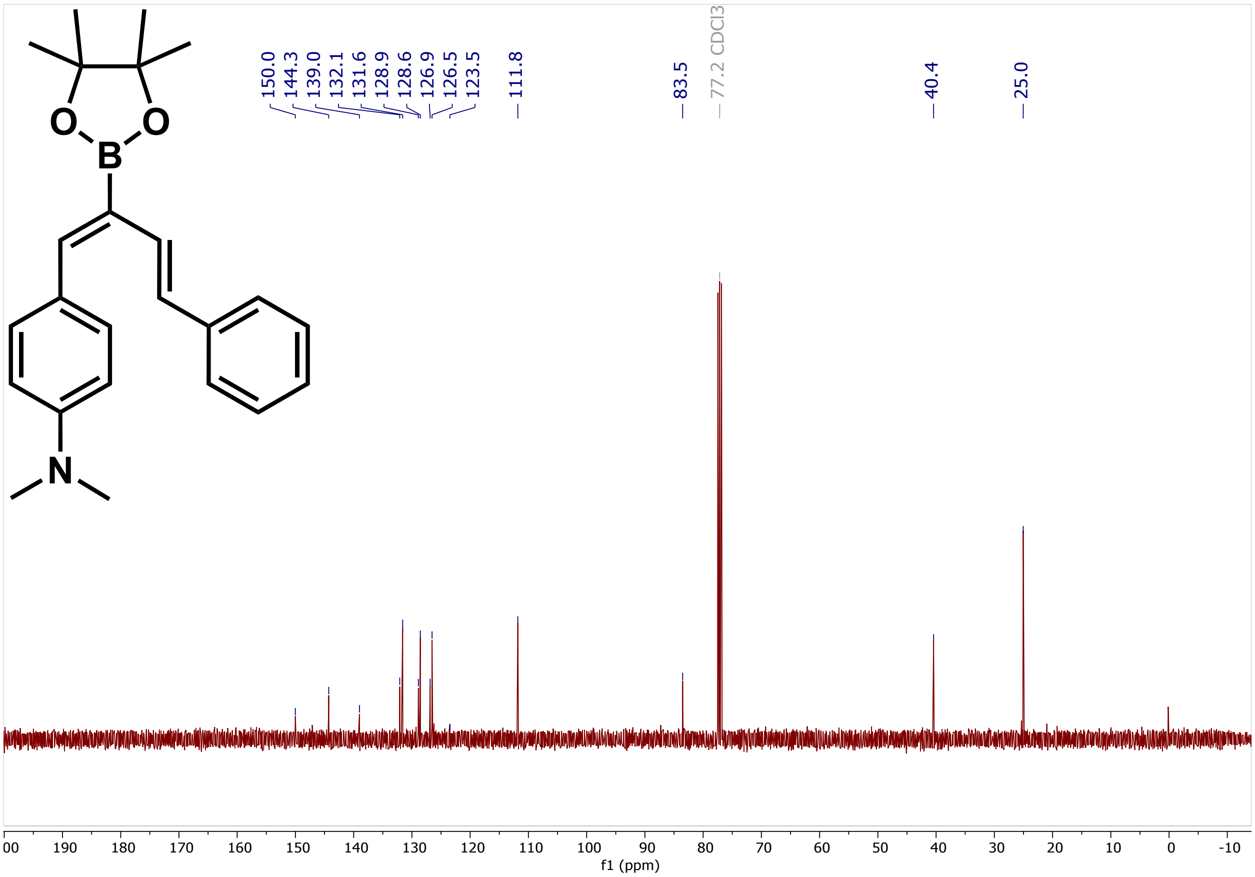

^{11}B NMR of **2t** ($CDCl_3$, 128 MHz)

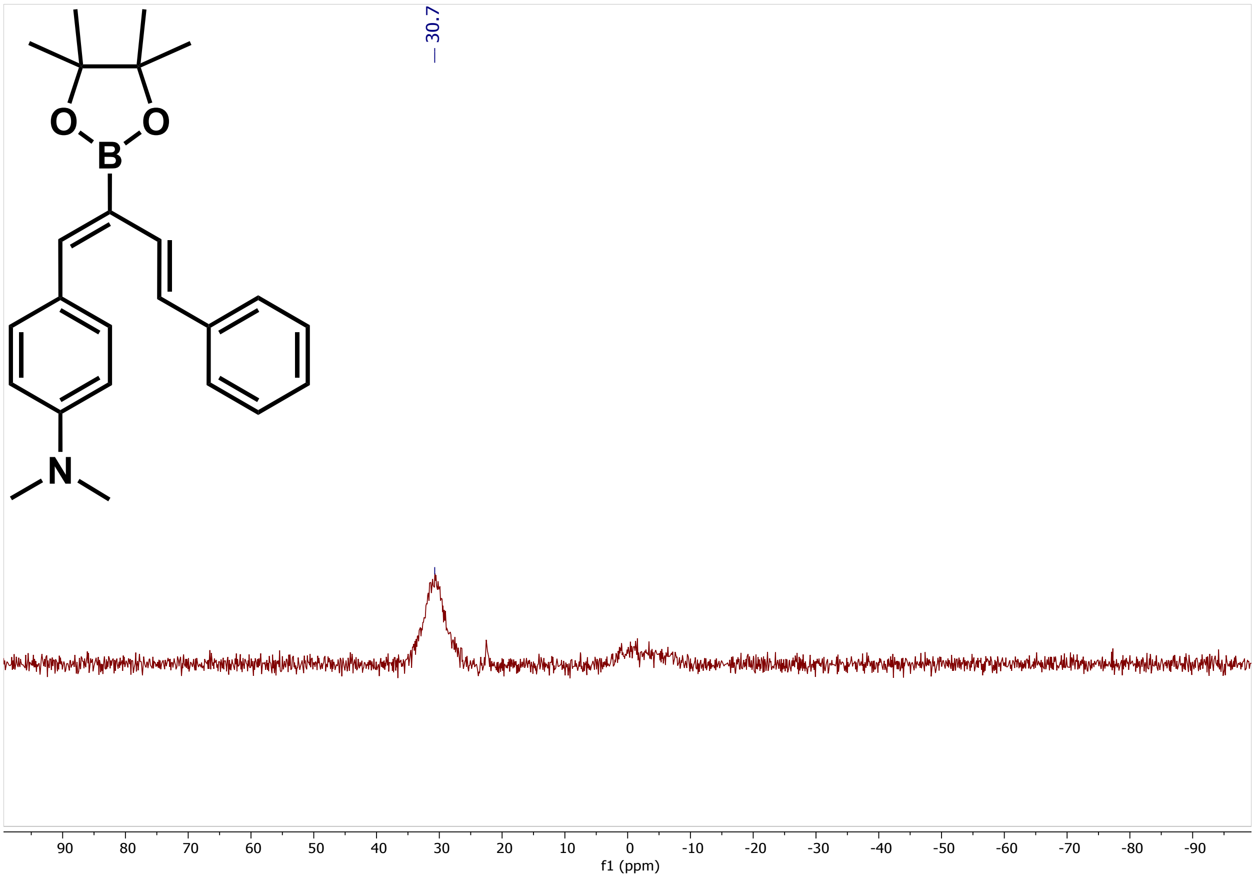

^{1}H NMR of **2u** ($CDCl_3$, 400 MHz)

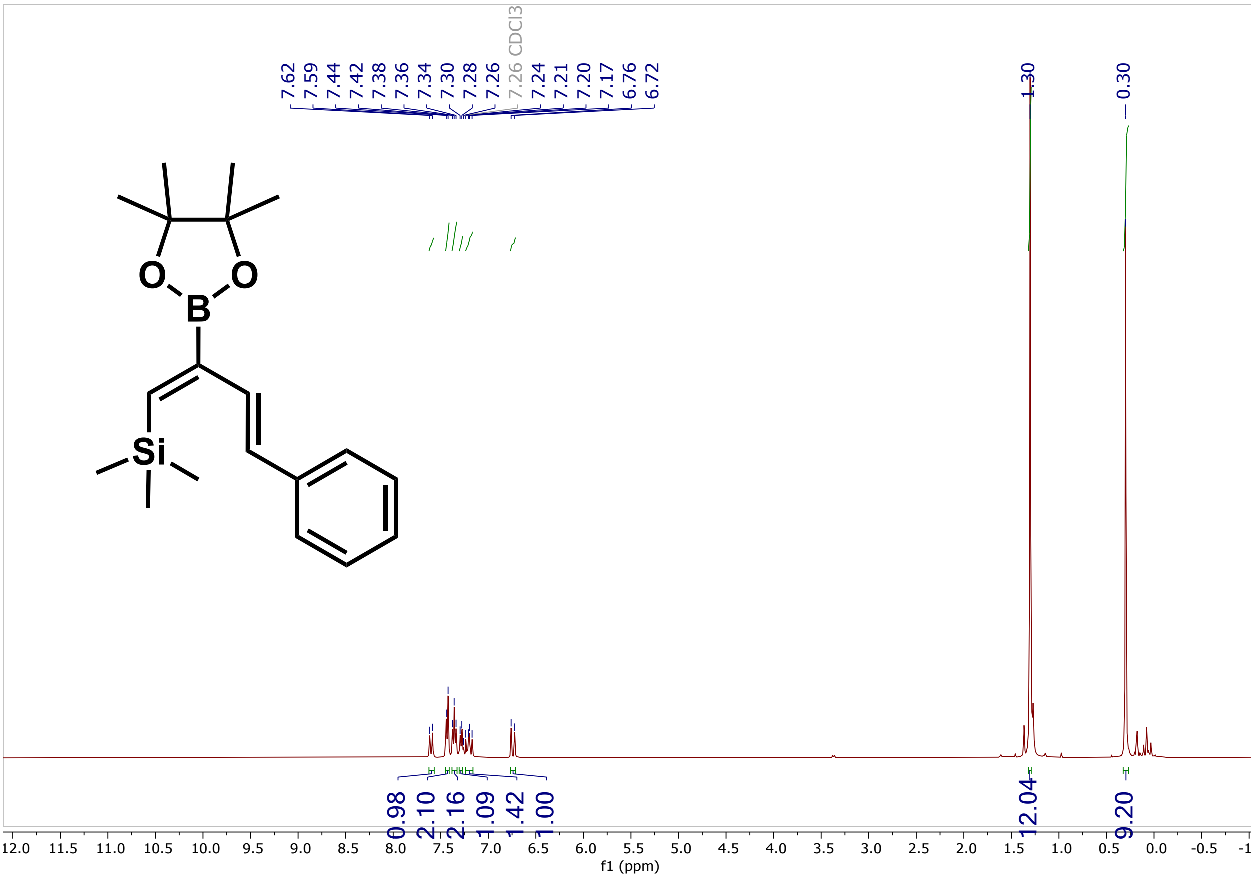

^{13}C NMR of **2u** ($CDCl_3$, 101 MHz)

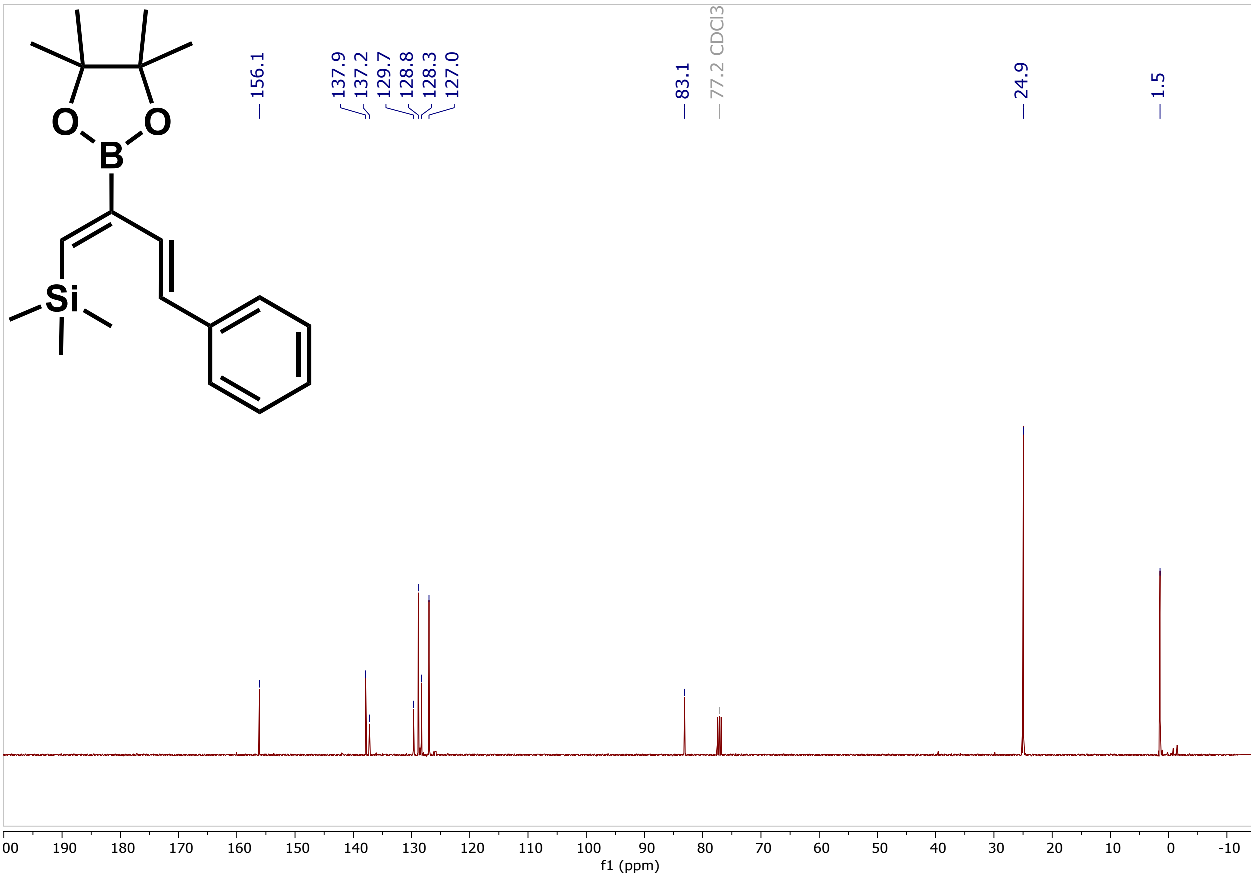

^{11}B NMR of **2u** ($CDCl_3$, 128 MHz)

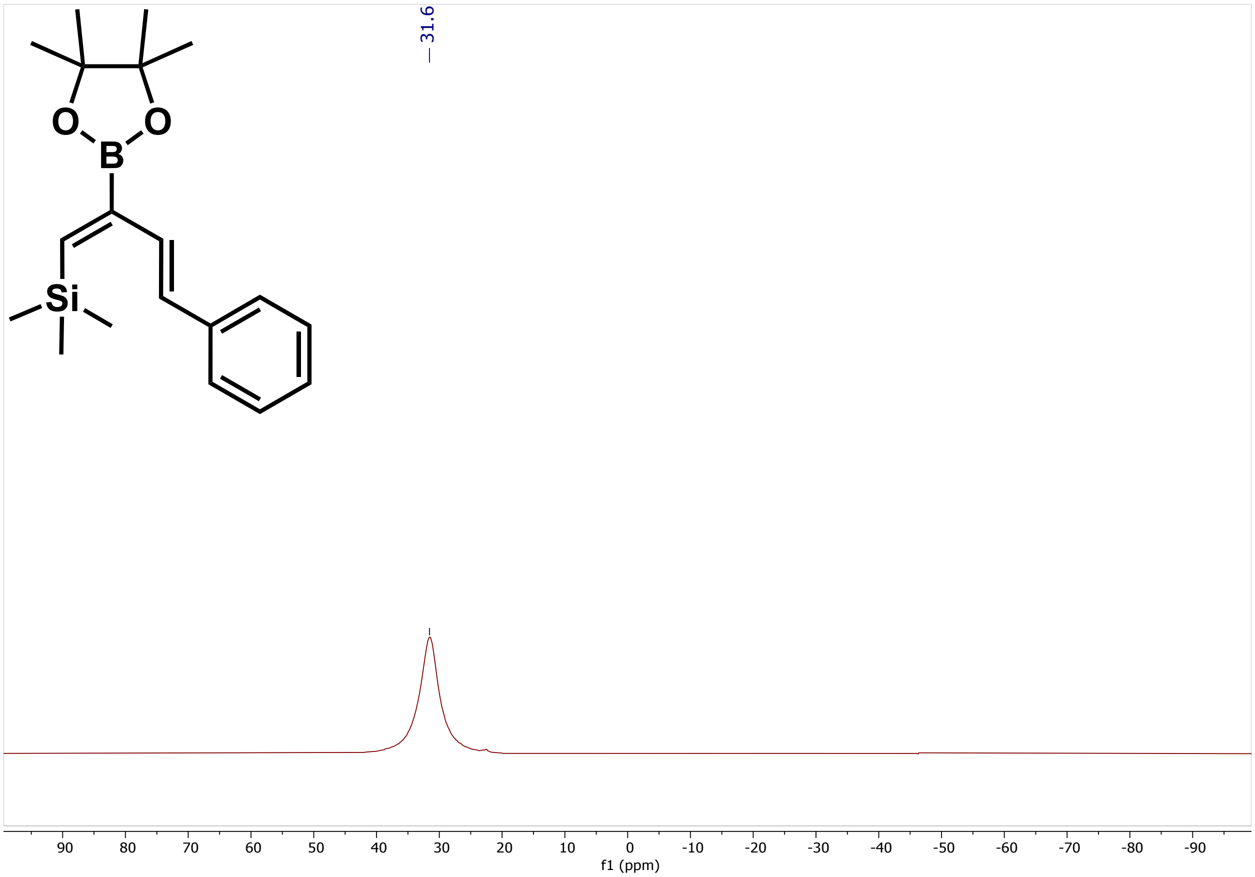

^{1}H NMR of **2v** ($CDCl_3$, 400 MHz)

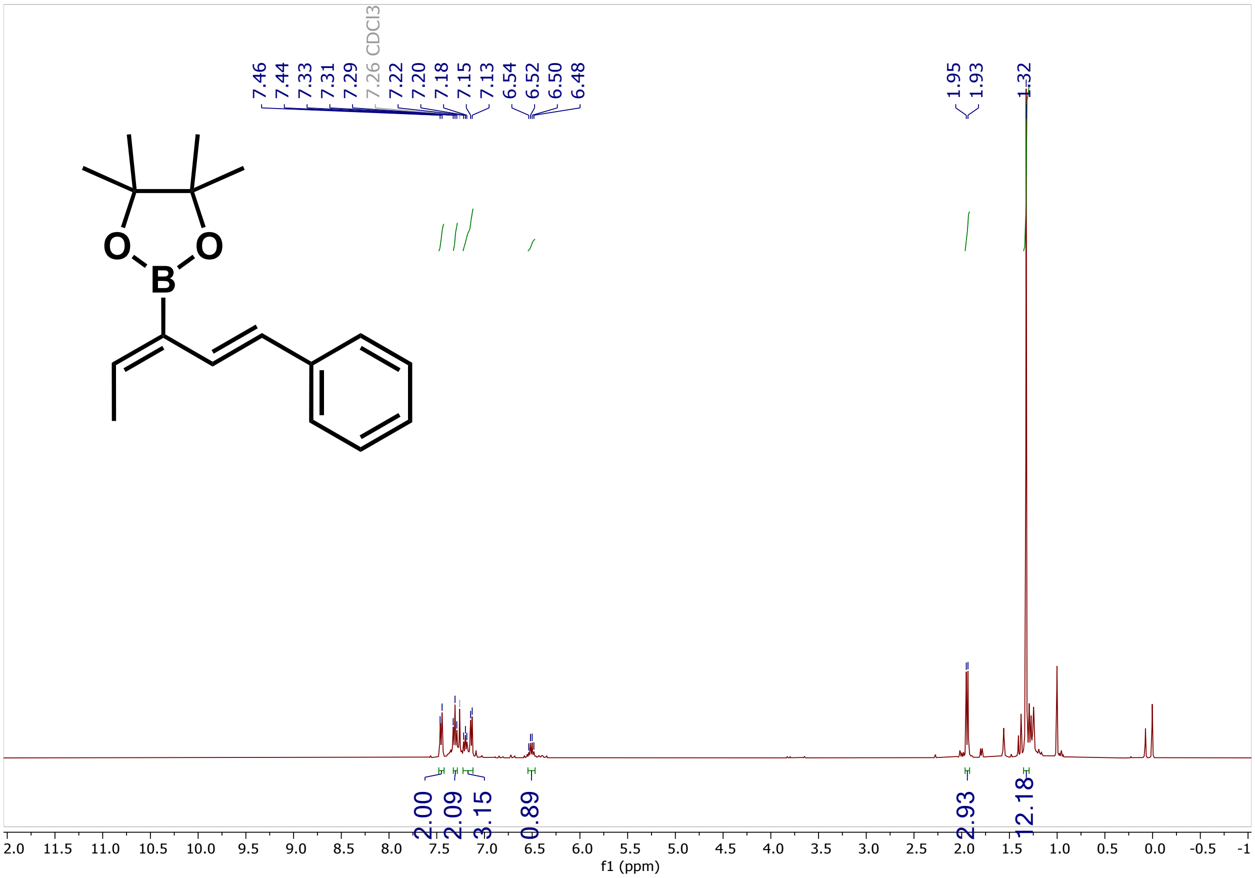

^{13}C NMR of **2v** ($CDCl_3$, 101 MHz)

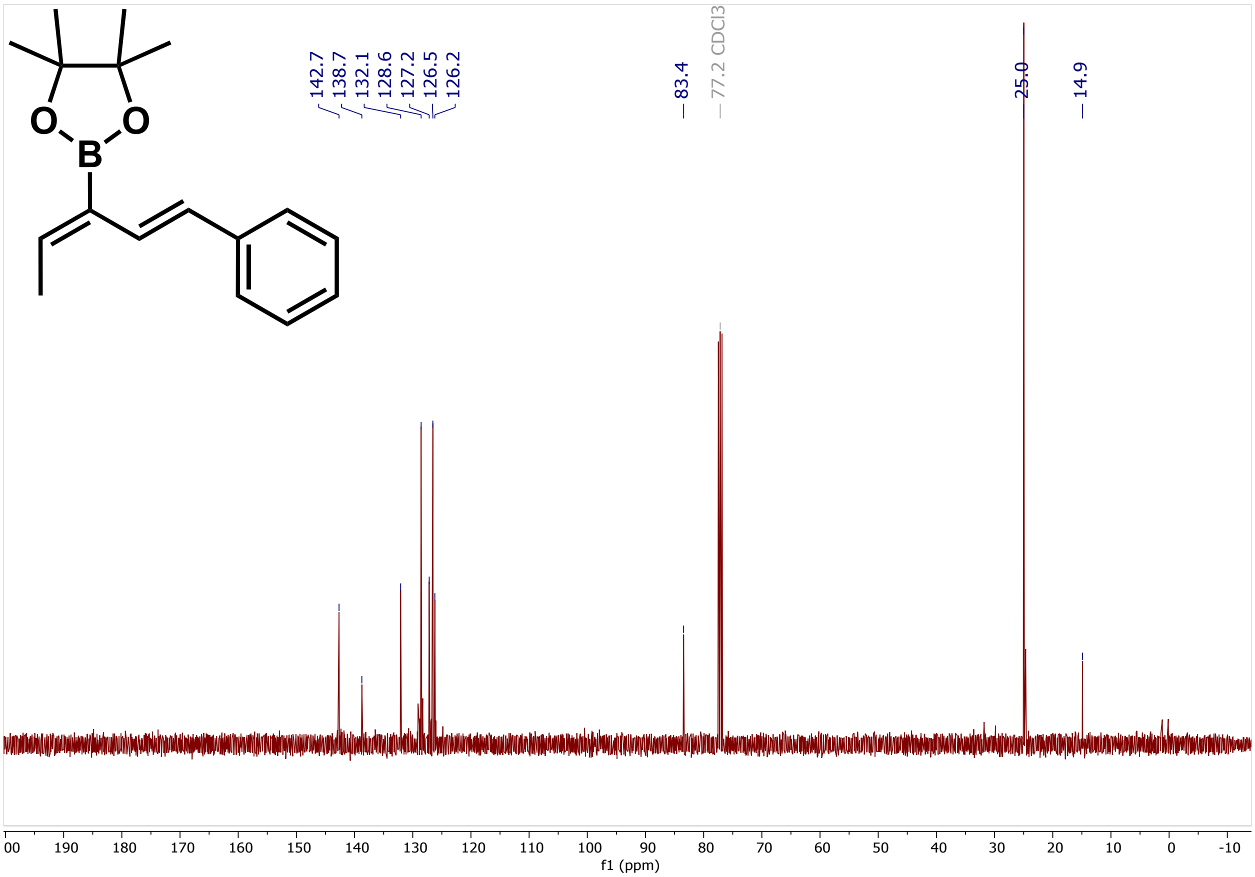

^{11}B NMR of **2v** ($CDCl_3$, 128 MHz)

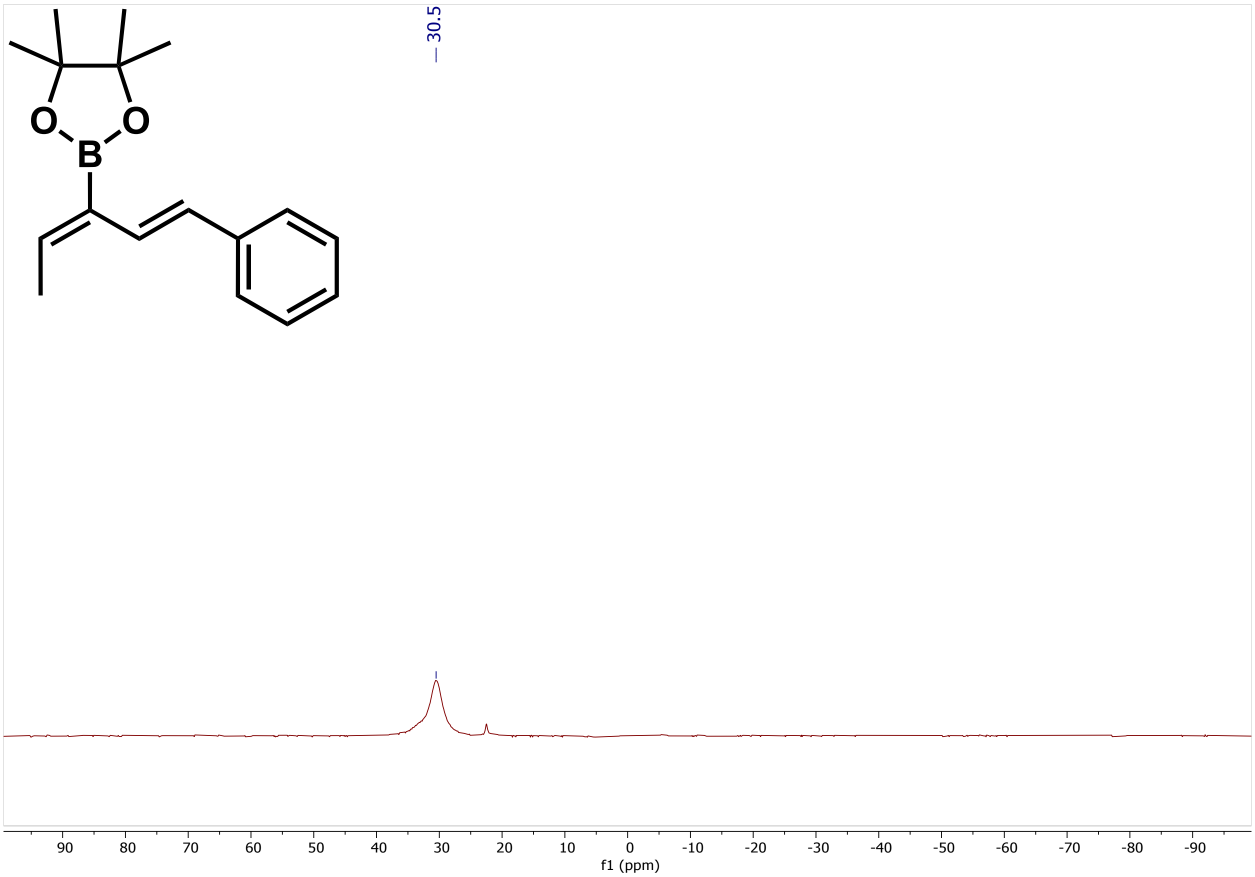

^{1}H NMR of **2w** ($CDCl_3$, 400 MHz)

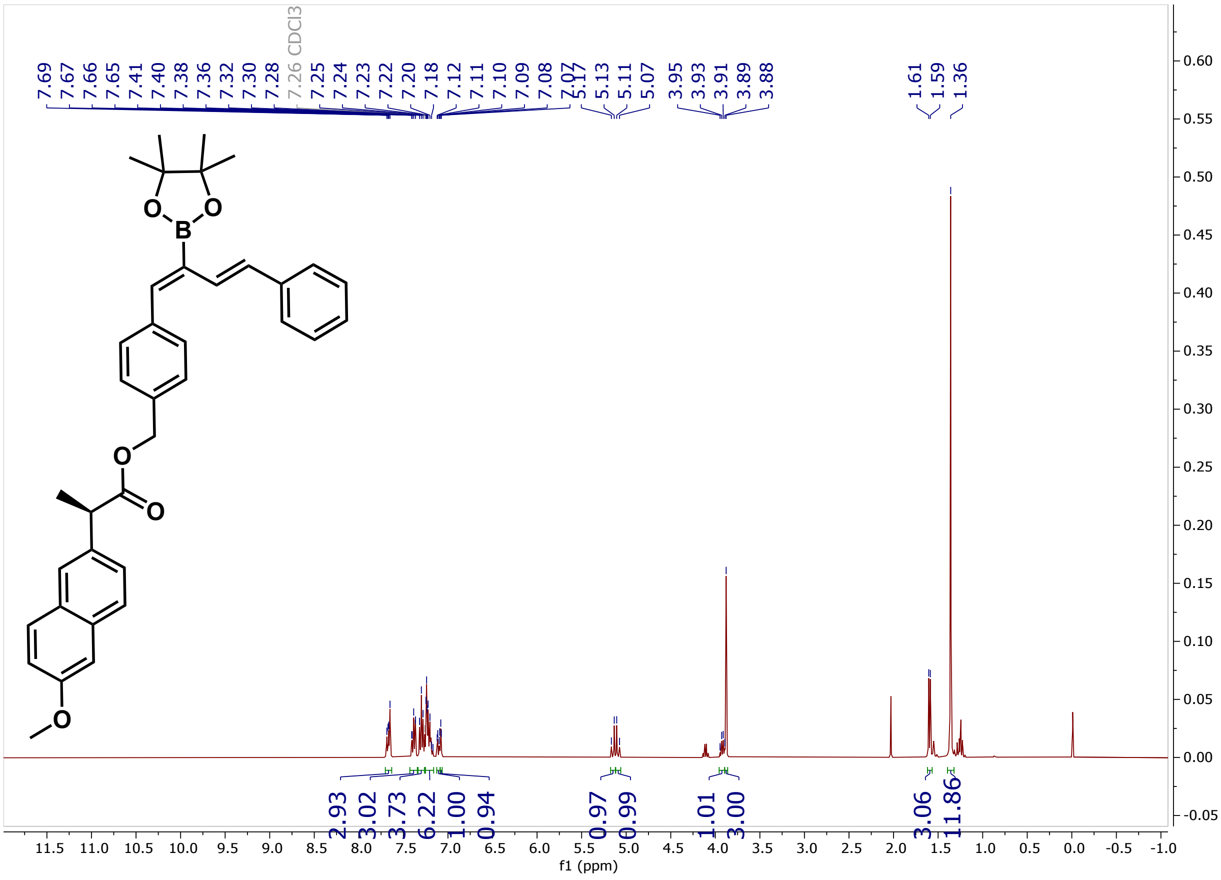

^{13}C NMR of **2w** ($CDCl_3$, 101 MHz)

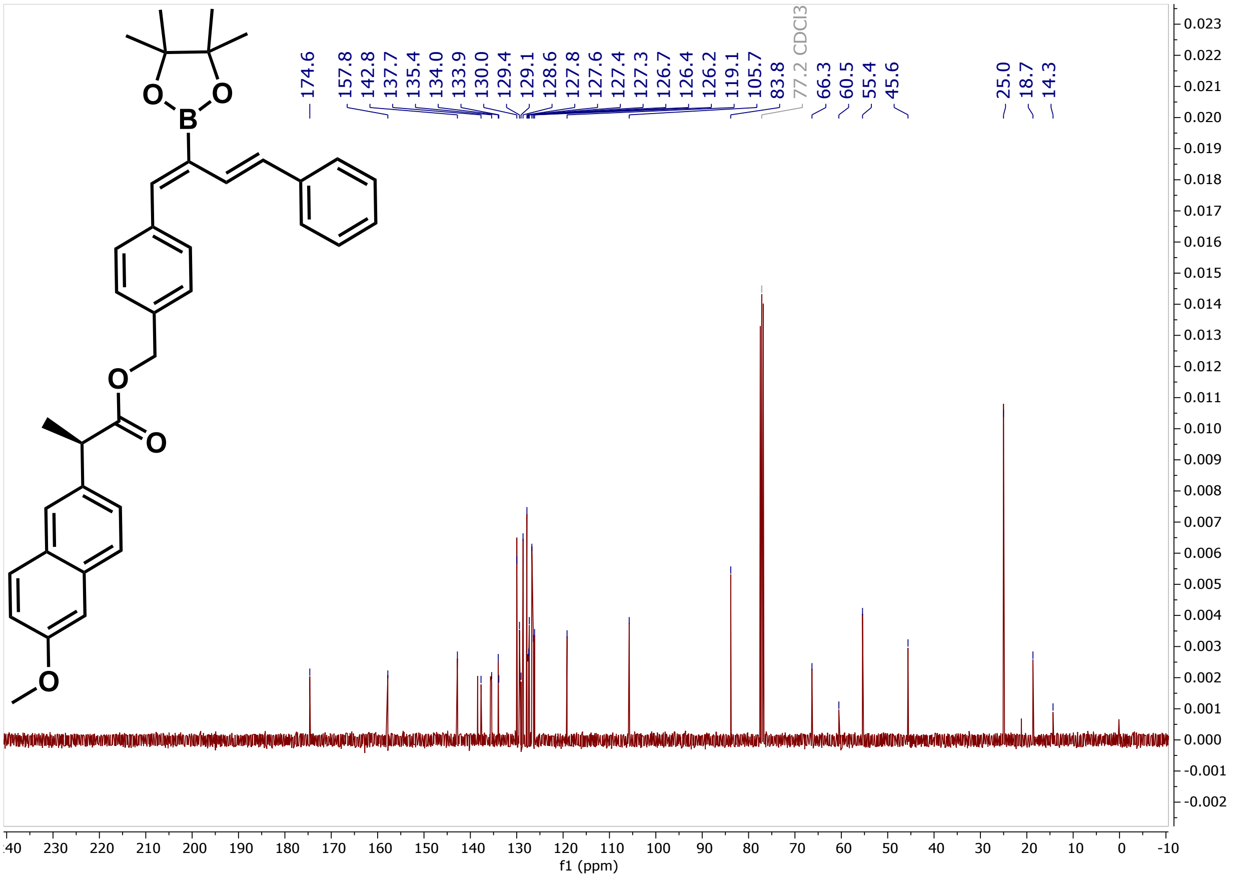

^{11}B NMR of **2w** ($CDCl_3$, 128 MHz)

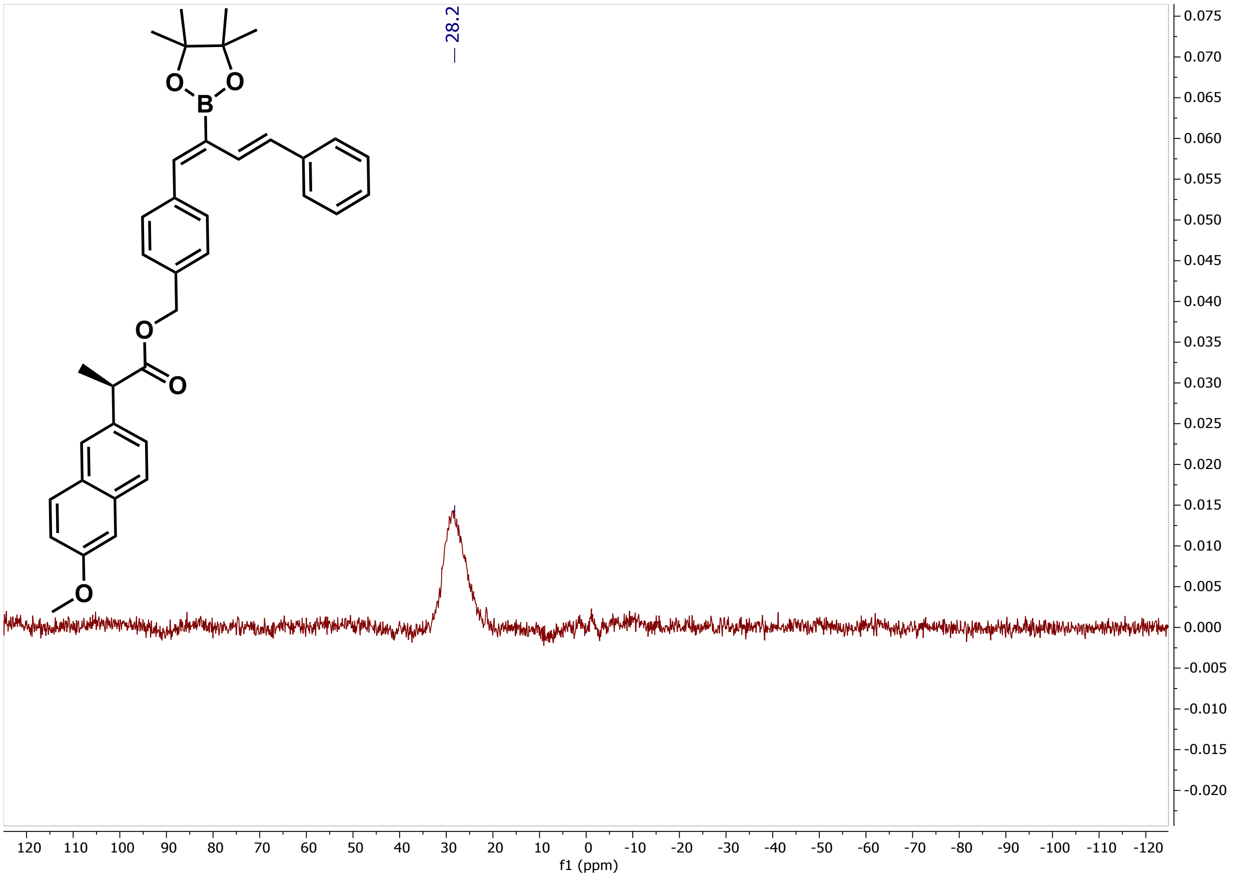

^{1}H NMR of **2x** ($CDCl_3$, 400 MHz)

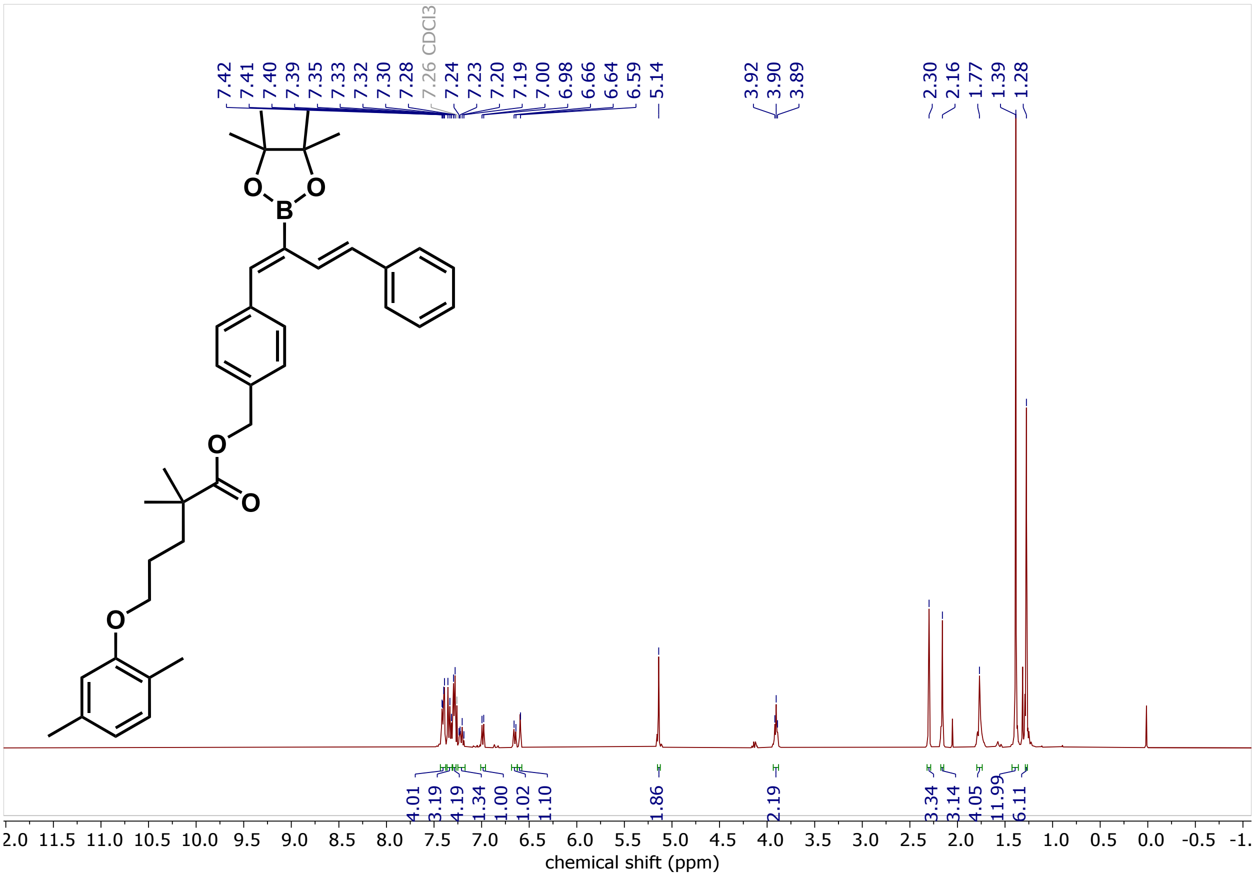

^{13}C NMR of **2x** ($CDCl_3$, 101 MHz)

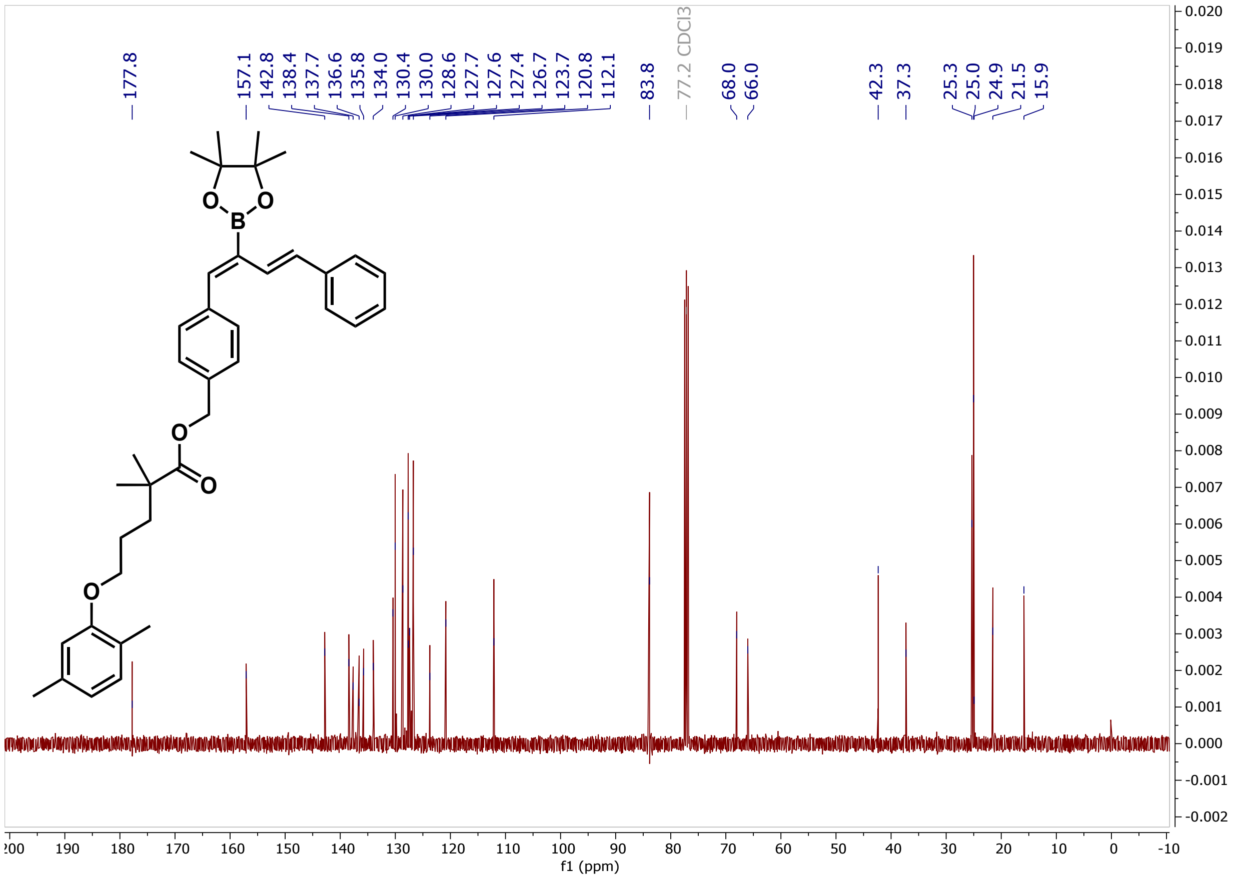

^{11}B NMR of **2x** ($CDCl_3$, 128 MHz)

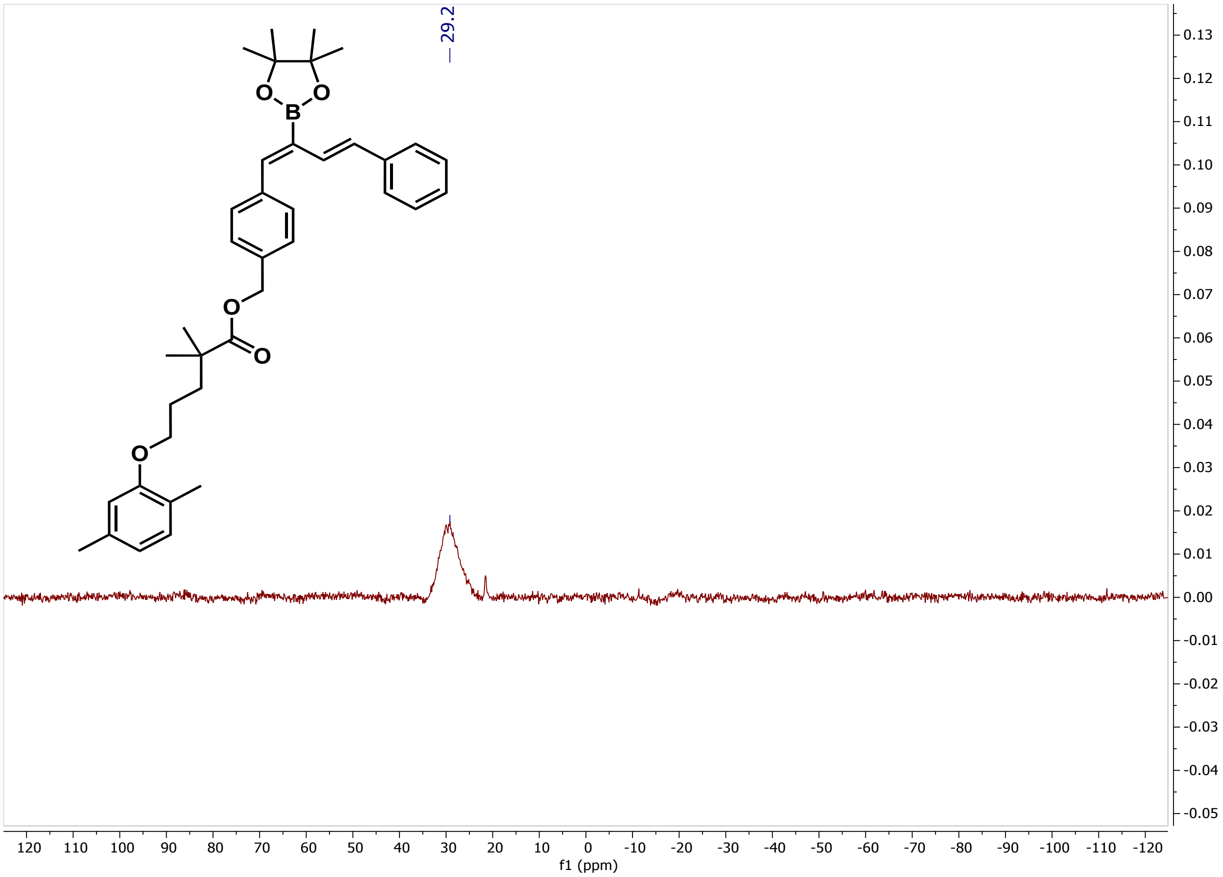

^{1}H NMR of **3** ($CDCl_3$, 400 MHz)

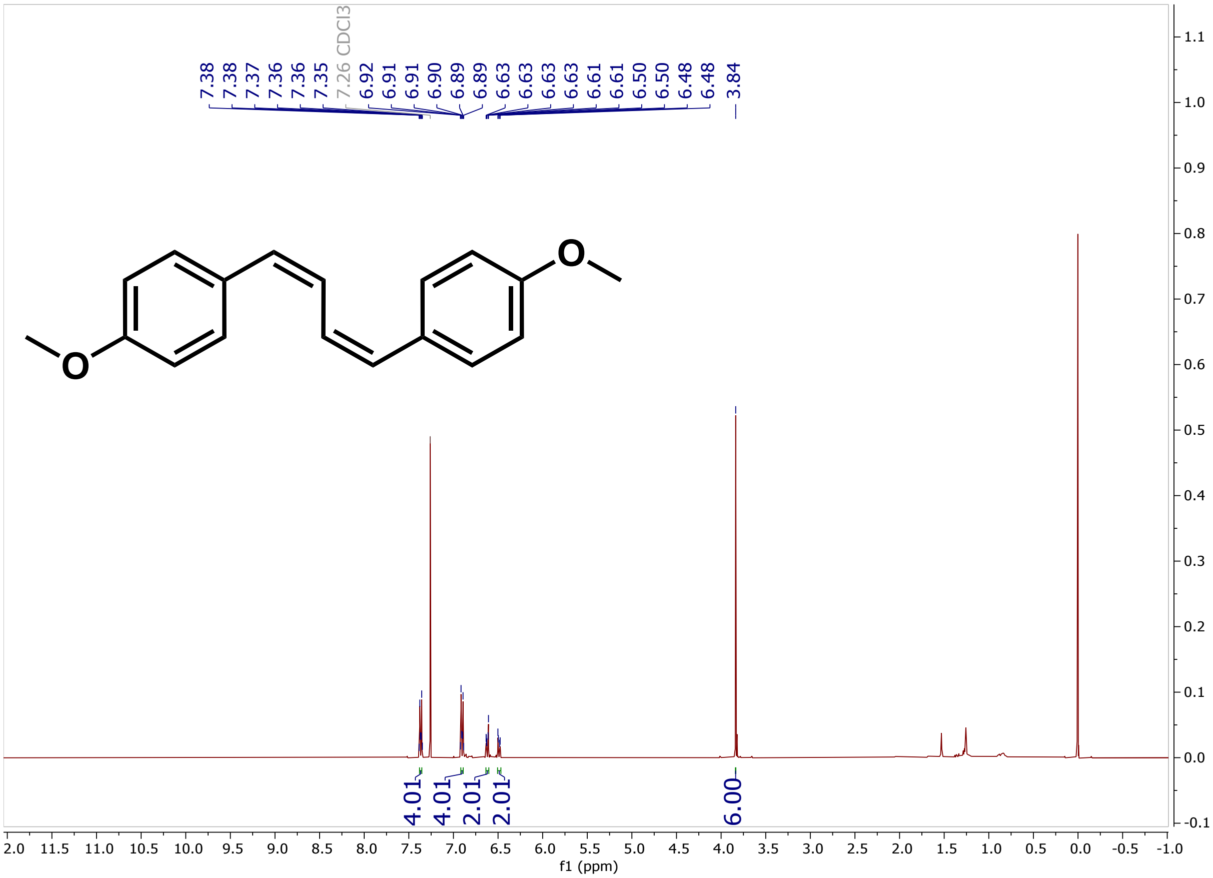

^{13}C NMR of **3** ($CDCl_3$, 101 MHz)

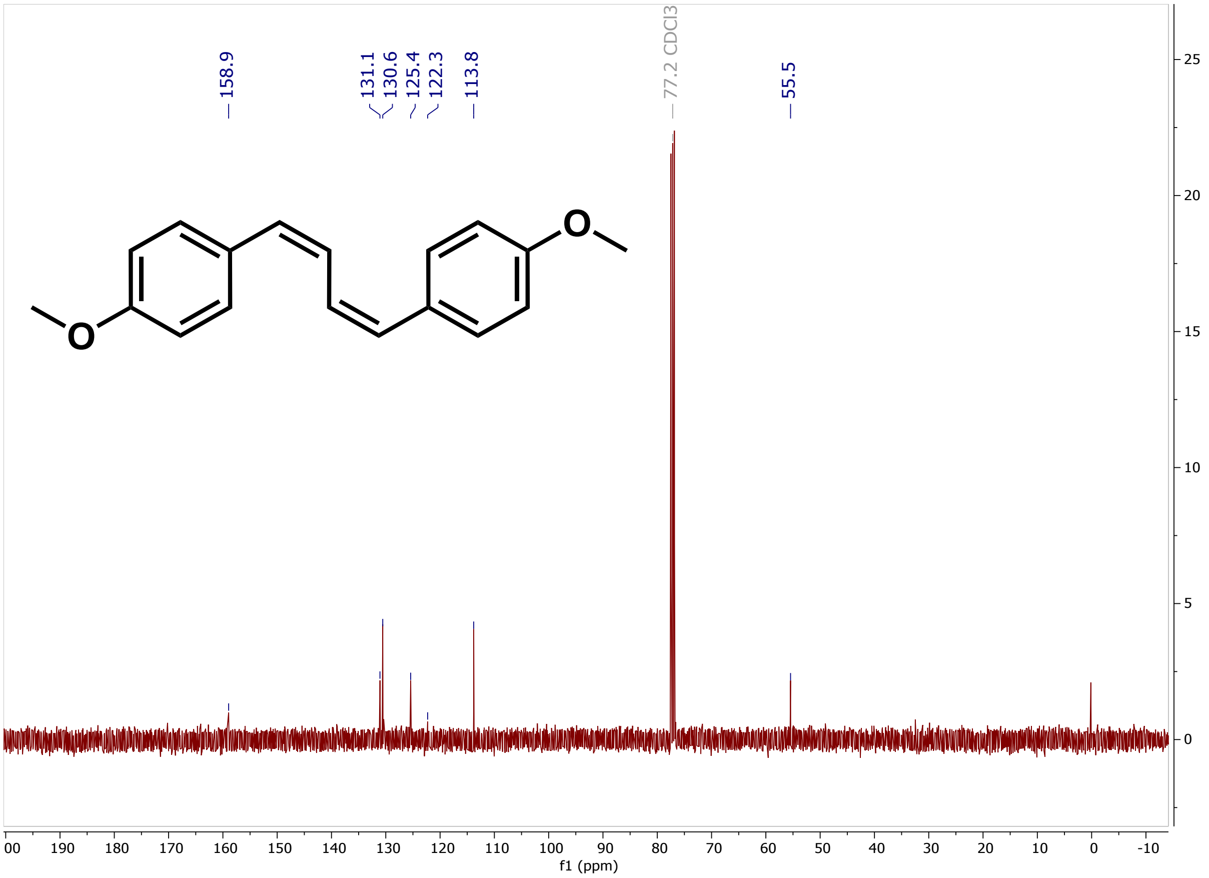

^{1}H NMR of **5** ($CDCl_3$, 400 MHz)

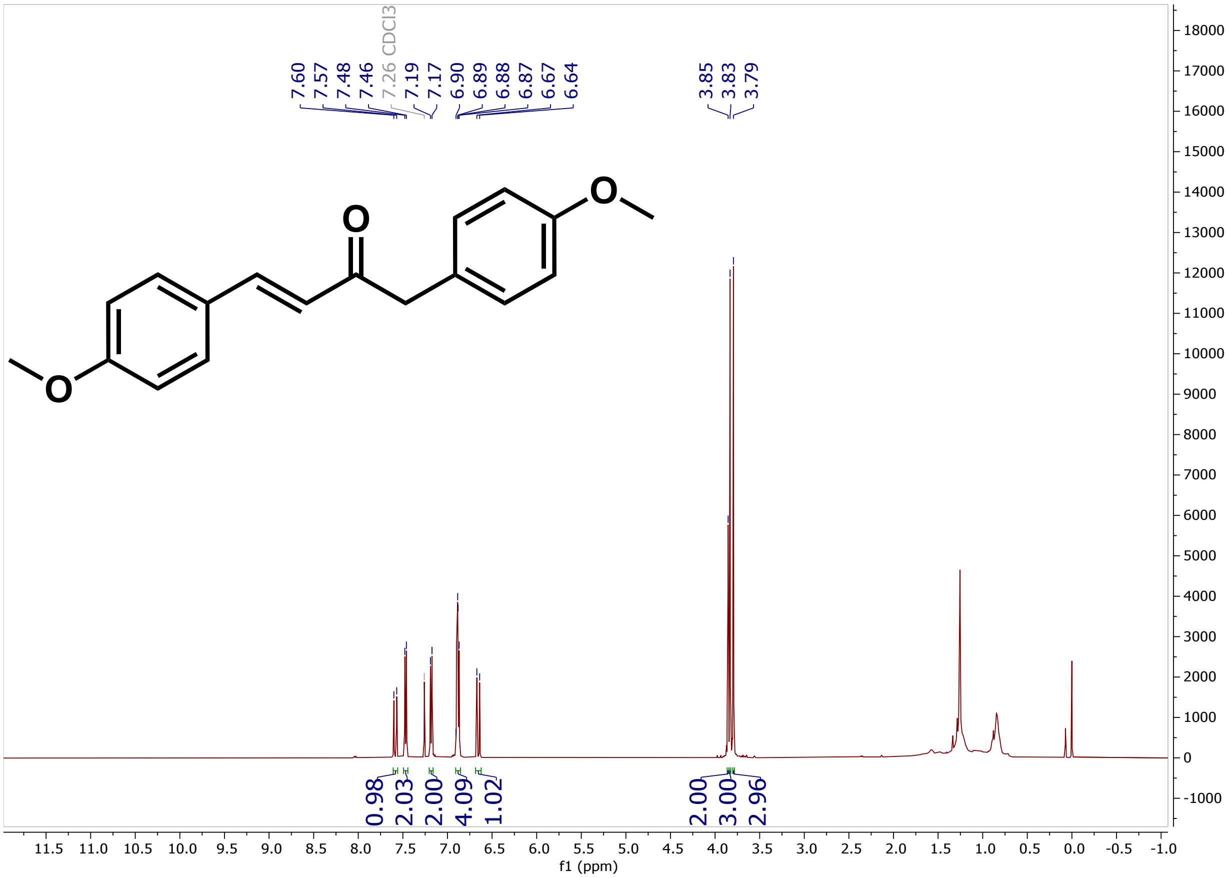

^{13}C NMR of **5** ($CDCl_3$, 101 MHz)

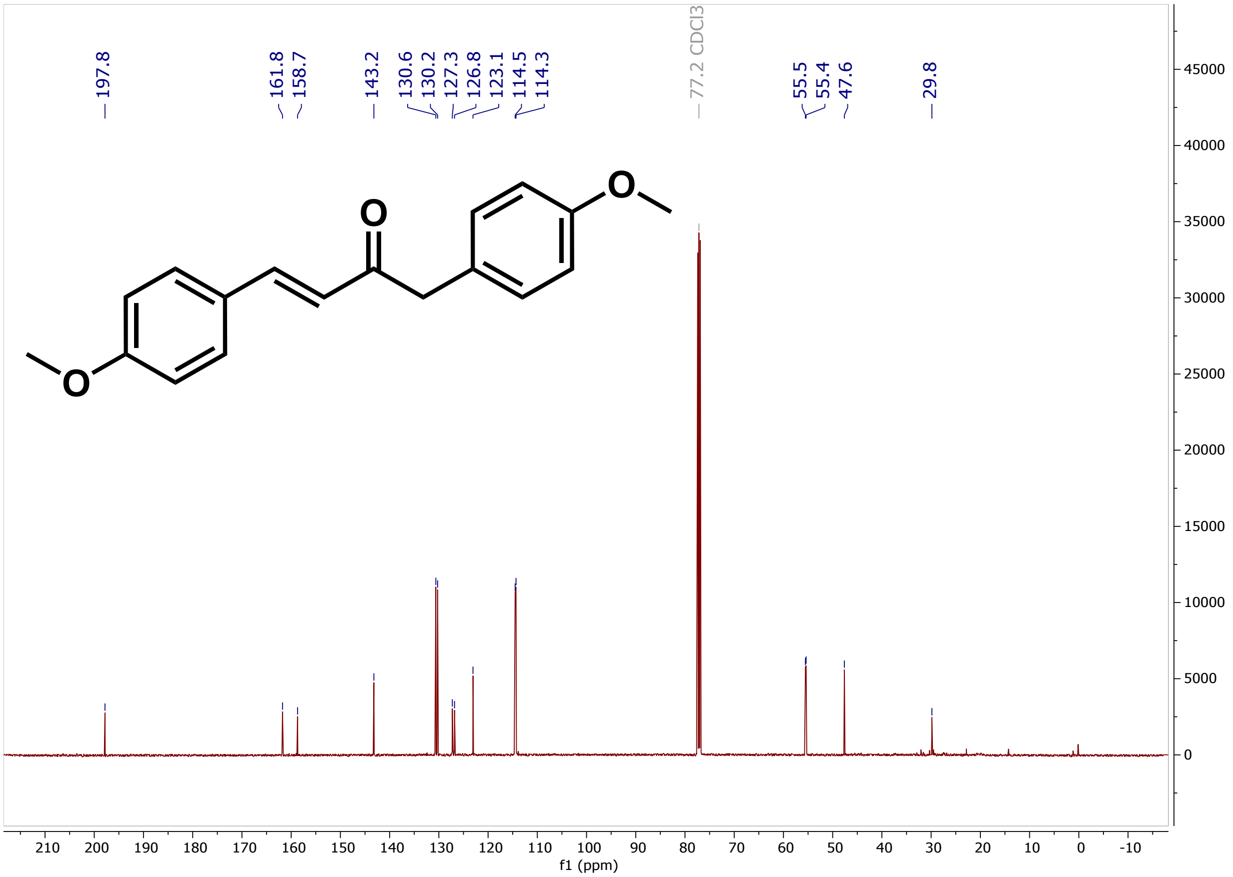

^{1}H NMR of **6** ($CDCl_3$, 500 MHz)

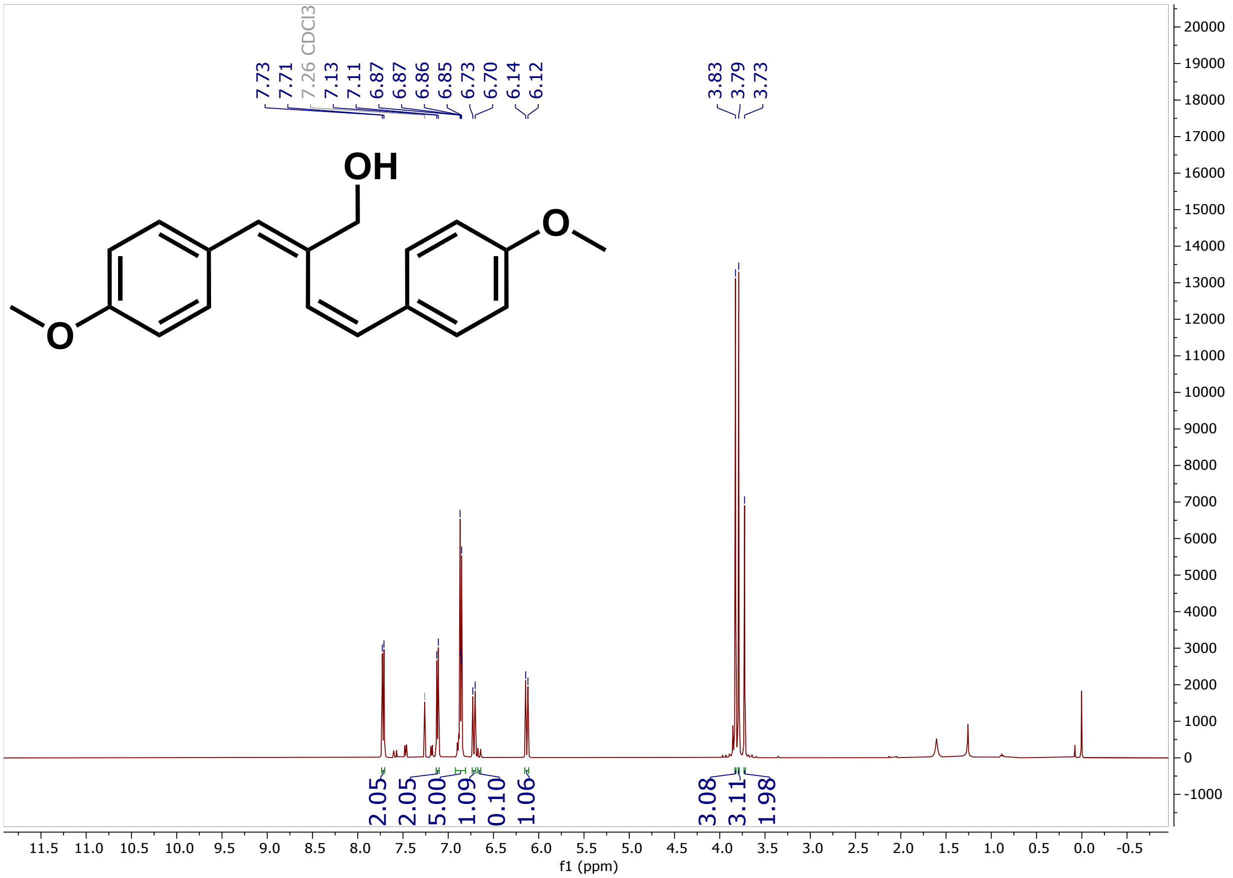

^{13}C NMR of **6** ($CDCl_3$, 126 MHz)

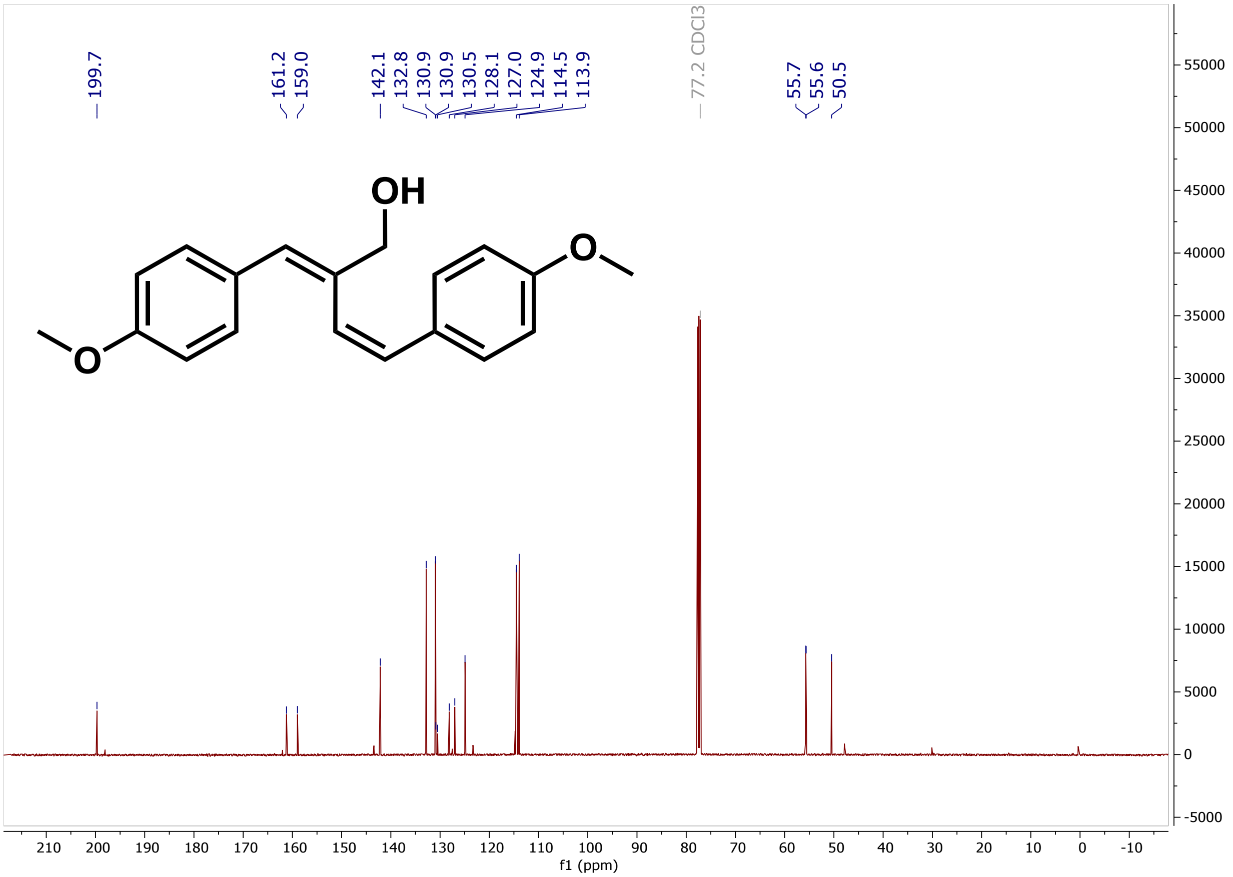

^{1}H NMR of **4** ($CDCl_3$, 500 MHz)

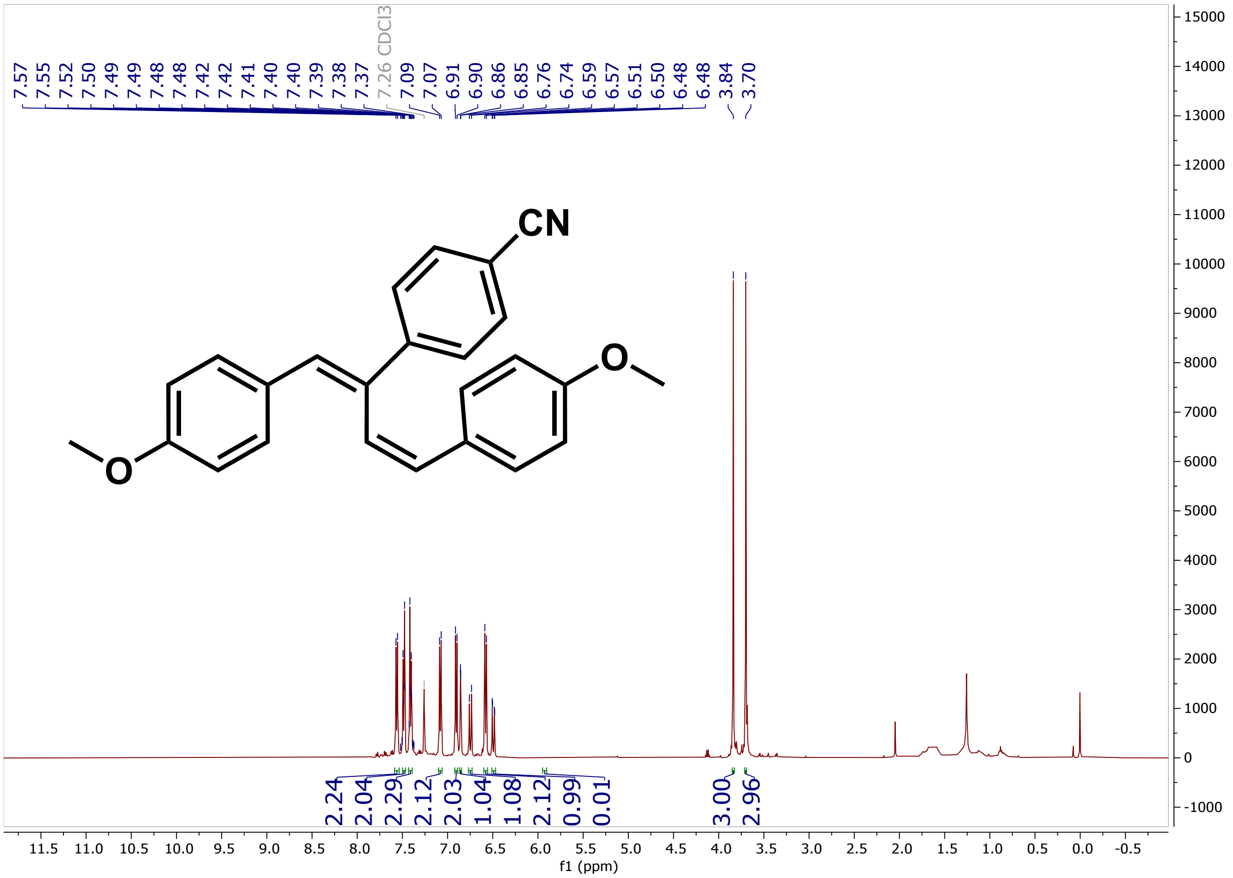

^{13}C NMR of **4** ($CDCl_3$, 126 MHz)

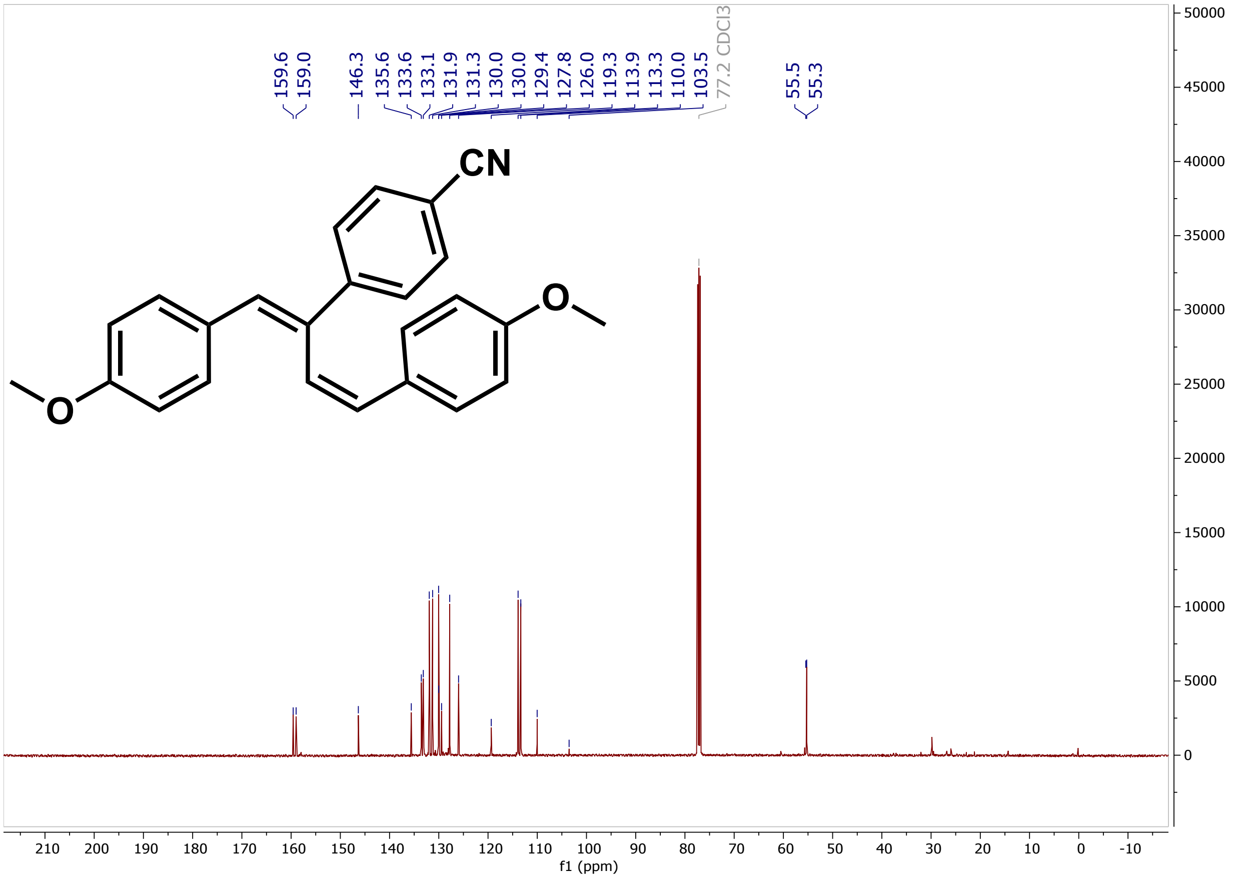

Hidden Borane Catalysis Experiment Crude ^{11}B NMR ($CDCl_3$, 128 Hz)

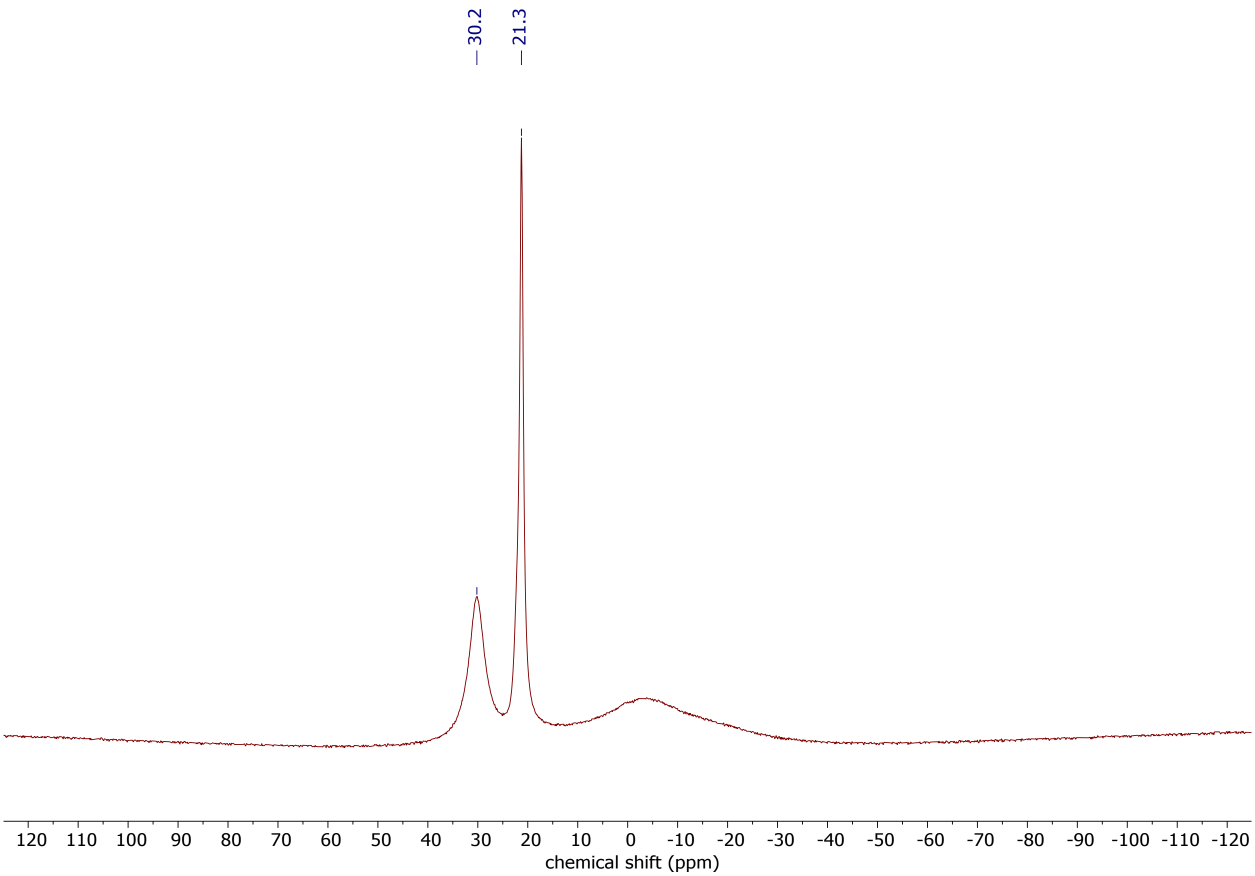

11. References

1. CrysAlisPro Software System, v1.171.42.xx, Rigaku Oxford Diffraction, **2022**, Rigaku Corporation, Oxford, UK.

2. Sheldrick, G. M. "SHELXT – Integrated space-group and crystal structure determination." *Acta Cryst*. **2015**, A71, 3–8.

3. Sheldrick, G. M. "Crystal structure refinement with SHELXL." *Acta Cryst.* **2015**, C71, 3-8.

4. Dolomanov, O.V.; Bourhis, L. J.; Gildea, R. J.; Howard, J. A. K.; Puschmann, H. *J. Appl. Cryst.* **2009**, 42, 339–341.

5. Macrae, C. F.; Sovago, I.; Cottrell, S. J.; Galek, P. T. A.; McCabe, P.; Pidcock, E.; Platings, M.; Shields, G. P.; Stevens, J. S.; Towler M.; Wood, P. A. *J. Appl. Cryst.* **2020**, 53, 226-235.
